# Update of variants identified in the pancreatic β‐cell K_ATP_ channel genes *KCNJ11* and *ABCC8* in individuals with congenital hyperinsulinism and diabetes

**DOI:** 10.1002/humu.23995

**Published:** 2020-02-17

**Authors:** Elisa De Franco, Cécile Saint‐Martin, Klaus Brusgaard, Amy E. Knight Johnson, Lydia Aguilar‐Bryan, Pamela Bowman, Jean‐Baptiste Arnoux, Annette Rønholt Larsen, May Sanyoura, Siri Atma W. Greeley, Raúl Calzada‐León, Bradley Harman, Jayne A. L. Houghton, Elisa Nishimura‐Meguro, Thomas W. Laver, Sian Ellard, Daniela del Gaudio, Henrik Thybo Christesen, Christine Bellanné‐Chantelot, Sarah E. Flanagan

**Affiliations:** ^1^ Institute of Biomedical and Clinical Science University of Exeter Medical School Exeter UK; ^2^ Department of Genetics, Pitié‐Salpêtrière Hospital, AP‐HP Sorbonne University Paris France; ^3^ Department of Clinical Genetics Odense University Hospital Odense Denmark; ^4^ Department of Human Genetics, University of Chicago Genetic Services Laboratory The University of Chicago Chicago Illinois; ^5^ Pacific Northwest Research Institute Seattle Washington; ^6^ Reference Center for Inherited Metabolic Diseases Necker‐Enfants Malades Hospital Paris France; ^7^ Hans Christian Andersen Children's Hospital Odense University Hospital Odense Denmark; ^8^ Section of Adult and Pediatric Endocrinology, Diabetes, and Metabolism, Kovler Diabetes Center University of Chicago Chicago Illinois; ^9^ Pediatric Endocrinology, Endocrine Service National Institute for Pediatrics Mexico City Mexico; ^10^ Department of Molecular Genetics Royal Devon and Exeter NHS Foundation Trust Exeter UK; ^11^ Department of Pediatric Endocrinology, Children's Hospital, National Medical Center XXI Century Instituto Mexicano del Seguro Social Mexico City Mexico; ^12^ Odense Pancreas Center Odense University Hospital Odense Denmark

**Keywords:** *ABCC8*, congenital hyperinsulinism, K‐ATP channel, *KCNJ11*, neonatal diabetes

## Abstract

The most common genetic cause of neonatal diabetes and hyperinsulinism is pathogenic variants in *ABCC8* and *KCNJ11*. These genes encode the subunits of the β‐cell ATP‐sensitive potassium channel, a key component of the glucose‐stimulated insulin secretion pathway. Mutations in the two genes cause dysregulated insulin secretion; inactivating mutations cause an oversecretion of insulin, leading to congenital hyperinsulinism, whereas activating mutations cause the opposing phenotype, diabetes. This review focuses on variants identified in *ABCC8* and *KCNJ11*, the phenotypic spectrum and the treatment implications for individuals with pathogenic variants.

## Graphical abstract

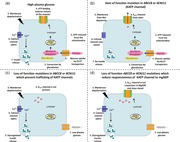

## 1 INTRODUCTION

**Figure 1:**
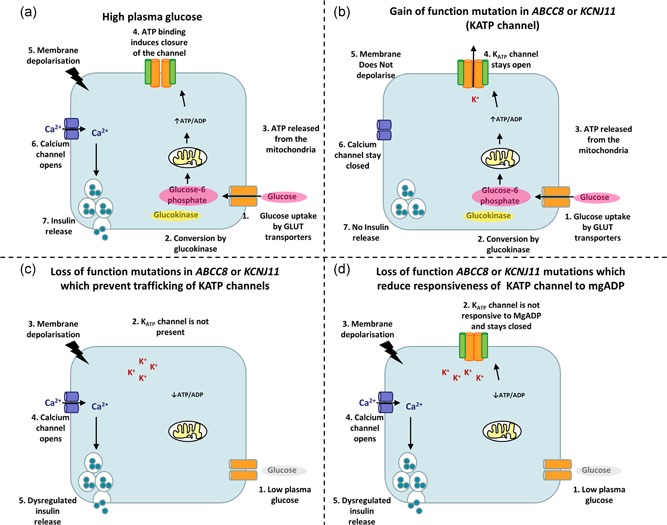
Schematic representation of insulin secretion in the pancreatic β‐cell. (a) In a normal cell in a high plasma glucose environment. (b) In a cell with an activating K_ATP_ channel mutation. (c) In a cell with an inactivating mutation resulting in the absence/reduction in protein at the membrane surface d) In a cell with an inactivating mutation that impairs the stimulatory effect of MgADP (a) Glucose is metabolized after entry into the β‐cell via a GLUT transporter. This results in change in the ATP:ADP ratio, leading to channel closure and membrane depolarization and activation of voltage‐dependent calcium channels. Calcium enters the cell, which triggers insulin release. (b) An activating mutation in a K_ATP_ channel gene results in the membrane being maintained in a hyperpolarized state. Calcium channels remain closed and insulin is not secreted. (c) Loss‐of‐function mutations can result in an absence/reduction in protein at the membrane surface. This keeps the membrane in a depolarized state, regardless of the metabolic state ultimately leading to unregulated insulin secretion. (d) Loss‐of‐function missense mutations can produce channels that traffic to the membrane but have impaired mgADP activation

ATP‐sensitive potassium (K_ATP_) channels were found to couple glucose metabolism to membrane electrical activity and insulin release over 30 years ago (Ashcroft, Harrison, & Ashcroft, 1984; Cook & Hales, 1984; Rorsman & Trube, 1985). This landmark discovery was fundamental to further understanding of the insulin secretion pathway whereby glucose metabolism results in a change in ratio of ADP and ATP. The binding of ATP to the channel induces channel closure, depolarization of the membrane, and activation of voltage‐dependent calcium channels, leading to calcium influx, and insulin granule exocytosis (Figure 1a).

Given the role of the K_ATP_ channel in insulin secretion, it is not unexpected that variants in *KCNJ11*, encoding the four pore‐forming inwardly rectifying Kir6.2 subunits, and *ABCC8*, encoding the four sulphonylurea receptor 1 (SUR1) subunits of the channel, can cause hypo‐ or hyperglycemia (Babenko et al., 2006; Gloyn, Pearson, et al., 2004; Thomas et al., 1995; Thomas, Ye, & Lightner, 1996). Identifying these mutations is important for informing prognosis, medical management, and recurrence risk.

Over recent years, the number of variants identified in these two genes has expanded tremendously. In 2006, 124 disease‐causing mutations were reported, which increased to 265 pathogenic variants 3 years later (Flanagan et al., 2009; Gloyn, Siddiqui, & Ellard, 2006). By combining published reports together with data from five international molecular genetic screening laboratories in the UK, Denmark, France, and the United States of America, we now report 953 pathogenic *ABCC8* and *KCNJ11* variants (Tables S1–S6) and discuss the role of these genes in congenital hyperinsulinism (CHI) and monogenic diabetes.

## 2 CONGENITAL HYPERINSULINISM

CHI is characterized by the inappropriate secretion of insulin despite low blood glucose, which can result in irreversible brain damage if not promptly treated (Helleskov et al., 2017). The condition has a variable phenotype usually presenting during the neonatal period or infancy with seizures and/or coma and a large birth weight due to high levels of insulin acting as a growth factor in utero.

Although most cases of CHI are sporadic, rare familial forms have been well documented. Sporadic CHI has an estimated incidence of between 1 in 27,000 and 1 in 50,000 live births (Glaser, Thornton, Otonkoski, & Junien, 2000; Otonkoski et al., 1999). However, in some isolated populations or in countries with high rates of consanguineous unions, the incidence is higher (i.e., 1 in 2,675 to 1 in 3,200; Mathew et al., 1988; Otonkoski et al., 1999).

### 2.1 CHI due to K_ATP_ channel mutations

Loss‐of‐function *ABCC8* mutations were first described in 1995 (Thomas et al., 1995). These mutations either prevent trafficking of the channel to the membrane surface or are associated with channels that reach the surface but are not fully responsive to MgADP activation (Figure 1; Ashcroft, 2005; Nichols et al., 1996; Taschenberger et al., 2002). The majority of *ABCC8* loss‐of‐function mutations are recessively acting with a small number of dominant missense mutations reported that produce channels that traffic to the membrane but have impaired mgADP activation.

Fewer loss‐of‐function mutations have been reported in *KCNJ11* in keeping with the gene being much smaller (1173 vs. 4749 bases, respectively; Thomas et al., 1996). Similar to *ABCC8*, both dominant and recessively acting *KCNJ11* mutations have been described (Pinney et al., 2013). Mutations in these two genes together account for 36–70% of CHI cases (Kapoor et al., 2013; Snider et al., 2013).

There exist mouse models for K_ATP_ channel CHI; however, their inability to fully recapitulate the human phenotype means that they have a limited value for studying specific disease mechanisms. For example, mice generated with a deletion of *ABCC8* or *KCNJ11*, or the homozygous recessive *KCNJ11* mutation p.(Tyr12Ter), do not have the sustained neonatal hypoglycemia observed in humans with homozygous null mutations. Instead the blood glucose levels normalize in the mouse within a few days of birth with glucose intolerance developing in later life (Hugill, Shimomura, Ashcroft, & Cox, 2010; Miki et al., 1998; Seghers, Nakazaki, DeMayo, Aguilar‐Bryan, & Bryan, 2000). The differences in the phenotype between mice and humans are not fully understood, but they highlight the need to develop human‐specific models for studying disease mechanisms.

### 2.2 Clinical management of K_ATP_ channel CHI

In 2015, the Pediatric Endocrine Society published recommendations for the evaluation and management of persistent hypoglycemia in neonates, infants, and children (Thornton et al., 2015). The main treatment for CHI is the K_ATP_ channel‐opener diazoxide; however, patients with *ABCC8/KCNJ11* mutations that prevent trafficking to the membrane do not respond to the drug as diazoxide targets the SUR1 subunit of the K_ATP_ channel. For approximately 50% of patients with mutations that do not prevent the channel from reaching the membrane, diazoxide is an effective treatment (Boodhansingh et al., 2019). For patients with diazoxide‐unresponsive CHI, second‐line treatment with somatostatin analogs may be helpful to control hypoglycemia; however, adverse effects on somatostatin analogs, and likewise diazoxide, have been reported (Demirbilek et al., 2014; Herrera et al., 2018).

The mode of inheritance of the K_ATP_ channel mutation determines the pancreatic histological subtype (de Lonlay et al., 1997; de Lonlay et al., 2002; Jack, Walker, Thomsett, Cotterill, & Bell, 2000; Rahier et al., 1984). Inheritance of two recessively acting or one dominant *ABCC8/KCNJ11* mutation results in diffuse disease affecting the entire pancreas. Focal disease is caused by somatic loss of the maternal chromosome 11p15.5 region by uniparental disomy that unmasks a paternally inherited K_ATP_ channel mutation at 11p15.1. These focal lesions often appear histologically as small regions of islet adenomatosis that develop as a result of the imbalanced expression of maternally imprinted tumor suppressor genes H19 and p57^Kip2^, and the increased expression of the paternally derived insulin‐like growth factor II gene (Craigie et al., 2018; Damaj et al., 2008; de Lonlay et al., 1997). Rarely, giant focal lesions have been described where virtually the whole of the pancreas is affected (Ismail et al., 2012). Atypical mosaic disease has also been reported in a small number of cases (Han et al., 2017; Houghton et al., 2019; Hussain et al., 2008; Sempoux et al., 2011).

The identification of a single recessively acting K_ATP_ channel mutation in an individual with CHI predicts focal disease with 84–97% sensitivity, with a positive predictive value up to 94% (Mohnike et al., 2014; Snider et al., 2013). ^18^F‐DOPA PET/CT scanning can identify and localize a focal lesion before surgery (Otonkoski et al., 2006). Intraoperative ultrasound may further aid the surgeon to perform tissue‐sparing pancreatic resection in focal CHI, which is potentially curative (Bendix et al., 2018).

## 3 DIABETES MELLITUS

Diabetes is the opposing disorder to CHI and results from hyper‐ rather than hypoglycemia. Current estimates suggest that approximately 0.4% of all diabetes (and up to 3.5% of those diagnosed under 30 years of age) has a monogenic cause (Shepherd et al., 2016; Shields et al., 2017). Individuals diagnosed with monogenic diabetes outside of infancy are generally classified as having maturity onset diabetes of the young, whereas neonatal diabetes (NDM) describes congenital diabetes. In individuals with NDM, impaired insulin secretion results in a low birth weight and hyperglycemia diagnosed before the age of 6 months (Hattersley & Ashcroft, 2005). The minimal incidence of NDM has been calculated to be between 1 in 89,000 and 1 in 160,949 live births (Grulich‐Henn et al., 2010; Wiedemann et al., 2010).

### 3.1 Later‐onset diabetes due to K_ATP_ channel mutations

Dominantly acting mutations in the K_ATP_ channel genes have been rarely described in individuals with later‐onset diabetes in the absence of documented hyper‐ or hypoglycemia in the neonatal period (Bowman et al., 2012; Hartemann‐Heurtier et al., 2009; Huopio et al., 2003; Koufakis et al., 2019; Tarasov et al., 2008). The mechanism(s) leading to this variable penetrance are not fully understood and may differ according to whether the mutation is causing a gain or loss of channel function. Interestingly, in one study, the generation of a mouse model harboring a homozygous dominantly acting loss‐of‐function *ABCC8* mutation p.(Glu1507Lys) recapitulated the biphasic phenotype with the mice having increased insulin secretion in early life and reduced insulin secretion later on. This was shown to be resulting from a reduction in insulin content rather than a reduction of islet number and/or size. Heterozygosity for the mutation did, however, not result in a phenotype in the mouse, further highlighting differences between the mouse models and human disease (Shimomura et al., 2013).

### 3.2 Neonatal diabetes due to K_ATP_ channel mutations

Strong support for the role of gain‐of‐function K_ATP_ channel mutations in the etiology of diabetes came from the observation that mice overexpressing a mutant K_ATP_ channel with reduced ATP sensitivity developed diabetes within 2 days (Koster, Marshall, Ensor, Corbett, & Nichols, 2000). In 2004, the first heterozygous activating *KCNJ11* mutations causing NDM were described in humans with activating *ABCC8* mutations reported 2 years later (Babenko et al., 2006; Gloyn, Pearson, et al., 2004; Proks et al., 2006). Mutations in these two genes together have now been shown to account for approximately 40% of NDM cases (De Franco et al., 2015; Stoy et al., 2008).

Both dominant and recessive activating mutations are frequently identified in *ABCC8*. Conversely for *KCNJ11*, all, but one of the mutations reported so far, p.(Gly324Arg), have been dominantly acting. The majority (~60%) of dominant mutations arise “de novo,” so there is often no family history of diabetes; however, germline mosaicism has been observed in some families (Edghill et al., 2007; Gloyn, Cummings, et al., 2004).

There is added complexity associated with *ABCC8* mutations, as compound heterozygosity for both an activating and an inactivating mutation can cause diabetes (Ellard et al., 2007). Furthermore, a recessively inherited *ABCC8* nonsense variant has been reported in two cases with NDM, which leads to the deletion of the in‐frame exon 17 likely resulting in enhanced sensitivity of the channel to intracellular MgADP/ATP (Flanagan et al., 2017).

The specific K_ATP_ channel mutation identified determines whether the diabetes will cause permanent or transient NDM (Gloyn, Reimann, et al., 2005; Patch, Flanagan, Boustred, Hattersley, & Ellard, 2007). Variable penetrance within families with mutations leading to transient diabetes is observed with some individuals being diagnosed with diabetes at birth, yet others developing diabetes for the first time in adulthood (see previous section on adult‐onset diabetes; Flanagan, Edghill, Gloyn, Ellard, & Hattersley, 2006).

### 3.3 Spectrum of central nervous system features in K_ATP_ channel NDM

Central nervous system (CNS) features are frequently reported in individuals with K_ATP_ channel NDM due to the Kir6.2 and SUR1 proteins being expressed in the brain (Karschin, Ecke, Ashcroft, & Karschin, 1997; Liss, Bruns, & Roeper, 1999; Sakura, Ammala, Smith, Gribble, & Ashcroft, 1995; Schmahmann & Sherman, 1998). The most severe neurological phenotype is termed as developmental delay, epilepsy and neonatal diabetes (DEND) syndrome, which includes muscle weakness and hypotonia (Hattersley & Ashcroft, 2005). Intermediate DEND (iDEND) syndrome is diagnosed when epilepsy is absent or presents after the age of 12 months (Gloyn, Diatloff, et al. 2006). Clinical studies have reported CNS features in approximately 20–30% of individuals with K_ATP_ channel permanent NDM (De Franco et al., 2015; Massa et al., 2005; Sagen et al., 2004).

Since these initial reports, studies on larger cohorts of individuals affected with K_ATP_ channel NDM have characterized the neurological features in more detail. Additional features reported include autism and attention deficit hyperactivity disorder (ADHD), anxiety and sleep disorders, dyspraxia, and learning difficulties, resulting in impaired attention, memory, visuospatial abilities, and executive function (Beltrand et al., 2015; Bowman et al., 2016; Bowman et al., 2017; Bowman, Day, et al., 2018; Busiah et al., 2013; Landmeier, Lanning, Carmody, Greeley, & Msall, 2017). More important, it is now recognized that some degree of impairment can be detected on neuropsychological testing in the majority of patients with K_ATP_ channel mutations, even if there is no obvious CNS involvement (Busiah et al., 2013; Carmody et al., 2016).

### 3.4 Clinical management of neonatal diabetes and CNS features due to K_ATP_ channel mutations

The identification of a K_ATP_ channel mutation can have an impact on the medical management of patients with NDM as approximately 90% can transfer from insulin injections to high‐dose sulphonylurea tablets (Pearson et al., 2006; Zung, Glaser, Nimri, & Zadik, 2004). Sulphonylureas bind to the SUR1 subunit of the K_ATP_ channel and close it independently of ATP, resulting in excellent long‐term glycemic control and improved quality of life for affected patients and their families (Babenko et al., 2006; Bowman, Sulen et al., 2018; Rafiq et al., 2008). Patients who are unable to transfer to sulphonylureas tend to have a longer duration of diabetes before attempting transfer or functionally severe mutations (Babiker et al., 2016; Thurber et al., 2015). Few side effects and no episodes of severe hypoglycemia involving seizures or loss of consciousness have been reported in individuals with sulphonylurea‐treated neonatal diabetes (Bowman, Sulen, et al., 2018; Codner, Flanagan, Ellard, Garcia, & Hattersley, 2005; Kumaraguru et al., 2009; Lanning et al., 2018).

Sulphonylureas can improve the neurological features in people with K_ATP_ channel NDM, particularly in the first year of treatment (Beltrand et al., 2015; Fendler et al., 2013; Stoy et al., 2008). However, these features do not fully resolve after sulphonylurea therapy and persist for a long term into adulthood (Bowman, Day et al., 2018; Bowmen, Sulen, et al., 2018). Higher doses of sulphonylureas are recommended for patients with severe neurological features in an attempt to mitigate this (https://www.diabetesgenes.org/). In addition, starting sulphonylurea therapy as early as possible after a genetic diagnosis is crucial as the largest improvements appear to occur in younger patients (Beltrand et al., 2015; Shah, Spruyt, Kragie, Greeley, & Msall, 2012).

## 4 **GENETIC VARIATION IN** *ABCC8* **AND** *KCNJ11*

*KCNJ11* (MIM# 600937) is located 4.5Kb from *ABCC8* on chromosome 11p15.1 and has a single exon encoding for the 390‐amino acid Kir6.2 protein (GenBank NM_000525.3). *ABCC8* consists of 39 exons that encode for the 1,582 amino acids of SUR1 (NM_001287174.1; MIM# 600509). This gene has an alternatively spliced recognition site at the 5′ end of exon 17, which results in two different transcripts differing in length by a single amino acid (GenBank AH003589.2). This alternative splicing has led to discrepancies in the literature for nomenclature of variants present in 17–39, which differ by a single amino acid depending on the isoform used (1581 amino acids, NM_000249.3 and 1582 amino acids, NM_001287174.1). For the purpose of this review, we have described *ABCC8* variants according to the longer isoform (NM_001287174.1).

### 4.1 Disease‐causing variants

A total of 748 *ABCC8* and 205 *KCNJ11* pathogenic or likely pathogenic variants have been identified in individuals with CHI or NDM (Table 1 and Table 3 and Tables S1 and S4) — please note that these tables are meant to direct to the appropriate references and laboratories. They do not provide in‐depth clinical information and variants that had been previously reported as pathogenic with a GnomAD frequency compatible with the disease frequency (as calculated by http://cardiodb.org/allelefrequencyapp/ using a biallelic mode of inheritance, a prevalence of 1/50,000, an allelic heterogeneity of 0.1, genetic heterogeneity of 0.5, and penetrance of 0.5) were not re‐assessed.

**Table 1:** Unpublished pathogenic variants identified in *KCNJ11* (NM_000525.3)

Protein change	Nucleotide change	Mutation type	Phenotype	Zygosity	Likely dominant or recessively acting	GnomAD MAF	Reporting laboratory
p.(Arg4Cys)	c.10C>T	Missense	TNDM PNDM	Heterozygous	Dominant	0.00002150	Exeter
p.(Leu17Pro)	c.50T>C	Missense	PNDM	Heterozygous^denovo^	Dominant	0	Exeter
p.(Tyr26Ter)	c.78C>A	Nonsense	HI	Homozygous	Recessive	0	Exeter
p.(Arg27Cys)	c.79C>T	Missense	HI	Heterozygous^Pat^	Recessive	0.000007976	Chicago
p.(Lys38Glu)	c.112A>G	Missense	HI	Homozygous	Recessive	0	Exeter
p.(Gly40Ala)	c.119G>C	Missense	HI	Homozygous	Recessive	0	Exeter
p.(Ile49Phe)	c.145A>T	Missense	TNDM	Heterozygous^denovo^	Dominant	0	Exeter
p.(Glu51Gly)	c.152A>G	Missense	PNDM	Heterozygous^denovo^	Dominant	0	Exeter
p.(Arg54Cys)	c.160C>T	Missense	HI/ Later‐onset diabetes	Homozygous/ Heterozygous	Recessive/ Dominant	0.000007078	Exeter/ Paris
p.(Leu56Gly)	c.166_167delinsGG	Missense	HI	Homozygous	Recessive	0	Exeter
p.(Thr62SerfsTer68)	c.185del	Frameshift	HI	Homozygous	Recessive	0	Exeter
p.(Cys81AlafsTer49)	c.240del	Frameshift	HI	Heterozygous^Pat^	Recessive	0	Exeter
p.(Asp99Tyr)	c.295G>T	Missense	HI	Heterozygous^denovo^	Dominant	0	Paris
p.(Ala120CysfsTer7)	c.356dup	Frameshift	HI	Homozygous	Recessive	0	Exeter
p.(Val129Met)	c.385G>A	Missense	NDM	Heterozygous^denovo^	Dominant	0	Exeter
p.(Gly132TyrfsTer10)	c.390_393dup	Frameshift	HI	Homozygous	Recessive	0	Exeter
p.(Cys166Trp)	c.498C>G	Missense	NDM	Heterozygous	Not known	0	Chicago
p.(Met169Thr)	c.506T>C	Missense	PNDM	Heterozygous^denovo^	Dominant	0	Exeter
p.(Ala178LeufsTer11)	c.532del	Frameshift	HI	Heterozygous^Pat^	Recessive	0	Exeter
p.(Glu179Lys)	c.535G>A	Missense	TNDM	Heterozygous^denovo^	Dominant	0	Exeter
p.(Arg206His)	c.617G>A	Missense	Later‐onset diabetes/HI	Heterozygous/ Heterozygous^denovo^ /Heterozygous^Pat^	Not known/ Dominant/ Not known	0	Paris/Paris/ Odense
p.(Ser208Thr)	c.623G>C	Missense	HI	Heterozygous^denovo^	Dominant	0	Exeter
p.(Tyr258Ter)	c.774C>A	Nonsense	HI	Heterozygous^Pat^	Recessive	0	Exeter
p.(His259MetfsTer61)	c.775del	Missense	HI	Homozygous	Recessive	0	Exeter
p.(Gln279Ter)	c.835C>T	Nonsense	HI	Homozygous	Recessive	0	Exeter
p.(Gln289Ala)	c.866G>C	Missense	HI	Heterozygous^Pat^	Recessive	0	Chicago
p.(Gly295Ser)	c.883G>A	Missense	HI	Homozygous	Recessive	0	Paris
p.(Val328Met)	c.982G>A	Missense	TNDM	Heterozygous	Dominant	0	Exeter
p.(Tyr330Asn)	c.988T>A	Missense	TNDM	Heterozygous	Dominant	0	Exeter
p.(Tyr330His)	c.988T>C	Missense	Diabetes	Heterozygous	Not known	0	Chicago
p.(Ser331Pro)	c.991T>C	Missense	PNDM	Heterozygous^denovo^	Dominant	0	Exeter
p.(Gly334Ser)	c.1000G>A	Missense	PNDM	Heterozygous	Dominant	0	Exeter
p.(Gly334Arg)	c.1000G>C	Missense	PNDM	Heterozygous^denovo^	Dominant	0	Exeter

*Note*: The phenotype column highlights a new phenotype; the reporting laboratory column indicates which laboratory has identified the variant in a patient with the new phenotype. See Supporting Information data for details of inclusion criteria for variants in this table.

Abbreviations: HI, hyperinsulinism; PNDM,  permanent neonatal diabetes mellitus; Ter, termination codon; TNDM,  transient neonatal diabetes mellitus.

Founder mutations have been identified in many populations with the best recognized example being the *ABCC8* p.(Phe1388del) and c.3992‐9G>A mutations present in >90% of cases from the Ashkenazi Jewish population (Nestorowicz et al., 1996; Otonkoski et al., 1999). In the Irish population, a deep intronic *ABCC8* founder mutation at position c.1333‐1013G>A has been described that generates a cryptic splice site and causes pseudoexon activation (Flanagan et al., 2013). Founder mutations have also been reported in Hispanic (Aguilar‐Bryan & Bryan, 1999), Bedouin (Tornovsky et al., 2004), Spanish (Fernandez‐Marmiesse et al., 2006), Finnish (Otonkoski et al., 1999), and Turkish populations (Flanagan et al., 2013).

### 4.2 Common variation in *ABCC8* and *KCNJ11*

Three hundred and sixty‐eight benign/likely benign variants and variants of uncertain significance have been observed in both genes (Tables 2, 3, 4, S2, S3, S5, and S6). Two common variants in linkage disequilibrium, p.(Glu23Lys) in *KCNJ11* and p.(Ser1370Ala) in *ABCC8*, predispose to type 2 diabetes (Florez et al., 2004). Although their effect size is small (odds ratio ~1.2), given that 58% of the population carry at least one lysine allele at residue 23 in *KCNJ11*, this equates to a sizeable population risk (Gloyn, Weedon, et al., 2003; Nielsen et al., 2003).

**Table 2:** Unpublished variants of uncertain clinical significance identified in *KCNJ11* (NM_000525.3)

Protein change	Nucleotide position	Mutation type	Phenotype	Zygosity	Inheritance	GnomAD MAF	Reporting laboratory
p.(Arg4His)	c.11G>A	Missense	HI	Heterozygous	Unaffected mother	0.000008066	Exeter
p.(Cys42Tyr)	c.125G>A	Missense	Diabetes	Heterozygous	Not known	0	Paris
p.(Ala45Ser)	c.133G>T	Missense	Diabetes	Heterozygous	Unaffected parent	0	Exeter
p.(Arg50Trp)	c.148C>T	Missense	Later‐onset diabetes/HI	Heterozygous/ Homozygous/ Heterozygous	Affected parent/ Not known/ Unaffected father	0	Paris/ Paris/ Exeter
p.(Gln52Pro)	c.155A>C	Missense	NDM	Heterozygous	Not known	0	Exeter
p.(Asp58Val)	c.173A>T	Missense	HI	Heterozygous	Unaffected father	0	Paris
p.(Phe60Ser)	c.179T>C	Missense	HI	Heterozygous (in *cis* with VUS)	Unaffected mother	0	Chicago
p.(Leu84Arg)	c.251T>G	Missense	HI	Homozygous	Bi‐parental	0	Exeter
p.(Ala96Val)	c.287C>T	Missense	HI	Heterozygous	Unaffected father	0	Exeter
p.(His97Tyr)	c.289C>T	Missense	Diabetes	Heterozygous	Unaffected parent	0	Exeter
p.(Ile114Thr)	c.341T>C	Missense	Diabetes	Heterozygous	Not known	0	Paris
p.(His115Leu)	c.344A>T	Missense	HI	Heterozygous	Unaffected father	0	Paris
p.(Ser118Leu)	c.353C>T	Missense	Diabetes	Heterozygous/Heterozygous	Not known/ Not known	0.00002389	Paris/ Chicago
p.(Phe121Ser)	c.362T>C	Missense	HI	Heterozygous	Unaffected father	0	Paris
p.(Ile131dup)	c.391_393dup	In‐Frame duplication	HI	Homozygous	Bi‐parental	0	Paris
p.(Ile131Val)	c.391A>G	Missense	HI	Heterozygous	Unaffected father	0	Exeter
p.(Thr139Pro)	c.415A>C	Missense	HI	Heterozygous (in *cis* with VUS)	Unaffected father	0	Paris
p.(Glu140Lys)	c.418G>A	Missense	HI	Homozygous	Bi‐parental	0	Paris
p.(Cys142Tyr)	c.425G>A	Missense	HI	Heterozygous	Unaffected father	0	Exeter
p.(Val155Leu)	c.463G>T	Missense	HI	Heterozygous	Unaffected mother	0	Exeter
p.(Val155Met)	c.463G>A	Missense	Diabetes	Heterozygous/ Heterozygous	Not known/ Not known	0.00001199	Chicago/ Paris
p.(Leu157Val)	c.469C>G	Missense	HI	Heterozygous	Unaffected mother	0	Exeter
p.(Asn160Lys)	c.480C>G	Missense	HI	Heterozygous	Not known	0	Paris
p.(Ile167Val)	c.499A>G	Missense	HI	Heterozygous (in *cis* with VUS)	Unaffected father	0	Paris
p.(Thr171Asn)	c.512C>A	Missense	HI	Heterozygous	Unaffected father	0	Exeter
p.(Thr180Ile)	c.539C>T	Missense	HI	Heterozygous	Unaffected father	0	Paris
p.(Ser208Asn)	c.623G>A	Missense	Diabetes	Heterozygous	Not known	0	Paris
p.(Lys222Gln)	c.664A>C	Missense	HI	Heterozygous	Unaffected mother	0.00001064	Exeter
p.(Ser265Ile)	c.794G>T	Missense	HI	Heterozygous	Unaffected father	0.000003978	Exeter
p.(Tyr268His)	c.802T>C	Missense	HI	Heterozygous	Unaffected father	0	Exeter
p.(Asp274His)	c.820G>C	Missense	HI	Heterozygous	Unaffected father	0	Exeter
p.(Leu287Pro)	c.860T>C	Missense	HI	Heterozygous	Unaffected father	0	Paris
p.(Thr297Asn)	c.890C>A	Missense	NDM	Heterozygous	Unaffected parent	0	Exeter
p.(Ala300Asp)	c.899C>A	Missense	HI	Heterozygous	Not known	0	Paris
p.(Leu310Pro)	c.929T>C	Missense	HI	Heterozygous	Not maternal	0	Exeter
p.(Ile318Val)	c.952A>G	Missense	Diabetes	Heterozygous	Not known (affected sibling also heterozygous)	0.00001061	Paris
p.(Arg325Ser)	c.973C>A	Missense	HI	Heterozygous (in *cis* with VUS)	Unaffected mother	0.00001591	Chicago
p.(Arg325His)	c.974G>A	Missense	HI	Heterozygous	Unaffected father	0.00001591	Exeter
p.(Thr336Ala)	c.1006A>G	Missense	Diabetes	Heterozygous	Not known	0	Exeter
p.(Leu343Val)	c.1027C>G	Missense	NDM	Heterozygous	Unaffected parent	0	Exeter
p.(Arg369Ser)	c.1105C>A	Missense	Diabetes	Heterozygous	Not known	0.00003988	Paris
p.(Arg369His)	c.1106G>A	Missense	Diabetes	Heterozygous	Unaffected parent	0.000003989	Exeter
p.(Arg369Leu)	c.1106G>T	Missense	HI	Heterozygous	Paternal	0.000003989	Chicago
p.(Ala376Ser)	c.1126G>T	Missense	HI	Heterozygous	Maternal	0	Paris
p.(Pro380_Lys381dup)	c.1138_1143dup	In‐Frame duplication	Diabetes	Heterozygous	Not known	0.00007098	Paris

*Note*: The phenotype column highlights a new phenotype; the reporting laboratory column indicates which laboratory has identified the variant in a patient with the new phenotype. See Supporting Information data for details of inclusion criteria for variants in this table.

Abbreviations: HI, hyperinsulinism; NDM, neonatal diabetes.

**Table 3:** Unpublished pathogenic variants identified in *ABCC8* (NM_001287174.1)

Protein change	Nucleotide position	Position	Mutation type	Phenotype	Zygosity	Likely dominant or recessive	GnomAD MAF	Reporting laboratory
p.?	c.(?‐1)_(1011+1_1012–1)del	Exons 1–6	Deletion	HI	Heterozygous^Pat^	Recessive	0	Exeter
p.?	c.(?‐1)_(4749+?)del	Exons 1–39	Deletion	HI	Heterozygous^Pat^	Recessive	0	Exeter
p.(Gly7Cys)	c.19G>T	Exon1	Missense	HI	Compound heterozygous	Recessive	0	Paris
p.(Glu9Ter)	c.25G>T	Exon 1	Nonsense	HI	Homozygous	Recessive	0	Exeter
p.(Asn10ThrfsTer68)	c.29del	Exon 1	Frameshift	HI	Heterozygous	Not known	0	Exeter
p.(Gln19Ter)	c.55C>T	Exon 1	Nonsense	HI	Homozygous/ Homozygous	Recessive/ Recessive	0.000004209	Exeter/ Odense
p.(Gly25AlafsTer53)	c.74del	Exon 1	Frameshift	HI	Heterozygous^Pat^	Recessive	0	Exeter
p.(Cys26Trp)	c.78C>G	Exon 1	Missense	HI	Heterozygous^Pat^	Recessive	0	Paris
p.(Val28SerfsTer61)	c.81_82insA	Exon 1	Frameshift	HI	Heterozygous	Not known	0	Exeter
p.(Ile46Thr)	c.137T>C	Exon 1	Missense	HI	Compound heterozygous	Recessive	0	Paris
p.?	c.(148+1_149–1)_(290+1_291–1)del	Exon 2	Deletion	HI	Homozygous/ Homozygous	Recessive/ Recessive	0	Exeter/ Odense
p.(Trp65Ter)	c.195G>A	Exon 2	Nonsense	HI	Homozygous	Recessive	0	Paris
p.(Arg74Leu)	c.221G>T	Exon 2	Missense	HI	Heterozygous^Pat^	Recessive	0.000003978	Odense
p.(Trp75CysfsTer12)	c.225_229del	Exon 2	Frameshift	HI	Compound heterozygous	Recessive	0	Exeter
p.?	c.(290+1_291–1)_822+1_823–1)del	Exons 3–5	Deletion	HI	Homozygous	Recessive	0	Exeter
p.(Pro133Arg)	c.398C>G	Exon 3	Missense	HI	Homozygous	Recessive	0	Seattle
p.?	c.(412+1_413–1)_(579+1_580–1)del	Exon 4	Deletion	HI	Compound heterozygous	Recessive	0	Paris
p.(Leu175AlafsTer97)	c.522dup	Exon 4	Frameshift	HI	Homozygous	Recessive	0	Exeter
p.?	c.580–2A>G	Intron 4	Aberrant splicing	HI	Homozygous/ Heterozygous^Pat^	Recessive/ Recessive	0	Exeter/ Odense
p.(Pro206Leu)	c.617C>T	Exon 5	Missense	TNDM	Heterozygous^denovo^	Dominant	0	Exeter
p.(Asp212Gly)	c.635A>G	Exon 5	Missense	NDM	Heterozygous^denovo^	Dominant	0	Exeter
p.(Asp212Glu)	c.636C>G	Exon 5	Missense	NDM	Heterozygous	Dominant	0	Chicago
p.(Leu225_Ser226insThrLysTer)	c.674_675insCACGAAGTAGCA	Exon 5	Nonsense	HI	Heterozygous^Pat^	Recessive	0	Odense
p.(Tyr230Cys)	c.689A>G	Exon 5	Missense	HI	Heterozygous^Pat^	Recessive	0.0001034	Odense
p.(Ala235Val)	c.704C>T	Exon 5	Missense	NDM	Heterozygous	Not known	0	Exeter
p.(Pro254Leu)	c.761C>T	Exon 5	Missense	HI	Heterozygous^Pat^	Recessive	0	Odense
p.(Gln282Ter)	c.844C>T	Exon 6	Nonsense	HI	Heterozygous^Pat^	Recessive	0	Exeter
p.(Lys329Ter)	c.985A>T	Exon 6	Nonsense	HI	Compound heterozygous	Recessive	0	Exeter
p.?	c.1012–2A>G	Intron 6	Aberrant splicing	HI	Heterozygous/ Heterozygous^Pat^	Not known/ Recessive	0	Exeter/ Odense
p.(Glu350Gly)	c.1049A>G	Exon 7	Missense	PNDM	Homozygous	Recessive	0	Exeter
p.(Tyr356Ter)	c.1068C>G	Exon 7	Nonsense	HI	Homozygous	Recessive	0	Exeter
p.(Val360Ala)	c.1079T>C	Exon 7	Missense	TNDM	Heterozygous^denovo^	Dominant	0	Exeter
p.(Leu362ArgfsTer26)	c.1085del	Exon 7	Frameshift	HI	Homozygous	Recessive	0	Exeter
p.(Leu366Phe)	c.1096C>T	Exon 7	Missense	HI	Heterozygous	Not known	0	Odense
p.(Thr371Ile)	c.1112C>T	Exon 7	Missense	HI	Assumed compound heterozygous with pathogenic variant	Assumed recessive	0.000007953	Paris
p.(Gln374Ter)	c.1120C>T	Exon 7	Nonsense	HI	Heterozygous^Pat^	Recessive	0	Exeter
p.(Ala380ProfsTer8)	c.1138del	Exon 7	Frameshift	HI	Homozygous	Recessive	0.000003976	Exeter
p.(Gly384Ter)	c.1150_1159del	Exon 7	Nonsense	HI	Homozygous	Recessive	0	Exeter
p.?	c.1332+1G>A	Intron 8	Aberrant splicing	HI	Heterozygous^Pat^	Recessive	0	Paris
p.?	c.1332+3A>G	Intron 8	Aberrant splicing	HI	Homozygous	Recessive	0	Exeter
p.?	c.(1332+1_1333–1)_(1671+1_1672–1)dup	Exon 9–11	Duplication	HI	Heterozygous^Pat^	Recessive	0	Exeter
p.(Val447LeufsTer4)	c.1337_1338dup	Exon 9	Frameshift	HI	Compound heterozygous	Recessive	0	Paris/ Odense
p.?	c.1467+6T>G	Intron 9	Aberrant splicing	HI	Compound heterozygous	Recessive	0	Paris
p.?	c.1468–48G>A	Intron 9	Aberrant splicing	HI	Homozygous	Recessive	0	Exeter
p.(Asn500GlnfsTer122)	c.1497dup	Exon 10	Frameshift	HI	Heterozygous^Pat^	Recessive	0	Exeter
p.(Gly505Arg)	c.1513G>C	Exon 10	Missense	HI	Heterozygous^denovo^ /Heterozygous^denovo^	Dominant/ Dominant	0	Exeter/ Paris
p.(Phe536Ser)	c.1607T>C	Exon 10	Missense	NDM	Heterozygous^denovo^	Dominant	0	Exeter
p.?	c.1631–2A>T	Intron 10	Aberrant splicing	HI	Compound heterozygous	Recessive	0	Paris
p.?	c.1672–20A>T	Intron 11	Aberrant splicing	HI	Homozygous	Recessive	0	Exeter
p.(His562GlnfsTer58)	c.1683_1687del	Exon 12	Frameshift	HI	Heterozygous^Pat^	Recessive	0	Exeter
p.(Phe577Leu)	c.1731T>G	Exon 12	Missense	TNDM	Heterozygous^denovo^	Dominant	0	Exeter
p.(Val587Asp)	c.1760T>A	Exon 12	Missense	NDM	Heterozygous^denovo^	Dominant	0	Exeter
p.(Ser594Pro)	c.1780T>C	Exon 12	Missense	HI	Heterozygous^Pat^	Recessive	0	Odense
p.(Lys609ArgfsTer2)	c.1826_1828delinsGG	Exon 13	Frameshift	HI	Compound heterozygous	Recessive	0	Paris
p.(Glu612Asp)	c.1836G>T	Exon 13	Missense	HI	Heterozygous^Pat^	Recessive	0.000007974	Odense
p.?	c.1924–2A>T	Intron 13	Aberrant splicing	HI	Heterozygous^Pat^	Recessive	0	Odense
p.(Glu654Ter)	c.1960G>T	Exon 14	Nonsense	HI	Compound heterozygous	Recessive	0	Exeter
p.?	c.2041–2A>G	Intron 14	Aberrant splicing	HI	Heterozygous	Not known	0	Exeter
p.?	c.2041–1G>A	Intron 14	Aberrant splicing	HI	Heterozygous^Pat^	Recessive	0	Odense
p.(Arg705Ter)	c.2113C>T	Exon 15	Nonsense	HI	Homozygous	Recessive	0.000003989	Exeter
p.(Gly713Arg)	c.2137G>C	Exon 16	Missense	HI	Heterozygous^Pat^	Recessive	0	Exeter
p.(Glu729Ter)	c.2185G>T	Exon 16	Nonsense	HI	Heterozygous	Paternal	0	Exeter
p.?	c.2222+1G>A	Intron 16	Aberrant splicing	HI	Homozygous	Recessive	0	Exeter
p.(Glu757Ter)	c.2269G>T	Exon 18	Nonsense	HI	Compound heterozygous	Recessive	0	Exeter
p.(Arg767SerfsTer21)	c.2298_2310delinsAA	Exon 19	Frameshift	HI	Heterozygous^Pat^	Recessive	0	Chicago
p.(Gly768ProfsTer23)	c.2301_2302del	Exon 19	Frameshift	HI	Homozygous	Recessive	0	Exeter
p.(Phe794SerfsTer71)	c.2379del	Exon 19	Frameshift	HI	Heterozygous^Pat^	Recessive	0	Paris
p.(Tyr799Ter)	c.2397del	Exon 20	Nonsense	HI	Assumed compound heterozygous with pathogenic variant	Assumed recessive	0	Paris
p.(Cys806Tyr)	c.2417G>A	Exon 20	Missense	HI	Homozygous	Recessive	0	Exeter
p.(Asp811Val)	c.2432A>T	Exon 20	Missense	TNDM	Heterozygous	Dominant	0	Chicago
p.(His817Arg)	c.2450A>G	Exon 20	Missense	Later onset diabetes	Heterozygous	Not known	0.00001768	Paris
p.?	c.2479–1G>A	Intron 20	Aberrant splicing	HI	Heterozygous^Pat^	Recessive	0	Exeter
p.(Gly827AlafsTer38)	c.2480del	Exon 21	Frameshift	HI	Compound heterozygous	Recessive	0	Paris
p.(Arg842GlufsTer23)	c.2524del	Exon 21	Frameshift	HI	Homozygous	Recessive	0	Exeter
p.(Arg842Pro)	c.2525G>C	Exon 21	Missense	HI	Heterozygous^Pat^	Recessive	0	Odense
p.?	c.2559+3_2559+15delinsCCTGGGGTCCTTGT	Intron 21	Aberrant splicing	HI	Heterozygous^Pat^	Recessive	0	Paris
p.?	c.2560–1G>A	Intron 21	Aberrant splicing	HI	Heterozygous^Pat^	Recessive	0	Exeter
p.?	c.(2559+1_2560–1)_(3332+1_3333–1)del	Exons 22–26	Deletion	HI	Compound heterozygous	Recessive	0	Exeter
p.(Gln892Ter)	c.2674C>T	Exon 22	Nonsense	HI	Compound heterozygous	Recessive	0	Exeter
p.(Gln892ProfsTer28)	c.2675_2679del	Exon 22	Frameshift	HI	Homozygous	Recessive	0	Exeter
p.(Gly912Arg)	c.2734G>C	Exon 23	Missense	HI	Compound heterozygous	Recessive	0	Paris
p.(Leu939TrpfsTer104)	c.2815del	Exon 23	Frameshift	HI	Compound heterozygous	Recessive	0	Exeter
p.?	c.2823+1G>A	Intron 23	Aberrant splicing	HI	Homozygous	Recessive	0	Exeter
p.(Glu973ArgfsTer70)	c.2917del	Exon 24	Frameshift	HI	Homozygous	Recessive	0	Exeter
p.(Glu974Gly)	c.2921A>G	Exon 24	Missense	HI	Heterozygous	Dominant	0	Paris
p.?	c.2924–1G>A	Intron 24	Aberrant splicing	HI	Homozygous	Recessive	0.000004162	Exeter
p.?	c.3165+2T>A	Intron 25	Aberrant splicing	HI	Homozygous	Recessive	0	Exeter
p.?	c.3166–1G>A	Intron 25	Aberrant splicing	HI	Homozygous	Recessive	0.000003977	Exeter
p.(Gln1061Ter)	c.3181C>T	Exon 26	Nonsense	HI	Homozygous	Recessive	0	Exeter
p.(Cys1079Ter)	c.3237C>A	Exon 26	Nonsense	HI	Heterozygous	Recessive	0	Exeter
p.(His1098Arg)	c.3293A>G	Exon 26	Missense	HI	Homozygous	Recessive	0	Exeter
p.(Met1110HisfsTer5)	c.3327dup	Exon 26	Frameshift	HI	Heterozygous^Pat^	Recessive	0	Odense
p.(Gln1134Ter)	c.3400C>T	Exon 27	Nonsense	HI	Homozygous	Recessive	0	Exeter
p.(Gln1134Arg)	c.3401A>G	Exon 27	Missense	HI	Compound heterozygous	Recessive	0.00001193	Odense
p.?	c.(3402+1_3403–1)_(3653+1_3654–1)del	Exons 28–29	Deletion	HI	Heterozygous	Not known	0	Exeter
p.(Thr1139HisfsTer7)	c.3410_3414dup	Exon 28	Frameshift	HI	Homozygous	Recessive	0	Exeter
p.(Glu1141Ter)	c.3421G>T	Exon 28	Nonsense	HI	Heterozygous^Pat^	Recessive	0	Exeter
p.(Glu1141Gly)	c.3422A>G	Exon 28	Missense	TNDM	Heterozygous^denovo^	Dominant	0	Paris
p.(Cys1150Ter)	c.3450T>A	Exon 28	Nonsense	HI	Heterozygous^Pat^	Recessive	0.000003990	Exeter
p.(Ala1153Val)	c.3458C>T	Exon 28	Missense	HI	Heterozygous^denovo^	Dominant	0	Exeter
p.(Ala1153Gly)	c.3458C>G	Exon 28	Missense	NDM	Heterozygous	Dominant	0	Exeter
p.(Tyr1181Ter)	c.3543C>A	Exon 28	Nonsense	HI	Homozygous	Recessive	0	Paris
p.(Phe1182Leu)	c.3546C>A	Exon 28	Missense	PNDM/ TNDM	Homozygous/ Heterozygous	Recessive/ Dominant	0	Exeter/ Exeter
p.(Asp1194Val)	c.3581A>T	Exon 29	Missense	HI	Homozygous	Recessive	0.00005303	Odense
p.(Pro1199Ser)	c.3595C>T	Exon 29	Missense	TNDM	Heterozygous^denovo^	Dominant	0	Exeter
p.(Pro1199Gln)	c.3596C>A	Exon 29	Missense	TNDM	Heterozygous^denovo^	Dominant	0	Exeter
p.(Leu1201ThrfsTer18)	c.3600_3604del	Exon 29	Frameshift	HI	Heterozygous^Pat^	Recessive	0	Odense
p.?	c.3653+2T>A	Intron 29	Aberrant splicing	HI	Homozygous	Recessive	0	Exeter
p.?	c.3757–17_3823del	Intron 30	Aberrant splicing	HI	Homozygous	Recessive	0	Exeter
p.(Glu1253Ter)	c.3757G>T	Exon 31	Nonsense	HI	Homozygous	Recessive	0	Exeter
p.(Ser1267Phe)	c.3800C>T	Exon 31	Missense	NDM	Heterozygous	Dominant	0	Chicago
p.(Leu1276Pro)	c.3827T>C	Exon 31	Missense	Later‐onset diabetes	Heterozygous	Dominant	0	Paris
p.(Leu1283AlafsTer8)	c.3844_3845dup	Exon 31	Frameshift	HI	Heterozygous^Pat^	Not known	0	Paris
p.(Tyr1287Ter)	c.3861C>A	Exon 31	Nonsense	HI	Homozygous/ Heterozygous^Pat^	Recessive/ Recessive	0	Exeter/ Odense
p.(Met1290Ile)	c.3870G>T	Exon 31	Missense	HI	Assumed compound heterozygous with pathogenic variant	Assumed recessive	0	Paris
p.?	c.3871–2A>G	Intron 31	Aberrant splicing	HI	Homozygous	Recessive	0	Exeter
p.(Leu1295Phe)	c.3883C>T	Exon 32	Missense	PNDM	Heterozygous^denovo^	Dominant	0	Exeter
p.(Glu1324Ter)	c.3970G>T	Exon 32	Nonsense	HI	Compound heterozygous	Recessive	0	Exeter
p.(Tyr1326Ter)	c.3978del	Exon 32	Nonsense	HI	Compound heterozygous	Recessive	0	Exeter
p.(Glu1327Ter)	c.3979G>T	Exon 32	Nonsense	HI	Homozygous	Recessive	0	Exeter
p.?	c.3991+1G>A	Intron 32	Aberrant splicing	HI	Heterozygous^Pat^	Recessive	0	Exeter
p.(Ser1333Ter)	c.3998C>A	Exon 33	Nonsense	HI	Heterozygous^denovo^	Recessive	0.000003977	Paris
p.(Ile1347Phe)	c.4039A>T	Exon 33	Missense	HI	Compound heterozygous	Recessive	0	Paris
p.(Asn1349SerfsTer5)	c.4045_4061delinsT	Exon 33	Frameshift	HI	Heterozygous^Pat^	Recessive	0	Exeter
p.(Arg1380Pro)	c.4139G>C	Exon 34	Missense	NDM	Heterozygous	Dominant	0	Exeter
p.(Thr1381Asn)	c.4142C>A	Exon 34	Missense	TNDM	Heterozygous^denovo^	Dominant	0	Exeter
p.(Gly1401Trp)	c.4201G>T	Exon 34	Missense	HI	Heterozygous^Pat^	Recessive	0	Odense
p.(His1402ThrfsTer59)	c.4203del	Exon 35	Frameshift	HI	Homozygous	Recessive	0	Exeter
p.(Ile1405del)	c.4212_4214del	Exon 35	In frame deletion	HI	Homozygous	Recessive	0	Exeter
p.(Ser1423Pro)	c.4267T>C	Exon 35	Missense	HI	Heterozygous^Pat^	Recessive	0	Exeter
p.(Ser1423Cys)	c.4268C>G	Exon 35	Missense	NDM	Heterozygous	Dominant	0	Chicago
p.(Asp1428ArgfsTer6)	c.4282_4298del	Exon 35	Frameshift	HI	Heterozygous^Pat^	Recessive	0	Chicago
p.(Pro1429LeufsTer8)	c.4286_4293del	Exon 35	Frameshift	HI	Heterozygous^Pat^	Recessive	0	Exeter
p.?	c.4311–1G>T	Intron 35	Aberrant splicing	HI	Compound heterozygous	Recessive	0	Paris
p.(Trp1452Cys)	c.4356G>C	Exon 36	Missense	HI	Compound heterozygous	Recessive	0	Paris
p.?	c.(4414+1_4415–1)_(*4749+34)del	Exons 37–39	Deletion	HI	Compound heterozygous	Recessive	0	Exeter
p.(Gly1485Val)	c.4454G>T	Exon 37	Missense	HI	Heterozygous^denovo^	Dominant	0	Chicago
p.(Gln1486Ter)	c.4456C>T	Exon 37	Nonsense	HI	Homozygous	Recessive	0.000003977	Exeter
p.(Gln1488Arg)	c.4463A>G	Exon 37	Missense	HI	Heterozygous^denovo^	Dominant	0	Exeter
p.(Cys1491AlafsTer7)	c.4471del	Exon 37	Frameshift	HI	Homozygous	Recessive	0	Paris
p.(Ser1501Arg)	c.4503C>A	Exon 37	Missense	Later‐onset diabetes	Heterozygous	Dominant	0	Exeter
p.(Met1505Thr)	c.4514T>C	Exon 37	Missense	Later‐onset diabetes	Heterozygous	Dominant	0.00001194	Paris
p.(Asp1506Asn)	c.4516G>A	Exon 37	Missense	HI progressed to diabetes	Heterozygous	Dominant	0	Paris
p.(Glu1507_Asp1513dup)	c.4519_4539dup	Exon 37	In frame duplication	HI	Heterozygous	Dominant	0	Chicago
p.?	c.4548+1G>C	Intron 37	Aberrant splicing	HI	Heterozygous^Pat^	Recessive	0	Odense
p.(Val1523Met)	c.4567G>A	Exon 38	Missense	Later‐onset diabetes	Heterozygous	Dominant	0	Paris
p.?	c.4611+4A>G	Intron 38	Aberrant splicing	HI	Homozygous	Recessive	0	Paris
p.(Arg1539Ter)	c.4615C>T	Exon 39	Nonsense	HI	Heterozygous^Pat^	Recessive	0	Paris
p.(Val1540Met)	c.4618G>A	Exon 39	Missense	TNDM	Heterozygous	Dominant	0	Exeter
p.(Glu1559Ter)	c.4675G>T	Exon 39	Nonsense	HI	Compound heterozygous	Recessive	0	Exeter
p.(Ser1572Arg)	c.4716C>A	Exon 39	Missense	HI	Heterozygous^Pat^	Recessive	0	Paris
p.(Arg1579GlnfsTer31)	c.4734_4737del	Exon 39	Frameshift	HI	Compound heterozygous	Recessive	0	Paris

*Note*: The phenotype column highlights a new phenotype; the reporting laboratory column indicates which laboratory has identified the variant in a patient with the new phenotype. See Supporting Information data for details of inclusion criteria for variants in this table.

Abbreviations: HI, hyperinsulinism; NDM, neonatal diabetes; Ter, termination codon; TNDM, transient neonatal diabetes mellitus.

**Table 4:** Unpublished variants of uncertain clinical significance identified in *ABCC8* (NM_001287174.1)

Protein change	Nucleotide position	Position	Mutation type	Phenotype	Zygosity	Inheritance	GnomAD MAF	Reporting laboratory
p.(Ala14Ser)	c.40G>T	Exon 1	Missense	Diabetes	Heterozygous	Not known	0	Paris
p.(Tyr15Phe)	c.44A>T	Exon 1	Missense	HI	Heterozygous	Not known	0	Paris
p.(Phe41Leu)	c.121T>C	Exon 1	Missense	Diabetes	Heterozygous	Not known	0	Paris
p.(His59Asn)	c.175C>A	Exon 2	Missense	HI	Homozygous	Bi‐parental	0	Paris
p.(Gly97=)	c.291G>T	Exon 3	Missense	Diabetes	Heterozygous	Not known	0	Paris
p.(Val121Met)	c.361G>A	Exon 3	Missense	Diabetes	Heterozygous	Affected parent	0	Paris
p.(Val121Ala)	c.362T>C	Exon 3	Missense	NDM	Heterozygous	Not known	0	Chicago
p.(Ile127Thr)	c.380T>C	Exon 3	Missense	Diabetes	Heterozygous	Not known	0	Paris
p.(Ile137Ser)	c.410T>G	Exon 3	Missense	Diabetes	Heterozygous	Not known	0	Paris
p.?	c.580–16_580–14del	Intron 4	Intronic deletion	Diabetes	Heterozygous	Not known	0.00001776	Paris
p.(Arg194Lys)	c.581G>A	Exon 5	Missense	Diabetes	Heterozygous	Not known	0	Paris
p.(Pro201Leu)	c.602C>T	Exon 5	Missense	HI	Heterozygous	Maternal	0	Paris
p.(Ala240Thr)	c.718G>A	Exon 5	Missense	HI	Heterozygous	Maternal	0	Paris
p.(Met257Leu)	c.769A>C	Exon 5	Missense	Diabetes	Heterozygous	Not known	0.000003976	Paris
p.(Met257Thr)	c.770T>C	Exon 5	Missense	Diabetes	Heterozygous	Not known	0	Paris
p.(Phe270Cys)	c.809T>G	Exon 5	Missense	Diabetes	Heterozygous	Not known	0	Paris
p.(His293Pro)	c.878A>C	Exon 6	Missense	HI	Heterozygous	Paternal	0	Chicago
p.(Gly316Glu)	c.947G>A	Exon 6	Missense	HI	Heterozygous	Paternal	0	Chicago
p.(Gly342Arg)	c.1024G>A	Exon 7	Missense	Diabetes	Heterozygous	Not known	0.00001591	Paris
p.(Val357Ile)	c.1069G>A	Exon 7	Missense	HI/ Later‐onset diabetes	Heterozygous/ Heterozygous	Not known/ Not known	0.00003181	Odense/ Paris
p.(Ile395Phe)	c.1183A>T	Exon 8	Missense	NDM	Heterozygous	Not known	0.000007953	Chicago
p.(Thr413Ser)	c.1238C>G	Exon 8	Missense	Diabetes	Heterozygous	Maternal	0	Exeter
p.(Asp424Gly)	c.1271A>G	Exon 9	Missense	PNDM	Homozygous	Recessive	0	Paris
p.(Ile446Thr)	c.1337T>C	Exon 9	Missense	Diabetes	Heterozygous	Not known	0.00001194	Paris
p.(Gly457Arg)	c.1369G>A	Exon 9	Missense	Diabetes	Heterozygous	Affected parent	0.00004598	Paris
p.(Arg504Cys)	c.1510C>T	Exon 10	Missense	Diabetes	Heterozygous	Unaffected parent	0.000007969	Paris
p.(Gly505Cys)	c.1513G>T	Exon 10	Missense	HI	Heterozygous	Paternal	0	Paris
p.(Ala513Thr)	c.1537G>A	Exon 10	Missense	Diabetes	Heterozygous	Unaffected mother	0.00004601	Paris
p.(Arg521Trp)	c.1561C>T	Exon 10	Missense	Diabetes	Heterozygous/ Heterozygous	Not known/ Dominant	0.00002787	Chicago/ Paris
p.(Arg521Gln)	c.1562G>A	Exon 10	Missense	Diabetes	Heterozygous	Not known	0.00009556	Paris
p.(Val522Met)	c.1564G>A	Exon 10	Missense	Diabetes	Heterozygous	Not known	0.000007078	Paris
p.(Ala537Thr)	c.1609G>A	Exon 10	Missense	HI	Heterozygous	Paternal	0	Paris
p.(Val575Met)	c.1723G>A	Exon 12	Missense	Diabetes	Heterozygous	Not known	0.00001591	Paris
p.(Phe613Leu)	c.1837T>C	Exon 13	Missense	Diabetes	Heterozygous	Not known	0	Paris
p.?	c.1924–44A>G	Intron 13	Intronic substitution	HI	Heterozygous	Paternal	0	Odense
p.(Cys656Phe)	c.1967G>T	Exon 14	Missense	Diabetes	Heterozygous	Not known	0.000003984	Paris
p.(Arg702Cys)	c.2104C>T	Exon 15	Missense	Diabetes	Heterozygous	Not known	0.00008768	Paris
p.?	c.2116+61A>G	Intron 15	Intronic substitution	Diabetes	Heterozygous	Not known	0.00003187	Paris
p.(Gln731Glu)	c.2191C>G	Exon 16	Missense	HI	Heterozygous	Not known	0.00001444	Paris
p.(Val770Met)	c.2308G>A	Exon 19	Missense	HI	Assumed compound heterozygous with pathogenic variant	Assumed recessive	0.00002031	Paris
p.(Ser831Thr)	c.2491T>A	Exon 21	Missense	Diabetes	Heterozygous	Not known	0	Paris
p.(Arg835His)	c.2504G>A	Exon 21	Missense	Diabetes	Heterozygous	Not known	0.00002442	Paris
p.(Ile838Val)	c.2512A>G	Exon 21	Missense	Diabetes	Heterozygous	Not known	0	Paris
p.(Val840Ala)	c.2519T>C	Exon 21	Missense	Diabetes	Heterozygous	Not known	0	Paris
p.(Asn849Thr)	c.2546A>C	Exon 21	Missense	Diabetes	Heterozygous	Not known	0	Paris
p.(His863Arg)	c.2588A>G	Exon 22	Missense	Diabetes	Heterozygous	Affected parent	0.000007953	Paris/ Exeter
p.(Arg934Gln)	c.2801G>A	Intron 23	Missense	HI	Heterozygous	Paternal	0.00001193	Paris
p.(Ala1002Thr)	c.3004G>A	Exon 25	Missense	HI	Homozygous, *in cis* with VUS	Recessive	0.00003575	Paris
p.(Ser1019Leu)	c.3056C>T	Exon 25	Missense	Diabetes/HI	Heterozygous/ Compound heterozygous/ Heterozygous	Unknown/ Recessive/ Affected father	0.000008152	Paris
p.(Thr1038Asn)	c.3113C>A	Exon 25	Missense	Diabetes	Heterozygous	Not known	0	Paris
p.(Val1166Met)	c.3496G>A	Exon 28	Missense	Diabetes	Compound heterozygous/ Heterozygous	Recessive/ Dominant	0.00008843	Chicago/ Paris
p.?	c.3561–19A>C	Intron 28	Intronic substitution	HI	Heterozygous	Not known	0	Chicago
p.(Asp1194Val)	c.3581A>T	Exon 29	Missense	Diabetes	Heterozygous	Not known	0.00005303	Paris
p.(Glu1209Lys)	c.3625G>A	Exon 29	Missense	HI	Heterozygous	Affected grandparent	0	Paris
p.(Phe1217Leu)	c.3651C>G	Exon 29	Missense	TNDM	Heterozygous	Unaffected parent	0	Paris
p.?	c.3653+4C>G	Intron 29	Intronic substitution	Later‐onset diabetes/HI	Heterozygous/ Heterozygous	Affected parent/ Not known	0.0001449	Paris/ Exeter
p.(Leu1241Arg)	c.3722T>G	Exon 30	Missense	HI	Heterozygous	Paternal	0	Paris
p.(Glu1249Ala)	c.3746A>C	Exon 30	Missense	HI	Heterozygous	Affected mother	0	Paris
p.(Glu1253Gly)	c.3758A>G	Exon 31	Missense	HI	Heterozygous	Maternal	0	Chicago
p.(Val1260Met)	c.3778G>A	Exon 31	Missense	Diabetes	Heterozygous	Affected parent	0.00005321	Paris
p.?	c.3992–10C>T	Intron 32	Intronic substitution	HI	Heterozygous	Maternal	0.0004177	Odense
p.?	c.4123–17T>C	Intron 33	Intronic substitution	Diabetes	Heterozygous	Not known	0	Chicago
p.(Ser1423Phe)	c.4268C>T	Exon 35	Missense	HI	Compound heterozygous with VUS	Recessive	0	Paris
p.(Gln1427Lys)	c.4279C>A	Exon 35	Missense	Diabetes	Heterozygous	Not known	0	Paris
p.(Asn1439=)	c.4317C>T	Exon 36	Synonymous	HI	Compound heterozygous	Recessive	0.00001458	Paris
p.(Pro1442Leu)	c.4325C>T	Exon 36	Missense	HI	Homozygous, *in cis* with VUS	Recessive	0	Paris
p.(Gly1478=)	c.4434C>T	Exon 37	Synonymous	HI	Heterozygous	Not known	0.0001697	Chicago
p.(Ala1495=)	c.4485C>T	Exon 37	Synonymous	HI	Heterozygous	Not known	0.0002228	Chicago
p.(Val1497Met)	c.4489G>A	Exon 37	Missense	HI	Heterozygous/ Heterozygous	Paternal/ Paternal	0.000007957	Paris/ Odense
p.(Ile1504Asn)	c.4511T>A	Exon 37	Missense	Diabetes	Heterozygous	Not known	0	Paris
p.(Arg1531His)	c.4592G>A	Exon 38	Missense	Diabetes	Heterozygous	Not known	0.00001061	Chicago
p.(Val1534Leu)	c.4600G>C	Exon 38	Missense	Diabetes	Heterozygous	Unaffected parent	0	Paris
p.(Ser1576Pro)	c.4726T>C	Exon 39	Missense	HI	Compound heterozygous	Recessive	0	Paris
p.(Arg1579His)	c.4736G>A	Exon 39	Missense	Diabetes	Heterozygous	Not known	0.00004952	Paris

*Note*: The Phenotype column highlights a new phenotype; the reporting laboratory column indicates which laboratory identified the variant in patient with the new phenotype. See Supporting Information data for details of inclusion criteria for variants in this table.

Abbreviations: HI, hyperinsulinism; NDM, neonatal diabetes; TNDM, transient neonatal diabetes mellitus.

### 4.3 Variant interpretation

Given the highly polymorphic nature of *ABCC8* and *KCNJ11*, the occurrence of both activating and inactivating mutations, the multiple modes of inheritance of disease, and the variable penetrance associated with dominantly acting mutations, interpreting variants identified in these genes can be extremely challenging. Although the identification of a null *ABCC8* or *KCNJ11* variant(s) in an individual with CHI provides strong evidence for pathogenicity, finding a missense variant is insufficient to assign disease causality and, as such, additional support is required to achieve a “pathogenic” classification according to the guidelines set out by the American College of Medical Genetics (Richards et al., 2015).

Large variant databases such as GnomAD and LOVD are powerful tools that aid in variant interpretation and allow for reclassification of variants (Fokkema et al., 2011; Lek et al., 2016). As such, some variants previously reported as pathogenic in the literature have now been found to be too common to be causative of disease and have now be reassigned as a variant of uncertain significance or a benign variant (Tables S2, S3, S5, and S6).

## 5 FUTURE PROSPECTS

Although sulphonylureas provide a safe and effective treatment for the majority of individuals with K_ATP_ channel NDM, for patients with CHI, pharmacological management of the condition is not always successful. Current efforts are, therefore, focusing on the development of new pharmacological treatments for this condition (Banerjee, De Leon, & Dunne, 2017; De Leon et al., 2008; Ng, Tang, Seeholzer, Zou, & De Leon, 2018; Patel et al., 2018; Powell et al., 2011; Senniappan et al., 2014).

Progress is also being made in terms of genetic screening, with a recent report describing the use of noninvasive prenatal testing of a paternally inherited *KCNJ11* activating mutation in cell‐free fetal DNA (De Franco et al., 2017). Implementation of noninvasive prenatal testing for maternally inherited mutations will be extremely important, as a previous study suggested that sulphonylurea can cross the placenta and influence fetal growth with implications for treatment of monogenic diabetes pregnancies (Myngheer et al., 2014; Shepherd, Brook, Chakera, & Hattersley, 2017).

## 6 SUMMARY

The discovery of both inactivating and activating K_ATP_ channel mutations has firmly established the critical role of the channel in insulin secretion. The highly polymorphic nature of the two genes along with the occurrence of both gain‐of‐function and loss‐of‐function mutations as well as multiple different modes of inheritance can make variant interpretation extremely challenging. Rapid testing is absolutely crucial for all patients with CHI or NDM because finding a mutation has a huge impact on the clinical management of these conditions.

## Supporting information

Supporting information

## Data Availability

All the novel variants reported in this manuscript have been uploaded to LOVD (https://www.lovd.nl/).

## References

[humu23995-bib-0001] Aguilar‐Bryan, L. , & Bryan, J. (1999). Molecular biology of adenosine triphosphate‐sensitive potassium channels. Endocrine Reviews, 20(2), 101–135. 10204114 10.1210/edrv.20.2.0361

[humu23995-bib-0002] Ashcroft, F. M. (2005). ATP‐sensitive potassium channelopathies: Focus on insulin secretion. Journal of Clinical Investigation, 115(8), 2047–2058. 16075046 10.1172/JCI25495 PMC1180549

[humu23995-bib-0003] Ashcroft, F. M. , Harrison, D. E. , & Ashcroft, S. J. (1984). Glucose induces closure of single potassium channels in isolated rat pancreatic beta‐cells. Nature, 312(5993), 446–448. 6095103 10.1038/312446a0

[humu23995-bib-0004] Babenko, A. P. , Polak, M. , Cave, H. , Busiah, K. , Czernichow, P. , Scharfmann, R. , … Froguel, P. (2006). Activating mutations in the ABCC8 gene in neonatal diabetes mellitus. New England Journal of Medicine, 355(5), 456–466. 16885549 10.1056/NEJMoa055068

[humu23995-bib-0005] Babiker, T. , Vedovato, N. , Patel, K. , Thomas, N. , Finn, R. , Männikkö, R. , … Hattersley, A. T. (2016). Successful transfer to sulfonylureas in KCNJ11 neonatal diabetes is determined by the mutation and duration of diabetes. Diabetologia, 59(6), 1162–1166. 27033559 10.1007/s00125-016-3921-8 PMC4869695

[humu23995-bib-0006] Banerjee, I. , De Leon, D. , & Dunne, M. J. (2017). Extreme caution on the use of sirolimus for the congenital hyperinsulinism in infancy patient. Orphanet Journal of Rare Diseases, 12(1), 70. 28410602 10.1186/s13023-017-0621-5 PMC5391606

[humu23995-bib-0007] Beltrand, J. , Elie, C. , Busiah, K. , Fournier, E. , Boddaert, N. , Bahi‐Buisson, N. , … Polak, M. (2015). Sulfonylurea Therapy benefits neurological and psychomotor functions in patients with neonatal diabetes owing to potassium channel mutations. Diabetes Care, 38(11), 2033–2041. 26438614 10.2337/dc15-0837

[humu23995-bib-0008] Bendix, J. , Laursen, M. G. , Mortensen, M. B. , Melikian, M. , Globa, E. , Detlefsen, S. , … Christesen, H. T. (2018). Intraoperative ultrasound: A tool to support tissue‐sparing curative pancreatic resection in focal congenital hyperinsulinism. Frontiers in Endocrinology (Lausanne), 9, 478. 10.3389/fendo.2018.00478 PMC6113400 30186238

[humu23995-bib-0009] Boodhansingh, K. E. , Kandasamy, B. , Mitteer, L. , Givler, S. , De Leon, D. D. , Shyng, S. L. , … Stanley, C. A. (2019). Novel dominant KATP channel mutations in infants with congenital hyperinsulinism: Validation by in vitro expression studies and in vivo carrier phenotyping. American Journal of Medical Genetics. Part A, 179, 2214–2227. 31464105 10.1002/ajmg.a.61335 PMC6852436

[humu23995-bib-0010] Bowman, P. , Broadbridge, E. , Knight, B. A. , Pettit, L. , Flanagan, S. E. , Reville, M. , … Hattersley, A. T. (2016). Psychiatric morbidity in children with KCNJ11 neonatal diabetes. Diabetic Medicine, 33(10), 1387–1391. 27086753 10.1111/dme.13135 PMC5031218

[humu23995-bib-0011] Bowman, P , Day, J , Torrens, L , Shepherd, MH , Knight, BA , Ford, TJ , … Zeman, A. (2018). Cognitive, neurological, and behavioral features in adults with KCNJ11 neonatal diabetes. Diabetes Care, 42(2), 215–224. 30377186 10.2337/dc18-1060 PMC6354912

[humu23995-bib-0012] Bowman, P. , Flanagan, S. E. , Edghill, E. L. , Damhuis, A. , Shepherd, M. H. , Paisey, R. , … Ellard, S. (2012). Heterozygous ABCC8 mutations are a cause of MODY. Diabetologia, 55(1), 123–127. 21989597 10.1007/s00125-011-2319-x

[humu23995-bib-0013] Bowman, P. , Hattersley, A. T. , Knight, B. A. , Broadbridge, E. , Pettit, L. , Reville, M. , … Tonks, J. (2017). Neuropsychological impairments in children with KCNJ11 neonatal diabetes. Diabetic Medicine, 34(8), 1171–1173. 28477417 10.1111/dme.13375

[humu23995-bib-0014] Bowman, P. , Sulen, Å. , Barbetti, F. , Beltrand, J. , Svalastoga, P. , Codner, E. , … øddegård, R. (2018). Effectiveness and safety of long‐term treatment with sulfonylureas in patients with neonatal diabetes due to KCNJ11 mutations: An international cohort study. The Lancet. Diabetes & Endocrinology, 6(8), 637–646. 29880308 10.1016/S2213-8587(18)30106-2 PMC6058077

[humu23995-bib-0015] Busiah, K. , Drunat, S. , Vaivre‐Douret, L. , Bonnefond, A. , Simon, A. , Flechtner, I. , … Cavé, H. (2013). Neuropsychological dysfunction and developmental defects associated with genetic changes in infants with neonatal diabetes mellitus: A prospective cohort study [corrected]. The Lancet. Diabetes & Endocrinology, 1(3), 199–207. 24622368 10.1016/S2213-8587(13)70059-7

[humu23995-bib-0016] Carmody, D. , Pastore, A. N. , Landmeier, K. A. , Letourneau, L. R. , Martin, R. , Hwang, J. L. , … Greeley, S. A. W. (2016). Patients with KCNJ11‐related diabetes frequently have neuropsychological impairments compared with sibling controls. Diabetic Medicine, 33(10), 1380–1386. 27223594 10.1111/dme.13159 PMC5654490

[humu23995-bib-0017] Codner, E. , Flanagan, S. , Ellard, S. , Garcia, H. , & Hattersley, A. T. (2005). High‐dose glibenclamide can replace insulin therapy despite transitory diarrhea in early‐onset diabetes caused by a novel R201L Kir6.2 mutation. Diabetes Care, 28(3), 758–759. 15735229 10.2337/diacare.28.3.758

[humu23995-bib-0018] Cook, D. L. , & Hales, C. N. (1984). Intracellular ATP directly blocks K+ channels in pancreatic B‐cells. Nature, 311(5983), 271–273. 6090930 10.1038/311271a0

[humu23995-bib-0019] Craigie, R. J. , Salomon‐Estebanez, M. , Yau, D. , Han, B. , Mal, W. , Newbould, M. , … Dunne, M. J. (2018). Clinical diversity in focal congenital hyperinsulinism in infancy correlates with histological heterogeneity of islet cell lesions. Frontiers in Endocrinology (Lausanne), 9, 619. 10.3389/fendo.2018.00619 PMC6199412 30386300

[humu23995-bib-0020] Damaj, L. , le Lorch, M. , Verkarre, V. , Werl, C. , Hubert, L. , Nihoul‐Fékété, C. , … Jaubert, F. (2008). Chromosome 11p15 paternal isodisomy in focal forms of neonatal hyperinsulinism. Journal of Clinical Endocrinology and Metabolism, 93(12), 4941–4947. 18796520 10.1210/jc.2008-0673

[humu23995-bib-0021] De Franco, E. , Caswell, R. , Houghton, J. A. , Iotova, V. , Hattersley, A. T. , & Ellard, S. (2017). Analysis of cell‐free fetal DNA for non‐invasive prenatal diagnosis in a family with neonatal diabetes. Diabetic Medicine, 34(4), 582–585. 27477181 10.1111/dme.13180 PMC5096683

[humu23995-bib-0022] De Franco, E. , Flanagan, S. E. , Houghton, J. A. , Lango Allen, H. , Mackay, D. J. , Temple, I. K. , … Hattersley, A. T. (2015). The effect of early, comprehensive genomic testing on clinical care in neonatal diabetes: An international cohort study. Lancet, 386(9997), 957–963. 26231457 10.1016/S0140-6736(15)60098-8 PMC4772451

[humu23995-bib-0023] De Leon, D. D. , Li, C. , Delson, M. I. , Matschinsky, F. M. , Stanley, C. A. , & Stoffers, D. A. (2008). Exendin‐(9‐39) corrects fasting hypoglycemia in SUR‐1‐/‐ mice by lowering cAMP in pancreatic beta‐cells and inhibiting insulin secretion. Journal of Biological Chemistry, 283(38), 25786–25793. 18635551 10.1074/jbc.M804372200 PMC3258866

[humu23995-bib-0024] Demirbilek, H. , Shah, P. , Arya, V. B. , Hinchey, L. , Flanagan, S. E. , Ellard, S. , & Hussain, K. (2014). Long‐term follow‐up of children with congenital hyperinsulinism on octreotide therapy. Journal of Clinical Endocrinology and Metabolism, 99(10), 3660–3667. 24937539 10.1210/jc.2014-1866

[humu23995-bib-0025] Edghill, E. L. , Gloyn, A. L. , Goriely, A. , Harries, L. W. , Flanagan, S. E. , Rankin, J. , … Ellard, S. (2007). Origin of de novo KCNJ11 mutations and risk of neonatal diabetes for subsequent siblings. Journal of Clinical Endocrinology and Metabolism, 92(5), 1773–1777. 17327377 10.1210/jc.2006-2817 PMC7611879

[humu23995-bib-0026] Ellard, S. , Flanagan, S. E. , Girard, C. A. , Patch, A. M. , Harries, L. W. , Parrish, A. , … Ashcroft, F. M. (2007). Permanent neonatal diabetes caused by dominant, recessive, or compound heterozygous SUR1 mutations with opposite functional effects. American Journal of Human Genetics, 81(2), 375–382. 17668386 10.1086/519174 PMC1950816

[humu23995-bib-0027] Fendler, W. , Pietrzak, I. , Brereton, M. F. , Lahmann, C. , Gadzicki, M. , Bienkiewicz, M. , … Mlynarski, W. M. (2013). Switching to sulphonylureas in children with iDEND syndrome caused by KCNJ11 mutations results in improved cerebellar perfusion. Diabetes Care, 36(8), 2311–2316. 23462667 10.2337/dc12-2166 PMC3714477

[humu23995-bib-0028] Fernandez‐Marmiesse, A. , Salas, A. , Vega, A. , Fernandez‐Lorenzo, J. R. , Barreiro, J. , & Carracedo, A. (2006). Mutation spectra of ABCC8 gene in Spanish patients with hyperinsulinism of infancy (HI). Human Mutation, 27(2), 214. 10.1002/humu.9401 16429405

[humu23995-bib-0029] Flanagan, S. E. , Clauin, S. , Bellanne‐Chantelot, C. , de Lonlay, P. , Harries, L. W. , Gloyn, A. L. , & Ellard, S. (2009). Update of mutations in the genes encoding the pancreatic beta‐cell K(ATP) channel subunits Kir6.2 (KCNJ11) and sulfonylurea receptor 1 (ABCC8) in diabetes mellitus and hyperinsulinism. Human Mutation, 30(2), 170–180. 18767144 10.1002/humu.20838

[humu23995-bib-0030] Flanagan, S. E. , Dung, V. C. , Houghton, J. A. L. , De Franco, E. , Ngoc, C. T. B. , Damhuis, A. , … Ellard, S. (2017). An ABCC8 nonsense mutation causing neonatal diabetes through altered transcript expression. Journal of Clinical Research in Pediatric Endocrinology, 9(3), 260–264. 28663158 10.4274/jcrpe.4624 PMC5596808

[humu23995-bib-0031] Flanagan, S. E. , Edghill, E. L. , Gloyn, A. L. , Ellard, S. , & Hattersley, A. T. (2006). Mutations in KCNJ11, which encodes Kir6.2, are a common cause of diabetes diagnosed in the first 6 months of life, with the phenotype determined by genotype. Diabetologia, 49(6), 1190–1197. 16609879 10.1007/s00125-006-0246-z

[humu23995-bib-0032] Flanagan, S. E. , Xie, W. , Caswell, R. , Damhuis, A. , Vianey‐Saban, C. , Akcay, T. , … Ellard, S. (2013). Next‐generation sequencing reveals deep intronic cryptic ABCC8 and HADH splicing founder mutations causing hyperinsulinism by pseudoexon activation. American Journal of Human Genetics, 92(1), 131–136. 23273570 10.1016/j.ajhg.2012.11.017 PMC3542457

[humu23995-bib-0033] Florez, J. C. , Burtt, N. , de Bakker, P. I. W. , Almgren, P. , Tuomi, T. , Holmkvist, J. , … Altshuler, D. (2004). Haplotype structure and genotype‐phenotype correlations of the sulfonylurea receptor and the islet ATP‐sensitive potassium channel gene region. Diabetes, 53(5), 1360–1368. 15111507 10.2337/diabetes.53.5.1360

[humu23995-bib-0034] Fokkema, I. F. , Taschner, P. E. , Schaafsma, G. C. , Celli, J. , Laros, J. F. , & den Dunnen, J. T. (2011). LOVD v.2.0: The next generation in gene variant databases. Human Mutation, 32(5), 557–563. 21520333 10.1002/humu.21438

[humu23995-bib-0035] Glaser, B. , Thornton, P. , Otonkoski, T. , & Junien, C. (2000). Genetics of neonatal hyperinsulinism. Archives of Disease in Childhood. Fetal and Neonatal Edition, 82(2), F79–F86. 10685979 10.1136/fn.82.2.F79 PMC1721059

[humu23995-bib-0036] Gloyn, A. L. , Cummings, E. A. , Edghill, E. L. , Harries, L. W. , Scott, R. , Costa, T. , … Ellard, S. (2004). Permanent neonatal diabetes due to paternal germline mosaicism for an activating mutation of the KCNJ11 Gene encoding the Kir6.2 subunit of the beta‐cell potassium adenosine triphosphate channel. Journal of Clinical Endocrinology and Metabolism, 89(8), 3932–3935. 15292329 10.1210/jc.2004-0568

[humu23995-bib-0037] Gloyn, A. L. , Diatloff‐Zito, C. , Edghill, E. L. , Bellanne‐Chantelot, C. , Nivot, S. , Coutant, R. , … Robert, J. J. (2006a). KCNJ11 activating mutations are associated with developmental delay, epilepsy and neonatal diabetes syndrome and other neurological features. European Journal of Human Genetics, 14(7), 824–830. 16670688 10.1038/sj.ejhg.5201629

[humu23995-bib-0038] Gloyn, A. L. , Pearson, E. R. , Antcliff, J. F. , Proks, P. , Bruining, G. J. , Slingerland, A. S. , … Hattersley, A. T. (2004). Activating mutations in the gene encoding the ATP‐sensitive potassium‐channel subunit Kir6.2 and permanent neonatal diabetes. New England Journal of Medicine, 350(18), 1838–1849. 15115830 10.1056/NEJMoa032922

[humu23995-bib-0039] Gloyn, A. L. , Reimann, F. , Girard, C. , Edghill, E. L. , Proks, P. , Pearson, E. R. , … Hattersley, A. T. (2005). Relapsing diabetes can result from moderately activating mutations in KCNJ11. Human Molecular Genetics, 14(7), 925–934. 15718250 10.1093/hmg/ddi086

[humu23995-bib-0040] Gloyn, A. L. , Siddiqui, J. , & Ellard, S. (2006). Mutations in the genes encoding the pancreatic beta‐cell KATP channel subunits Kir6.2 (KCNJ11) and SUR1 (ABCC8) in diabetes mellitus and hyperinsulinism. Human Mutation, 27(3), 220–231. 16416420 10.1002/humu.20292

[humu23995-bib-0041] Gloyn, A. L. , Weedon, M. N. , Owen, K. R. , Turner, M. J. , Knight, B. A. , Hitman, G. , … Frayling, T. M. (2003). Large‐scale association studies of variants in genes encoding the pancreatic beta‐cell KATP channel subunits Kir6.2 (KCNJ11) and SUR1 (ABCC8) confirm that the KCNJ11 E23K variant is associated with type 2 diabetes. Diabetes, 52(2), 568–572. 12540637 10.2337/diabetes.52.2.568

[humu23995-bib-0042] Grulich‐Henn, J. , Wagner, V. , Thon, A. , Schober, E. , Marg, W. , Kapellen, T. M. , … Holl, R. W. (2010). Entities and frequency of neonatal diabetes: Data from the diabetes documentation and quality management system (DPV). Diabetic Medicine, 27(6), 709–712. 20546293 10.1111/j.1464-5491.2010.02965.x

[humu23995-bib-0043] Han, B. , Mohamed, Z. , Estebanez, M. S. , Craigie, R. J. , Newbould, M. , Cheesman, E. , … Dunne, M. J. (2017). Atypical forms of congenital hyperinsulinism in infancy are associated with mosaic patterns of immature islet cells. Journal of Clinical Endocrinology and Metabolism, 102(9), 3261–3267. 28605545 10.1210/jc.2017-00158 PMC5587070

[humu23995-bib-0044] Hartemann‐Heurtier, A. , Simon, A. , Bellanne‐Chantelot, C. , Reynaud, R. , Cave, H. , Polak, M. , … Grimaldi, A. (2009). Mutations in the ABCC8 gene can cause autoantibody‐negative insulin‐dependent diabetes. Diabetes & Metabolism, 35(3), 233–235. 19342262 10.1016/j.diabet.2009.01.003

[humu23995-bib-0045] Hattersley, A. T. , & Ashcroft, F. M. (2005). Activating mutations in Kir6.2 and neonatal diabetes: New clinical syndromes, new scientific insights, and new therapy. Diabetes, 54(9), 2503–2513. 16123337 10.2337/diabetes.54.9.2503

[humu23995-bib-0046] Helleskov, A. , Melikyan, M. , Globa, E. , Shcherderkina, I. , Poertner, F. , Larsen, A. M. , … Christesen, H. T. (2017). Both low blood glucose and insufficient treatment confer risk of neurodevelopmental impairment in congenital hyperinsulinism: A multinational cohort study. Frontiers in Endocrinology(Lausanne), 8, 156. 10.3389/fendo.2017.00156 PMC5502348 28740482

[humu23995-bib-0047] Herrera, A. , Vajravelu, M. E. , Givler, S. , Mitteer, L. , Avitabile, C. M. , Lord, K. , & De Leon, D. D. (2018). Prevalence of adverse events in children with congenital hyperinsulinism treated with diazoxide. Journal of Clinical Endocrinology and Metabolism, 103(12), 4365–4372. 30247666 10.1210/jc.2018-01613 PMC6207144

[humu23995-bib-0048] Houghton, J. A. , Banerjee, I. , Shaikh, G. , Jabbar, S. , Laver, T. W. , Cheesman, E. , … Dunne, M. J. (2019). Unravelling the genetic causes of mosaic islet morphology in congenital hyperinsulinism. The Journal of Pathology, 6(6), 12–16. https://www.diabetesgenes.org/ 31577849 10.1002/cjp2.144 PMC6966704

[humu23995-bib-0049] Hugill, A. , Shimomura, K. , Ashcroft, F. M. , & Cox, R. D. (2010). A mutation in KCNJ11 causing human hyperinsulinism (Y12X) results in a glucose‐intolerant phenotype in the mouse. Diabetologia, 53(11), 2352–2356. 20694718 10.1007/s00125-010-1866-x PMC5894805

[humu23995-bib-0050] Huopio, H. , Otonkoski, T. , Vauhkonen, I. , Reimann, F. , Ashcroft, F. M. , & Laakso, M. (2003). A new subtype of autosomal dominant diabetes attributable to a mutation in the gene for sulfonylurea receptor 1. Lancet, 361(9354), 301–307. 12559865 10.1016/S0140-6736(03)12325-2

[humu23995-bib-0051] Hussain, K. , Flanagan, S. E. , Smith, V. V. , Ashworth, M. , Day, M. , Pierro, A. , & Ellard, S. (2008). An ABCC8 gene mutation and mosaic uniparental isodisomy resulting in atypical diffuse congenital hyperinsulinism. Diabetes, 57(1), 259–263. 17942822 10.2337/db07-0998

[humu23995-bib-0052] Ismail, D. , Kapoor, R. R. , Smith, V. V. , Ashworth, M. , Blankenstein, O. , Pierro, A. , … Hussain, K. (2012). The heterogeneity of focal forms of congenital hyperinsulinism. Journal of Clinical Endocrinology and Metabolism, 97(1), E94–E99. 22031516 10.1210/jc.2011-1628 PMC7611920

[humu23995-bib-0053] Jack, M. M. , Walker, R. M. , Thomsett, M. J. , Cotterill, A. M. , & Bell, J. R. (2000). Histologic findings in persistent hyperinsulinemic hypoglycemia of infancy: Australian experience. Pediatric and Developmental Pathology, 3(6), 532–547. 11000331 10.1007/s100240010117

[humu23995-bib-0054] Kapoor, R. R. , Flanagan, S. E. , Arya, V. B. , Shield, J. P. , Ellard, S. , & Hussain, K. (2013). Clinical and molecular characterisation of 300 patients with congenital hyperinsulinism. European Journal of Endocrinology, 168(4), 557–564. 23345197 10.1530/EJE-12-0673 PMC3599069

[humu23995-bib-0055] Karschin, C. , Ecke, C. , Ashcroft, F. M. , & Karschin, A. (1997). Overlapping distribution of K(ATP) channel‐forming Kir6.2 subunit and the sulfonylurea receptor SUR1 in rodent brain. FEBS Letters, 401(1), 59–64. 9003806 10.1016/s0014-5793(96)01438-x

[humu23995-bib-0056] Koster, J. C. , Marshall, B. A. , Ensor, N. , Corbett, J. A. , & Nichols, C. G. (2000). Targeted overactivity of beta cell K(ATP) channels induces profound neonatal diabetes. Cell, 100(6), 645–654. 10761930 10.1016/s0092-8674(00)80701-1

[humu23995-bib-0057] Koufakis, T. , Sertedaki, A. , Tatsi, E. B. , Trakatelli, C. M. , Karras, S. N. , Manthou, E. , … Kotsa, K. (2019). First Report of Diabetes Phenotype due to a Loss‐of‐Function ABCC8 Mutation Previously Known to Cause Congenital Hyperinsulinism. Case Reports in Genetics, 2019, 3654618–5. 31110826 10.1155/2019/3654618 PMC6487141

[humu23995-bib-0058] Kumaraguru, J. , Flanagan, S. E. , Greeley, S. A. , Nuboer, R. , Stoy, J. , Philipson, L. H. , … Rubio‐Cabezas, O. (2009). Tooth discoloration in patients with neonatal diabetes after transfer onto glibenclamide: A previously unreported side effect. Diabetes Care, 32(8), 1428–1430. 19435956 10.2337/dc09-0280 PMC2713626

[humu23995-bib-0059] Landmeier, K. A. , Lanning, M. , Carmody, D. , Greeley, S. A. W. , & Msall, M. E. (2017). ADHD, learning difficulties and sleep disturbances associated with KCNJ11‐related neonatal diabetes. Pediatric Diabetes, 18(7), 518–523. 27555491 10.1111/pedi.12428 PMC5720354

[humu23995-bib-0060] Lanning, M. S. , Carmody, D. , Szczerbinski, L. , Letourneau, L. R. , Naylor, R. N. , & Greeley, S. A. W. (2018). Hypoglycemia in sulfonylurea‐treated KCNJ11‐neonatal diabetes: Mild‐moderate symptomatic episodes occur infrequently but none involving unconsciousness or seizures. Pediatric Diabetes, 19(3), 393–397. 29205704 10.1111/pedi.12599 PMC5918230

[humu23995-bib-0061] Lek, M. , Karczewski, K. J. , Minikel, E. V. , Samocha, K. E. , Banks, E. , Fennell, T. , … MacArthur, D. G. (2016). Analysis of protein‐coding genetic variation in 60,706 humans. Nature, 536(7616), 285–291. 27535533 10.1038/nature19057 PMC5018207

[humu23995-bib-0062] Liss, B. , Bruns, R. , & Roeper, J. (1999). Alternative sulfonylurea receptor expression defines metabolic sensitivity of K‐ATP channels in dopaminergic midbrain neurons. EMBO Journal, 18(4), 833–846. 10022826 10.1093/emboj/18.4.833 PMC1171176

[humu23995-bib-0063] de Lonlay, P. , Fournet, J. C. , Rahier, J. , Gross‐Morand, M. S. , Poggi‐Travert, F. , Foussier, V. , … Junien, C. (1997). Somatic deletion of the imprinted 11p15 region in sporadic persistent hyperinsulinemic hypoglycemia of infancy is specific of focal adenomatous hyperplasia and endorses partial pancreatectomy. Journal of Clinical Investigation, 100(4), 802–807. 9259578 10.1172/JCI119594 PMC508251

[humu23995-bib-0064] de Lonlay, P. , Fournet, J. C. , Touati, G. , Groos, M. S. , Martin, D. , Sevin, C. , … Robert, J. J. (2002). Heterogeneity of persistent hyperinsulinaemic hypoglycaemia. A series of 175 cases. European Journal of Pediatrics, 161(1), 37–48. 11808879 10.1007/s004310100847

[humu23995-bib-0065] Massa, O. , Iafusco, D. , D'Amato, E. , Gloyn, A. L. , Hattersley, A. T. , Pasquino, B. , … Barbetti, F. (2005). KCNJ11 activating mutations in Italian patients with permanent neonatal diabetes. Human Mutation, 25(1), 22–27. 15580558 10.1002/humu.20124

[humu23995-bib-0066] Mathew, P. M. , Young, J. M. , Abu‐Osba, Y. K. , Mulhern, B. D. , Hammoudi, S. , Hamdan, J. A. , & Sa'di, A. R. (1988). Persistent neonatal hyperinsulinism. Clinical Pediatrics (Cleveland, OH), 27(3), 148–151. 10.1177/000992288802700307 3342599

[humu23995-bib-0067] Miki, T. , Nagashima, K. , Tashiro, F. , Kotake, K. , Yoshitomi, H. , Tamamoto, A. , … Seino, S. (1998). Defective insulin secretion and enhanced insulin action in KATP channel‐deficient mice. Proceedings of the National Academy of Sciences of the United States of America, 95(18), 10402–10406. 9724715 10.1073/pnas.95.18.10402 PMC27906

[humu23995-bib-0068] Mohnike, K. , Wieland, I. , Barthlen, W. , Vogelgesang, S. , Empting, S. , Mohnike, W. , … Zenker, M. (2014). Clinical and genetic evaluation of patients with KATP channel mutations from the German registry for congenital hyperinsulinism. Hormone research in pædiatrics, 81(3), 156–168. 10.1159/000356905 24401662

[humu23995-bib-0069] Myngheer, N. , Allegaert, K. , Hattersley, A. , McDonald, T. , Kramer, H. , Ashcroft, F. M. , … Casteels, K. (2014). Fetal macrosomia and neonatal hyperinsulinemic hypoglycemia associated with transplacental transfer of sulfonylurea in a mother with KCNJ11‐related neonatal diabetes. Diabetes Care, 37(12), 3333–3335. 25231897 10.2337/dc14-1247 PMC5894804

[humu23995-bib-0070] Nestorowicz, A. (1996). Mutations in the sulonylurea receptor gene are associated with familial hyperinsulinism in Ashkenazi Jews. Human Molecular Genetics, 5(11), 1813–1822. 8923011 10.1093/hmg/5.11.1813

[humu23995-bib-0071] Ng, C. M. , Tang, F. , Seeholzer, S. H. , Zou, Y. , & De Leon, D. D. (2018). Population pharmacokinetics of exendin‐(9‐39) and clinical dose selection in patients with congenital hyperinsulinism. British Journal of Clinical Pharmacology, 84(3), 520–532. 29077992 10.1111/bcp.13463 PMC5809353

[humu23995-bib-0072] Nichols, C. G. , Shyng, S. L. , Nestorowicz, A. , Glaser, B. , Clement, J. Pt , Gonzalez, G. , … Bryan, J. (1996). Adenosine diphosphate as an intracellular regulator of insulin secretion. Science, 272(5269), 1785–1787. 8650576 10.1126/science.272.5269.1785

[humu23995-bib-0073] Nielsen, E. M. , Hansen, L. , Carstensen, B. , Echwald, S. M. , Drivsholm, T. , Glumer, C. , … Pedersen, O. (2003). The E23K variant of Kir6.2 associates with impaired post‐OGTT serum insulin response and increased risk of type 2 diabetes. Diabetes, 52(2), 573–577. 12540638 10.2337/diabetes.52.2.573

[humu23995-bib-0074] Otonkoski, T. , Ammala, C. , Huopio, H. , Cote, G. J. , Chapman, J. , Cosgrove, K. , … Thomas, P. M. (1999). A point mutation inactivating the sulfonylurea receptor causes the severe form of persistent hyperinsulinemic hypoglycemia of infancy in Finland. Diabetes, 48(2), 408–415. 10334322 10.2337/diabetes.48.2.408

[humu23995-bib-0075] Otonkoski, T. , Nanto‐Salonen, K. , Seppanen, M. , Veijola, R. , Huopio, H. , Hussain, K. , … Minn, H. (2006). Noninvasive diagnosis of focal hyperinsulinism of infancy with [18F]‐DOPA positron emission tomography. Diabetes, 55(1), 13–18. 16380471

[humu23995-bib-0076] Patch, A. M. , Flanagan, S. E. , Boustred, C. , Hattersley, A. T. , & Ellard, S. (2007). Mutations in the ABCC8 gene encoding the SUR1 subunit of the KATP channel cause transient neonatal diabetes, permanent neonatal diabetes or permanent diabetes diagnosed outside the neonatal period. Diabetes, obesity & metabolism, 9(Suppl 2), 28–39. 10.1111/j.1463-1326.2007.00772.x PMC7611803 17919176

[humu23995-bib-0077] Patel, P. , Charles, L. , Corbin, J. , Goldfine, I. D. , Johnson, K. , Rubin, P. , & De Leon, D. D. (2018). A unique allosteric insulin receptor monoclonal antibody that prevents hypoglycemia in the SUR‐1(‐/‐) mouse model of KATP hyperinsulinism. mAbs, 10(5), 796–802. 29589989 10.1080/19420862.2018.1457599 PMC6150619

[humu23995-bib-0078] Pearson, E. R. , Flechtner, I. , Njølstad, P. R. , Malecki, M. T. , Flanagan, S. E. , Larkin, B. , … Hattersley, A. T. (2006). Switching from insulin to oral sulfonylureas in patients with diabetes due to Kir6.2 mutations. New England Journal of Medicine, 355(5), 467–477. 16885550 10.1056/NEJMoa061759

[humu23995-bib-0079] Pinney, S. E. , Ganapathy, K. , Bradfield, J. , Stokes, D. , Sasson, A. , Mackiewicz, K. , … Stanley, C. A. (2013). Dominant form of congenital hyperinsulinism maps to HK1 region on 10q. Hormone Research in Paediatrics, 80(1), 18–27. 23859901 10.1159/000351943 PMC3876732

[humu23995-bib-0080] Powell, P. D. , Bellanne‐Chantelot, C. , Flanagan, S. E. , Ellard, S. , Rooman, R. , Hussain, K. , … Cosgrove, K. E. (2011). In vitro recovery of ATP‐sensitive potassium channels in {beta}‐cells from patients with congenital hyperinsulinism of infancy. Diabetes, 60(4), 1223–1228. 21411514 10.2337/db10-1443 PMC3064095

[humu23995-bib-0081] Proks, P. , Arnold, A. L. , Bruining, J. , Girard, C. , Flanagan, S. E. , Larkin, B. , … Ellard, S. (2006). A heterozygous activating mutation in the sulphonylurea receptor SUR1 (ABCC8) causes neonatal diabetes. Human Molecular Genetics, 15(11), 1793–1800. 16613899 10.1093/hmg/ddl101

[humu23995-bib-0082] Rafiq, M. , Flanagan, S. E. , Patch, A. M. , Shields, B. M. , Ellard, S. , & Hattersley, A. T. (2008). Effective treatment with oral sulfonylureas in patients with diabetes due to sulfonylurea receptor 1 (SUR1) mutations. Diabetes Care, 31(2), 204–209. 18025408 10.2337/dc07-1785 PMC7611807

[humu23995-bib-0083] Rahier, J. , Falt, K. , Muntefering, H. , Becker, K. , Gepts, W. , & Falkmer, S. (1984). The basic structural lesion of persistent neonatal hypoglycaemia with hyperinsulinism: Deficiency of pancreatic D cells or hyperactivity of B cells? Diabetologia, 26(4), 282–289. 6376236 10.1007/BF00283651

[humu23995-bib-0084] Richards, S. , Aziz, N. , Bale, S. , Bick, D. , Das, S. , Gastier‐Foster, J. , … Rehm, H. L. (2015). Standards and guidelines for the interpretation of sequence variants: A joint consensus recommendation of the American College of Medical Genetics and Genomics and the Association for Molecular Pathology. Genetics in Medicine, 17(5), 405–424. 25741868 10.1038/gim.2015.30 PMC4544753

[humu23995-bib-0085] Rorsman, P. , & Trube, G. (1985). Glucose dependent K + ‐channels in pancreatic beta‐cells are regulated by intracellular ATP. Pflügers Archiv: European Journal of Physiology, 405(4), 305–309. 2417189 10.1007/BF00595682

[humu23995-bib-0086] Sagen, J. V. , Raeder, H. , Hathout, E. , Shehadeh, N. , Gudmundsson, K. , Baevre, H. , … Njolstad, P. R. (2004). Permanent neonatal diabetes due to mutations in KCNJ11 encoding Kir6.2: Patient characteristics and initial response to sulfonylurea therapy. Diabetes, 53(10), 2713–2718. 15448106 10.2337/diabetes.53.10.2713

[humu23995-bib-0087] Sakura, H. , Ammala, C. , Smith, P. A. , Gribble, F. M. , & Ashcroft, F. M. (1995). Cloning and functional expression of the cDNA encoding a novel ATP‐sensitive potassium channel subunit expressed in pancreatic beta‐cells, brain, heart and skeletal muscle. FEBS Letters, 377(3), 338–344. 8549751 10.1016/0014-5793(95)01369-5

[humu23995-bib-0088] Schmahmann, J. D. , & Sherman, J. C. (1998). The cerebellar cognitive affective syndrome. Brain, 121(Pt 4), 561–579. 9577385 10.1093/brain/121.4.561

[humu23995-bib-0089] Seghers, V. , Nakazaki, M. , DeMayo, F. , Aguilar‐Bryan, L. , & Bryan, J. (2000). Sur1 knockout mice. A model for K(ATP) channel‐independent regulation of insulin secretion. Journal of Biological Chemistry, 275(13), 9270–9277. 10734066 10.1074/jbc.275.13.9270

[humu23995-bib-0090] Sempoux, C. , Capito, C. , Bellanne‐Chantelot, C. , Verkarre, V. , de Lonlay, P. , Aigrain, Y. , … Rahier, J. (2011). Morphological mosaicism of the pancreatic islets: A novel anatomopathological form of persistent hyperinsulinemic hypoglycemia of infancy. Journal of Clinical Endocrinology and Metabolism, 96(12), 3785–3793. 21956412 10.1210/jc.2010-3032

[humu23995-bib-0091] Senniappan, S. , Alexandrescu, S. , Tatevian, N. , Shah, P. , Arya, V. , Flanagan, S. , … Hussain, K. (2014). Sirolimus therapy in infants with severe hyperinsulinemic hypoglycemia. New England Journal of Medicine, 370(12), 1131–1137. 24645945 10.1056/NEJMoa1310967

[humu23995-bib-0092] Shah, R. P. , Spruyt, K. , Kragie, B. C. , Greeley, S. A. , & Msall, M. E. (2012). Visuomotor performance in KCNJ11‐related neonatal diabetes is impaired in children with DEND‐associated mutations and may be improved by early treatment with sulfonylureas. Diabetes Care, 35(10), 2086–2088. 22855734 10.2337/dc11-2225 PMC3447845

[humu23995-bib-0093] Shepherd, M. , Brook, A. J. , Chakera, A. J. , & Hattersley, A. T. (2017). Management of sulfonylurea‐treated monogenic diabetes in pregnancy: Implications of placental glibenclamide transfer. Diabetic Medicine, 34(10), 1332–1339. 28556992 10.1111/dme.13388 PMC5612398

[humu23995-bib-0094] Shepherd, M. , Shields, B. , Hammersley, S. , Hudson, M. , McDonald, T. J. , Colclough, K. , … Hattersley, A. T. (2016). Systematic population screening, using biomarkers and genetic testing, identifies 2.5% of the U.K. pediatric diabetes population with monogenic diabetes. Diabetes Care, 39(11), 1879–1888. 27271189 10.2337/dc16-0645 PMC5018394

[humu23995-bib-0095] Shields, B. M. , Shepherd, M. , Hudson, M. , McDonald, T. J. , Colclough, K. , Peters, J. , … Hattersley, A. T. (2017). Population‐based assessment of a biomarker‐based screening pathway to aid diagnosis of monogenic diabetes in young‐onset patients. Diabetes Care, 40(8), 1017–1025. 28701371 10.2337/dc17-0224 PMC5570522

[humu23995-bib-0096] Shimomura, K. , Tusa, M. , Iberl, M. , Brereton, M. F. , Kaizik, S. , Proks, P. , … Ashcroft, F. M. (2013). A mouse model of human hyperinsulinism produced by the E1506K mutation in the sulphonylurea receptor SUR1. Diabetes, 62(11), 3797–3806. 23903354 10.2337/db12-1611 PMC3806602

[humu23995-bib-0097] Snider, K. E. , Becker, S. , Boyajian, L. , Shyng, S. L. , MacMullen, C. , Hughes, N. , … Ganguly, A. (2013). Genotype and phenotype correlations in 417 children with congenital hyperinsulinism. Journal of Clinical Endocrinology and Metabolism, 98(2), E355–E363. 23275527 10.1210/jc.2012-2169 PMC3565119

[humu23995-bib-0098] Stoy, J. , Greeley, S. A. , Paz, V. P. , Ye, H. , Pastore, A. N. , Skowron, K. B. , … Philipson, L. H. (2008). Diagnosis and treatment of neonatal diabetes: A United States experience. Pediatric Diabetes, 9(5), 450–459. 18662362 10.1111/j.1399-5448.2008.00433.x PMC2574846

[humu23995-bib-0099] Tarasov, A. I. , Nicolson, T. J. , Riveline, J. P. , Taneja, T. K. , Baldwin, S. A. , Baldwin, J. M. , … Rutter, G. A. (2008). A rare mutation in ABCC8/SUR1 leading to altered ATP‐sensitive K + channel activity and beta‐cell glucose sensing is associated with type 2 diabetes in adults. Diabetes, 57(6), 1595–1604. 18346985 10.2337/db07-1547 PMC6101196

[humu23995-bib-0100] Taschenberger, G. , Mougey, A. , Shen, S. , Lester, L. B. , LaFranchi, S. , & Shyng, S. L. (2002). Identification of a familial hyperinsulinism‐causing mutation in the sulfonylurea receptor 1 that prevents normal trafficking and function of KATP channels. Journal of Biological Chemistry, 277(19), 17139–17146. 11867634 10.1074/jbc.M200363200

[humu23995-bib-0101] Thomas, P. , Ye, Y. , & Lightner, E. (1996). Mutation of the pancreatic islet inward rectifier Kir6.2 also leads to familial persistent hyperinsulinemic hypoglycemia of infancy. Human Molecular Genetics, 5(11), 1809–1812. 8923010 10.1093/hmg/5.11.1809

[humu23995-bib-0102] Thomas, P. M. , Cote, G. J. , Wohllk, N. , Haddad, B. , Mathew, P. M. , Rabl, W. , … Bryan, J. (1995). Mutations in the sulfonylurea receptor gene in familial persistent hyperinsulinemic hypoglycemia of infancy. Science, 268(5209), 426–429. 7716548 10.1126/science.7716548

[humu23995-bib-0103] Thornton, P. S. , Stanley, C. A. , De Leon, D. D. , Harris, D. , Haymond, M. W. , Hussain, K. , … Wolfsdorf, J. I. (2015). Recommendations from the pediatric endocrine society for evaluation and management of persistent hypoglycemia in neonates, infants, and children. Journal of Pediatrics, 167(2), 238–245. 25957977 10.1016/j.jpeds.2015.03.057 PMC11891912

[humu23995-bib-0104] Thurber, B. W. , Carmody, D. , Tadie, E. C. , Pastore, A. N. , Dickens, J. T. , Wroblewski, K. E. , … Greeley, S. A. , United States Neonatal Diabetes Working G. (2015). Age at the time of sulfonylurea initiation influences treatment outcomes in KCNJ11‐related neonatal diabetes. Diabetologia, 58(7), 1430–1435. 25877689 10.1007/s00125-015-3593-9 PMC4641523

[humu23995-bib-0105] Tornovsky, S. , Crane, A. , Cosgrove, K. E. , Hussain, K. , Lavie, J. , Heyman, M. , … Glaser, B. (2004). Hyperinsulinism of infancy: Novel ABCC8 and KCNJ11 mutations and evidence for additional locus heterogeneity. Journal of Clinical Endocrinology and Metabolism, 89(12), 6224–6234. 15579781 10.1210/jc.2004-1233

[humu23995-bib-0106] Wiedemann, B. , Schober, E. , Waldhoer, T. , Koehle, J. , Flanagan, S. E. , Mackay, D. J. , … Hofer, S. (2010). Incidence of neonatal diabetes in Austria‐calculation based on the Austrian Diabetes Register. Pediatric Diabetes, 11(1), 18–23. 19496964 10.1111/j.1399-5448.2009.00530.x

[humu23995-bib-0107] Zung, A. , Glaser, B. , Nimri, R. , & Zadik, Z. (2004). Glibenclamide treatment in permanent neonatal diabetes mellitus due to an activating mutation in Kir6.2. Journal of Clinical Endocrinology and Metabolism, 89(11), 5504–5507. 15531505 10.1210/jc.2004-1241

