# Supplementary material for: Update of variants identified in the pancreatic β‐cell KATP channel genes KCNJ11 and ABCC8 in individuals with congenital hyperinsulinism and diabetes
Source: Hum Mutat. 2020 Feb 17;41(5):884–905. doi: 10.1002/humu.23995 (PMC7187370; doi:10.1002/humu.23995)
Supplement: Supplementary file 1 — Supporting information [file HUMU-41-884-s001.DOCX]

**Supplementary Information:**

**Collation genetic variant tables.**

Published variants in the *ABCC8* and *KCNJ11* genes were identified following a search of the U.S. National Library of Medicine, Medline Database.

Unpublished *ABCC8* and *KCNJ11* variants identified by 5 international molecular genetic screening laboratories in the UK, Denmark, France and the United States of America were assessed using the following criteria for pathogenicity: 1) confirmed *de novo*; 2) supported by *in vitro* or *in vivo* functional studies (including evidence of sulphonylurea response in the patients); 3) identified in at least 2 unrelated patients with the same phenotype within the same laboratory. Unpublished inactivating variants were included in the pathogenic variants table if they fulfilled one or more of the following criteria: 1) being frameshift or nonsense; 2) affecting the canonical splice site ((+1, +2 or -1, -2); 3) confirmed *de novo*; 4) confirmed recessive in the context of diffuse disease, identified in 2 or more unrelated patients with the same phenotype and rare in GnomAD ([Lek, et al., 2016](#_ENREF_220)); and 5) heterozygous when paternally inherited with confirmed focal disease.

Variants were classified as disease causing (tables 1, 4 and Supp Table S1 and S4) if they fulfilled one or more of these criteria. Unpublished activating variants which did not fulfil at least one of the criteria were listed as variants of uncertain significance (tables 2 and 4 and Supp tables S2 and S4).

Unpublished variants were included in Supp Tables S3 and S6 if they were listed in public databases at a frequency above a carrier frequency compatible with neonatal diabetes, hyperinsulinism or later onset diabetes.

Variants which had been previously reported as pathogenic which have a GnomAD frequency compatible with disease frequency (as calculated by <http://cardiodb.org/allelefrequencyapp/>) were not re-assessed. We would therefore recommend referring to the cited papers and/or contacting the listed laboratories for further information on phenotypic features and treatment response observed in patients with each of these variants.

**Supp Table S1: Pathogenic variants identified in *KCNJ11* (NM_000525.3).** NDM = Neonatal Diabetes. PNDM = Permanent Neonatal Diabetes Mellitus. TNDM = Transient Neonatal Diabetes Mellitus. DEND = Developmental Delay and Neonatal Diabetes Syndrome. i-DEND = Intermediate DEND syndrome. HI = Hyperinsulinism. Ter = Termination codon. * in the Phenotype column highlights a new phenotype; the * in the Reference column indicates which laboratory has identified the variant in a patients with the new phenotype.

| Protein Change | **Nucleotide Change** | **Mutation Type** | **Protein domain (UniProt)** | **Phenotype** | **GnomAD AC** | **GnomAD MAF** | **Reference** |
| --- | --- | --- | --- | --- | --- | --- | --- |
| p.? | c.-656G>T | Upstream substitution |  | HI | 0 | 0 | ([Tornovsky, et al., 2004](#_ENREF_392)) |
| p.? | c.-54C>T | 5' UTR substitution |  | HI | 3 | 0.00009578 | ([Huopio, et al., 2002](#_ENREF_157)) |
| p.(Leu2Pro) | c.5T>C | Missense | Cytoplasmic | NDM | 0 | 0 | ([Alkorta-Aranburu, et al., 2014](#_ENREF_13)) |
| p.(Ser3Cys) | c.8C>G | Missense | Cytoplasmic | PNDM | 0 | 0 | ([Jose, 2009](#_ENREF_184)) |
| p.(Arg4Cys) | c.10C>T | Missense | Cytoplasmic | TNDM  PNDM | 6 | 0.00002150 | Exeter unpublished |
| p.(Tyr12Ter) | c.36C>A | Nonsense |  | HI | 0 | 0 | ([Hugill, et al., 2010](#_ENREF_156); [Nestorowicz, et al., 1997](#_ENREF_266)) |
| p.(Leu17Pro) | c.50T>C | Missense | Cytoplasmic | PNDM | 0 | 0 | Exeter unpublished |
| p.(Tyr26Ter) | c.78C>A | Nonsense |  | HI | 0 | 0 | Exeter unpublished |
| p.(Arg27Cys) | c.79C>T | Missense | Cytoplasmic | HI | 2 | 0.000007976 | Chicago unpublished |
| p.(Arg27His) | c.80G>A | Missense | Cytoplasmic | Later-onset diabetes | 5 | 0.00001772 | ([Liu, et al., 2013](#_ENREF_228)) |
| p.(Ala28Val) | c.83C>T | Missense | Cytoplasmic | HI | 1 | 0.000003987 | ([Yang, et al., 2011](#_ENREF_414)) |
| p.(Ala30_Arg34del) | c.81_95del | In-Frame Deletion | Cytoplasmic | PNDM | 0 | 0 | ([Craig, et al., 2009](#_ENREF_71)) |
| p.(Arg34Cys) | c.100C>T | Missense | Cytoplasmic | HI | 1 | 0.000003985 | ([Salomon-Estebanez, et al., 2016](#_ENREF_328); [Snider, et al., 2013](#_ENREF_356)) |
| p.(Arg34His) | c.101G>A | Missense | Cytoplasmic | HI | 1 | 0.000003985 | ([Albaqumi, et al., 2014](#_ENREF_12); [Bellanne-Chantelot, et al., 2010](#_ENREF_31); [Ni, et al., 2019](#_ENREF_268); [Ohkubo, et al., 2005](#_ENREF_275)) |
| p.(Phe35Val) | c.103T>G | Missense | Cytoplasmic | PNDM | 0 | 0 | ([Proks, et al., 2006b](#_ENREF_310); [Sagen, et al., 2004](#_ENREF_323)) |
| p.(Phe35Leu) | c.105T>G | Missense | Cytoplasmic | PNDM | 0 | 0 | ([Flechtner, et al., 2008](#_ENREF_115); [Polak and Cave, 2007](#_ENREF_300); [Proks, et al., 2006b](#_ENREF_310); [Vaxillaire, et al., 2004](#_ENREF_397)) |
| p.(Lys38Glu) | c.112A>G | Missense | Cytoplasmic | HI | 0 | 0 | Exeter unpublished |
| p.(Lys39Arg) | c.116A>G | Missense | Cytoplasmic | NDM | 0 | 0 | ([Zhang, et al., 2015a](#_ENREF_419)) |
| p.(Gly40Ser) | c.118G>A | Missense | Cytoplasmic | HI | 1 | 0.000003981 | ([Mohnike, et al., 2014](#_ENREF_256)) |
| p.(Gly40Asp) | c.119G>A | Missense | Cytoplasmic | HI | 0 | 0 | ([Dastamani, et al., 2019](#_ENREF_77); [Salomon-Estebanez, et al., 2016](#_ENREF_328); [Suchi, et al., 2006](#_ENREF_365)) |
| p.(Gly40Ala) | c.119G>C | Missense | Cytoplasmic | HI | 0 | 0 | Exeter unpublished |
| p.(Cys42Arg) | c.124T>C | Missense | Cytoplasmic | TNDM | 0 | 0 | ([Hashimoto, et al., 2017](#_ENREF_149); [Jahnavi, et al., 2013](#_ENREF_176); [Yorifuji, et al., 2005](#_ENREF_418)) |
| p.(His46Tyr) | c.136C>T | Missense | Cytoplasmic | PNDM  TNDM | 0 | 0 | ([Flanagan, et al., 2006](#_ENREF_110); [Girard, et al., 2006](#_ENREF_123); [Rica, et al., 2007](#_ENREF_316); [Stoy, et al., 2008](#_ENREF_362)) |
| p.(His46Arg) | c.137A>G | Missense | Cytoplasmic | NDM | 0 | 0 | ([Bennett, et al., 2015](#_ENREF_33); [Moritani, et al., 2013](#_ENREF_258)) |
| p.(His46Leu) | c.137A>T | Missense | Cytoplasmic | i-DEND | 0 | 0 | ([Gach, et al., 2009](#_ENREF_120); [Klupa, et al., 2010b](#_ENREF_205); [Mlynarski, et al., 2007](#_ENREF_253)) |
| p.(Asn48Asp) | c.142A>G | Missense | Cytoplasmic | TNDM | 0 | 0 | ([Girard, et al., 2006](#_ENREF_123); [Martin-Frias, et al., 2009](#_ENREF_242); [Rica, et al., 2007](#_ENREF_316)) |
| p.(Asn48Ile) | c.143A>T | Missense | Cytoplasmic | PNDM | 0 | 0 | ([Bennett, et al., 2015](#_ENREF_33); [Shahawy, et al., 2011](#_ENREF_339)) |
| p.(Ile49Phe) | c.145A>T | Missense | Cytoplasmic | TNDM | 0 | 0 | Exeter unpublished |
| p.(Ile49Asn) | c.146T>A | Missense | Cytoplasmic | HI | 0 | 0 | ([Fan, et al., 2015](#_ENREF_103)) |
| p.(Arg50Gly) | c.148C>G | Missense | Cytoplasmic | DEND | 0 | 0 | ([Suzuki, et al., 2007](#_ENREF_367)) |
| p.(Arg50Gln) | c.149G>A | Missense | Cytoplasmic | PNDM  TNDM | 0 | 0 | ([Flanagan, et al., 2006](#_ENREF_110); [Hashimoto, et al., 2017](#_ENREF_149); [Huopio, et al., 2016](#_ENREF_158); [Iafusco, et al., 2012](#_ENREF_162); [Ioannou, et al., 2011](#_ENREF_169); [Khurana, et al., 2012](#_ENREF_198); [Rica, et al., 2007](#_ENREF_316); [Shimomura, et al., 2006](#_ENREF_347); [Suzuki, et al., 2007](#_ENREF_367)) |
| p.(Arg50Pro) | c.149G>C | Missense | Cytoplasmic | DEND  i-DEND | 0 | 0 | ([Carmody, et al., 2014](#_ENREF_54); [Hashimoto, et al., 2017](#_ENREF_149); [Maejima, et al., 2015](#_ENREF_232); [Massa, et al., 2005](#_ENREF_247); [Pena-Almazan, 2015](#_ENREF_292); [Shimomura, et al., 2006](#_ENREF_347)) |
| p.(Glu51Ter) | c.151G>T | Nonsense |  | HI | 0 | 0 | ([Faletra, et al., 2013a](#_ENREF_101)) |
| p.(Glu51Ala) | c.152A>C | Missense | Cytoplasmic | PNDM | 0 | 0 | ([Flanagan, et al., 2010](#_ENREF_112)) |
| p.(Glu51Gly) | c.152A>G | Missense | Cytoplasmic | PNDM | 0 | 0 | Exeter unpublished |
| p.(Gln52Ter) | c.154C>T | Nonsense |  | HI | 1 | 0.000003981 | ([Rozenkova, et al., 2015](#_ENREF_318)) |
| p.(Gln52Arg) | c.155A>G | Missense | Cytoplasmic | DEND | 0 | 0 | ([Gloyn, et al., 2004b](#_ENREF_131); [Gonen, et al., 2012](#_ENREF_135); [Ioacara, et al., 2017](#_ENREF_168); [Koster, et al., 2005](#_ENREF_211); [Lin, et al., 2006a](#_ENREF_223); [Proks, et al., 2004](#_ENREF_306); [Shaw and Majzoub, 2009](#_ENREF_341); [Singh, et al., 2014](#_ENREF_353); [Sumnik, et al., 2007](#_ENREF_366)) |
| p.(Gln52Leu) | c.155A>T | Missense | Cytoplasmic | i-DEND | 0 | 0 | ([Doneray, et al., 2014](#_ENREF_90)) |
| p.(Gln52Leu) | c.155_156delinsTT | Missense | Cytoplasmic | PNDM | 0 | 0 | ([Huopio, et al., 2016](#_ENREF_158)) |
| p.(Gly53Ser) | c.157G>A | Missense | Cytoplasmic | TNDM | 0 | 0 | ([Cao, et al., 2016](#_ENREF_53); [Gloyn, et al., 2005](#_ENREF_132); [Koster, et al., 2008](#_ENREF_210)) |
| p.(Gly53Arg) | c.157G>C | Missense | Cytoplasmic | TNDM | 0 | 0 | ([Gloyn, et al., 2005](#_ENREF_132); [Koster, et al., 2008](#_ENREF_210)) |
| p.(Gly53Asp) | c.158G>A | Missense | Cytoplasmic | DEND  i-DEND  PNDM | 0 | 0 | ([Carmody, et al., 2014](#_ENREF_54); [Flanagan, et al., 2006](#_ENREF_110); [Globa, et al., 2015](#_ENREF_128); [Gurgel, et al., 2007](#_ENREF_140); [Hashimoto, et al., 2017](#_ENREF_149); [Kanakatti Shankar, et al., 2013](#_ENREF_187); [Klupa, et al., 2010b](#_ENREF_205); [Koster, et al., 2008](#_ENREF_210); [Vendramini, et al., 2010](#_ENREF_399)) |
| p.(Gly53Val) | c.158G>T | Missense | Cytoplasmic | TNDM | 0 | 0 | ([Khadilkar, et al., 2010](#_ENREF_194)) |
| p.(Gly53Asn) | N/A | Missense | Cytoplasmic | PNDM | 0 | 0 | ([Flechtner, et al., 2008](#_ENREF_115); [Polak and Cave, 2007](#_ENREF_300); [Vaxillaire, et al., 2004](#_ENREF_397)) |
| p.(Arg54Cys) | c.160C>T | Missense | Cytoplasmic | HI  Later-onset diabetes* | 2 | 0.000007078 | Exeter unpublished  Paris unpublished* |
| p.(Arg54His) | c.161G>A | Missense | Cytoplasmic | HI | 1 | 0.000003983 | ([Arya, et al., 2014b](#_ENREF_20); [Kapoor, et al., 2013](#_ENREF_188)) |
| p.(Phe55Leu) | c.165C>A | Missense | Cytoplasmic | HI | 1 | 0.000003982 | ([Lin, et al., 2006b](#_ENREF_227)) |
| p.(Leu56Gly) | c.166_167delinsGG | Missense | Cytoplasmic | HI | 0 | 0 | Exeter unpublished |
| p.(Val59Met) | c.175G>A | Missense | Cytoplasmic | i-DEND  PNDM  DEND | 0 | 0 | ([Al Senani, et al., 2018](#_ENREF_11); [Brereton, et al., 2014](#_ENREF_45); [Cao, et al., 2016](#_ENREF_53); [Clark, et al., 2012](#_ENREF_65); [Clark, et al., 2010](#_ENREF_66); [de Wet and Proks, 2015](#_ENREF_82); [Flanagan, et al., 2006](#_ENREF_110); [Flechtner, et al., 2008](#_ENREF_115); [Girard, et al., 2009](#_ENREF_124); [Globa, et al., 2015](#_ENREF_128); [Gloyn, et al., 2006](#_ENREF_130); [Gloyn, et al., 2004b](#_ENREF_131); [Hashimoto, et al., 2017](#_ENREF_149); [Irgens, et al., 2013](#_ENREF_170); [Jones and Hattersley, 2010](#_ENREF_183); [Kim, et al., 2007](#_ENREF_200); [Klupa, et al., 2010b](#_ENREF_205); [Koster, et al., 2005](#_ENREF_211); [Kumaraguru, et al., 2009](#_ENREF_213); [Lahmann, et al., 2014](#_ENREF_215); [Lin, et al., 2006a](#_ENREF_223); [Massa, et al., 2005](#_ENREF_247); [McTaggart, et al., 2013](#_ENREF_249); [Mohamadi, et al., 2009](#_ENREF_254); [Nieves-Rivera and Gonzalez-Pijem, 2011](#_ENREF_270); [Oka, et al., 2014](#_ENREF_277); [Prado-Carro, et al., 2014](#_ENREF_303); [Proks, et al., 2005a](#_ENREF_309); [Sagen, et al., 2004](#_ENREF_323); [Sang, et al., 2011](#_ENREF_330); [Shah, et al., 2012b](#_ENREF_338); [Shahawy, et al., 2011](#_ENREF_339); [Slingerland, et al., 2008](#_ENREF_355); [Stoy, et al., 2008](#_ENREF_362); [Ting, et al., 2009](#_ENREF_390); [Tonini, et al., 2006](#_ENREF_391); [Vaxillaire, et al., 2004](#_ENREF_397); [Zhang, et al., 2015a](#_ENREF_419)) |
| p.(Val59Ala) | c.176T>C | Missense | Cytoplasmic | DEND | 0 | 0 | ([Hashimoto, et al., 2017](#_ENREF_149); [Itoh, et al., 2013](#_ENREF_174); [Russo, et al., 2011b](#_ENREF_321); [Shah, et al., 2012b](#_ENREF_338); [Winkler, et al., 2009](#_ENREF_405)) |
| p.(Val59Gly) | c.176T>G | Missense | Cytoplasmic | DEND | 0 | 0 | ([Gloyn, et al., 2004b](#_ENREF_131); [Gonen, et al., 2012](#_ENREF_135); [Lin, et al., 2006a](#_ENREF_223); [Proks, et al., 2004](#_ENREF_306); [Proks, et al., 2005a](#_ENREF_309); [Winkler, et al., 2009](#_ENREF_405)) |
| p.(Phe60Tyr) | c.179T>A | Missense | Cytoplasmic | DEND | 0 | 0 | ([Mannikko, et al., 2010](#_ENREF_238)) |
| p.(Thr62Met) | c.185C>T | Missense | Cytoplasmic | HI | 3 | 0.00009563 | ([Arya, et al., 2014b](#_ENREF_20); [Mohnike, et al., 2014](#_ENREF_256); [Snider, et al., 2013](#_ENREF_356)) |
| p.(Thr62SerfsTer68) | c.185del | Frameshift |  | HI | 0 | 0 | Exeter unpublished |
| p.(Val64Met) | c.190G>A | Missense | Transmembrane | DEND | 0 | 0 | ([Hashimoto, et al., 2017](#_ENREF_149)) |
| p.(Val64Gly) | c.191T>G | Missense | Transmembrane | HI | 0 | 0 | ([Snider, et al., 2013](#_ENREF_356)) |
| p.(Lys67Asn) | c.201G>C | Missense | Transmembrane | HI | 7 | 0.00002790 | ([Huopio, et al., 2002](#_ENREF_157); [Reimann, et al., 2003](#_ENREF_315)) |
| p.(Trp68Arg) | c.202T>C | Missense | Transmembrane | TNDM | 0 | 0 | ([Mannikko, et al., 2011b](#_ENREF_239)) |
| p.(Trp68Gly) | c.202T>G | Missense | Transmembrane | PNDM | 1 | 0.000003985 | ([O'Connell, et al., 2015](#_ENREF_273)) |
| p.(Trp68Leu) | c.203G>T | Missense | Transmembrane | NDM | 0 | 0 | ([O'Connell, et al., 2015](#_ENREF_273)) |
| p.(Trp68Cys) | c.204G>C | Missense | Transmembrane | PNDM | 0 | 0 | ([Alkorta-Aranburu, et al., 2014](#_ENREF_13)) |
| p.(Thr71Ile) | c.212C>T | Missense | Transmembrane | HI | 1 | 0.000003984 | ([Snider, et al., 2013](#_ENREF_356)) |
| p.(Met77CysfsTer53) | c.228del | Frameshift |  | HI | 0 | 0 | ([Biagiotti, et al., 2007](#_ENREF_35)) |
| p.(Cys81AlafsTer49) | c.240del | Frameshift |  | HI | 0 | 0 | Exeter unpublished |
| p.(Trp83Ter) | c.244_247dup | Frameshift |  | HI | 0 | 0 | ([Henquin, et al., 2011](#_ENREF_151)) |
| p.(Trp91Arg) | c.271T>C | Missense | Transmembrane | HI | 0 | 0 | ([Aguilar-Bryan and Bryan, 1999](#_ENREF_7); [Bennett, et al., 2010](#_ENREF_34); [Sharma, et al., 2000](#_ENREF_340)) |
| p.(Trp91Ter) | c.272G>A | Nonsense |  | HI | 0 | 0 | ([Demirbilek, et al., 2014](#_ENREF_88); [Ocal, et al., 2011](#_ENREF_274); [Park, et al., 2011](#_ENREF_288)) |
| p.(Trp91Ter) | c.273G>A | Nonsense |  | HI | 0 | 0 | ([Park, et al., 2011](#_ENREF_288)) |
| p.(Ala96Thr) | c.286G>A | Missense | Extracellular | HI | 3 | 0.00001196 | ([Craigie, et al., 2018](#_ENREF_72); [Mohnike, et al., 2014](#_ENREF_256)) |
| p.(Gly98Ser) | c.292G>A | Missense | Extracellular | HI | 0 | 0 | ([Snider, et al., 2013](#_ENREF_356)) |
| p.(Asp99Asn) | c.295G>A | Missense | Extracellular | HI | 0 | 0 | ([Martinez, et al., 2016](#_ENREF_243)) |
| p.(Asp99Tyr) | c.295G>T | Missense | Extracellular | HI | 0 | 0 | Paris unpublished |
| p.(Ala101Asp) | c.302C>A | Missense | Extracellular | HI | 7 | 0.00002480 | ([Stanley, et al., 2004](#_ENREF_361); [Suchi, et al., 2006](#_ENREF_365)) |
| p.(Ser103GlnfsTer6) | c.305_306insG | Frameshift |  | HI | 0 | 0 | ([Fan, et al., 2015](#_ENREF_103)) |
| p.(Ser116Pro) | c.346T>C | Missense | Extracellular | HI | 0 | 0 | ([Craigie, et al., 2018](#_ENREF_72); [Suchi, et al., 2006](#_ENREF_365)) |
| p.(Ala120CysfsTer7) | c.356dup | Frameshift |  | HI | 0 | 0 | Exeter unpublished |
| p.(Glu126Lys) | c.376G>A | Missense | Transmembrane Pore-forming | HI | 0 | 0 | ([Demirbilek, et al., 2014](#_ENREF_88)) |
| p.(Glu126Ter) | c.376G>T | Nonsense |  | HI | 0 | 0 | ([Lee, et al., 2015](#_ENREF_219)) |
| p.(Val129Met) | c.385G>A | Missense | Transmembrane Pore-Forming | NDM | 0 | 0 | Exeter unpublished |
| p.(Gly132TyrfsTer10) | c.390_393dup | Frameshift |  | HI | 0 | 0 | Exeter unpublished |
| p.(Gly132Asp) | c.395G>A | Missense | Transmembrane Pore-forming | HI | 0 | 0 | ([Kapoor, et al., 2013](#_ENREF_188)) |
| p.(Gly134Ala) | c.401G>C | Missense | Transmembrane Pore-forming | HI | 0 | 0 | ([Stanley, et al., 2004](#_ENREF_361); [Suchi, et al., 2003](#_ENREF_364)) |
| p.(Arg136AlafsTer5) | c.405dup | Frameshift |  | HI | 5 | 0.00001771 | ([Arya, et al., 2014b](#_ENREF_20); [Fournet, et al., 2001](#_ENREF_117)) |
| p.(Arg136Cys) | c.406C>T | Missense | Extracellular | HI | 3 | 0.00001063 | ([Bellanne-Chantelot, et al., 2010](#_ENREF_31); [Park, et al., 2011](#_ENREF_288)) |
| p.(Arg136Ser) | c.406C>A | Missense | Extracellular | HI | 0 | 0 | ([Mohnike, et al., 2014](#_ENREF_256)) |
| p.(Arg136His) | c.407G>A | Missense | Extracellular | HI | 0 | 0 | ([Sherif, et al., 2013](#_ENREF_343)) |
| p.(Arg136Leu) | c.407G>T | Missense | Extracellular | HI | 1 | 0.000003984 | ([Gong, et al., 2016](#_ENREF_136); [Stanley, et al., 2004](#_ENREF_361); [Suchi, et al., 2006](#_ENREF_365)) |
| p.(Val138Glu) | c.413T>A | Missense | Extracellular | HI | 0 | 0 | ([Ni, et al., 2019](#_ENREF_268)) |
| p.(Leu147Pro) | c.440T>C | Missense | Transmembrane | HI | 1 | 0.000003986 | ([Thomas, et al., 1996a](#_ENREF_386)) |
| p.(Gly156Arg) | c.466G>A | Missense | Transmembrane | HI | 0 | 0 | ([Bushman, et al., 2010](#_ENREF_48); [Pinney, et al., 2008](#_ENREF_299)) |
| p.(Leu157Phe) | c.469C>T | Missense | Transmembrane | HI | 0 | 0 | ([Szymanowski, et al., 2016](#_ENREF_368)) |
| p.(Ala161Thr) | c.481G>A | Missense | Transmembrane | PNDM | 0 | 0 | ([Babiker, et al., 2016](#_ENREF_25)) |
| p.(Ala161Val) | c.482C>T | Missense | Transmembrane | HI | 0 | 0 | ([Kapoor, et al., 2013](#_ENREF_188)) |
| p.(Leu164Phe) | c.490C>T | Missense | Transmembrane | HI | 0 | 0 | ([Boodhansingh, et al., 2019](#_ENREF_40)) |
| p.(Leu164Pro) | c.491T>C | Missense | Transmembrane | PNDM | 0 | 0 | ([Flanagan, et al., 2006](#_ENREF_110); [Hashimoto, et al., 2017](#_ENREF_149); [Rubio-Cabezas, et al., 2012](#_ENREF_319); [Tammaro, et al., 2008](#_ENREF_375)) |
| p.(Cys166TrpfsTer15) | c.491_494dup | Frameshift |  | HI | 0 | 0 | ([Bellanne-Chantelot, et al., 2010](#_ENREF_31)) |
| p.(Cys166Tyr) | c.497G>A | Missense | Transmembrane | DEND | 0 | 0 | ([Bennett, et al., 2015](#_ENREF_33); [Della Manna, et al., 2008](#_ENREF_87); [Flanagan, et al., 2006](#_ENREF_110); [Suzuki, et al., 2007](#_ENREF_367)) |
| p.(Cys166Phe) | c.497G>T | Missense | Transmembrane | DEND | 0 | 0 | ([Gloyn, et al., 2006](#_ENREF_130)) |
| p.(Cys166Trp) | c.498C>G | Missense | Transmembrane | NDM | 0 | 0 | Chicago unpublished |
| p.(Ile167Leu) | c.499A>C | Missense | Cytoplasmic | DEND | 0 | 0 | ([Shimomura, et al., 2007](#_ENREF_348)) |
| p.(Ile167Phe) | c.499A>T | Missense | Cytoplasmic | DEND | 0 | 0 | ([Chang, et al., 2014](#_ENREF_60)) |
| p.(Met169Thr) | c.506T>C | Missense | Cytoplasmic | PNDM | 0 | 0 | Exeter unpublished |
| p.(Lys170Thr) | c.509A>C | Missense | Cytoplasmic | PNDM | 0 | 0 | ([Flanagan, et al., 2006](#_ENREF_110); [Hashimoto, et al., 2017](#_ENREF_149); [Tarasov, et al., 2007](#_ENREF_380)) |
| p.(Lys170Arg) | c.509A>G | Missense | Cytoplasmic | PNDM | 0 | 0 | ([Kim, et al., 2007](#_ENREF_200); [Massa, et al., 2005](#_ENREF_247); [Rubio-Cabezas, et al., 2012](#_ENREF_319)) |
| p.(Lys170Asn) | c.510G>C | Missense | Cytoplasmic | i-DEND  PNDM | 0 | 0 | ([Massa, et al., 2005](#_ENREF_247); [Skupien, et al., 2006](#_ENREF_354); [Taberner, et al., 2016](#_ENREF_369)) |
| p.(Ala172Val) | c.515C>T | Missense | Cytoplasmic | HI | 0 | 0 | ([Snider, et al., 2013](#_ENREF_356)) |
| p.(Ala174Gly) | c.521C>G | Missense | Cytoplasmic | TNDM  PNDM | 0 | 0 | ([Madani, 2019](#_ENREF_231); [Suzuki, et al., 2007](#_ENREF_367)) |
| p.(Ala174Val) | c.521C>T | Missense | Cytoplasmic | HI | 0 | 0 | ([Sogno Valin, et al., 2013](#_ENREF_357)) |
| p.(Arg176His) | c.527G>A | Missense | Cytoplasmic | HI | 1 | 0.000004020 | ([Szymanowski, et al., 2016](#_ENREF_368)) |
| p.(Arg177Trp) | c.529A>T | Missense | Cytoplasmic | HI | 0 | 0 | ([Arya, et al., 2014b](#_ENREF_20)) |
| p.(Ala178LeufsTer11) | c.532del | Frameshift |  | HI | 0 | 0 | Exeter unpublished |
| p.(Glu179Lys) | c.535G>A | Missense | Cytoplasmic | TNDM | 0 | 0 | Exeter unpublished |
| p.(Glu179Ala) | c.536A>C | Missense | Cytoplasmic | TNDM | 0 | 0 | ([Flanagan, et al., 2007](#_ENREF_113)) |
| p.(Ile182Val) | c.544A>G | Missense | Cytoplasmic | TNDM | 0 | 0 | ([Gloyn, et al., 2005](#_ENREF_132); [Koster, et al., 2005](#_ENREF_211)) |
| p.(Ile182Thr) | c.545T>C | Missense | Cytoplasmic | NDM | 0 | 0 | ([Bonnefond, et al., 2014](#_ENREF_39)) |
| p.(Lys185Gln) | c.553A>C | Missense | Cytoplasmic | PNDM | 0 | 0 | ([Clark, et al., 2012](#_ENREF_65); [Shimomura, et al., 2010](#_ENREF_345)) |
| p.(Lys185Thr) | c.554A>C | Missense | Cytoplasmic | PNDM | 0 | 0 | ([Babiker, et al., 2016](#_ENREF_25)) |
| p.(His186Asp) | c.556C>G | Missense | Cytoplasmic | PNDM | 0 | 0 | ([Ahn, et al., 2015](#_ENREF_8)) |
| p.(Ala187Val) | c.560C>T | Missense | Cytoplasmic | HI | 0 | 0 | ([Fournet, et al., 2001](#_ENREF_117); [Park, et al., 2011](#_ENREF_288)) |
| p.(Arg192His) | c.575G>A | Missense | Cytoplasmic | Later-onset diabetes | 3 | 0.00001077 | ([Liu, et al., 2013](#_ENREF_228)) |
| p.(Arg201Ser) | c.601C>A | Missense | Cytoplasmic | PNDM | 0 | 0 | ([Russo, et al., 2011b](#_ENREF_321)) |
| p.(Arg201Gly) | c.601C>G | Missense | Cytoplasmic | PNDM | 0 | 0 | ([Hashimoto, et al., 2017](#_ENREF_149)) |
| p.(Arg201Cys) | c.601C>T | Missense | Cytoplasmic | i-DEND  PNDM | 0 | 0 | ([Abujbara, et al., 2014](#_ENREF_5); [Bennett, et al., 2015](#_ENREF_33); [Carmody, et al., 2014](#_ENREF_54); [Chai-Udom, et al., 2016](#_ENREF_57); [de Wet and Proks, 2015](#_ENREF_82); [Deeb, et al., 2016](#_ENREF_85); [Dupont, et al., 2012](#_ENREF_94); [Edghill, et al., 2004](#_ENREF_96); [Edghill, et al., 2007](#_ENREF_97); [Flanagan, et al., 2006](#_ENREF_110); [Flechtner, et al., 2008](#_ENREF_115); [Globa, et al., 2015](#_ENREF_128); [Gloyn, et al., 2004a](#_ENREF_129); [Gloyn, et al., 2004b](#_ENREF_131); [Gonen, et al., 2012](#_ENREF_135); [Hashimoto, et al., 2017](#_ENREF_149); [Huopio, et al., 2016](#_ENREF_158); [Jahnavi, et al., 2013](#_ENREF_176); [Kumaraguru, et al., 2009](#_ENREF_213); [Letha, et al., 2007](#_ENREF_221); [Lin, et al., 2006a](#_ENREF_223); [Massa, et al., 2005](#_ENREF_247); [Nagano, et al., 2012](#_ENREF_262); [Nyangabyaki-Twesigye, et al., 2015](#_ENREF_272); [Proks, et al., 2004](#_ENREF_306); [Proks, et al., 2005a](#_ENREF_309); [Rica, et al., 2007](#_ENREF_316); [Russo, et al., 2011a](#_ENREF_320); [Russo, et al., 2011b](#_ENREF_321); [Sebastian Ochoa, et al., 2008](#_ENREF_333); [Shah, et al., 2012b](#_ENREF_338); [Shahawy, et al., 2011](#_ENREF_339); [Stoy, et al., 2008](#_ENREF_362); [Suzuki, et al., 2007](#_ENREF_367); [Taberner, et al., 2016](#_ENREF_369); [Vaxillaire, et al., 2004](#_ENREF_397); [Wang, et al., 2012](#_ENREF_404)) |
| p.(Arg201His) | c.602G>A | Missense | Cytoplasmic | i-DEND  PNDM  TNDM | 0 | 0 | ([Al-Mahdi, et al., 2010](#_ENREF_10); [Alkorta-Aranburu, et al., 2014](#_ENREF_13); [Bennett, et al., 2015](#_ENREF_33); [Bremer, et al., 2008](#_ENREF_44); [Cao, et al., 2016](#_ENREF_53); [Carmody, et al., 2014](#_ENREF_54); [Chan and Laffel, 2007](#_ENREF_59); [Codner, et al., 2007](#_ENREF_68); [Colombo, et al., 2005](#_ENREF_69); [Daublin, et al., 2007](#_ENREF_78); [Edghill, et al., 2007](#_ENREF_97); [Feigerlova, et al., 2006](#_ENREF_105); [Flanagan, et al., 2006](#_ENREF_110); [Flechtner, et al., 2008](#_ENREF_115); [Gaal, et al., 2012](#_ENREF_119); [Globa, et al., 2015](#_ENREF_128); [Gloyn, et al., 2006](#_ENREF_130); [Gloyn, et al., 2004b](#_ENREF_131); [Hashimoto, et al., 2017](#_ENREF_149); [Heo, et al., 2013](#_ENREF_153); [Iafusco, et al., 2012](#_ENREF_162); [Ille, et al., 2010](#_ENREF_164); [Ioannou, et al., 2011](#_ENREF_169); [Klupa, et al., 2005](#_ENREF_202); [Klupa, et al., 2010a](#_ENREF_204); [Klupa, et al., 2010b](#_ENREF_205); [Kumaraguru, et al., 2009](#_ENREF_213); [Lauridsen, et al., 2009](#_ENREF_218); [Lin, et al., 2006a](#_ENREF_223); [Proks, et al., 2004](#_ENREF_306); [Proks, et al., 2005a](#_ENREF_309); [Rica, et al., 2007](#_ENREF_316); [Sagen, et al., 2004](#_ENREF_323); [Shah, et al., 2012b](#_ENREF_338); [Shahawy, et al., 2011](#_ENREF_339); [Skupien, et al., 2006](#_ENREF_354); [Stoy, et al., 2008](#_ENREF_362); [Suzuki, et al., 2007](#_ENREF_367); [Taberner, et al., 2016](#_ENREF_369); [Tarasov, et al., 2006](#_ENREF_379); [Thewjitcharoen, et al., 2014](#_ENREF_385); [Tonini, et al., 2006](#_ENREF_391); [Vaxillaire, et al., 2004](#_ENREF_397); [Wagner, et al., 2009](#_ENREF_402); [Wambach, et al., 2010](#_ENREF_403); [Xiao, et al., 2009](#_ENREF_407); [Zhang, et al., 2015a](#_ENREF_419); [Zung, et al., 2004](#_ENREF_423)) |
| p.(Arg201Leu) | c.602G>T | Missense | Cytoplasmic | PNDM | 0 | 0 | ([Codner, et al., 2005](#_ENREF_67)) |
| p.(Arg201Pro) | c.602G>C | Missense | Cytoplasmic | HI | 0 | 0 | ([Ni, et al., 2019](#_ENREF_268)) |
| p.(Asp204Glu) | c.612C>A | Missense | Cytoplasmic | HI | 0 | 0 | ([Mohnike, et al., 2014](#_ENREF_256); [Pinney, et al., 2013](#_ENREF_298)) |
| p.(Arg206Cys) | c.616C>T | Missense | Cytoplasmic | HI | 3 | 0.00001207 | ([Bennett, et al., 2015](#_ENREF_33); [Coventry, et al., 2010](#_ENREF_70); [Salomon-Estebanez, et al., 2016](#_ENREF_328)) |
| p.(Arg206Leu) | c.617G>T | Missense | Cytoplasmic | HI | 0 | 0 | ([Salomon-Estebanez, et al., 2016](#_ENREF_328)) |
| p.(Arg206His) | c.617G>A | Missense | Cytoplasmic | HI  Later-onset diabetes* | 0 | 0 | ([Boodhansingh, et al., 2019](#_ENREF_40))  Odense unpublished  Paris unpublished* |
| p.(Ser208Thr) | c.623G>C | Missense | Cytoplasmic | HI | 0 | 0 | Exeter unpublished |
| p.(Met209Ile) | c.627G>A | Missense | Cytoplasmic | HI | 0 | 0 | ([Arya, et al., 2014b](#_ENREF_20)) |
| p.(Ala213Thr) | c.637G>A | Missense | Cytoplasmic | HI | 2 | 0.000007118 | ([Bellanne-Chantelot, et al., 2010](#_ENREF_31); [Bennett, et al., 2015](#_ENREF_33); [Yorifuji, et al., 2011](#_ENREF_417)) |
| p.(Arg221His) | c.662G>A | Missense | Cytoplasmic | HI | 1 | 0.000003994 | ([Kocaay, et al., 2016](#_ENREF_206)) |
| p.(Thr223Pro) | c.667A>C | Missense | Cytoplasmic | HI | 0 | 0 | ([Bellanne-Chantelot, et al., 2010](#_ENREF_31)) |
| p.(Ser225_Pro232delinsThr) | c.674_694del | Missense | Cytoplasmic | i-DEND | 0 | 0 | ([Battaglia, et al., 2012](#_ENREF_30); [Lin, et al., 2013](#_ENREF_226)) |
| p.(Glu227Lys) | c.679G>A | Missense | Cytoplasmic | TNDM Diabetes | 1 | 0.000003988 | ([Abbasi, et al., 2012](#_ENREF_1); [Bonnefond, et al., 2012](#_ENREF_38); [Edghill, et al., 2007](#_ENREF_97); [Girard, et al., 2006](#_ENREF_123); [Hashimoto, et al., 2017](#_ENREF_149); [Kochar and Kulkarni, 2010](#_ENREF_207); [Landau, et al., 2007](#_ENREF_216); [Martins, et al., 2015](#_ENREF_244); [Rica, et al., 2007](#_ENREF_316); [Stoy, et al., 2008](#_ENREF_362); [Taberner, et al., 2016](#_ENREF_369)) |
| p.(Glu227Leu) | c.679_680delinsCT | Missense | Cytoplasmic | TNDM | 0 | 0 | ([D'Amato, et al., 2008](#_ENREF_73)) |
| p.(Gly228Ala) | c.683G>C | Missense | Cytoplasmic | NDM | 0 | 0 | ([Bonnefond, et al., 2014](#_ENREF_39)) |
| p.(Glu229Lys) | c.685G>A | Missense | Cytoplasmic | TNDM | 0 | 0 | ([Bennett, et al., 2015](#_ENREF_33); [Cao, et al., 2016](#_ENREF_53); [Edghill, et al., 2007](#_ENREF_97); [Flanagan, et al., 2007](#_ENREF_113); [Gaal, et al., 2012](#_ENREF_119); [Girard, et al., 2006](#_ENREF_123); [Globa, et al., 2015](#_ENREF_128); [Iafusco, et al., 2012](#_ENREF_162); [Klupa, et al., 2010b](#_ENREF_205); [Orio Hernandez, et al., 2008](#_ENREF_279); [Rica, et al., 2007](#_ENREF_316)) |
| p.(Leu233Phe) | c.697C>T | Missense | Cytoplasmic | PNDM | 0 | 0 | ([Joshi and Phatarpekar, 2011](#_ENREF_185)) |
| p.(Gln235Glu) | c.703C>G | Missense | Cytoplasmic | HI | 0 | 0 | ([Sang, et al., 2014a](#_ENREF_331)) |
| p.(Val252Met) | c.754G>A | Missense | Cytoplasmic | TNDM | 0 | 0 | ([Flanagan, et al., 2010](#_ENREF_112)) |
| p.(Val252Leu) | c.754G>C | Missense | Cytoplasmic | PNDM | 0 | 0 | ([Jesic, et al., 2011](#_ENREF_179)) |
| p.(Val252Ala) | c.755T>C | Missense | Cytoplasmic | TNDM  PNDM | 0 | 0 | ([Girard, et al., 2006](#_ENREF_123); [Ooi and Wu, 2012](#_ENREF_278); [Rica, et al., 2007](#_ENREF_316)) |
| p.(Val252Gly) | c.755T>G | Missense | Cytoplasmic | PNDM | 0 | 0 | ([Flanagan, et al., 2010](#_ENREF_112)) |
| p.(Ala253Asp) | c.758C>A | Missense | Cytoplasmic | HI | 0 | 0 | ([Korula, et al., 2018](#_ENREF_209)) |
| p.(Pro254Leu) | c.761C>T | Missense | Cytoplasmic | HI | 1 | 0.000003979 | ([Martinez, et al., 2016](#_ENREF_243); [Tornovsky, et al., 2004](#_ENREF_392)) |
| p.(Pro254Gln) | c.761C>A | Missense | Cytoplasmic | TNDM | 0 | 0 | ([Gole, et al., 2018](#_ENREF_134)) |
| p.(Tyr258Ter) | c.774C>A | Nonsense |  | HI | 0 | 0 | Exeter unpublished |
| p.(His259MetfsTer61) | c.775del | Missense | Cytoplasmic | HI | 0 | 0 | Exeter unpublished |
| p.(His259Arg) | c.776A>G | Missense | Cytoplasmic | HI | 12 | 0.00004245 | ([Bennett, et al., 2010](#_ENREF_34); [Kalish, et al., 2016](#_ENREF_186); [Marthinet, et al., 2005](#_ENREF_241)) |
| p.(His259Gln) | c.777T>G | Missense | Cytoplasmic | HI | 0 | 0 | ([Bellanne-Chantelot, et al., 2010](#_ENREF_31)) |
| p.(Pro266Leu) | c.797C>T | Missense | Cytoplasmic | HI | 0 | 0 | ([Stanley, et al., 2004](#_ENREF_361); [Suchi, et al., 2003](#_ENREF_364)) |
| p.(Leu270Met) | c.808C>A | Missense | Cytoplasmic | HI | 7 | 0.00002785 | ([Guven, et al., 2016](#_ENREF_142)) |
| p.(Pro272SerfsTer37) | c.813delinsTT | Frameshift |  | HI | 1 | 0.000004063 | ([Biagiotti, et al., 2007](#_ENREF_35)) |
| p.(Pro272LeufsTer49) | c.813delinsTCT | Frameshift |  | HI | 0 | 0 | ([Fournet, et al., 2001](#_ENREF_117)) |
| p.(Gln279Ter) | c.835C>T | Nonsense |  | HI | 0 | 0 | Exeter unpublished |
| p.(Glu282Lys) | c.844G>A | Missense | Cytoplasmic | HI | 7 | 0.00002476 | ([Christesen, et al., 2007](#_ENREF_62); [Hardy, et al., 2007](#_ENREF_146); [Kalish, et al., 2016](#_ENREF_186); [Taneja, et al., 2009 Mohnike, 2014 #1086](#_ENREF_377)) |
| p.(Ile284del) | c.850_852del | In-Frame Deletion | Cytoplasmic | HI | 0 | 0 | ([Arya, et al., 2014b](#_ENREF_20); [Kapoor, et al., 2013](#_ENREF_188); [Snider, et al., 2013](#_ENREF_356)) |
| p.(Val285Ile) | c.853G>A | Missense | Cytoplasmic | TNDM | 6 | 0.00002122 | ([Jahnavi, et al., 2013](#_ENREF_176)) |
| p.(Glu288Lys) | c.862G>A | Missense | Cytoplasmic | HI | 0 | 0 | ([Guven, et al., 2016](#_ENREF_142)) |
| p.(Gln289Val) | c.866G>T | Missense | Cytoplasmic | HI | 0 | 0 | ([Mohnike, et al., 2014](#_ENREF_256)) |
| p.(Gln289Ala) | c.866G>C | Missense | Cytoplasmic | HI | 0 | 0 | Chicago unpublished |
| p.(Val290Met) | c.868G>A | Missense | Cytoplasmic | HI | 5 | 0.00001989 | ([Loechner, et al., 2011](#_ENREF_229); [Maiorana, et al., 2014](#_ENREF_234)) |
| p.(Glu292Lys) | c.874G>A | Missense | Cytoplasmic | HI | 0 | 0 | ([Arya, et al., 2014b](#_ENREF_20)) |
| p.(Glu292Gly) | c.875A>G | Missense | Cytoplasmic | PNDM | 0 | 0 | ([Girard, et al., 2006](#_ENREF_123); [Rica, et al., 2007](#_ENREF_316)) |
| p.(Thr293Asn) | c.878C>A | Missense | Cytoplasmic | i-DEND  DEND | 0 | 0 | ([Hashimoto, et al., 2017](#_ENREF_149); [Shimomura, et al., 2009](#_ENREF_346)) |
| p.(Thr294Met) | c.881C>T | Missense | Cytoplasmic | HI | 5 | 0.00001989 | ([Arya, et al., 2014b](#_ENREF_20); [Bellanne-Chantelot, et al., 2010](#_ENREF_31); [Gong, et al., 2016](#_ENREF_136); [Ilamaran, et al., 2010](#_ENREF_163); [Salomon-Estebanez, et al., 2016](#_ENREF_328); [Shimomura, et al., 2009](#_ENREF_346)) |
| p.(Gly295Ser) | c.883G>A | Missense | Cytoplasmic | HI | 0 | 0 | Paris unpublished |
| p.(Ile296Leu) | c.886A>C | Missense | Cytoplasmic | DEND | 0 | 0 | ([Gloyn, et al., 2004b](#_ENREF_131); [Khurana, et al., 2011](#_ENREF_197); [Koster, et al., 2005](#_ENREF_211); [Lin, et al., 2006a](#_ENREF_223); [Proks, et al., 2005b](#_ENREF_311); [Tammaro and Ashcroft, 2009](#_ENREF_374)) |
| p.(Thr298del) | c.892_894del | In-Frame deletions | Cytoplasmic | HI | 0 | 0 | ([Boodhansingh, et al., 2019](#_ENREF_40)) |
| p.(Gln299Arg) | c.896A>G | Missense | Cytoplasmic | HI | 0 | 0 | ([Han, et al., 2016](#_ENREF_144)) |
| p.(Gln299His) | c.897G>C | Missense | Cytoplasmic | HI | 0 | 0 | ([Kocaay, et al., 2016](#_ENREF_206)) |
| p.(Arg301Gly) | c.901C>G | Missense | Cytoplasmic | HI | 0 | 0 | ([Lin, et al., 2008](#_ENREF_225); [Mohnike, et al., 2014](#_ENREF_256)) |
| p.(Arg301His) | c.902G>A | Missense | Cytoplasmic | HI | 3 | 0.00001193 | ([Bellanne-Chantelot, et al., 2010](#_ENREF_31); [Craigie, et al., 2018](#_ENREF_72); [Lin, et al., 2008](#_ENREF_225); [Stanley, et al., 2004](#_ENREF_361); [Suchi, et al., 2006](#_ENREF_365)) |
| p.(Arg301Pro) | c.902G>C | Missense | Cytoplasmic | HI | 0 | 0 | ([Lin, et al., 2008](#_ENREF_225); [Qubbaj, et al., 2011](#_ENREF_313)) |
| p.(Leu310ProfsTer12) | c.928dup | Frameshift |  | HI | 0 | 0 | ([Snider, et al., 2013](#_ENREF_356)) |
| p.(Gly312Cys) | c.934G>T | Missense | Cytoplasmic | HI | 0 | 0 | ([Arya, et al., 2014b](#_ENREF_20)) |
| p.(Phe315Ile) | c.943T>A | Missense | Cytoplasmic | HI | 0 | 0 | ([Unal, et al., 2016](#_ENREF_393)) |
| p.(Glu322Lys) | c.964G>A | Missense | Cytoplasmic | PNDM | 0 | 0 | ([Flechtner, et al., 2008](#_ENREF_115); [Nieves-Rivera and Gonzalez-Pijem, 2011](#_ENREF_270); [Polak and Cave, 2007](#_ENREF_300); [Shah, et al., 2012b](#_ENREF_338); [Stoy, et al., 2008](#_ENREF_362); [Tarasov, et al., 2007](#_ENREF_380); [Vaxillaire, et al., 2004](#_ENREF_397)) |
| p.(Glu322Ala) | c.965A>C | Missense | Cytoplasmic | TNDM | 0 | 0 | ([Siklar, et al., 2011](#_ENREF_351)) |
| p.(Gly324Arg) | c.970G>A | Missense | Cytoplasmic | TNDM | 3 | 0.00001061 | ([Bennett, et al., 2015](#_ENREF_33); [Vedovato, et al., 2016](#_ENREF_398)) |
| p.(Val328Met) | c.982G>A | Missense | Cytoplasmic | TNDM | 0 | 0 | Exeter unpublished |
| p.(Tyr330Asn) | c.988T>A | Missense | Cytoplasmic | TNDM | 0 | 0 | Exeter unpublished |
| p.(Tyr330His) | c.988T>C | Missense | Cytoplasmic | Diabetes | 0 | 0 | Chicago unpublished |
| p.(Tyr330Ser) | c.989A>C | Missense | Cytoplasmic | PNDM | 0 | 0 | ([Pearson, et al., 2006](#_ENREF_291)) |
| p.(Tyr330Ser) | c.989_990delinsCT | Missense | Cytoplasmic | PNDM | 0 | 0 | ([Flanagan, et al., 2006](#_ENREF_110)) |
| p.(Tyr330Cys) | c.989A>G | Missense | Cytoplasmic | PNDM | 0 | 0 | ([Bruederle, et al., 2011](#_ENREF_46); [Flechtner, et al., 2008](#_ENREF_115); [Polak and Cave, 2007](#_ENREF_300); [Shah, et al., 2012b](#_ENREF_338)) |
| p.(Ser331Pro) | c.991T>C | Missense | Cytoplasmic | PNDM | 0 | 0 | Exeter unpublished |
| p.(Phe333Ile) | c.997T>A | Missense | Cytoplasmic | PNDM | 0 | 0 | ([Bruederle, et al., 2011](#_ENREF_46); [Mankouri, et al., 2006](#_ENREF_236); [Sagen, et al., 2004](#_ENREF_323); [Tammaro and Ashcroft, 2007](#_ENREF_373); [Tammaro and Ashcroft, 2009](#_ENREF_374); [Tammaro, et al., 2005](#_ENREF_376)) |
| p.(Phe333Ser) | c.998T>C | Missense | Cytoplasmic | HI | 0 | 0 | ([Bellanne-Chantelot, et al., 2010](#_ENREF_31); [Bendix, et al., 2018](#_ENREF_32)) |
| p.(Phe333Leu) | c.999T>G | Missense | Cytoplasmic | PNDM | 0 | 0 | ([Philla, et al., 2013](#_ENREF_296)) |
| p.(Gly334Ser) | c.1000G>A | Missense | Cytoplasmic | PNDM | 0 | 0 | Exeter unpublished |
| p.(Gly334Arg) | c.1000G>C | Missense | Cytoplasmic | PNDM | 0 | 0 | Exeter unpublished |
| p.(Gly334Cys) | c.1000G>T | Missense | Cytoplasmic | PNDM | 0 | 0 | ([Babiker, et al., 2016](#_ENREF_25); [Myngheer, et al., 2014](#_ENREF_261)) |
| p.(Gly334Asp) | c.1001G>A | Missense | Cytoplasmic | DEND | 0 | 0 | ([de Wet and Proks, 2015](#_ENREF_82); [Drain, et al., 1998](#_ENREF_91); [Masia, et al., 2007b](#_ENREF_246); [Proks, et al., 2010](#_ENREF_308)) |
| p.(Gly334Val) | c.1001G>T | Missense | ytoplasmic | PNDM | 0 | 0 | ([Flanagan, et al., 2014](#_ENREF_108); [Lau, et al., 2015](#_ENREF_217)) |
| p.(Val339Gly) | c.1016T>G | Missense | Cytoplasmic | HI | 2 | 0.000007953 | ([Snider, et al., 2013](#_ENREF_356)) |
| p.(Pro340His) | c.1019C>A | Missense | Cytoplasmic | HI | 0 | 0 | ([Mohnike, et al., 2014](#_ENREF_256)) |
| p.(Cys344Ter) | c.1032del | Frameshift |  | HI | 0 | 0 | ([Ohkubo, et al., 2005](#_ENREF_275)) |
| p.(Asp352His) | c.1054G>C | Missense | Cytoplasmic | TNDM | 0 | 0 | ([Siklar, et al., 2011](#_ENREF_351)) |
| p.(Arg365Cys) | c.1093C>T | Missense | Cytoplasmic | HI | 8 | 0.00002833 | ([Snider, et al., 2013](#_ENREF_356)) |
| p.(Gly366GlufsTer128) | c.1097_1109delinsAG | Frameshift |  | HI | 0 | 0 | ([Mohnike, et al., 2014](#_ENREF_256)) |
| p.(Ter391ArgextTer93) | c.1171T>C | Stop loss |  | HI | 0 | 0 | ([Arya, et al., 2014b](#_ENREF_20)) |

**Supp Table S2: Variants of uncertain clinical significance identified in *KCNJ11* (NM_000525.3)*.*** NDM = Neonatal Diabetes. PNDM = Permanent Neonatal Diabetes Mellitus. TNDM = Transient Neonatal Diabetes Mellitus. HI= Hyperinsulinism. * in the Phenotype column highlights a new phenotype; the * in the Reference column indicates which laboratory has identified the variant in a patients with the new phenotype.

| **Protein Change** | **Nucleotide Position** | **Mutation Type** | **Protein domain (UniProt)** | **Phenotype** | **GnomAD AC** | **GnomAD MAF** | **Reference** |
| --- | --- | --- | --- | --- | --- | --- | --- |
| p.(Arg4His) | c.11G>A | Missense | Cytoplasmic | HI | 2 | 0.000008066 | Exeter unpublished |
| p.(Arg29His) | c.86G>A | Missense | Cytoplasmic | Unaffected | 3 | 0.00001063 | ([Coventry, et al., 2010](#_ENREF_70)) |
| p.(Cys42Tyr) | c.125G>A | Missense | Cytoplasmic | Diabetes | 0 | 0 | Paris unpublished |
| p.(Ala45Ser) | c.133G>T | Missense | Cytoplasmic | Diabetes | 0 | 0 | Exeter unpublished |
| p.(Arg50Trp) | c.148C>T | Missense | Cytoplasmic | Diabetes  HI | 0 | 0 | Paris unpublished,  Exeter unpublished |
| p.(Gln52Pro) | c.155A>C | Missense | Cytoplasmic | NDM | 0 | 0 | Exeter unpublished |
| p.(Asp58Val) | c.173A>T | Missense | Cytoplasmic | HI | 0 | 0 | Paris unpublished |
| p.(Phe60Ser) | c.179T>C | Missense | Cytoplasmic | HI | 0 | 0 | Chicago unpublished |
| p.(Val64Leu) | c.190G>C | Missense | Cytoplasmic | NDM | 0 | 0 | ([Mannikko, et al., 2010](#_ENREF_238)) |
| p.(Leu84Arg) | c.251T>G | Missense | Transmembrane | HI | 0 | 0 | Exeter unpublished |
| p.(Ala96Val) | c.287C>T | Missense | Extracellular | HI | 0 | 0 | Exeter unpublished |
| p.(His97Gln) | N/A | Missense | Extracellular | Unaffected | 0 | 0 | ([Coventry, et al., 2010](#_ENREF_70)) |
| p.(His97Tyr) | c.289C>T | Missense | Extracellular | Diabetes | 0 | 0 | Exeter unpublished |
| p.(Glu104Gln) | c.310G>C | Missense | Extracellular | Unaffected | 0 | 0 | ([Coventry, et al., 2010](#_ENREF_70)) |
| p.(Ile114Thr) | c.341T>C | Missense | Extracellular | Diabetes | 0 | 0 | Paris unpublished |
| p.(His115Leu) | c.344A>T | Missense | Extracellular | HI | 0 | 0 | Paris unpublished |
| p.(Phe117_Ser118del) | c.348_353del | In-Frame deletion | Transmembrane | Diabetes | 0 | 0 | ([Liu, et al., 2013](#_ENREF_228)) |
| p.(Phe117del) | c.350_352del | In-Frame deletion | Transmembrane | HI | 3 | 0.00001194 | ([Mohnike, et al., 2014](#_ENREF_256); [Yorifuji, et al., 2013](#_ENREF_416)) |
| p.(Ser118Leu) | c.353C>T | Missense | Transmembrane | Diabetes | 6 | 0.00002389 | Paris unpublished, Chicago unpublished |
| p.(Phe121Ser) | c.362T>C | Missense | Transmembrane | HI | 0 | 0 | Paris unpublished |
| p.(Gln128Arg) | c.383A>G | Missense | Transmembrane | HI | 0 | 0 | ([Bellanne-Chantelot, et al., 2010](#_ENREF_31)) |
| p.(Ile131dup) | c.391_393dup | In-Frame duplication | Transmembrane Pore-Forming | HI | 0 | 0 | Paris unpublished |
| p.(Ile131Val) | c.391A>G | Missense | Transmembrane Pore-Forming | HI | 0 | 0 | Exeter unpublished |
| p.(Ile131Thr) | c.392T>C | Missense | Transmembrane Pore-Forming | Diabetes | 0 | 0 | ([Ang, et al., 2016](#_ENREF_14)) |
| p.(Thr139Pro) | c.415A>C | Missense | Extracellular | HI | 0 | 0 | Paris unpublished |
| p.(Glu140Lys) | c.418G>A | Missense | Extracellular | HI | 0 | 0 | Paris unpublished |
| p.(Cys142Tyr) | c.425G>A | Missense | Extracellular | HI | 0 | 0 | Exeter unpublished |
| p.(Gln152His) | c.456G>T | Missense | Transmembrane | Unaffected | 1 | 0.000003991 | ([Coventry, et al., 2010](#_ENREF_70)) |
| p.(Val155Leu) | c.463G>T | Missense | Transmembrane | HI | 0 | 0 | Exeter unpublished |
| p.(Val155Met) | c.463G>A | Missense | Transmembrane | Diabetes | 3 | 0.00001199 | Chicago unpublished  Paris unpublished |
| p.(Leu157Val) | c.469C>G | Missense | Transmembrane | HI | 0 | 0 | Exeter unpublished |
| p.(Asn160Lys) | c.480C>G | Missense | Transmembrane | HI | 0 | 0 | Paris unpublished |
| p.(Ile167Val) | c.499A>G | Missense | Cytoplasmic | HI | 0 | 0 | Paris unpublished |
| p.(Thr171Asn) | c.512C>A | Missense | Cytoplasmic | HI | 0 | 0 | Exeter unpublished |
| p.(Arg176Cys) | c.526C>T | Missense | Cytoplasmic | Diabetes Unaffected | 11 | 0.00003928 | ([Coventry, et al., 2010](#_ENREF_70); [Edghill, et al., 2004](#_ENREF_96)) |
| p.(Thr180Ile) | c.539C>T | Missense | Cytoplasmic | HI | 0 | 0 | Paris unpublished |
| p.(Val202Gly) | c.605T>G | Missense | Cytoplasmic | Unaffected | 0 | 0 | ([Coventry, et al., 2010](#_ENREF_70)) |
| p.(Ser208Asn) | c.623G>A | Missense | Cytoplasmic | Diabetes | 0 | 0 | Paris unpublished |
| p.(Met217Val) | c.649A>G | Missense | Cytoplasmic | Unaffected | 1 | 0.000003997 | ([Coventry, et al., 2010](#_ENREF_70)) |
| p.(Lys222Gln) | c.664A>C | Missense | Cytoplasmic | HI | 3 | 0.00001064 | Exeter unpublished |
| p.(Val231Leu) | c.691G>C | Missense | Cytoplasmic | NDM | 0 | 0 | ([Bennett, et al., 2015](#_ENREF_33)) |
| p.(Ser265Ile) | c.794G>T | Missense | Cytoplasmic | HI | 1 | 0.000003978 | Exeter unpublished |
| p.(Tyr268His) | c.802T>C | Missense | Cytoplasmic | HI | 0 | 0 | Exeter unpublished |
| p.(Asp274His) | c.820G>C | Missense | Cytoplasmic | HI | 0 | 0 | Exeter unpublished |
| p.(Leu287Pro) | c.860T>C | Missense | Cytoplasmic | HI | 0 | 0 | Paris unpublished |
| p.(Thr297Asn) | c.890C>A | Missense | Cytoplasmic | NDM | 0 | 0 | Exeter unpublished |
| p.(Ala300Asp) | c.899C>A | Missense | Cytoplasmic | HI | 0 | 0 | Paris unpublished |
| p.(Arg301Cys) | c.901C>T | Missense | Cytoplasmic | HI | 2 | 0.000007071 | ([Bellanne-Chantelot, et al., 2010](#_ENREF_31); [John, et al., 2001](#_ENREF_182); [Lin, et al., 2008](#_ENREF_225)) |
| p.(Leu310Pro) | c.929T>C | Missense | Cytoplasmic | HI | 0 | 0 | Exeter unpublished |
| p.(Gly312Ser) | c.934G>A | Missense | Cytoplasmic | Unaffected | 7 | 0.00002475 | ([Coventry, et al., 2010](#_ENREF_70)) |
| p.(Arg314His) | c.941G>A | Missense | Cytoplasmic | Unaffected | 12 | 0.00004242 | ([Coventry, et al., 2010](#_ENREF_70)) |
| p.(Ile318Val) | c.952A>G | Missense | Cytoplasmic | Diabetes | 3 | 0.00001061 | Paris unpublished |
| p.(Arg325Ser) | c.973C>A | Missense | Cytoplasmic | HI | 4 | 0.00001591 | Chicago unpublished |
| p.(Arg325His) | c.974G>A | Missense | Cytoplasmic | HI | 4 | 0.00001591 | Exeter unpublished |
| p.(Thr336Ala) | c.1006A>G | Missense | Cytoplasmic | Diabetes | 0 | 0 | Exeter unpublished |
| p.(Val339Leu) | c.1015G>C | Missense | Cytoplasmic | HI | 0 | 0 | ([Thomas, et al., 1996a](#_ENREF_386)) |
| p.(Val339=) | c.1017G>T | Synonymous | Cytoplasmic | HI | 10 | 0.00003535 | ([Faletra, et al., 2013a](#_ENREF_101)) |
| p.(Leu343Val) | c.1027C>G | Missense | Cytoplasmic | NDM | 0 | 0 | Exeter unpublished |
| p.(Arg347His) | c.1040G>A | Missense | Cytoplasmic | Unaffected | 6 | 0.00002387 | ([Coventry, et al., 2010](#_ENREF_70); [Kapoor, et al., 2013](#_ENREF_188)) |
| p.(Ser354Gly) | c.1060A>G | Missense | Cytoplasmic | Unaffected | 9 | 0.00003183 | ([Coventry, et al., 2010](#_ENREF_70)) |
| p.(Leu355Pro) | c.1064T>C | Missense | Cytoplasmic | Diabetes | 0 | 0 | ([Sakura, et al., 1996](#_ENREF_327)) |
| p.(Leu361Phe) | c.1081C>T | Missense | Cytoplasmic | Unaffected | 1 | 0.000003982 | ([Coventry, et al., 2010](#_ENREF_70)) |
| p.(Ala362Thr) | c.1084G>A | Missense | Cytoplasmic | Diabetes | 6 | 0.00002390 | ([Mohan, et al., 2018](#_ENREF_255)) |
| p.(Arg365His) | c.1094G>A | Missense | Cytoplasmic | TNDM | 8 | 0.00003189 | ([Flanagan, et al., 2007](#_ENREF_113); [Stanik, et al., 2008](#_ENREF_360)) |
| p.(Gly366Trp) | c.1096G>T | Missense | Cytoplasmic | Diabetes | 0 | 0 | ([Sang, et al., 2014b](#_ENREF_332)) |
| p.(Arg369Ser) | c.1105C>A | Missense | Cytoplasmic | Unaffected | 10 | 0.00003988 | Paris unpublished |
| p.(Arg369His) | c.1106G>A | Missense | Cytoplasmic | Diabetes | 1 | 0.000003989 | Exeter unpublished |
| p.(Arg369Leu) | c.1106G>T | Missense | Cytoplasmic | HI | 1 | 0.000003989 | Chicago unpublished |
| p.(Arg371His) | c.1112G>A | Missense | Cytoplasmic | Unaffected | 4 | 0.00001418 | ([Coventry, et al., 2010](#_ENREF_70); [Jeron, et al., 2004](#_ENREF_178)) |
| p.(Pro374Arg) | c.1121C>G | Missense | Cytoplasmic | Unaffected | 2 | 0.00006373 | ([Coventry, et al., 2010](#_ENREF_70)) |
| p.(Ala376Ser) | c.1126G>T | Missense | Cytoplasmic | HI | 0 | 0 | Paris unpublished |
| p.(Pro380_Lys381dup) | c.1138_1143dup | In-Frame duplication | Cytoplasmic | Diabetes | 20 | 0.00007098 | Paris unpublished |

**Supp Table S3: Benign variants identified in *KCNJ11* (NM_000525.3).**

| **Protein Change** | **Nucleotide Position** | **GnomAD AC** | **GnomAD MAF** | **Reference** |
| --- | --- | --- | --- | --- |
| p.? | c.-745G>A | 0 | 0 | ([Snider, et al., 2013](#_ENREF_356)) |
| p.? | c.-645G>A | 13494 | 0.4416 | ([Snider, et al., 2013](#_ENREF_356)) |
| p.? | c.-614del | 0 | 0 | ([Snider, et al., 2013](#_ENREF_356)) |
| p.? | c.-507del | 13625 | 0.4437 | ([Snider, et al., 2013](#_ENREF_356)) |
| p.(Pro9=) | c.27C>T | 40 | 0.0001426 | Exeter unpublished |
| p.(Glu10Lys) | c.28G>A | 1 | 0.000004013 | ([Inoue, et al., 1997](#_ENREF_166)) |
| p.(Val13Met) | c.37G>A | 94 | 0.0003345 | ([Coventry, et al., 2010](#_ENREF_70); [Snider, et al., 2013](#_ENREF_356)) |
| p.(Lys23Glu) | c.67A>G | 182578 | 0.6477 | ([Florez, et al., 2007](#_ENREF_116); [Inoue, et al., 1997](#_ENREF_166); [Sakura, et al., 1996](#_ENREF_327); [Someya, et al., 2000](#_ENREF_358)) |
| p.(Arg31Trp) | c.91C>T | 31 | 0.0001098 | ([Coventry, et al., 2010](#_ENREF_70)) |
| p.(Val36=) | c.108G>A | 422 | 0.001494 | ([Snider, et al., 2013](#_ENREF_356)) |
| p.(Ser103=) | c.309C>T | 179 | 0.0006342 | ([Snider, et al., 2013](#_ENREF_356)) |
| p.(Ile148Ser) | c.443T>G | 0 | 0 | ([Ohkubo, et al., 2005](#_ENREF_275)) |
| p.(Ile189=) | c.567C>T | 10 | 0.00003587 | Chicago unpublished |
| p.(Ala190=) | c.570C>T | 71448 | 0.2572 | ([Inoue, et al., 1997](#_ENREF_166); [Sakura, et al., 1996](#_ENREF_327); [Someya, et al., 2000](#_ENREF_358)) |
| p.(Arg195His) | c.584G>A | 109 | 0.0003912 | ([Coventry, et al., 2010](#_ENREF_70); [Russo, et al., 2011b](#_ENREF_321); [Salomon-Estebanez, et al., 2016](#_ENREF_328); [Snider, et al., 2013](#_ENREF_356)) |
| p.(Pro226=) | c.678C>T | 99 | 0.0003510 | ([Snider, et al., 2013](#_ENREF_356)) |
| p.(Leu267Val) | c.799C>G | 0 | 0 | ([Jeron, et al., 2004](#_ENREF_178)) |
| p.(Leu267=) | c.801C>G | 4685 | 0.01657 | ([Gloyn, et al., 2004b](#_ENREF_131); [Inoue, et al., 1997](#_ENREF_166); [Jeron, et al., 2004](#_ENREF_178)) |
| p.(Leu270Val) | c.808C>G | 11500 | 0.04068 | ([Gloyn, et al., 2004b](#_ENREF_131); [Inoue, et al., 1996](#_ENREF_167); [Jeron, et al., 2004](#_ENREF_178); [Sakura, et al., 1996](#_ENREF_327)) |
| p.(Ala271=) | c.813A>T | 1 | 0.000003979 | ([Biagiotti, et al., 2007](#_ENREF_35)) |
| p.(Ser273=) | c.819C>T | 17 | 0.00006014 | ([Snider, et al., 2013](#_ENREF_356)) |
| p.(Leu281=) | c.843C>T | 110 | 0.0003890 | ([Fan, et al., 2015](#_ENREF_103); [Snider, et al., 2013](#_ENREF_356)) |
| p.(Ile284=) | c.852C>A | 100 | 0.0003536 | ([Tanizawa, et al., 2000](#_ENREF_378)) |
| p.(Thr294=) | c.882G>A | 10 | 0.00003536 | Exeter unpublished |
| p.(Arg301=) | c.903C>T | 13 | 0.00004596 | Exeter unpublished |
| p.(Val337Ile) | c.1009G>A | 182457 | 0.6453 | ([Gloyn, et al., 2004b](#_ENREF_131); [Inoue, et al., 1997](#_ENREF_166); [Jeron, et al., 2004](#_ENREF_178); [Sakura, et al., 1996](#_ENREF_327); [Someya, et al., 2000](#_ENREF_358)) |
| p.(Ser363=) | c.1089A>G | 459 | 0.001625 | ([Sakura, et al., 1996](#_ENREF_327)) |
| p.(Arg365=) | c.1095C>T | 139 | 0.0004924 | ([Snider, et al., 2013](#_ENREF_356)) |
| p.(Lys381=) | c.1143G>A | 3484 | 0.01237 | ([Biagiotti, et al., 2007](#_ENREF_35); [Fernandez-Marmiesse, et al., 2006](#_ENREF_106); [Gloyn, et al., 2004b](#_ENREF_131); [Jeron, et al., 2004](#_ENREF_178)) |
| p.(Ser385Cys) | c.1154C>G | 1400 | 0.004973 | ([Coventry, et al., 2010](#_ENREF_70); [Gloyn, et al., 2004b](#_ENREF_131); [Sakura, et al., 1996](#_ENREF_327)) |
| p.? | c.*62G>A | 22255 | 0.7111 | ([Jeron, et al., 2004](#_ENREF_178)) |
| p.? | c.*215C>T | 13878 | 0.4439 | ([Snider, et al., 2013](#_ENREF_356)) |
| p.? | c.*441T>C | 13928 | 0.4449 | ([Snider, et al., 2013](#_ENREF_356)) |
| p.? | c.*1813C>T | 873 | 0.02782 | ([Snider, et al., 2013](#_ENREF_356)) |
| p.? | c.*1834A>G | 13744 | 0.4396 | ([Snider, et al., 2013](#_ENREF_356)) |
| p.? | c.*1859G>C | 15412 | 0.4928 | ([Snider, et al., 2013](#_ENREF_356)) |
| p.? | c.*1873del | 1223 | 0.03906 | ([Snider, et al., 2013](#_ENREF_356)) |
| p.? | c.*1878T>C | 13694 | 0.4380 | ([Snider, et al., 2013](#_ENREF_356)) |
| p.? | c.*2516C>G | 13638 | 0.4351 | ([Snider, et al., 2013](#_ENREF_356)) |

**Supp Table S4: Pathogenic variants identified in *ABCC8* (NM_001287174.1).** NDM = Neonatal Diabetes. PNDM = Permanent Neonatal Diabetes Mellitus. TNDM = Transient Neonatal Diabetes Mellitus. DEND = Developmental Delay and Neonatal Diabetes Syndrome. i-DEND = Intermediate DEND syndrome. HI = Hyperinsulinism. NBD = Nucleotide Binding Domain. Ter = termination codon. * in the Phenotype column highlights a new phenotype; the * in the Reference column indicates which laboratory has identified the variant in a patients with the new phenotype.

| **Protein Change** | **NucleotidePosition** | **Position** | **Mutation Type** | **Protein domain (UniProt)** | **Phenotype** | **GnomAD AC** | **GnomAD MAF** | **Reference** |
| --- | --- | --- | --- | --- | --- | --- | --- | --- |
| p.? | c.-64C>G | Promoter | Upstream substitution |  | HI | 0 | 0 | ([Tornovsky, et al., 2004](#_ENREF_392)) |
| p.? | c.(?-1)_(1011+1_1012-1)del | Exons  1-6 | Deletion |  | HI | 0 | 0 | Exeter unpublished |
| p.? | c.(?-1)_(1176+1_1177-1)del | Exons  1-7 | Deletion |  | HI | 0 | 0 | ([Arya, et al., 2014b](#_ENREF_20); [Flanagan, et al., 2012](#_ENREF_107)) |
| p.? | c.(?-1)_(2697+1_2698-1)del | Exons  1-22 | Deletion |  | HI | 0 | 0 | ([Adi, et al., 2015](#_ENREF_6); [Al-Agha and Ahmad, 2013](#_ENREF_9); [Bitner-Glindzicz, et al., 2000](#_ENREF_36)) |
| p.? | c.(?-1)_(4749+?)del | Exons  1-39 | Deletion |  | HI | 0 | 0 | Exeter unpublished |
| p.(Met1?) | c.1A>G | Exon 1 | Start loss |  | HI | 0 | 0 | ([Arya, et al., 2014b](#_ENREF_20); [Greer, et al., 2007](#_ENREF_138)) |
| p.(Met1?) | c.1del | Exon 1 | Start loss |  | HI | 0 | 0 | ([Jahnavi, et al., 2014](#_ENREF_175)) |
| p.(Ala4Val) | c.11C>T | Exon 1 | Missense | Extracellular | HI | 0 | 0 | ([Banerjee, et al., 2011](#_ENREF_27)) |
| p.(Cys6Gly) | c.16T>G | Exon 1 | Missense | Extracellular | HI | 0 | 0 | ([Snider, et al., 2013](#_ENREF_356)) |
| p.(Cys6Ter) | c.18C>A | Exon 1 | Nonsense |  | HI | 0 | 0 | ([Ocal, et al., 2011](#_ENREF_274)) |
| p.(Gly7Arg) | c.19G>C | Exon1 | Missense | Extracellular | HI | 1 | 0.000005045 | ([Chen, et al., 2013](#_ENREF_61); [Suchi, et al., 2006](#_ENREF_365); [Yan, et al., 2007](#_ENREF_413)) |
| p.(Gly7Cys) | c.19G>T | Exon1 | Missense | Extracellular | HI | 0 | 0 | Paris unpublished |
| p.(Glu9Ter) | c.25G>T | Exon 1 | Nonsense |  | HI | 0 | 0 | Exeter unpublished |
| p.(Asn10ThrfsTer68) | c.29del | Exon 1 | Frameshift |  | HI | 0 | 0 | Exeter unpublished |
| p.(Ser12Ter) | c.35C>A | Exon1 | Nonsense |  | HI | 0 | 0 | ([Arya, et al., 2014b](#_ENREF_20); [Craigie, et al., 2018](#_ENREF_72)) |
| p.(Arg16Pro) | c.47G>C | Exon 1 | Missense | Extracellular | HI | 0 | 0 | ([Xu, et al., 2018](#_ENREF_409)) |
| p.(Val17Ala) | c.50T>C | Exon 1 | Missense | Extracellular | HI | 1 | 0.000004255 | ([Mohnike, et al., 2014](#_ENREF_256)) |
| p.(Gln19Ter) | c.55C>T | Exon 1 | Nonsense |  | HI | 1 | 0.000004209 | Exeter unpublished Odense unpublished |
| p.(Val21Asp) | c.62T>A | Exon1 | Missense | Extracellular | HI | 15 | 0.00005518 | ([Bellanne-Chantelot, et al., 2010](#_ENREF_31); [Sandal, et al., 2009](#_ENREF_329); [Suchi, et al., 2006](#_ENREF_365)) |
| p.(Val21GlyfsTer68) | c.61dup | Exon 1 | Frameshift |  | HI | 0 | 0 | ([Yorifuji, et al., 2011](#_ENREF_417)) |
| p.(Val21SerfsTer57) | c.61del | Exon 1 | Frameshift |  | HI | 0 | 0 | ([Jahnavi, et al., 2014](#_ENREF_175)) |
| p.(Asn24Lys) | c.72C>A | Exon1 | Missense | Extracellular | HI | 1 | 0.0000041 | ([Chen, et al., 2013](#_ENREF_61); [Del Roio Liberatore, et al., 2015](#_ENREF_86); [Peranteau, et al., 2006](#_ENREF_294); [Yan, et al., 2007](#_ENREF_413)) |
| p.(Gly25AlafsTer53) | c.74del | Exon 1 | Frameshift |  | HI | 0 | 0 | Exeter unpublished |
| p.(Cys26Ter) | c.78C>A | Exon 1 | Nonsense |  | HI | 0 | 0 | ([Guven, et al., 2016](#_ENREF_142)) |
| p.(Cys26Trp) | c.78C>G | Exon 1 | Missense | Extracellular | HI | 0 | 0 | Paris unpublished |
| p.(Phe27Ser) | c.80T>C | Exon1 | Missense | Extracellular | HI | 0 | 0 | ([Chen, et al., 2013](#_ENREF_61); [Fan, et al., 2015](#_ENREF_103); [Stanley, et al., 2004](#_ENREF_361); [Suchi, et al., 2006](#_ENREF_365); [Yan, et al., 2007](#_ENREF_413); [Zhou, et al., 2014](#_ENREF_421)) |
| p.(Val28CysfsTer61) | c.81dup | Exon 1 | Frameshift |  | HI | 0 | 0 | ([Snider, et al., 2013](#_ENREF_356)) |
| p.(Val28SerfsTer61) | c.81_82insA | Exon 1 | Frameshift |  | HI | 0 | 0 | Exeter unpublished |
| p.(Asp29Gly) | c.86A>G | Exon 1 | Missense | Extracellular | HI | 0 | 0 | ([Gong, et al., 2016](#_ENREF_136); [Snider, et al., 2013](#_ENREF_356)) |
| p.(Ala30Thr) | c.88G>A | Exon 1 | Missense | Extracellular | HI | 1 | 0.0000319 | ([Snider, et al., 2013](#_ENREF_356)) |
| p.(Ala30Val) | c.89C>T | Exon 1 | Missense | Extracellular | NDM HI* | 0 | 0 | ([Han, et al., 2016](#_ENREF_144); [Lin, et al., 2012](#_ENREF_224))* |
| p.(Leu31Pro) | c.92T>C | Exon 1 | Missense | Extracellular | HI | 0 | 0 | ([Snider, et al., 2013](#_ENREF_356)) |
| p.(Asn32Lys) | c.96C>G | Exon 1 | Missense | Extracellular | HI | 0 | 0 | ([Snider, et al., 2013](#_ENREF_356)) |
| p.(His36Tyr) | c.106C>T | Exon 1 | Missense | Transmembrane | HI | 1 | 0.00000407 | ([Xu, et al., 2018](#_ENREF_409)) |
| p.(His36Arg) | c.107A>G | Exon1 | Missense | Transmembrane | HI | 1 | 0.000004069 | ([Banerjee, et al., 2011](#_ENREF_27)) |
| p.(Leu40Arg) | c.119T>G | Exon 1 | Missense | Transmembrane | HI | 0 | 0 | ([Snider, et al., 2013](#_ENREF_356)) |
| p.(Pro45Leu) | c.134C>T | Exon 1 | Missense | Transmembrane | PNDM | 0 | 0 | ([Del Roio Liberatore, et al., 2015](#_ENREF_86); [Ellard, et al., 2007](#_ENREF_98)) |
| p.(Ile46Thr) | c.137T>C | Exon 1 | Missense | Transmembrane | HI | 0 | 0 | Paris unpublished |
| p.(Phe48del) | c.142_144del | Exon 1 | In frame deletion | Transmembrane | HI | 0 | 0 | ([Rozenkova, et al., 2015](#_ENREF_318)) |
| p.(Ile49Phe) | c.145A>T | Exon 1 | Missense | Transmembrane | DEND | 0 | 0 | ([Zwaveling-Soonawala, et al., 2011](#_ENREF_424)) |
| p.? | c.148+1G>A | Intron 1 | Aberrant splicing- |  | HI | 1 | 0.000004077 | ([Han, et al., 2016](#_ENREF_144)) |
| p.? | c.149-1G>A | Intron 1 | Aberrant splicing |  | HI | 0 | 0 | ([Snider, et al., 2013](#_ENREF_356)) |
| p.? | c.149-2A>G | Intron 1 | Aberrant splicing |  | HI | 0 | 0 | ([Snider, et al., 2013](#_ENREF_356)) |
| p.? | c.149-2A>C | Intron 1 | Aberrant splicing |  | HI | 0 | 0 | ([Xu, et al., 2018](#_ENREF_409)) |
| p.? | c.(148+1_149-1)_(290+1_291-1)del | Exon 2 | Deletion | Transmembrane | HI | 0 | 0 | Exeter unpublished  Odense unpublished |
| p.(Gly52GlufsTer26) | c.155del | Exon 2 | Frameshift |  | HI | 0 | 0 | ([Darendeliler, et al., 2002](#_ENREF_76); [Guven, et al., 2016](#_ENREF_142)) |
| p.(Gln54Ter) | c.160C>T | Exon 2 | Nonsense |  | HI | 0 | 0 | ([Arya, et al., 2014b](#_ENREF_20); [Hussain, et al., 2008](#_ENREF_161)) |
| p.(His59Pro) | c.176A>C | Exon 2 | Missense | Cytoplasmic | HI | 0 | 0 | ([Haliloglu, et al., 2018](#_ENREF_143)) |
| p.(Ile60Asn) | c.179T>A | Exon 2 | Missense | Cytoplasmic | HI | 0 | 0 | ([Galcheva, et al., 2017](#_ENREF_121)) |
| p.(Trp65Ter) | c.195G>A | Exon 2 | Nonsense |  | HI | 0 | 0 | Paris unpublished |
| p.(Gly70Arg) | c.208G>A | Exon 2 | Missense | Cytoplasmic | HI | 6 | 0.00002122 | ([Banerjee, et al., 2011](#_ENREF_27)) |
| p.(Gly70Glu) | c.209G>A | Exon 2 | Missense | Cytoplasmic | HI | 0 | 0 | ([Tornovsky, et al., 2004](#_ENREF_392)) |
| p.(Asn72Ser) | c.215A>G | Exon 2 | Missense | Cytoplasmic | PNDM | 0 | 0 | ([Ellard, et al., 2007](#_ENREF_98); [Shield, et al., 2008](#_ENREF_344)) |
| p.(Asn72Lys) | c.216C>A | Exon 2 | Missense | Cytoplasmic | HI | 0 | 0 | ([Xu, et al., 2018](#_ENREF_409)) |
| p.(Arg74Trp) | c.220C>T | Exon 2 | Missense | Cytoplasmic | HI | 3 | 0.00001061 | ([Arya, et al., 2014b](#_ENREF_20); [Chen, et al., 2013](#_ENREF_61); [Fan, et al., 2015](#_ENREF_103); [Fernandez-Marmiesse, et al., 2006](#_ENREF_106); [Pratt, et al., 2009](#_ENREF_305); [Shah, et al., 2015](#_ENREF_337); [Stanley, et al., 2004](#_ENREF_361); [Suchi, et al., 2003](#_ENREF_364); [Suchi, et al., 2006](#_ENREF_365); [Yan, et al., 2007](#_ENREF_413)) |
| p.(Arg74Gln) | c.221G>A | Exon 2 | Missense | Cytoplasmic | HI | 2 | 0.000007956 | ([Bellanne-Chantelot, et al., 2010](#_ENREF_31); [Nestorowicz, et al., 1998](#_ENREF_265); [Thakur, et al., 2011](#_ENREF_384)) |
| p.(Arg74Leu) | c.221G>T | Exon 2 | Missense | Cytoplasmic | HI | 1 | 0.000003978 | Odense unpublished |
| p.(Trp75CysfsTer12) | c.225_229del | Exon 2 | Frameshift |  | HI | 0 | 0 | Exeter unpublished |
| p.(Met80Arg) | c.239T>G | Exon 2 | Missense | Transmembrane | HI | 1 | 0.000003977 | ([Greer, et al., 2007](#_ENREF_138)) |
| p.(Val84Ile) | c.250G>A | Exon 2 | Missense | Transmembrane | Later-onset diabetes | 6 | 0.00002122 | ([Gonsorcikova, et al., 2011](#_ENREF_137)) |
| p.(Val86Ala) | c.257T>C | Exon 2 | Missense | Transmembrane | PNDM | 0 | 0 | ([Jahnavi, et al., 2013](#_ENREF_176); [Klupa, et al., 2009](#_ENREF_203); [Stanik, et al., 2007](#_ENREF_359)) |
| p.(Val86Gly) | c.257T>G | Exon 2 | Missense | Transmembrane | PNDM | 0 | 0 | ([Del Roio Liberatore, et al., 2015](#_ENREF_86); [Ellard, et al., 2007](#_ENREF_98)) |
| p.(Cys87GlnfsTer9) | c.259_268del | Exon 2 | Frameshift |  | HI | 0 | 0 | ([Bellanne-Chantelot, et al., 2010](#_ENREF_31)) |
| p.(Ile89Met) | c.267T>G | Exon 2 | Missense | Transmembrane | HI | 0 | 0 | ([Darendeliler, et al., 2002](#_ENREF_76)) |
| p.(Ile89MetfsTer10) | c.267del | Exon 2 | Frameshift |  | HI | 0 | 0 | ([Jahnavi, et al., 2014](#_ENREF_175)) |
| p.(Ala90Val) | c.269C>T | Exon 2 | Missense | Transmembrane | PNDM | 0 | 0 | ([Suzuki, et al., 2007](#_ENREF_367)) |
| p.(Gly92Ser) | c.274G>A | Exon 2 | Missense | Transmembrane | HI | 2 | 0.00007071 | ([Apperley, et al., 2019](#_ENREF_16)) |
| p.(Gly92Asp) | c.275G>A | Exon 2 | Missense | Transmembrane | HI | 0 | 0 | ([Craigie, et al., 2018](#_ENREF_72); [Otonkoski, et al., 2006](#_ENREF_284)) |
| p.(Ile93Thr) | c.278T>C | Exon 2 | Missense | Transmembrane | NDM | 2 | 0.000007955 | ([Busiah, et al., 2014](#_ENREF_49)) |
| p.(Ile93HisfsTer5) | c.276_277insCATC | Exon 2 | Frameshift |  | HI | 0 | 0 | ([Gong, et al., 2016](#_ENREF_136)) |
| p.? | c.291-2A>G | Intron 2 | Aberrant splicing |  | HI | 0 | 0 | ([Snider, et al., 2013](#_ENREF_356)) |
| p.? | c.291-2451_c.412+3656/c.291-2451_c.412+3656 | Exon 3 | Deletion |  | HI | 0 | 0 | ([Khawash, et al., 2015](#_ENREF_195)) |
| p.? | c.(290+1_291-1)_822+1_823-1)del | Exons 3-5 | Deletion |  | HI | 0 | 0 | Exeter unpublished |
| p.(Glu100Ter) | c.298G>T | Exon 3 | Nonsense |  | HI | 0 | 0 | ([Park, et al., 2011](#_ENREF_288)) |
| p.(His105Pro) | c.314A>C | Exon 3 | Missense | Transmembrane | HI | 0 | 0 | ([Zhang, et al., 2015b](#_ENREF_420)) |
| p.(Gly111Arg) | c.331G>C | Exon 3 | Missense | Transmembrane | HI | 2 | 0.00000812 | ([Fernandez-Marmiesse, et al., 2006](#_ENREF_106)) |
| p.(Gly111Arg) | c.331G>A | Exon 3 | Missense | Transmembrane | HI | 2 | 0.000007956 | ([Arbizu Lostao, et al., 2008](#_ENREF_17); [Arya, et al., 2014b](#_ENREF_20); [De Vroede, et al., 2004](#_ENREF_81); [Del Roio Liberatore, et al., 2015](#_ENREF_86); [Gong, et al., 2016](#_ENREF_136); [Jahnavi, et al., 2014](#_ENREF_175); [Ni, et al., 2019](#_ENREF_268); [Thakur, et al., 2011](#_ENREF_384); [Tornovsky, et al., 2004](#_ENREF_392); [Xu, et al., 2018](#_ENREF_409); [Yorifuji, et al., 2011](#_ENREF_417)) |
| p.(Ala113Val) | c.338C>T | Exon 3 | Missense | Transmembrane | HI | 0 | 0 | ([Arya, et al., 2014b](#_ENREF_20); [Christesen, et al., 2012](#_ENREF_64); [Otonkoski, et al., 2006](#_ENREF_284)) |
| p.(Met115Val) | c.343A>G | Exon 3 | Missense | Transmembrane | HI | 63 | 0.0002506 | ([Celik, et al., 2013](#_ENREF_56)) |
| p.(Ala116Pro) | c.346G>C | Exon 3 | Missense | Transmembrane | HI | 0 | 0 | ([Aguilar-Bryan and Bryan, 1999](#_ENREF_7); [Chan, et al., 2003](#_ENREF_58); [Chen, et al., 2013](#_ENREF_61); [Yan, et al., 2004](#_ENREF_412)) |
| p.(Glu128Lys) | c.382G>A | Exon 3 | Missense | Cytoplasmic | HI | 2 | 0.000007955 | ([Arya, et al., 2014b](#_ENREF_20); [Chen, et al., 2013](#_ENREF_61); [Craigie, et al., 2018](#_ENREF_72); [Han, et al., 2016](#_ENREF_144); [Hosy, et al., 2010](#_ENREF_155); [Pratt and Shyng, 2011](#_ENREF_304); [Pratt, et al., 2009](#_ENREF_305); [Yan, et al., 2007](#_ENREF_413); [Yorifuji, et al., 2011](#_ENREF_417)) |
| p.(Asn131Thr) | c.392A>C | Exon 3 | Missense | Cytoplasmic | HI | 0 | 0 | ([Mohnike, et al., 2014](#_ENREF_256)) |
| p.(Asn131Lys) | c.393C>A | Exon 3 | Missense | Cytoplasmic | PNDM | 0 | 0 | ([Madani, 2019](#_ENREF_231)) |
| p.(Phe132Leu) | c.394T>C | Exon 3 | Missense | Cytoplasmic | DEND PNDM | 0 | 0 | ([Ellard, et al., 2007](#_ENREF_98); [Hosy, et al., 2010](#_ENREF_155); [Proks, et al., 2006a](#_ENREF_307); [Zhang, et al., 2015a](#_ENREF_419)) |
| p.(Phe132Val) | c.394T>G | Exon 3 | Missense | Cytoplasmic | PNDM | 0 | 0 | ([Ellard, et al., 2007](#_ENREF_98)) |
| p.(Pro133Arg) | c.398C>G | Exon 3 | Missense | Cytoplasmic | HI | 0 | 0 | Seattle unpublished |
| p.(Leu135Val) | c.403C>G | Exon 3 | Missense | Transmembrane | HI | 9 | 0.00003182 | ([Bellanne-Chantelot, et al., 2010](#_ENREF_31)) |
| p.(Leu135Pro) | c.404T>C | Exon 3 | Missense | Transmembrane | PNDM | 0 | 0 | ([Patch, et al., 2007](#_ENREF_290)) |
| p.? | c.(412+1_413-1)_(579+1_580-1)del | Exon 4 | Deletion | Transmembrane | HI | 0 | 0 | Paris unpublished |
| p.(Leu139CysfsTer38) | c.415del | Exon 4 | Frameshift |  | HI | 0 | 0 | ([Bendix, et al., 2018](#_ENREF_32)) |
| p.(Trp143Ter) | c.428G>A | Exon 4 | Nonsense |  | HI | 0 | 0 | ([Bellanne-Chantelot, et al., 2010](#_ENREF_31); [Snider, et al., 2013](#_ENREF_356); [Xu, et al., 2018](#_ENREF_409)) |
| p.(Ile148Thr) | c.443T>C | Exon 4 | Missense | Transmembrane | HI | 2 | 0.000008271 | ([Mohnike, et al., 2014](#_ENREF_256)) |
| p.(Ile152SerfsTer25) | c.453del | Exon 4 | Frameshift |  | HI | 0 | 0 | ([Martinez, et al., 2016](#_ENREF_243)) |
| p.(Gln166Ter) | c.496C>T | Exon 4 | Nonsense |  | HI | 0 | 0 | ([Bellanne-Chantelot, et al., 2010](#_ENREF_31)) |
| p.(Arg168Cys) | c.502C>T | Exon 4 | Missense | Transmembrane | HI | 1 | 0.0000323 | ([Greer, et al., 2007](#_ENREF_138); [Jain, et al., 2012](#_ENREF_177); [Kumaran, et al., 2010](#_ENREF_214)) |
| p.(Phe169Val) | c.505T>G | Exon 4 | Missense | Transmembrane | PNDM | 0 | 0 | ([Rubio-Cabezas, et al., 2012](#_ENREF_319)) |
| p.(Leu171Phe) | c.511C>T | Exon 4 | Missense | Transmembrane | HI | 0 | 0 | ([Isik, et al., 2019](#_ENREF_171)) |
| p.(Leu171Pro) | c.512T>C | Exon 4 | Missense | Transmembrane | HI | 0 | 0 | ([Snider, et al., 2013](#_ENREF_356)) |
| p.(Thr172HisfsTer100) | c.512dup | Exon 4 | Frameshift |  | HI | 0 | 0 | ([Banerjee, et al., 2011](#_ENREF_27)) |
| p.(Gly173Arg) | c.517G>A | Exon 4 | Missense | Transmembrane | HI | 0 | 0 | ([Hardy, et al., 2007](#_ENREF_146)) |
| p.(Leu175AlafsTer97) | c.522dup | Exon 4 | Frameshift |  | HI | 0 | 0 | Exeter unpublished |
| p.(Leu175Gln) | c.524T>A | Exon 4 | Missense | Transmembrane | HI | 1 | 0.000004311 | ([Snider, et al., 2013](#_ENREF_356)) |
| p.(Tyr179Cys) | c.536A>G | Exon 4 | Missense | Transmembrane | HI | 0 | 0 | ([Bellanne-Chantelot, et al., 2010](#_ENREF_31); [Damaj, et al., 2008](#_ENREF_75); [Xu, et al., 2018](#_ENREF_409)) |
| p.(Tyr179Ter) | c.536_539del | Exon 4 | Nonsense |  | HI | 1 | 0.000004261 | ([Ellard, et al., 2007](#_ENREF_98); [Stanley, et al., 2004](#_ENREF_361); [Suchi, et al., 2006](#_ENREF_365)) |
| p.(Val185GlyfsTer8) | c.554del | Exon 4 | Frameshift |  | HI | 0 | 0 | ([Arya, et al., 2014b](#_ENREF_20)) |
| p.(Val187Asp) | c.560T>A | Exon 4 | Missense | Transmembrane | HI | 42 | 0.0001576 | ([Arya, et al., 2014c](#_ENREF_21); [Chan, et al., 2003](#_ENREF_58); [Chen, et al., 2013](#_ENREF_61); [Henquin, et al., 2011](#_ENREF_151); [Huopio, et al., 2002](#_ENREF_157); [Otonkoski, et al., 1999](#_ENREF_283); [Otonkoski, et al., 2006](#_ENREF_284); [Saint-Martin, et al., 2011](#_ENREF_324); [Sandal, et al., 2009](#_ENREF_329); [Yan, et al., 2004](#_ENREF_412)) |
| p.(Asn188Ser) | c.563A>G | Exon 4 | Missense | Transmembrane | HI | 0 | 0 | ([Christesen, et al., 2001](#_ENREF_63); [Del Roio Liberatore, et al., 2015](#_ENREF_86); [Gong, et al., 2016](#_ENREF_136); [Nestorowicz, et al., 1998](#_ENREF_265); [Ni, et al., 2019](#_ENREF_268); [Shyng, et al., 1998](#_ENREF_350); [Stanley, et al., 2004](#_ENREF_361)) |
| p.? | c.580-2A>G | Intron 4 | Aberrant splicing |  | HI | 0 | 0 | Exeter unpublished  Odense unpublished |
| p.? | c.580-1G>C | Intron 4 | Aberrant splicing |  | HI | 0 | 0 | ([Arya, et al., 2014b](#_ENREF_20); [Bellanne-Chantelot, et al., 2010](#_ENREF_31); [Bendix, et al., 2018](#_ENREF_32); [Henquin, et al., 2011](#_ENREF_151)) |
| p.(Tyr195Ter) | c.584dup | Exon 5 | Nonsense |  | HI | 0 | 0 | ([Fernandez-Marmiesse, et al., 2006](#_ENREF_106)) |
| p.(Ile196Asn) | c.587T>A | Exon 5 | Missense | Cytoplasmic | PNDM | 0 | 0 | ([Patch, et al., 2007](#_ENREF_290)) |
| p.(Thr200ArgfsTer6) | c.597_598dup | Exon 5 | Frameshift | - | HI | 0 | 0 | ([Jindal, et al., 2014](#_ENREF_180)) |
| p.(Pro206Leu) | c.617C>T | Exon 5 | Missense | Cytoplasmic | TNDM | 0 | 0 | Exeter unpublished |
| p.(Pro207Ser) | c.619C>T | Exon 5 | Missense | Cytoplasmic | PNDM | 0 | 0 | ([Ellard, et al., 2007](#_ENREF_98)) |
| p.(Pro207AlafsTer61) | c.619_629del | Exon 5 | Frameshift |  | HI | 0 | 0 | ([Jahnavi, et al., 2014](#_ENREF_175)) |
| p.(Glu208Lys) | c.622G>A | Exon 5 | Missense | Cytoplasmic | TNDM PNDM | 0 | 0 | ([Ellard, et al., 2007](#_ENREF_98); [Vaxillaire, et al., 2007](#_ENREF_396); [Zhou, et al., 2010](#_ENREF_422)) |
| p.(Glu208Ter) | c.622G>T | Exon 5 | Nonsense |  | HI | 0 | 0 | ([Snider, et al., 2013](#_ENREF_356)) |
| p.(Asp209Asn) | c.625G>A | Exon 5 | Missense | Cytoplasmic | PNDM | 0 | 0 | ([Rafiq, et al., 2008](#_ENREF_314)) |
| p.(Asp209Glu) | c.627C>A | Exon 5 | Missense | Cytoplasmic | TNDM PNDM | 0 | 0 | ([Ellard, et al., 2007](#_ENREF_98); [Flanagan, et al., 2007](#_ENREF_113); [Zhang, et al., 2015a](#_ENREF_419)) |
| p.(Gln211Lys) | c.631C>A | Exon 5 | Missense | Cytoplasmic | PNDM | 0 | 0 | ([Codner, et al., 2007](#_ENREF_68); [Ooi and Wu, 2012](#_ENREF_278)) |
| p.(Asp212Asn) | c.634G>A | Exon 5 | Missense | Cytoplasmic | TNDM | 0 | 0 | ([Flanagan, et al., 2007](#_ENREF_113)) |
| p.(Asp212Tyr) | c.634G>T | Exon 5 | Missense | Cytoplasmic | PNDM | 0 | 0 | ([Jahnavi, et al., 2013](#_ENREF_176)) |
| p.(Asp212Ile) | c.634_635delinsAT | Exon 5 | Missense | Cytoplasmic | TNDM | 0 | 0 | ([Flanagan, et al., 2007](#_ENREF_113)) |
| p.(Asp212Gly) | c.635A>G | Exon 5 | Missense | Cytoplasmic | NDM | 0 | 0 | Exeter unpublished |
| p.(Asp212Glu) | c.636C>G | Exon 5 | Missense | Cytoplasmic | NDM | 0 | 0 | Chicago unpublished |
| p.(Leu213Pro) | c.638T>C | Exon 5 | Missense | Cytoplasmic | i-DEND DEND | 0 | 0 | ([Fanciullo, et al., 2012](#_ENREF_104); [Russo, et al., 2011b](#_ENREF_321)) |
| p.(Leu213Arg) | c.638T>G | Exon 5 | Missense | Cytoplasmic | i-DEND | 0 | 0 | ([Babenko, et al., 2006](#_ENREF_24)) |
| p.(Val215Ile) | c.643G>A | Exon 5 | Missense | Cytoplasmic | PNDM | 0 | 0 | ([Rubio-Cabezas, et al., 2012](#_ENREF_319)) |
| p.(Arg216Cys) | c.646C>T | Exon 5 | Missense | Cytoplasmic | TNDM | 4 | 0.00001591 | ([Hashimoto, et al., 2017](#_ENREF_149)) |
| p.(Gln219Ter) | c.655C>T | Exon 5 | Nonsense |  | HI | 0 | 0 | ([Bellanne-Chantelot, et al., 2010](#_ENREF_31); [Giurgea, et al., 2004](#_ENREF_125); [Hardy, et al., 2007](#_ENREF_146); [Saint-Martin, et al., 2015](#_ENREF_325)) |
| p.(Val222Met) | c.664G>A | Exon 5 | Missense | Cytoplasmic | Later-onset diabetes | 6 | 0.00002121 | ([Bowman, et al., 2012](#_ENREF_42)) |
| p.(Leu225Pro) | c.674T>C | Exon 5 | Missense | Cytoplasmic | PNDM | 0 | 0 | ([Bonnefond, et al., 2014](#_ENREF_39); [Ellard, et al., 2007](#_ENREF_98); [Masia, et al., 2007a](#_ENREF_245)) |
| p.(Leu225_Ser226insThrLysTer) | c.674_675insCACGAAGTAGCA | Exon 5 | Nonsense |  | HI | 0 | 0 | Odense unpublished |
| p.(Gly228Asp) | c.683G>A | Exon 5 | Missense | Cytoplasmic | HI | 0 | 0 | ([Bellanne-Chantelot, et al., 2010](#_ENREF_31); [Giurgea, et al., 2004](#_ENREF_125); [Greer, et al., 2007](#_ENREF_138); [Henquin, et al., 2011](#_ENREF_151); [Muzyamba, et al., 2007](#_ENREF_260); [Valayannopoulos, et al., 2007](#_ENREF_394)) |
| p.(Thr229Asn) | c.686C>A | Exon 5 | Missense | Cytoplasmic | PNDM | 0 | 0 | ([Hashimoto, et al., 2017](#_ENREF_149)) |
| p.(Thr229Ile) | c.686C>T | Exon 5 | Missense | Cytoplasmic | TNDM PNDM | 4 | 0.00001414 | ([Ellard, et al., 2007](#_ENREF_98); [Patch, et al., 2007](#_ENREF_290)) |
| p.(Tyr230Cys) | c.689A>G | Exon 5 | Missense | Cytoplasmic | HI | 26 | 0.0001034 | Odense unpublished |
| p.(Trp231Arg) | c.691T>C | Exon 5 | Missense | Cytoplasmic | HI | 0 | 0 | ([Sandal, et al., 2009](#_ENREF_329)) |
| p.(Trp231Ter) | c.692G>A | Exon 5 | Nonsense | - | HI | 0 | 0 | ([Kalish, et al., 2016](#_ENREF_186)) |
| p.(Trp232Gly) | c.694T>G | Exon 5 | Missense | Cytoplasmic | HI | 0 | 0 | ([Demirbilek, et al., 2014](#_ENREF_88)) |
| p.(Trp232Ter) | c.695G>A | Exon 5 | Nonsense | - | HI | 0 | 0 | ([Petraitiene, et al., 2014](#_ENREF_295); [Snider, et al., 2013](#_ENREF_356)) |
| p.(Met233Arg) | c.698T>G | Exon 5 | Missense | Cytoplasmic | HI | 0 | 0 | ([Fernandez-Marmiesse, et al., 2006](#_ENREF_106)) |
| p.(Ala235Val) | c.704C>T | Exon 5 | Missense | Cytoplasmic | NDM | 0 | 0 | Exeter unpublished |
| p.(His241ThrfsTer17) | c.721del | Exon 5 | Frameshift |  | HI | 0 | 0 | ([Ni, et al., 2019](#_ENREF_268)) |
| p.(Lys242ArgfsTer16) | c.725del | Exon 5 | Frameshift |  | HI | 0 | 0 | ([Powell, et al., 2011](#_ENREF_302)) |
| p.(Lys243_Lys252del) | c.727_756del | Exon 5 | In frame deletion | Cytoplasmic | HI | 0 | 0 | ([Bellanne-Chantelot, et al., 2010](#_ENREF_31)) |
| p.(Arg248Ter) | c.742C>T | Exon 5 | Nonsense |  | HI | 4 | 0.00001591 | ([Aguilar-Bryan and Bryan, 1999](#_ENREF_7); [Bellanne-Chantelot, et al., 2010](#_ENREF_31); [Del Roio Liberatore, et al., 2015](#_ENREF_86); [Fernandez-Marmiesse, et al., 2006](#_ENREF_106); [Martinez, et al., 2016](#_ENREF_243); [Ni, et al., 2019](#_ENREF_268); [Sandal, et al., 2009](#_ENREF_329)) |
| p.(Pro254Ser) | c.760C>T | Exon 5 | Missense | Cytoplasmic | PNDM | 2 | 0.000007953 | ([Flanagan, et al., 2014](#_ENREF_108)) |
| p.(Pro254Leu) | c.761C>T | Exon 5 | Missense | Cytoplasmic | HI | 0 | 0 | Odense unpublished |
| p.(Tyr263Asp) | c.787T>G | Exon 5 | Missense | Cytoplasmic | PNDM | 5 | 0.00001988 | ([Ellard, et al., 2007](#_ENREF_98)) |
| p.(Cys267Ter) | c.801C>A | Exon 5 | Nonsense |  | HI | 0 | 0 | ([Sandal, et al., 2009](#_ENREF_329)) |
| p.(Ala269ProfsTer90) | c.805del | Exon 5 | Frameshift |  | HI | 1 | 0.000003979 | ([Snider, et al., 2013](#_ENREF_356)) |
| p.(Ile287LeufsTer78) | c.836_852dup | Exon 5 | Frameshift |  | HI | 0 | 0 | ([Mohnike, et al., 2014](#_ENREF_256)) |
| p.(Gln282Ter) | c.844C>T | Exon 6 | Nonsense |  | HI | 0 | 0 | Exeter unpublished |
| p.(Ala284GlyfsTer99) | c.850dup | Exon 6 | Frameshift |  | HI | 0 | 0 | ([Xu, et al., 2018](#_ENREF_409)) |
| p.(Trp288Ter) | c.863G>A | Exon 6 | Nonsense |  | HI | 0 | 0 | ([Sang, et al., 2014a](#_ENREF_331); [Xu, et al., 2013](#_ENREF_408)) |
| p.(Gly296Arg) | c.886G>A | Exon 6 | Missense | Cytoplasmic | TNDM | 4 | 0.00001423 | ([Cao, et al., 2016](#_ENREF_53); [Lin, et al., 2012](#_ENREF_224)) |
| p.(Arg298Cys) | c.892C>T | Exon 6 | Missense | Cytoplasmic | HI | 61 | 0.0002169 | ([Snider, et al., 2013](#_ENREF_356)) |
| p.(Arg306His) | c.917G>A | Exon 6 | Missense | Cytoplasmic | NDM | 0 | 0 | ([Patch, et al., 2007](#_ENREF_290)) |
| p.(Asp310Asn) | c.928G>A | Exon 6 | Missense | Cytoplasmic | HI | 3 | 0.00001195 | ([Fernandez-Marmiesse, et al., 2006](#_ENREF_106); [Martinez, et al., 2016](#_ENREF_243); [Pinney, et al., 2008](#_ENREF_299); [Salomon-Estebanez, et al., 2016](#_ENREF_328)) |
| p.(Asp310Val) | c.929A>T | Exon 6 | Missense | Cytoplasmic | HI | 1 | 0.000003985 | ([Hardy, et al., 2007](#_ENREF_146)) |
| p.(Gly316Arg) | c.946G>A | Exon 6 | Missense | Transmembrane | HI | 3 | 0.00001062 | ([Craigie, et al., 2018](#_ENREF_72); [Wu, et al., 2016](#_ENREF_406)) |
| p.(Pro317HisfsTer42) | c.950del | Exon 6 | Frameshift |  | HI | 0 | 0 | ([Bellanne-Chantelot, et al., 2010](#_ENREF_31); [Nestorowicz, et al., 1998](#_ENREF_265)) |
| p.(Val324Met) | c.970G>A | Exon 6 | Missense | Transmembrane | TNDM PNDM | 0 | 0 | ([Flanagan, et al., 2007](#_ENREF_113); [Russo, et al., 2011b](#_ENREF_321); [Vaxillaire, et al., 2007](#_ENREF_396); [Zhou, et al., 2010](#_ENREF_422)) |
| p.(Lys329Ter) | c.985A>T | Exon 6 | Nonsense |  | HI | 0 | 0 | Exeter unpublished |
| p.? | c.1011+1G>A | Intron 6 | Aberrant splicing |  | HI | 0 | 0 | ([Snider, et al., 2013](#_ENREF_356)) |
| p.? | c.1012-3C>G | Intron 6 | Aberrant splicing |  | HI | 0 | 0 | ([Sandal, et al., 2009](#_ENREF_329)) |
| p.? | c.1012-2A>G | Intron 6 | Aberrant splicing |  | HI | 0 | 0 | Exeter unpublished, Odense unpublished |
| p.(Tyr344Ter) | c.1032C>G | Exon 7 | Nonsense |  | HI | 0 | 0 | ([Bendix, et al., 2018](#_ENREF_32)) |
| p.(Glu350Gly) | c.1049A>G | Exon 7 | Missense | Extracellular | PNDM | 0 | 0 | Exeter unpublished |
| p.(Glu350Asp) | c.1050G>C | Exon 7 | Missense | Extracellular | TNDM | 0 | 0 | ([Takagi, et al., 2016](#_ENREF_370)) |
| p.(Tyr356Cys) | c.1067A>G | Exon 7 | Missense | Transmembrane | Diabetes IGT | 11 | 0.00003889 | ([Riveline, et al., 2012](#_ENREF_317); [Tarasov, et al., 2008](#_ENREF_381)) |
| p.(Tyr356Ter) | c.1068C>G | Exon 7 | Nonsense |  | HI | 0 | 0 | Exeter unpublished |
| p.(Val360Ala) | c.1079T>C | Exon 7 | Missense | Transmembrane | TNDM | 0 | 0 | Exeter unpublished |
| p.(Leu362ArgfsTer26) | c.1085del | Exon 7 | Frameshift |  | HI | 0 | 0 | Exeter unpublished |
| p.(Leu366Phe) | c.1096C>T | Exon 7 | Missense | Transmembrane | HI | 0 | 0 | Odense unpublished |
| p.(Arg370Gly) | c.1108A>G | Exon 7 | Missense | Transmembrane | HI | 0 | 0 | ([Pinney, et al., 2008](#_ENREF_299)) |
| p.(Arg370Ser) | c.1110G>C | Exon 7 | Missense | Transmembrane | HI | 0 | 0 | ([Abdulhadi-Atwan, et al., 2008](#_ENREF_2)) |
| p.(Thr371Ile) | c.1112C>T | Exon 7 | Missense | Transmembrane | HI | 2 | 0.000007953 | Paris unpublished |
| p.(Gln374Ter) | c.1120C>T | Exon 7 | Nonsense |  | HI | 0 | 0 | Exeter unpublished |
| p.(Ala375HisfsTer13) | c.1122del | Exon 7 | Frameshift |  | HI | 0 | 0 | ([Ohkubo, et al., 2005](#_ENREF_275)) |
| p.(Ala380ProfsTer8) | c.1138del | Exon 7 | Frameshift |  | HI | 1 | 0.000003976 | Exeter unpublished |
| p.(Glu382Lys) | c.1144G>A | Exon 7 | Missense | Cytoplasmic | PNDM | 0 | 0 | ([Aydin, et al., 2012](#_ENREF_22); [Ellard, et al., 2007](#_ENREF_98)) |
| p.(Glu382Val) | c.1145A>T | Exon 7 | Missense | Cytoplasmic | PNDM | 0 | 0 | ([Dalvi, et al., 2017](#_ENREF_74); [Flanagan, et al., 2014](#_ENREF_108)) |
| p.(Gly384Ter) | c.1150_1159del | Exon 7 | Nonsense |  | HI | 0 | 0 | Exeter unpublished |
| p.(Gly389Arg) | c.1165G>A | Exon 7 | Missense | Cytoplasmic | HI | 1 | 0.000003976 | ([Rozenkova, et al., 2015](#_ENREF_318)) |
| p.(Gln392His) | c.1176G>C | Exon 7 | Missense | Cytoplasmic | HI | 0 | 0 | ([Ince, et al., 2014](#_ENREF_165)) |
| p.(Gln392=) | c.1176G>A | Exon 7 | Aberrant splicing |  | HI | 0 | 0 | ([Bellanne-Chantelot, et al., 2010](#_ENREF_31); [Mohnike, et al., 2014](#_ENREF_256)) |
| p.? | c.1176+1G>A | Intron 7 | Aberrant splicing |  | HI | 0 | 0 | ([Fan, et al., 2015](#_ENREF_103)) |
| p.? | c.1176+2T>C | Intron 7 | Aberrant splicing |  | HI | 7 | 0.00002784 | ([Hardy, et al., 2007](#_ENREF_146); [Henquin, et al., 2011](#_ENREF_151); [Mohnike, et al., 2014](#_ENREF_256)) |
| p.? | c.1177-53_c.1777-55del | Intron 7 | Aberrant splicing |  | HI | 0 | 0 | ([Aguilar-Bryan and Bryan, 1999](#_ENREF_7)) |
| p.? | c.(1176+1_1177-1)_(1332+1_1333-1)del | Exon 8 | In frame deletion | Cytoplasmic | HI | 0 | 0 | ([Bellanne-Chantelot, et al., 2010](#_ENREF_31)) |
| p.(Asn406Asp) | c.1216A>G | Exon 8 | Missense | Cytoplasmic | HI | 0 | 0 | ([Nestorowicz, et al., 1998](#_ENREF_265)) |
| p.(Ser408Pro) | c.1222T>C | Exon 8 | Missense | Cytoplasmic | HI | 0 | 0 | ([Calabria, et al., 2012](#_ENREF_51); [Snider, et al., 2013](#_ENREF_356)) |
| N/A | c.1260_1290ins31 | Exon 8 | Frameshift |  | HI | N/A | N/A | ([Aguilar-Bryan and Bryan, 1999](#_ENREF_7)) |
| p.(Val421GlyfsTer74) | c.1261dup | Exon 8 | Frameshift |  | HI | 0 | 0 | ([Lee, et al., 2015](#_ENREF_219)) |
| p.(Asp424Val) | c.1271A>T | Exon 8 | Missense | Cytoplasmic | NDM | 0 | 0 | ([Anik, et al., 2014](#_ENREF_15)) |
| p.(Met429Ter) | c.1254_1284dup | Exon 8 | Nonsense |  | HI | 4 | 0.00001591 | ([Arya, et al., 2014b](#_ENREF_20); [Banerjee, et al., 2011](#_ENREF_27); [Snider, et al., 2013](#_ENREF_356)) |
| p.(Trp430Ter) | c.1290G>A | Exon 8 | Nonsense |  | HI | 1 | 0.000003977 | ([Park, et al., 2011](#_ENREF_288); [Snider, et al., 2013](#_ENREF_356)) |
| p.(Leu434Ter) | c.1301T>A | Exon 8 | Nonsense |  | HI | 0 | 0 | ([Guven, et al., 2016](#_ENREF_142)) |
| p.(Cys435Arg) | c.1303T>C | Exon 8 | Missense | Transmembrane | TNDM Diabetes | 0 | 0 | ([Babenko, et al., 2006](#_ENREF_24); [Bourron, et al., 2012](#_ENREF_41); [Riveline, et al., 2012](#_ENREF_317)) |
| p.(Leu438Phe) | c.1312C>T | Exon 8 | Missense | Transmembrane | PNDM | 0 | 0 | ([Patch, et al., 2007](#_ENREF_290)) |
| p.(Trp439Ter) | c.1317G>A | Exon 8 | Nonsense |  | HI | 0 | 0 | ([Martinez, et al., 2016](#_ENREF_243)) |
| p.(Gln444Ter) | c.1330C>T | Exon 8 | Nonsense |  | HI | 0 | 0 | ([Craigie, et al., 2018](#_ENREF_72); [Jahnavi, et al., 2014](#_ENREF_175)) |
| p.(Gln444Arg) | c.1331A>G | Exon 8 | Missense | Transmembrane | HI | 0 | 0 | ([Bellanne-Chantelot, et al., 2010](#_ENREF_31); [Damaj, et al., 2008](#_ENREF_75)) |
| p.(Gln444His) | c.1332G>T | Exon 8 | Missense | Transmembrane | HI | 0 | 0 | ([Bendix, et al., 2018](#_ENREF_32); [Flanagan, et al., 2012](#_ENREF_107); [Hardy, et al., 2007](#_ENREF_146); [Mohnike, et al., 2014](#_ENREF_256); [Rozenkova, et al., 2015](#_ENREF_318)) |
| p.? | c.1332+1G>A | Intron 8 | Aberrant splicing |  | HI | 0 | 0 | Paris unpublished |
| p.? | c.1332+3A>G | Intron 8 | Aberrant splicing |  | HI | 0 | 0 | Exeter unpublished |
| p.? | c.1332+3A>T | Intron 8 | Aberrant splicing |  | HI | 0 | 0 | ([Snider, et al., 2013](#_ENREF_356)) |
| p.? | c.1333-1013A>G | Intron 8 | Aberrant splicing |  | HI | 0 | 0 | ([Arya, et al., 2014b](#_ENREF_20); [Flanagan, et al., 2013](#_ENREF_114)) |
| p.? | c.1333-1G>A | Intron 8 | Aberrant splicing |  | HI | 0 | 0 | ([Mohnike, et al., 2014](#_ENREF_256)) |
| p.? | c.(1332+1_1333-1)_(1630+1_1631-1)del | Exons 9-10 | Deletion | Transmembrane | HI | 0 | 0 | ([Fernandez-Marmiesse, et al., 2006](#_ENREF_106)) |
| p.? | c.(1332+1_1333-1)_(1671+1_1672-1)dup | Exon 9-11 | Duplication | Transmembrane | HI | 0 | 0 | Exeter unpublished |
| p.(Ile446MetfsTer4) | c.1338del | Exon 9 | Nonsense |  | HI | 0 | 0 | ([Tanizawa, et al., 2000](#_ENREF_378)) |
| p.(Val447LeufsTer4) | c.1337_1338dup | Exon 9 | Frameshift |  | HI | 0 | 0 | Paris unpublished  Odense unpublished |
| p.(Ile450SerfsTer44) | c.1347_1348del | Exon 9 | Frameshift |  | HI | 0 | 0 | ([Fernandez-Marmiesse, et al., 2006](#_ENREF_106)) |
| p.(Leu451Pro) | c.1352T>C | Exon 9 | Missense | Transmembrane | TNDM | 0 | 0 | ([Flanagan, et al., 2007](#_ENREF_113)) |
| p.(Gly457GlufsTer5) | c.1370del | Exon 9 | Frameshift |  | HI | 0 | 0 | ([Snider, et al., 2013](#_ENREF_356)) |
| p.(Ser459Arg) | c.1375A>C | Exon 9 | Missense | Transmembrane | TNDM | 0 | 0 | ([Iafusco, et al., 2012](#_ENREF_162)) |
| p.(Gln474Ter) | c.1420C>T | Exon 9 | Nonsense |  | HI | 0 | 0 | ([Henquin, et al., 2011](#_ENREF_151); [Suchi, et al., 2006](#_ENREF_365)) |
| p.(Gln474Arg) | c.1421A>G | Exon 9 | Missense | Transmembrane | HI | 0 | 0 | ([Christesen, et al., 2007](#_ENREF_62); [Macmullen, et al., 2011](#_ENREF_230); [Sang, et al., 2014a](#_ENREF_331)) |
| p.(Ala478del) | c.1432_1434del | Exon 9 | In frame deletion | Transmembrane | HI | 0 | 0 | ([Rozenkova, et al., 2015](#_ENREF_318)) |
| p.(Ala478Asp) | c.1433C>A | Exon 9 | Missense | Transmembrane | HI | 0 | 0 | ([Bennett, et al., 2015](#_ENREF_33); [Macmullen, et al., 2011](#_ENREF_230)) |
| p.(Gln485Arg) | c.1454A>G | Exon 9 | Missense | Cytoplasmic | Later-onset diabetes | 0 | 0 | ([Bowman, et al., 2012](#_ENREF_42)) |
| p.(Gln485His) | c.1455G>C | Exon 9 | Missense | Cytoplasmic | PNDM | 0 | 0 | ([Bonnefond, et al., 2010](#_ENREF_37)) |
| p.(Thr488Ile) | c.1463C>T | Exon 9 | Missense | Cytoplasmic | TNDM | 0 | 0 | ([Marshall, et al., 2015](#_ENREF_240)) |
| p.? | c.1467+5G>A | Intron 9 | Aberrant splicing |  | HI | 0 | 0 | ([Powell, et al., 2011](#_ENREF_302); [Salomon-Estebanez, et al., 2016](#_ENREF_328)) |
| p.? | c.1467+6T>G | Intron 9 | Aberrant splicing |  | HI | 0 | 0 | Paris unpublished |
| p.? | c.1468-52G>A | Intron 9 | Aberrant splicing |  | HI | 8 | 0.00002918 | ([Snider, et al., 2013](#_ENREF_356)) |
| p.? | c.1468-48G>A | Intron 9 | Aberrant splicing |  | HI | 0 | 0 | Exeter unpublished |
| p.(Glu490Ter) | c.1468G>T | Exon 10 | Nonsense |  | HI | 0 | 0 | ([Hardy, et al., 2007](#_ENREF_146); [Sandal, et al., 2009](#_ENREF_329)) |
| p.(Tyr491Ter) | c.1473T>G | Exon 10 | Nonsense |  | HI | 0 | 0 | ([Gong, et al., 2016](#_ENREF_136)) |
| p.(Tyr491Ter) | c.1468_1471dup | Exon 10 | Nonsense |  | HI | 0 | 0 | ([Snider, et al., 2013](#_ENREF_356)) |
| p.(Asn493Lys) | c.1479T>A | Exon 10 | Missense | Cytoplasmic | HI | 0 | 0 | ([Guven, et al., 2016](#_ENREF_142)) |
| p.(Arg495Gln) | c.1484G>A | Exon 10 | Missense | Cytoplasmic | HI | 1 | 0.000003994 | ([Sang, et al., 2014a](#_ENREF_331); [Stanley, et al., 2004](#_ENREF_361); [Yan, et al., 2007](#_ENREF_413)) |
| p.(Asn500GlnfsTer122) | c.1497dup | Exon 10 | Frameshift |  | HI | 0 | 0 | Exeter unpublished |
| p.(Glu501Leu) | N/A | Exon 10 | Missense | Cytoplasmic | HI | N/A | N/A | ([Hardy, et al., 2007](#_ENREF_146)) |
| p.(Glu501Lys) | c.1501G>A | Exon 10 | Missense | Cytoplasmic | HI | 2 | 0.000007087 | ([Gong, et al., 2016](#_ENREF_136); [Ni, et al., 2019](#_ENREF_268); [Stanley, et al., 2004](#_ENREF_361); [Suchi, et al., 2006](#_ENREF_365); [Yan, et al., 2007](#_ENREF_413)) |
| p.(Leu503Pro) | c.1508T>C | Exon 10 | Missense | Cytoplasmic | HI | 0 | 0 | ([Bellanne-Chantelot, et al., 2010](#_ENREF_31); [Sandal, et al., 2009](#_ENREF_329); [Suchi, et al., 2006](#_ENREF_365); [Yan, et al., 2007](#_ENREF_413)) |
| p.(Gly505Arg) | c.1513G>C | Exon 10 | Missense | Cytoplasmic | HI | 0 | 0 | Exeter unpublished  Paris unpublished |
| p.(Leu508Pro) | c.1523T>C | Exon 10 | Missense | Cytoplasmic | HI | 0 | 0 | ([Aguilar-Bryan and Bryan, 1999](#_ENREF_7); [Salomon-Estebanez, et al., 2016](#_ENREF_328)) |
| p.(Lys510Asn) | c.1530G>T | Exon 10 | Missense | Cytoplasmic | HI | 0 | 0 | ([Mohnike, et al., 2014](#_ENREF_256)) |
| p.(Leu511Met) | c.1531C>A | Exon 10 | Missense | Cytoplasmic | HI | 0 | 0 | ([Bellanne-Chantelot, et al., 2010](#_ENREF_31)) |
| p.(Leu511Pro) | c.1532T>C | Exon 10 | Missense | Cytoplasmic | HI | 1 | 0.00003186 | ([Li, et al., 2017](#_ENREF_222)) |
| p.(Tyr512Ter) | c.1536C>A | Exon 10 | Nonsense |  | HI | 0 | 0 | ([Meder, et al., 2015](#_ENREF_250); [Ohkubo, et al., 2005](#_ENREF_275)) |
| p.(Tyr512Ter) | c.1536C>G | Exon 10 | Nonsense |  | HI | 0 | 0 | ([Szymanowski, et al., 2016](#_ENREF_368)) |
| p.(Arg521Pro) | c.1562G>C | Exon 10 | Missense | Cytoplasmic | HI | 0 | 0 | ([Banerjee, et al., 2011](#_ENREF_27)) |
| p.(Arg526Cys) | c.1576C>T | Exon 10 | Missense | Cytoplasmic | HI | 5 | 0.0000177 | ([Arya, et al., 2014a](#_ENREF_19); [Salomon-Estebanez, et al., 2016](#_ENREF_328); [Snider, et al., 2013](#_ENREF_356)) |
| p.(Lys528ArgfsTer3) | c.1581del | Exon 10 | Frameshift |  | HI | 0 | 0 | ([Fournet, et al., 2001](#_ENREF_117)) |
| p.(Lys528GlyfsTer4) | c.1580_1581dup | Exon 10 | Frameshift |  | HI | 0 | 0 | ([Faletra, et al., 2013a](#_ENREF_101)) |
| p.(Ser532Gly) | c.1594A>G | Exon 10 | Missense | Cytoplasmic | TNDM | 0 | 0 | ([Demirbilek, et al., 2015](#_ENREF_89)) |
| p.(Leu533Pro) | c.1598T>C | Exon 10 | Missense | Cytoplasmic | HI | 0 | 0 | ([Mohnike, et al., 2014](#_ENREF_256)) |
| p.(Phe536Ser) | c.1607T>C | Exon 10 | Missense | Cytoplasmic | NDM | 0 | 0 | Exeter unpublished |
| p.(Phe536Leu) | c.1608T>G | Exon 10 | Missense | Cytoplasmic | NDM | 0 | 0 | ([Bennett, et al., 2015](#_ENREF_33)) |
| p.(Tyr539Ter) | c.1617T>A | Exon 10 | Nonsense |  | HI | 0 | 0 | ([Faletra, et al., 2013a](#_ENREF_101); [Mohnike, et al., 2014](#_ENREF_256); [Snider, et al., 2013](#_ENREF_356)) |
| p.? | c.1630+1G>T | Intron 10 | Aberrant splicing |  | HI | 6 | 0.00002388 | ([Bellanne-Chantelot, et al., 2010](#_ENREF_31); [Glaser, et al., 1999](#_ENREF_127); [Hardy, et al., 2007](#_ENREF_146); [Nestorowicz, et al., 1998](#_ENREF_265); [Ryan, et al., 1998](#_ENREF_322); [Sandal, et al., 2009](#_ENREF_329)) |
| p.? | c.1630+1G>A | Intron 10 | Aberrant splicing |  | HI | 1 | 0.00000398 | ([Stanley, et al., 2004](#_ENREF_361)) |
| p.? | c.1630+1G>C | Intron 10 | Aberrant splicing |  | HI | 0 | 0 | ([Park, et al., 2011](#_ENREF_288)) |
| p.? | c.1631-2A>C | Intron 10 | Aberrant splicing |  | HI | 0 | 0 | ([Arya, et al., 2014b](#_ENREF_20)) |
| p.? | c.1631-2A>T | Intron 10 | Aberrant splicing |  | HI | 0 | 0 | Paris unpublished |
| p.(Ile544Thr) | c.1631T>C | Exon 11 | Missense | Transmembrane | NDM | 0 | 0 | ([Zhang, et al., 2015a](#_ENREF_419)) |
| p.(Phe545SerfsTer2) | c.1634del | Exon 11 | Frameshift |  | HI | 1 | 0.000006331 | ([Snider, et al., 2013](#_ENREF_356); [Suchi, et al., 2006](#_ENREF_365)) |
| p.(Pro551Arg) | c.1652C>G | Exon 11 | Missense | Transmembrane | HI | 0 | 0 | ([Fernandez-Marmiesse, et al., 2006](#_ENREF_106)) |
| p.? | c.1672-9T>A | Intron 11 | Aberrant splicing |  | HI | 0 | 0 | ([Mohnike, et al., 2014](#_ENREF_256)) |
| p.? | c.1672-20A>G | Intron 11 | Aberrant splicing |  | HI | 1 | 0.000004047 | ([Thomas, et al., 1996b](#_ENREF_388)) |
| p.? | c.1672-20A>T | Intron 11 | Aberrant splicing |  | HI | 0 | 0 | Exeter unpublished |
| p.(His562GlnfsTer58) | c.1683_1687del | Exon 12 | Frameshift |  | HI | 0 | 0 | Exeter unpublished |
| p.(Phe577Leu) | c.1729T>C | Exon 12 | Missense | Extracellular | PNDM | 0 | 0 | ([Katanic, et al., 2017](#_ENREF_193)) |
| p.(Phe577Leu) | c.1731T>G | Exon 12 | Missense | Extracellular | TNDM | 0 | 0 | Exeter unpublished |
| p.(Ala578_Leu582dup) | c.1732_1746dup | Exon 12 | Duplication |  | HI | 3 | 0.00001193 | ([Bellanne-Chantelot, et al., 2010](#_ENREF_31); [Fernandez-Marmiesse, et al., 2006](#_ENREF_106); [Martinez, et al., 2016](#_ENREF_243)) |
| p.(Leu580Phe) | c.1738C>T | Exon 12 | Missense | Extracellular | HI | 0 | 0 | ([Bellanne-Chantelot, et al., 2010](#_ENREF_31)) |
| p.(Ser581Thr) | c.1741T>A | Exon 12 | Exon 12 | Extracellular | HI | 3 | 0.00001193 | ([Han, et al., 2016](#_ENREF_144)) |
| p.(Leu582Val) | c.1744C>G | Exon 12 | Missense | Extracellular | TNDM Later-onset diabetes | 0 | 0 | ([Babenko, et al., 2006](#_ENREF_24); [Riveline, et al., 2012](#_ENREF_317); [Vaxillaire, et al., 2007](#_ENREF_396)) |
| p.(Ile585Thr) | c.1754T>C | Exon 12 | Missense | Transmembrane | TNDM | 0 | 0 | ([Globa, et al., 2015](#_ENREF_128)) |
| p.(Val587Asp) | c.1760T>A | Exon 12 | Missense | Transmembrane | NDM | 0 | 0 | Exeter unpublished |
| p.(Val587Gly) | c.1760T>G | Exon 12 | Missense | Transmembrane | PNDM | 0 | 0 | ([Takeda, et al., 2015](#_ENREF_372)) |
| p.(Thr588Ile) | c.1763C>T | Exon 12 | Missense | Transmembrane | TNDM | 0 | 0 | ([Huopio, et al., 2016](#_ENREF_158)) |
| p.(Phe591Leu) | c.1771T>C | Exon 12 | Missense | Transmembrane | HI | 0 | 0 | ([Demirbilek, et al., 2014](#_ENREF_88)) |
| p.(Phe591Leu) | c.1773C>G | Exon 12 | Missense | Transmembrane | HI | 0 | 0 | ([Nestorowicz, et al., 1998](#_ENREF_265); [Shyng, et al., 1998](#_ENREF_350)) |
| p.(Leu592CysfsTer13) | c.1774del | Exon 12 | Frameshift |  | HI | 0 | 0 | ([Saito-Hakoda, et al., 2012](#_ENREF_326)) |
| p.(Leu593Pro) | c.1778T>C | Exon 12 | Missense | Transmembrane | HI | 0 | 0 | ([Xu, et al., 2018](#_ENREF_409)) |
| p.(Ser594Pro) | c.1780T>C | Exon 12 | Missense | Transmembrane | HI | 0 | 0 | Odense unpublished |
| p.(Arg598Ter) | c.1792C>T | Exon 12 | Nonsense |  | HI | 4 | 0.00001591 | ([Bellanne-Chantelot, et al., 2010](#_ENREF_31); [Damaj, et al., 2008](#_ENREF_75); [De Vroede, et al., 2004](#_ENREF_81); [Del Roio Liberatore, et al., 2015](#_ENREF_86); [Fernandez-Marmiesse, et al., 2006](#_ENREF_106); [Giurgea, et al., 2006](#_ENREF_126); [Gong, et al., 2016](#_ENREF_136); [Henquin, et al., 2011](#_ENREF_151); [Jahnavi, et al., 2014](#_ENREF_175); [Madani, 2019](#_ENREF_231); [Mohnike, et al., 2014](#_ENREF_256); [Sang, et al., 2014a](#_ENREF_331); [Stanley, et al., 2004](#_ENREF_361); [Suchi, et al., 2003](#_ENREF_364)) |
| p.(Arg598Gln) | c.1793G>A | Exon 12 | Missense | Transmembrane | PNDM | 2 | 0.00006373 | ([Martinez, et al., 2016](#_ENREF_243)) |
| p.(Val601Ile) | c.1801G>A | Exon 12 | Missense | Transmembrane | HI | 0 | 0 | ([Arya, et al., 2014b](#_ENREF_20)) |
| p.(Ser606Thr) | c.1817G>C | Exon 12 | Missense | Cytoplasmic | HI | 0 | 0 | ([Kumaran, et al., 2010](#_ENREF_214)) |
| p.(Ser606Asn) | c.1817G>A | Exon 12 | Missense | Cytoplasmic | HI | 0 | 0 | ([Henquin, et al., 2011](#_ENREF_151)) |
| p.? | c.1817+1G>C | Intron 12 | Aberrant splicing |  | HI | 0 | 0 | ([Guven, et al., 2016](#_ENREF_142)) |
| p.? | c.1817+2T>C | Intron 12 | Aberrant splicing |  | HI | 1 | 0.000003977 | ([Li, et al., 2017](#_ENREF_222)) |
| p.(Val607Met) | c.1819G>A | Exon 13 | Missense | Cytoplasmic | PNDM | 2 | 0.000008004 | ([Hashimoto, et al., 2017](#_ENREF_149); [Rubio-Cabezas, et al., 2012](#_ENREF_319)) |
| p.? | c.(1817+1_1818-1)_(1923+1_1923-1)del | Exon 13 | Deletion |  | HI | 0 | 0 | ([Banerjee, et al., 2011](#_ENREF_27); [Flanagan, et al., 2012](#_ENREF_107)) |
| p.(Gln608Ter) | c.1822C>T | Exon 13 | Nonsense |  | HI | 0 | 0 | ([Ni, et al., 2019](#_ENREF_268)) |
| p.(Lys609ArgfsTer2) | c.1826_1828delinsGG | Exon 13 | Frameshift |  | HI | 0 | 0 | Paris unpublished |
| p.(Leu610Arg) | c.1829T>G | Exon 13 | Missense | Cytoplasmic | HI | 0 | 0 | ([Martinez, et al., 2016](#_ENREF_243)) |
| p.(Glu612Asp) | c.1836G>T | Exon 13 | Missense | Cytoplasmic | HI | 2 | 0.000007974 | Odense unpublished |
| p.(His627MetfsTer20) | c.1879del | Exon 13 | Frameshift |  | HI | 0 | 0 | ([Arya, et al., 2014a](#_ENREF_19); [Arya, et al., 2014b](#_ENREF_20); [Han, et al., 2016](#_ENREF_144); [Mohnike, et al., 2014](#_ENREF_256); [Stanley, et al., 2004](#_ENREF_361); [Suchi, et al., 2003](#_ENREF_364); [Suchi, et al., 2006](#_ENREF_365)) |
| p.(Thr630HisfsTer17) | c.1887del | Exon 13 | Frameshift |  | HI | 0 | 0 | ([Aguilar-Bryan and Bryan, 1999](#_ENREF_7); [Sang, et al., 2014a](#_ENREF_331); [Xu, et al., 2013](#_ENREF_408)) |
| p.(Pro631LeufsTer16) | c.1892del | Exon 13 | Frameshift |  | HI |  |  | ([Ni, et al., 2019](#_ENREF_268)) |
| p.(Gln632ArgfsTer15) | c.1893del | Exon 13 | Frameshift |  | HI | 0 | 0 | ([Nestorowicz, et al., 1998](#_ENREF_265)) |
| p.(Ala640Val) | c.1919C>T | Exon 13 | Missense | Cytoplasmic | HI | 32 | 0.0001133 | ([Sang, et al., 2014a](#_ENREF_331)) |
| p.? | c.1923+5G>T | Intron 13 | Aberrant splicing |  | HI | 0 | 0 | ([Bellanne-Chantelot, et al., 2010](#_ENREF_31)) |
| p.? | c.1924-1G>T | Intron 13 | Aberrant splicing |  | HI | 1 | 0.000003997 | ([Craigie, et al., 2018](#_ENREF_72)) |
| p.? | c.1924-2A>T | Intron 13 | Aberrant splicing |  | HI | 0 | 0 | Odense unpublished |
| p.(Leu643ProfsTer33) | c.1927dup | Exon 14 | Frameshift |  | HI | 0 | 0 | ([Snider, et al., 2013](#_ENREF_356)) |
| p.(Glu654Ter) | c.1960G>T | Exon 14 | Nonsense |  | HI | 0 | 0 | Exeter unpublished |
| p.(Arg657Gln) | c.1970G>A | Exon 14 | Missense | Cytoplasmic | HI | 4 | 0.00001416 | ([Rozenkova, et al., 2015](#_ENREF_318)) |
| p.(Gln664Ter) | c.1990C>T | Exon 14 | Nonsense |  | HI | 0 | 0 | ([Gong, et al., 2016](#_ENREF_136)) |
| p.(Val679LeufsTer68) | c.2031_2034dup | Exon 14 | Frameshift |  | HI | 0 | 0 | ([Bellanne-Chantelot, et al., 2010](#_ENREF_31)) |
| p.? | c.2041-21G>A | Intron 14 | Aberrant splicing |  | HI | 7 | 0.00002537 | ([Hardy, et al., 2007](#_ENREF_146); [Lee, et al., 2015](#_ENREF_219); [Mohnike, et al., 2014](#_ENREF_256); [Ohkubo, et al., 2005](#_ENREF_275); [Salomon-Estebanez, et al., 2016](#_ENREF_328); [Suchi, et al., 2006](#_ENREF_365)) |
| p.? | c.2041-25G>A | Intron 14 | Aberrant splicing |  | HI | 0 | 0 | ([Bas, et al., 2012](#_ENREF_28)) |
| p.? | c.2041-12C>A | Intron 14 | Aberrant splicing |  | HI | 3 | 0.00001075 | ([Snider, et al., 2013](#_ENREF_356)) |
| p.? | c.2041-2A>G | Intron 14 | Aberrant splicing |  | HI | 0 | 0 | Exeter unpublished |
| p.? | c.2041-1G>A | Intron 14 | Aberrant splicing |  | HI | 0 | 0 | Odense unpublished |
| p.? | c.2041-1G>C | Intron 14 | Aberrant splicing |  | HI | 0 | 0 | ([Kiff, et al., 2019](#_ENREF_199)) |
| p.(Gly684Asp) | c.2051G>A | Exon 15 | Missense | Cytoplasmic | HI | 0 | 0 | ([Bellanne-Chantelot, et al., 2010](#_ENREF_31)) |
| p.(Phe686Ser) | c.2057T>C | Exon 15 | Missense | Cytoplasmic | HI | 0 | 0 | ([Stanley, et al., 2004](#_ENREF_361); [Suchi, et al., 2006](#_ENREF_365); [Yan, et al., 2007](#_ENREF_413)) |
| p.(Trp688Arg) | c.2062T>C | Exon 15 | Missense | Cytoplasmic | PNDM | 0 | 0 | ([Flanagan, et al., 2014](#_ENREF_108); [Russo, et al., 2011b](#_ENREF_321)) |
| p.(Trp688Ter) | c.2064G>A | Exon 15 | Nonsense |  | HI | 0 | 0 | ([Bellanne-Chantelot, et al., 2010](#_ENREF_31); [Giurgea, et al., 2004](#_ENREF_125); [Henquin, et al., 2011](#_ENREF_151)) |
| p.(Leu696Pro) | c.2087T>C | Exon 15 | Missense | Cytoplasmic | HI | 0 | 0 | ([Snider, et al., 2013](#_ENREF_356)) |
| p.(Ile703ThrfsTer6) | c.2108del | Exon 15 | Frameshift |  | HI | 0 | 0 | ([Snider, et al., 2013](#_ENREF_356)) |
| p.(Arg705Ter) | c.2113C>T | Exon 15 | Nonsense |  | HI | 1 | 0.000003989 | Exeter unpublished |
| p.? | c.2116+1G>C | Intron 15 | Aberrant splicing |  | HI | 0 | 0 | ([Han, et al., 2016](#_ENREF_144)) |
| p.? | c.2116+1G>T | Intron 15 | Aberrant splicing |  | HI | 0 | 0 | ([Suchi, et al., 2003](#_ENREF_364); [Suchi, et al., 2006](#_ENREF_365)) |
| p.? | c.2116+2T>C | Intron 15 | Aberrant splicing |  | HI | 2 | 0.000007979 | ([Bellanne-Chantelot, et al., 2010](#_ENREF_31)) |
| p.? | c.2117-2A>T | Intron 15 | Aberrant splicing |  | HI | 1 | 0.00000402 | ([Mohnike, et al., 2014](#_ENREF_256)) |
| p.? | c.2117-1G>A | Intron 15 | Aberrant splicing |  | HI | 0 | 0 | ([Nestorowicz, et al., 1998](#_ENREF_265)) |
| p.(Gln707HisfsTer2) | c.2121del | Exon 16 | Frameshift |  | HI | 0 | 0 | ([Snider, et al., 2013](#_ENREF_356)) |
| p.(Leu708_Met730del) | c.2123_2191del | Exon 16 | In frame deletion | Cytoplasmic | HI | 0 | 0 | ([Mohnike, et al., 2014](#_ENREF_256)) |
| p.(Thr709Ter) | c.2124_2127del | Exon 16 | Nonsense |  | HI | 0 | 0 | ([Bellanne-Chantelot, et al., 2010](#_ENREF_31)) |
| p.(Gly713Arg) | c.2137G>C | Exon 16 | Missense | NBD1 | HI | 0 | 0 | Exeter unpublished |
| p.(Gln714AlafsTer32) | c.2139dup | Exon 16 | Frameshift |  | HI | 0 | 0 | ([Snider, et al., 2013](#_ENREF_356)) |
| p.(Gln714Ter) | c.2140C>T | Exon 16 | Nonsense |  | HI | 0 | 0 | ([Bendix, et al., 2018](#_ENREF_32)) |
| p.(Val715TrpfsTer10) | c.2143del | Exon 16 | Frameshift |  | HI | 0 | 0 | ([Fernandez-Marmiesse, et al., 2006](#_ENREF_106)) |
| p.(Val715Met) | c.2143G>A | Exon 16 | Missense | NBD1 | HI | 0 | 0 | ([Boodhansingh, et al., 2019](#_ENREF_40)) |
| p.(Val715Ala) | c.2144T>C | Exon 16 | Missense | NBD1 | HI | 0 | 0 | ([Macmullen, et al., 2011](#_ENREF_230)) |
| p.(Val715Gly) | c.2144T>G | Exon 16 | Missense | NBD1 | HI | 0 | 0 | ([Macmullen, et al., 2011](#_ENREF_230)) |
| p.(Gly716Cys) | c.2146G>T | Exon 16 | Missense | NBD1 | HI | 0 | 0 | ([Mohnike, et al., 2014](#_ENREF_256)) |
| p.(Gly716Ser) | c.2146G>A | Exon 16 | Missense | NBD1 | HI | 0 | 0 | ([Faletra, et al., 2013a](#_ENREF_101)) |
| p.(Gly716Asp) | c.2147G>A | Exon 16 | Missense | NBD1 | HI | 0 | 0 | ([Bellanne-Chantelot, et al., 2010](#_ENREF_31); [Macmullen, et al., 2011](#_ENREF_230)) |
| p.(Gly716Val) | c.2147G>T | Exon 16 | Missense | NBD1 | HI | 0 | 0 | ([Thomas, et al., 1996b](#_ENREF_388); [Yan, et al., 2007](#_ENREF_413)) |
| p.(Cys717Ter) | c.2151C>A | Exon 16 | Nonsense |  | HI | 1 | 0.000003988 | ([Stanley, et al., 2004](#_ENREF_361); [Suchi, et al., 2003](#_ENREF_364); [Suchi, et al., 2006](#_ENREF_365)) |
| p.(Gly718AlafsTer7) | c.2153del | Exon 16 | Frameshift |  | HI | 0 | 0 | ([Bellanne-Chantelot, et al., 2010](#_ENREF_31)) |
| p.(Lys719Thr) | c.2156A>C | Exon 16 | Missense | NBD1 | HI | 0 | 0 | ([Fernandez-Marmiesse, et al., 2006](#_ENREF_106)) |
| p.(Ser720Phe) | c.2159C>T | Exon 16 | Missense | NBD1 | HI | 0 | 0 | ([Snider, et al., 2013](#_ENREF_356)) |
| p.(Leu724del) | c.2167_2169del | Exon 16 | In frame deletion | Cytoplasmic | HI | 2 | 0.000007959 | ([Snider, et al., 2013](#_ENREF_356)) |
| p.(Leu724Pro) | c.2171T>C | Exon 16 | Missense | Cytoplasmic | HI | 1 | 0.00000398 | ([Jahnavi, et al., 2014](#_ENREF_175)) |
| p.(Leu724del) | c.2169_2171del | Exon 16 | In frame deletion | Cytoplasmic | HI | 2 | 0.000007959 | ([Mohnike, et al., 2014](#_ENREF_256)) |
| p.(Leu727Arg) | c.2180T>G | Exon 16 | Missense | Cytoplasmic | HI | 0 | 0 | ([Mohnike, et al., 2014](#_ENREF_256)) |
| p.(Glu729Ter) | c.2185G>T | Exon 16 | Nonsense |  | HI | 0 | 0 | Exeter unpublished |
| p.(Ala736LeufsTer13) | c.2202del | Exon 16 | Frameshift |  | HI | 0 | 0 | ([Kapoor, et al., 2013](#_ENREF_188)) |
| p.? | c.2222+1G>A | Intron 16 | Aberrant splicing |  | HI | 0 | 0 | Exeter unpublished |
| p.? | c.2222+1G>T | Intron 16 | Aberrant splicing |  | HI | 0 | 0 | ([Snider, et al., 2013](#_ENREF_356)) |
| p.? | c.2222+3A>G | Intron 16 | Aberrant splicing |  | HI | 0 | 0 | ([Straub, et al., 2001](#_ENREF_363); [Tornovsky, et al., 2004](#_ENREF_392)) |
| p.(Glu747Ter) | c.2239G>T | Exon 17 | Nonsense |  | PNDM | 0 | 0 | ([Flanagan, et al., 2017](#_ENREF_109)) |
| p.? | c.(2258+1_2259-1)_(2294+1_2295-1)del | Exon 18 | Deletion |  | HI | 0 | 0 | ([Salomon-Estebanez, et al., 2016](#_ENREF_328)) |
| p.(Glu757Ter) | c.2269G>T | Exon 18 | Nonsense |  | HI | 0 | 0 | Exeter unpublished |
| p.? | c.2295-1G>A | Intron 18 | Aberrant splicing |  | HI | 0 | 0 | ([Thomas, et al., 1996b](#_ENREF_388)) |
| p.(Arg767SerfsTer21) | c.2298_2310delinsAA | Exon 19 | Frameshift |  | HI | 0 | 0 | Chicago unpublished |
| p.(Gly768ProfsTer23) | c.2301_2302del | Exon 19 | Frameshift |  | HI | 0 | 0 | Exeter unpublished |
| p.(Trp778Ter) | c.2334G>A | Exon 19 | Nonsense |  | HI | 0 | 0 | ([Xu, et al., 2018](#_ENREF_409)) |
| p.(Glu786del) | c.2356_2358del | Exon 19 | In frame deletion | Cytoplasmic | HI | 1 | 0.000003976 | ([Snider, et al., 2013](#_ENREF_356)) |
| p.(Ile788Asn) | c.2363T>A | Exon 19 | Missense | Cytoplasmic | HI | 0 | 0 | ([Snider, et al., 2013](#_ENREF_356)) |
| p.(Glu791Ter) | c.2371G>T | Exon 19 | Nonsense |  | HI | 0 | 0 | ([Simsek, et al., 2013](#_ENREF_352)) |
| p.(Phe794SerfsTer71) | c.2379del | Exon 19 | Frameshift |  | HI | 0 | 0 | Paris unpublished |
| p.(Arg798Trp) | c.2392C>T | Exon 19 | Missense | Cytoplasmic | HI | 13 | 0.00004596 | ([Snider, et al., 2013](#_ENREF_356)) |
| p.? | c.2394-1G>A | Exon 19 | Aberrant splicing |  | HI | 0 | 0 | ([Mohnike, et al., 2014](#_ENREF_256)) |
| p.? | c.2394-2A>G | Intron 19 | Aberrant splicing |  | HI | 0 | 0 | ([Fernandez-Marmiesse, et al., 2006](#_ENREF_106)) |
| p.(Tyr799Ter) | c.2397del | Exon 20 | Nonsense |  | HI | 0 | 0 | Paris unpublished |
| p.(Cys806Tyr) | c.2417G>A | Exon 20 | Missense | Cytoplasmic | HI | 0 | 0 | Exeter unpublished |
| p.(Gln809Lys) | c.2425C>A | Exon 20 | Missense | Cytoplasmic | Diabetes | 15 | 0.00005303 | ([Bennett, et al., 2015](#_ENREF_33)) |
| p.(Gln809Ter) | c.2425C>T | Exon 20 | Nonsense |  | HI | 0 | 0 | ([Bellanne-Chantelot, et al., 2010](#_ENREF_31); [Damaj, et al., 2008](#_ENREF_75)) |
| p.(Asp811Val) | c.2432A>T | Exon 20 | Missense | Cytoplasmic | TNDM | 0 | 0 | Chicago unpublished |
| p.(Asp813Asn) | c.2437G>A | Exon 20 | Missense | Cytoplasmic | HI | 20 | 0.00007953 | ([Park, et al., 2011](#_ENREF_288)) |
| p.(His817Arg) | c.2450A>G | Exon 20 | Missense | Cytoplasmic | PNDM  Later-onset diabetes* | 5 | 0.00001768 | ([Rubio-Cabezas, et al., 2012](#_ENREF_319))  Paris unpublished* |
| p.(Gln822Ter) | c.2464C>T | Exon 20 | Nonsense |  | HI | 0 | 0 | ([Kapoor, et al., 2013](#_ENREF_188)) |
| p.(Glu825Lys) | c.2473G>A | Exon 20 | Missense | Cytoplasmic | HI | 0 | 0 | ([Bellanne-Chantelot, et al., 2010](#_ENREF_31)) |
| p.(Glu825Ter) | c.2473G>T | Exon 20 | Nonsense |  | HI | 0 | 0 | ([Snider, et al., 2013](#_ENREF_356)) |
| p.(Arg826Trp) | c.2476C>T | Exon 20 | Missense | Cytoplasmic | TNDM PNDM  Later-onset diabetes* | 1 | 0.000003976 | ([Abujbara, et al., 2014](#_ENREF_5); [Busiah, et al., 2014](#_ENREF_49); [Cao, et al., 2016](#_ENREF_53); [de Wet, et al., 2008](#_ENREF_83); [Flanagan, et al., 2007](#_ENREF_113); [Huopio, et al., 2016](#_ENREF_158); [Klupa, et al., 2009](#_ENREF_203); [Ozsu, et al., 2016](#_ENREF_285); [Patch, et al., 2007](#_ENREF_290); [Piccini, et al., 2018](#_ENREF_297); [Riveline, et al., 2012](#_ENREF_317); [Taberner, et al., 2016](#_ENREF_369); [Vaxillaire, et al., 2007](#_ENREF_396))  Paris unpublished* |
| p.? | c.2478+1G>A | Intron 20 | Aberrant splicing |  | HI | 0 | 0 | ([Yen, et al., 2016](#_ENREF_415)) |
| p.? | c.2479-1G>A | Intron 20 | Aberrant splicing |  | HI | 0 | 0 | Exeter unpublished |
| p.(Gly827AlafsTer38) | c.2480del | Exon 21 | Frameshift |  | HI | 0 | 0 | Paris unpublished |
| p.(Gly832Cys) | c.2494G>T | Exon 21 | Missense | Cytoplasmic | TNDM | 0 | 0 | ([Hashimoto, et al., 2017](#_ENREF_149); [Yamazaki, et al., 2017](#_ENREF_411)) |
| p.(Gly833Asp) | c.2498G>A | Exon 21 | Missense | Cytoplasmic | NDM | 0 | 0 | ([Zhang, et al., 2015a](#_ENREF_419)) |
| p.(Gln834Ter) | c.2500C>T | Exon 21 | Nonsense |  | HI | 0 | 0 | ([Dalvi, et al., 2017](#_ENREF_74)) |
| p.(Arg837Ter) | c.2509C>T | Exon 21 | Nonsense |  | HI | 17 | 0.00006112 | ([Aguilar-Bryan and Bryan, 1999](#_ENREF_7); [Bennett, et al., 2015](#_ENREF_33); [Craigie, et al., 2018](#_ENREF_72); [Fan, et al., 2015](#_ENREF_103); [Greer, et al., 2007](#_ENREF_138); [Hardy, et al., 2007](#_ENREF_146); [Hashimoto, et al., 2015](#_ENREF_150); [Lee, et al., 2015](#_ENREF_219); [Moreira, et al., 2013](#_ENREF_257); [Park, et al., 2011](#_ENREF_288); [Tornovsky, et al., 2004](#_ENREF_392); [Yorifuji, et al., 2011](#_ENREF_417)) |
| p.(Val840Glu) | c.2519T>A | Exon 21 | Missense | Cytoplasmic | HI | 0 | 0 | ([Snider, et al., 2013](#_ENREF_356)) |
| p.(Arg842Gly) | c.2524C>G | Exon 21 | Missense | Cytoplasmic | HI | 0 | 0 | ([de Lonlay-Debeney, et al., 1999](#_ENREF_79)) |
| p.(Arg842Ter) | c.2524C>T | Exon 21 | Nonsense |  | HI | 2 | 0.000008082 | ([Brunetti-Pierri, et al., 2008](#_ENREF_47); [Mohnike, et al., 2014](#_ENREF_256); [Salomon-Estebanez, et al., 2016](#_ENREF_328)) |
| p.(Arg842GlufsTer23) | c.2524del | Exon 21 | Frameshift |  | HI | 0 | 0 | Exeter unpublished |
| p.(Arg842Gln) | c.2525G>A | Exon 21 | Missense | Cytoplasmic | HI | 0 | 0 | ([Banerjee, et al., 2011](#_ENREF_27)) |
| p.(Arg842Pro) | c.2525G>C | Exon 21 | Missense | Cytoplasmic | HI | 0 | 0 | Odense unpublished |
| p.(Ala848Thr) | c.2542G>A | Exon 21 | Missense | Cytoplasmic | HI | 5 | 0.00002028 | ([Jahnavi, et al., 2014](#_ENREF_175)) |
| p.? | c.2559+3_2559+15delinsCCTGGGGTCCTTGT | Intron 21 | Aberrant splicing |  | HI | 0 | 0 | Paris unpublished |
| p.? | c.2560-1G>A | Intron 21 | Aberrant splicing |  | HI | 0 | 0 | Exeter unpublished |
| p.? | c.2560-1G>C | Intron 21 | Aberrant splicing |  | HI | 0 | 0 | ([Fan, et al., 2015](#_ENREF_103)) |
| p.? | c.(2559+1_2560-1)_(2697+1_2698-1)del | Exon 22 | In frame deletion | Cytoplasmic | HI | 0 | 0 | ([Bellanne-Chantelot, et al., 2010](#_ENREF_31); [Flanagan, et al., 2012](#_ENREF_107)) |
| p.? | c.(2559+1_2560-1)_(3332+1_3333-1)del | Exons 22-26 | Deletion |  | HI | 0 | 0 | Exeter unpublished |
| p.(Asp855Glu) | c.2565C>A | Exon 22 | Missense | Cytoplasmic | HI | 0 | 0 | ([Arya, et al., 2014b](#_ENREF_20)) |
| p.(Asp861His) | c.2581G>C | Exon 22 | Missense | Cytoplasmic | HI | 0 | 0 | ([Bellanne-Chantelot, et al., 2010](#_ENREF_31)) |
| p.(His863Tyr) | c.2587C>T | Exon 22 | Missense | Cytoplasmic | NDM,  Later-onset diabetes | 0 | 0 | ([Klee, et al., 2012](#_ENREF_201)) |
| p.(His863ArgfsTer5) | c.2588_2591del | Exon 22 | Frameshift |  | HI | 0 | 0 | ([Henquin, et al., 2011](#_ENREF_151)) |
| p.(Thr888Pro) | c.2662A>C | Exon 22 | Missense | Cytoplasmic | HI | 0 | 0 | ([Macmullen, et al., 2011](#_ENREF_230)) |
| p.(Lys890Thr) | c.2669A>C | Exon 22 | Missense | Cytoplasmic | HI | 5 | 0.00001768 | ([Aguilar-Bryan and Bryan, 1999](#_ENREF_7); [Bellanne-Chantelot, et al., 2010](#_ENREF_31); [Giurgea, et al., 2004](#_ENREF_125)) |
| p.(Lys890SerfsTer12) | c.2669_2675del | Exon 22 | Frameshift |  | HI | 0 | 0 | ([Arya, et al., 2014b](#_ENREF_20)) |
| p.(Leu891Pro) | c.2672T>C | Exon 22 | Missense | Cytoplasmic | HI | 0 | 0 | ([Bellanne-Chantelot, et al., 2010](#_ENREF_31)) |
| p.(Gln892Ter) | c.2674C>T | Exon 22 | Nonsense |  | HI | 0 | 0 | Exeter unpublished |
| p.(Gln892ProfsTer28) | c.2675_2679del | Exon 22 | Frameshift |  | HI | 0 | 0 | Exeter unpublished |
| p.(Asp898Val) | c.2693A>T | Exon 22 | Missense | Cytoplasmic | HI | 1 | 0.000003978 | ([Ni, et al., 2019](#_ENREF_268)) |
| p.(Trp899GlyfsTer5) | c.2694del | Exon 22 | Frameshift |  | HI | 0 | 0 | ([Ni, et al., 2019](#_ENREF_268)) |
| p.(Trp899Ter) | c.2696G>A | Exon 22 | Nonsense |  | HI | 1 | 0.000003979 | ([Snider, et al., 2013](#_ENREF_356)) |
| p.(Trp899Ter) | c.2697G>A | Exon 22 | Nonsense |  | HI | 0 | 0 | ([Jahnavi, et al., 2014](#_ENREF_175)) |
| p.? | c.2697+1G>A | Intron 22 | Aberrant splicing |  | HI | 1 | 0.000003979 | ([Snider, et al., 2013](#_ENREF_356)) |
| p.? | c.2697+3G>C | Intron 22 | Aberrant splicing |  | HI | 0 | 0 | ([Aguilar-Bryan and Bryan, 1999](#_ENREF_7)) |
| p.? | c.2697+4A>T | Intron 22 | Aberrant splicing |  | HI | 0 | 0 | ([Arya, et al., 2014b](#_ENREF_20)) |
| p.? | c.2697+5G>A | Intron 22 | Aberrant splicing |  | HI | 0 | 0 | ([Senniappan, et al., 2015](#_ENREF_335)) |
| p.? | c.2698-2A>T | Intron 22 | Aberrant splicing |  | HI | 0 | 0 | ([Snider, et al., 2013](#_ENREF_356)) |
| p.? | c.2698-2A>G | Intron 22 | Aberrant splicing |  | HI | 0 | 0 | ([Mohnike, et al., 2014](#_ENREF_256)) |
| p.? | c.2698-1G>C | Intron 22 | Aberrant splicing |  | HI | 0 | 0 | ([Snider, et al., 2013](#_ENREF_356)) |
| p.(Ile901Phe) | c.2701A>T | Exon 23 | Missense | Cytoplasmic | HI | 0 | 0 | ([Martinez, et al., 2016](#_ENREF_243)) |
| p.(Ile901Thr) | c.2702T>C | Exon 23 | Missense | Cytoplasmic | HI | 1 | 0.000004023 | ([Bellanne-Chantelot, et al., 2010](#_ENREF_31); [Snider, et al., 2013](#_ENREF_356)) |
| p.(Gly912Arg) | c.2734G>C | Exon 23 | Missense | Cytoplasmic | HI | 0 | 0 | Paris unpublished |
| p.(Gln918Ter) | c.2752C>T | Exon 23 | Nonsense |  | HI | 0 | 0 | ([Sandal, et al., 2009](#_ENREF_329)) |
| p.(Gln923Ter) | c.2767C>T | Exon 23 | Missense | Cytoplasmic | HI | 0 | 0 | ([Bendix, et al., 2018](#_ENREF_32); [Park, et al., 2011](#_ENREF_288)) |
| p.(Trp928Ter) | c.2784G>A | Exon 23 | Nonsense |  | HI | 0 | 0 | ([Bellanne-Chantelot, et al., 2010](#_ENREF_31); [Faletra, et al., 2013a](#_ENREF_101); [Giurgea, et al., 2004](#_ENREF_125)) |
| p.(Arg934Ter) | c.2800C>T | Exon 23 | Nonsense |  | HI | 4 | 0.00001415 | ([Arya, et al., 2014b](#_ENREF_20); [Fan, et al., 2015](#_ENREF_103); [Fernandez-Marmiesse, et al., 2006](#_ENREF_106); [Gussinyer, et al., 2008](#_ENREF_141); [Kalish, et al., 2016](#_ENREF_186); [Martinez, et al., 2016](#_ENREF_243); [Saint-Martin, et al., 2015](#_ENREF_325)) |
| p.(Gln935Ter) | c.2803C>T | Exon 23 | Nonsense |  | HI | 0 | 0 | ([Bellanne-Chantelot, et al., 2010](#_ENREF_31); [Zhang, et al., 2015b](#_ENREF_420)) |
| p.(Leu939TrpfsTer104) | c.2815del | Exon 23 | Frameshift |  | HI | 0 | 0 | Exeter unpublished |
| p.? | c.2823+1G>A | Intron 23 | Aberrant splicing |  | HI | 0 | 0 | Exeter unpublished |
| p.(Glu946Ter) | c.2836G>T | Exon 24 | Nonsense |  | HI | 0 | 0 | ([Greer, et al., 2007](#_ENREF_138)) |
| p.(Arg947LysfsTer95) | c.2838_2841del | Exon 24 | Frameshift |  | HI | 0 | 0 | ([Martinez, et al., 2016](#_ENREF_243); [Stanley, et al., 2004](#_ENREF_361)) |
| p.(Gln954Ter) | c.2860C>T | Exon 24 | Nonsense |  | HI | 4 | 0.0000159 | ([Arya, et al., 2014b](#_ENREF_20); [Bellanne-Chantelot, et al., 2010](#_ENREF_31); [Faletra, et al., 2013a](#_ENREF_101); [Mohnike, et al., 2014](#_ENREF_256); [Nestorowicz, et al., 1998](#_ENREF_265); [Stanley, et al., 2004](#_ENREF_361); [Suchi, et al., 2003](#_ENREF_364); [Suchi, et al., 2006](#_ENREF_365)) |
| p.(Gln954ArgfsTer89) | c.2860del | Exon 24 | Frameshift |  | HI | 0 | 0 | ([Fan, et al., 2015](#_ENREF_103)) |
| p.(Ser957LeufsTer86) | c.2869del | Exon 24 | Frameshift |  | HI | 0 | 0 | ([Bendix, et al., 2018](#_ENREF_32)) |
| p.(Ser957Phe) | c.2870C>T | Exon 24 | Missense | Cytoplasmic | HI | 0 | 0 | ([Aguilar-Bryan and Bryan, 1999](#_ENREF_7)) |
| p.(Glu973ArgfsTer70) | c.2917del | Exon 24 | Frameshift |  | HI | 0 | 0 | Exeter unpublished |
| p.(Glu974Gly) | c.2921A>G | Exon 24 | Missense | Cytoplasmic | HI | 0 | 0 | Paris unpublished |
| p.? | c.2924-10C>A | Intron 24 | Aberrant splicing |  | HI | 0 | 0 | ([Stanley, et al., 2004](#_ENREF_361); [Suchi, et al., 2006](#_ENREF_365)) |
| p.? | c.2924-9G>A | Intron 24 | Aberrant splicing |  | HI | 6 | 0.00002538 | ([Bellanne-Chantelot, et al., 2010](#_ENREF_31); [Snider, et al., 2013](#_ENREF_356)) |
| p.? | c.2924-2A>G | Intron 24 | Aberrant splicing |  | HI | 0 | 0 | ([Bellanne-Chantelot, et al., 2010](#_ENREF_31); [Bendix, et al., 2018](#_ENREF_32)) |
| p.? | c.2924-1G>A | Intron 24 | Aberrant splicing |  | HI | 1 | 0.000004162 | Exeter unpublished |
| p.(Arg993Cys) | c.2977C>T | Exon 25 | Missense | Cytoplasmic | TNDM | 0 | 0 | ([Mukherjee, et al., 2017](#_ENREF_259)) |
| p.(Glu995Ter) | c.2983G>T | Exon 25 | Nonsense |  | HI | 1 | 0.000004038 | ([Arya, et al., 2014b](#_ENREF_20)) |
| p.(Trp998Ter) | c.2994G>A | Exon 25 | Nonsense |  | HI | 0 | 0 | ([Bellanne-Chantelot, et al., 2010](#_ENREF_31); [Fan, et al., 2015](#_ENREF_103)) |
| p.(Arg999Ter) | c.2995C>T | Exon 25 | Nonsense |  | HI | 3 | 0.0000121 | ([Han, et al., 2016](#_ENREF_144); [Hardy, et al., 2007](#_ENREF_146); [Peranteau, et al., 2007](#_ENREF_293); [Stanley, et al., 2004](#_ENREF_361); [Suchi, et al., 2003](#_ENREF_364); [Suchi, et al., 2006](#_ENREF_365); [Yorifuji, et al., 2011](#_ENREF_417)) |
| p.(Cys1001Ter) | c.3003C>A | Exon 25 | Nonsense |  | HI | 1 | 0.000004027 | ([Sang, et al., 2014a](#_ENREF_331)) |
| p.(Gln1020Ter) | c.3058C>T | Exon 25 | Nonsense |  | HI | 0 | 0 | ([Abraham, et al., 2015](#_ENREF_3)) |
| p.(His1024Tyr) | c.3070C>T | Exon 25 | Missense | Transmembrane | TNDM | 0 | 0 | ([Babenko, et al., 2006](#_ENREF_24)) |
| p.(His1024Arg) | c.3071A>G | Exon 25 | Missense | Transmembrane | PNDM | 0 | 0 | ([Mak, et al., 2012](#_ENREF_235)) |
| p.(Ala1029ProfsTer14) | c.3085del | Exon 25 | Frameshift |  | HI | 0 | 0 | ([Suchi, et al., 2006](#_ENREF_365)) |
| p.(Asp1031Asn) | c.3091G>A | Exon 25 | Missense | Extracellular | HI | 0 | 0 | ([Arya, et al., 2014b](#_ENREF_20)) |
| p.(Trp1037Ter) | c.3111G>A | Exon 25 | Nonsense |  | HI | 0 | 0 | ([Bellanne-Chantelot, et al., 2010](#_ENREF_31); [Snider, et al., 2013](#_ENREF_356)) |
| p.(Thr1043GlnfsTer75) | c.3127_3129delinsCAGCCAGGACCTG | Exon 25 | Frameshift |  | HI PNDM | 0 | 0 | ([Ellard, et al., 2007](#_ENREF_98); [Fan, et al., 2015](#_ENREF_103); [Ni, et al., 2019](#_ENREF_268); [Shah, et al., 2012a](#_ENREF_336)) |
| p.(Thr1045LeufsTer63) | c.3133_3152del | Exon 25 | Frameshift |  | HI | 2 | 0.000007972 | ([Bellanne-Chantelot, et al., 2010](#_ENREF_31); [Fernandez-Marmiesse, et al., 2006](#_ENREF_106); [Fournet, et al., 2001](#_ENREF_117); [Greer, et al., 2007](#_ENREF_138); [Mohnike, et al., 2014](#_ENREF_256); [Snider, et al., 2013](#_ENREF_356)) |
| p.? | c.3165+1G>A | Intron 25 | Aberrant splicing |  | HI | 0 | 0 | ([Fournet, et al., 2001](#_ENREF_117); [Mohnike, et al., 2014](#_ENREF_256)) |
| p.? | c.3165+2T>A | Intron 25 | Aberrant splicing |  | HI | 0 | 0 | Exeter unpublished |
| p.? | c.3166-1G>A | Intron 25 | Aberrant splicing |  | HI | 1 | 0.000003977 | Exeter unpublished |
| p.(Gln1061Ter) | c.3181C>T | Exon 26 | Nonsense |  | HI | 0 | 0 | Exeter unpublished |
| p.(Cys1079Ter) | c.3237C>A | Exon 26 | Nonsense |  | HI | 0 | 0 | Exeter unpublished |
| p.(Glu1087_Ala1094delinsAspLysSerAspThr) | N/A | Exon 26 | In frame indel | Transmembrane | HI | N/A | N/A | ([Park, et al., 2011](#_ENREF_288)) |
| p.(His1098ProfsTer16) | c.3291_3292del | Exon 26 | Frameshift |  | HI | 0 | 0 | ([Fernandez-Marmiesse, et al., 2006](#_ENREF_106)) |
| p.(His1098Arg) | c.3293A>G | Exon 26 | Missense | Cytoplasmic | HI | 0 | 0 | Exeter unpublished |
| p.(Met1110HisfsTer5) | c.3327dup | Exon 26 | Frameshift |  | HI | 0 | 0 | Odense unpublished |
| p.? | c.3332+2T>C | Intron 26 | Aberrant splicing |  | HI | 0 | 0 | ([Snider, et al., 2013](#_ENREF_356)) |
| p.(Glu1114Ter) | c.3339dup | Exon 27 | Nonsense |  | HI | 0 | 0 | ([Tornovsky, et al., 2004](#_ENREF_392)) |
| p.(Asn1123Asp) | c.3367A>G | Exon 27 | Missense | Cytoplasmic | PNDM | 0 | 0 | ([Suzuki, et al., 2007](#_ENREF_367)) |
| p.(Thr1131Pro) | c.3391A>C | Exon 27 | Missense | Cytoplasmic | HI | 0 | 0 | ([Bellanne-Chantelot, et al., 2010](#_ENREF_31); [Fernandez-Marmiesse, et al., 2006](#_ENREF_106); [Giurgea, et al., 2004](#_ENREF_125)) |
| p.(Gln1134Ter) | c.3400C>T | Exon 27 | Nonsense |  | HI | 0 | 0 | Exeter unpublished |
| p.(Gln1134Arg) | c.3401A>G | Exon 27 | Missense | Cytoplasmic | HI | 3 | 0.00001193 | Odense unpublished |
| p.(Gln1134His) | c.3402G>T | Exon 27 | Missense | Cytoplasmic | HI | 0 | 0 | ([Park, et al., 2011](#_ENREF_288)) |
| p.? | c.3402+2T>C | Intron 27 | Aberrant splicing |  | HI | 0 | 0 | ([Mohnike, et al., 2014](#_ENREF_256)) |
| p.? | c.3403-1G>A | Intron 27 | Aberrant splicing |  | HI | 1 | 0.000004253 | ([Snider, et al., 2013](#_ENREF_356)) |
| p.? | c.(3402+1_3403-1)_(3653+1_3654-1)del | Exons 28-29 | Deletion |  | HI | 0 | 0 | Exeter unpublished |
| p.(Thr1139HisfsTer7) | c.3410_3414dup | Exon 28 | Frameshift |  | HI | 0 | 0 | Exeter unpublished |
| p.(Glu1141Ter) | c.3421G>T | Exon 28 | Nonsense |  | HI | 0 | 0 | Exeter unpublished |
| p.(Glu1141Gly) | c.3422A>G | Exon 28 | Missense | Transmembrane | TNDM | 0 | 0 | Paris unpublished |
| p.(Leu1148Arg) | c.3443T>G | Exon 28 | Missense | Transmembrane | HI | 2 | 0.000007995 | ([Fernandez-Marmiesse, et al., 2006](#_ENREF_106); [Gussinyer, et al., 2008](#_ENREF_141)) |
| p.(Cys1150Ter) | c.3450T>A | Exon 28 | Nonsense |  | HI | 1 | 0.00000399 | Exeter unpublished |
| p.(Ala1153Thr) | c.3457G>A | Exon 28 | Missense | Transmembrane | HI | 0 | 0 | ([Arya, et al., 2014b](#_ENREF_20)) |
| p.(Ala1153Val) | c.3458C>T | Exon 28 | Missense | Transmembrane | HI | 0 | 0 | Exeter unpublished |
| p.(Ala1153Gly) | c.3458C>G | Exon 28 | Missense | Transmembrane | NDM | 0 | 0 | Exeter unpublished |
| p.(Pro1162Arg) | c.3485C>G | Exon 28 | Missense | Transmembrane | HI | 0 | 0 | ([Xu, et al., 2018](#_ENREF_409)) |
| p.(Phe1164Leu) | c.3490T>C | Exon 28 | Missense | Transmembrane | DEND | 0 | 0 | ([Dalvi, et al., 2017](#_ENREF_74); [Thakkar, et al., 2014](#_ENREF_383)) |
| p.(Leu1171ArgfsTer38) | c.3512del | Exon 28 | Frameshift |  | HI | 0 | 0 | ([Arya, et al., 2014b](#_ENREF_20); [Calton, et al., 2013](#_ENREF_52); [Snider, et al., 2013](#_ENREF_356)) |
| p.(Cys1175Phe) | c.3524G>T | Exon 28 | Missense | Transmembrane | HI | 0 | 0 | ([Ortiz, et al., 2012](#_ENREF_282)) |
| p.(Tyr1176Cys) | c.3527A>G | Exon 28 | Missense | Transmembrane | TNDM | 0 | 0 | ([Poovazhagi, 2014](#_ENREF_301)) |
| p.(Phe1177Leu) | c.3529T>C | Exon 28 | Missense | Transmembrane | TNDM | 0 | 0 | ([Hashimoto, et al., 2017](#_ENREF_149)) |
| p.(Tyr1181Ter) | c.3543C>A | Exon 28 | Nonsense |  | HI | 0 | 0 | Paris unpublished |
| p.(Gln1179Arg) | c.3536A>G | Exon 28 | Missense | Transmembrane | PNDM | 0 | 0 | ([Babenko, 2008](#_ENREF_23); [Ortiz, et al., 2013](#_ENREF_281); [Ortiz, et al., 2012](#_ENREF_282)) |
| p.(Phe1182Ser) | c.3546C>G | Exon 28 | Missense | Cytoplasmic | TNDM | 0 | 0 | ([Vasanwala, et al., 2014](#_ENREF_395)) |
| p.(Phe1182Leu) | c.3546C>A | Exon 28 | Missense | Cytoplasmic | TNDM  PNDM | 0 | 0 | Exeter unpublished  Exeter unpublished |
| p.(Arg1183Trp) | c.3547C>T | Exon 28 | Missense | Cytoplasmic | TNDM  Later-onset diabetes | 0 | 0 | ([Batra, et al., 2009](#_ENREF_29); [Bonnefond, et al., 2014](#_ENREF_39); [Flanagan, et al., 2007](#_ENREF_113); [Hashimoto, et al., 2017](#_ENREF_149); [Huopio, et al., 2016](#_ENREF_158); [Kong and Kim, 2011](#_ENREF_208); [Patch, et al., 2007](#_ENREF_290); [Zhang, et al., 2015a](#_ENREF_419)) |
| p.(Arg1183Gln) | c.3548G>A | Exon 28 | Missense | Cytoplasmic | TNDM  Later-onset diabetes | 0 | 0 | ([Babenko, et al., 2006](#_ENREF_24); [Flanagan, et al., 2007](#_ENREF_113); [Ortiz and Bryan, 2015](#_ENREF_280); [Ortiz, et al., 2012](#_ENREF_282); [Vaxillaire, et al., 2007](#_ENREF_396)) |
| p.(Ala1185Glu) | c.3554C>A | Exon 28 | Missense | Cytoplasmic | HI | 1 | 0.000003979 | ([Arya, et al., 2014b](#_ENREF_20); [Ellard, et al., 2007](#_ENREF_98); [Haliloglu, et al., 2018](#_ENREF_143)) |
| p.(Ala1185Val) | c.3554C>T | Exon 28 | Missense | Cytoplasmic | HI | 1 | 0.00000406 | ([Apperley, et al., 2019](#_ENREF_16); [Arya, et al., 2014b](#_ENREF_20)) |
| p.(Ser1186Ala) | c.3556T>G | Exon 28 | Missense | Cytoplasmic | HI | 0 | 0 | ([Ortiz and Bryan, 2015](#_ENREF_280); [Ortiz, et al., 2012](#_ENREF_282)) |
| p.(Arg1187Trp) | c.3559A>T | Exon 28 | Missense | Cytoplasmic | HI | 0 | 0 | ([Snider, et al., 2013](#_ENREF_356)) |
| p.(Arg1187Gly) | c.3559A>G | Exon 28 | Missense | Cytoplasmic | HI | 0 | 0 | ([Henquin, et al., 2011](#_ENREF_151)) |
| p.? | c.3560+1G>A | Intron 28 | Aberrant splicing |  | HI | 0 | 0 | ([Bendix, et al., 2018](#_ENREF_32)) |
| p.? | c.3561-3C>G | Intron 28 | Aberrant splicing |  | HI | 0 | 0 | ([Henquin, et al., 2011](#_ENREF_151)) |
| p.(Asp1193MetfsTer16) | c.3577del | Exon 29 | Frameshift |  | HI | 0 | 0 | ([Bellanne-Chantelot, et al., 2010](#_ENREF_31); [Fernandez-Marmiesse, et al., 2006](#_ENREF_106); [Greer, et al., 2007](#_ENREF_138); [Gussinyer, et al., 2008](#_ENREF_141); [Martinez, et al., 2016](#_ENREF_243); [Stanley, et al., 2004](#_ENREF_361); [Suchi, et al., 2003](#_ENREF_364); [Suchi, et al., 2006](#_ENREF_365)) |
| p.(Asp1194Val) | c.3581A>T | Exon 29 | Missense | Cytoplasmic | HI | 15 | 0.00005303 | Odense unpublished |
| p.(Gln1197Ter) | c.3589C>T | Exon 29 | Nonsense |  | HI | 0 | 0 | ([Sang, et al., 2014a](#_ENREF_331); [Xu, et al., 2013](#_ENREF_408)) |
| p.(Pro1199Ser) | c.3595C>T | Exon 29 | Missense | Cytoplasmic | TNDM | 0 | 0 | Exeter unpublished |
| p.(Pro1199Gln) | c.3596C>A | Exon 29 | Missense | Cytoplasmic | TNDM | 0 | 0 | Exeter unpublished |
| p.(Pro1199Leu) | c.3596C>T | Exon 29 | Missense | Cytoplasmic | PNDM | 0 | 0 | ([Abraham, et al., 2014](#_ENREF_4); [Hashimoto, et al., 2017](#_ENREF_149); [Oztekin, et al., 2012](#_ENREF_286); [Takagi, et al., 2013](#_ENREF_371)) |
| p.(Leu1201_Ser1202del) | c.3600_3605del | Exon 29 | In frame deletion | Cytoplasmic | HI | 0 | 0 | ([Craigie, et al., 2018](#_ENREF_72)) |
| p.(Leu1201ThrfsTer18) | c.3600_3604del | Exon 29 | Frameshift |  | HI | 0 | 0 | Odense unpublished |
| p.(Glu1209del) | c.3626_3628del | Exon 29 | In frame deletion | Cytoplasmic | HI | 0 | 0 | ([Martinez, et al., 2016](#_ENREF_243)) |
| p.(Gly1210ArgfsTer12) | c.3627_3628insCGTA | Exon 29 | Frameshift |  | HI | 0 | 0 | ([Park, et al., 2011](#_ENREF_288)) |
| p.(Ile1214ProfsTer53) | c.3637_3638dup | Exon 29 | Frameshift |  | HI | 0 | 0 | ([Mohnike, et al., 2014](#_ENREF_256)) |
| p.(Arg1215Trp) | c.3643C>T | Exon 29 | Missense | Cytoplasmic | HI | 15 | 0.00005302 | ([Al-Agha and Ahmad, 2013](#_ENREF_9); [Brady, et al., 2015](#_ENREF_43); [Mohnike, et al., 2014](#_ENREF_256); [Peranteau, et al., 2006](#_ENREF_294); [Rozenkova, et al., 2015](#_ENREF_318); [Stanley, et al., 2004](#_ENREF_361); [Suchi, et al., 2006](#_ENREF_365); [Yan, et al., 2007](#_ENREF_413); [Zhang, et al., 2015a](#_ENREF_419)) |
| p.(Arg1215Gln) | c.3644G>A | Exon 29 | Missense | Cytoplasmic | HI | 6 | 0.00002386 | ([Bellanne-Chantelot, et al., 2010](#_ENREF_31); [Nestorowicz, et al., 1998](#_ENREF_265); [Shyng, et al., 1998](#_ENREF_350); [Stanley, et al., 2004](#_ENREF_361); [Suchi, et al., 2003](#_ENREF_364)) |
| p.(Arg1218Lys) | c.3653G>A | Exon 29 | Missense | Cytoplasmic | HI | 0 | 0 | ([Sang, et al., 2014a](#_ENREF_331)) |
| p.? | c.3653+2T>A | Intron 29 | Aberrant splicing |  | HI | 0 | 0 | Exeter unpublished |
| p.? | c.3653+2T>C | Intron 29 | Aberrant splicing |  | HI | 0 | 0 | ([Ohkubo, et al., 2005](#_ENREF_275)) |
| p.? | c.3654-1G>T | Intron 29 | Aberrant splicing |  | HI | 0 | 0 | ([Aguilar-Bryan and Bryan, 1999](#_ENREF_7)) |
| p.(Asn1245Asp) | c.3733A>G | Exon 30 | Missense | Cytoplasmic | Later-onset diabetes | 0 | 0 | ([Bowman, et al., 2012](#_ENREF_42)) |
| p.(Arg1246Gly) | c.3736A>G | Exon 30 | Missense | Cytoplasmic | HI | 0 | 0 | ([Xu, et al., 2018](#_ENREF_409)) |
| p.(Trp1247Arg) | c.3739T>A | Exon 30 | Missense | Cytoplasmic | HI | 0 | 0 | ([Mohnike, et al., 2014](#_ENREF_256)) |
| p.(Trp1247Arg) | c.3739T>C | Exon 30 | Missense | Cytoplasmic | HI | 0 | 0 | ([Xu, et al., 2018](#_ENREF_409)) |
| p.(Leu1248ArgfsTer18) | c.3743del | Exon 30 | Frameshift |  | HI | 0 | 0 | ([Mohnike, et al., 2014](#_ENREF_256)) |
| p.(Val1250Phe) | c.3748G>T | Exon 30 | Missense | Cytoplasmic | HI | 0 | 0 | ([Yorifuji, et al., 2011](#_ENREF_417)) |
| p.(Arg1251Ter) | c.3751C>T | Exon 30 | Nonsense |  | HI | 0 | 0 | ([Bellanne-Chantelot, et al., 2010](#_ENREF_31); [Fan, et al., 2015](#_ENREF_103); [Fernandez-Marmiesse, et al., 2006](#_ENREF_106); [Gussinyer, et al., 2008](#_ENREF_141); [Martinez, et al., 2016](#_ENREF_243); [Mohnike, et al., 2014](#_ENREF_256); [Yorifuji, et al., 2011](#_ENREF_417)) |
| p.(Met1252GlyfsTer154) | c.3754_3755del | Exon 30 | Frameshift |  | HI | 0 | 0 | ([Szymanowski, et al., 2016](#_ENREF_368)) |
| p.? | c.3756+1G>A | Intron 30 | Aberrant splicing |  | HI | 0 | 0 | ([Snider, et al., 2013](#_ENREF_356)) |
| p.? | c.3757-2A>C | Intron 30 | Aberrant splicing |  | HI | 0 | 0 | ([Jahnavi, et al., 2014](#_ENREF_175)) |
| p.? | c.3757-17_3823del | Intron 30 | Aberrant splicing |  | HI | 0 | 0 | Exeter unpublished |
| p.(Glu1253Ter) | c.3757G>T | Exon 31 | Nonsense |  | HI | 0 | 0 | Exeter unpublished |
| p.(Gly1256Ser) | c.3766G>A | Exon 31 | Missense | Transmembrane | PNDM | 0 | 0 | ([Jain, et al., 2012](#_ENREF_177)) |
| p.(Ala1263Thr) | c.3787G>A | Exon 31 | Missense | Transmembrane | HI | 3 | 0.00001065 | ([Arya, et al., 2014b](#_ENREF_20); [Christesen, et al., 2012](#_ENREF_64); [Salomon-Estebanez, et al., 2016](#_ENREF_328)) |
| p.(Ala1264Glu) | c.3791C>A | Exon 31 | Missense | Transmembrane | PNDM | 0 | 0 | ([Flanagan, et al., 2014](#_ENREF_108)) |
| p.(Ala1264Val) | c.3791C>T | Exon 31 | Missense | Transmembrane | TNDM | 0 | 0 | ([Hashimoto, et al., 2017](#_ENREF_149)) |
| p.(Ser1267Phe) | c.3800C>T | Exon 31 | Missense | Transmembrane | NDM | 0 | 0 | Chicago unpublished |
| p.(Asn1270_Ser1271del) | c.3808_3813del | Exon 31 | In frame deletion | Transmembrane | PNDM | 0 | 0 | ([Ganesh, et al., 2017](#_ENREF_122)) |
| p.(Leu1276Pro) | c.3827T>C | Exon 31 | Missense | Extracellular | Later-onset diabetes | 0 | 0 | Paris unpublished |
| p.(Ala1278LeufsTer12) | c.3831del | Exon 31 | Frameshift |  | HI | 1 | 0.000003995 | ([Snider, et al., 2013](#_ENREF_356)) |
| p.(Leu1283AlafsTer8) | c.3844_3845dup | Exon 31 | Frameshift |  | HI | 0 | 0 | Paris unpublished |
| p.(Val1281_Gly1282insAlaTyrProAlaGlnArgAlaLeuHisGln) | c.3844_3845insCTTACCCTGCACAGAGAGCTCTCCACCAGG | Exon 31 | In frame insertion | Transmembrane | HI | 0 | 0 | ([Snider, et al., 2013](#_ENREF_356)) |
| p.(Tyr1287Ter) | c.3861C>A | Exon 31 | Nonsense |  | HI | 0 | 0 | Exeter unpublished  Odense unpublished |
| p.(Met1290Val) | c.3868A>G | Exon 31 | Missense | Transmembrane | PNDM | 0 | 0 | ([Patch, et al., 2007](#_ENREF_290)) |
| p.(Met1290Ile) | c.3870G>T | Exon 31 | Missense | Transmembrane | HI | 0 | 0 | Paris unpublished |
| p.? | c.3871-1G>A | Intron 31 | Aberrant splicing |  | HI | 4 | 0.00001614 | ([Yadav, et al., 2012](#_ENREF_410)) |
| p.? | c.3871-2A>G | Intron 31 | Aberrant splicing |  | HI | 0 | 0 | Exeter unpublished |
| p.? | c.3871-31_3994del | Intron 31 - Exon 32 | Deletion | Transmembrane | HI | 0 | 0 | ([Mohnike, et al., 2014](#_ENREF_256)) |
| p.? | c.(3870+1_3871-1)_(3991+1_3992-1)del | Exon 32 | Deletion | Transmembrane | HI | 0 | 0 | ([Bennett, et al., 2015](#_ENREF_33)) |
| p.(Leu1295Phe) | c.3883C>T | Exon 32 | Missense | Transmembrane | PNDM | 0 | 0 | Exeter unpublished |
| p.(Asn1296Lys) | c.3888C>G | Exon 32 | Missense | Transmembrane | HI | 0 | 0 | ([Fernandez-Marmiesse, et al., 2006](#_ENREF_106)) |
| p.(Trp1297Ter) | c.3891G>A | Exon 32 | Nonsense |  | HI | 0 | 0 | ([Gong, et al., 2016](#_ENREF_136)) |
| p.(Ala1311GlyfsTer96) | c.3931dup | Exon 32 | Frameshift |  | HI | 0 | 0 | ([Yorifuji, et al., 2011](#_ENREF_417)) |
| p.(Arg1314His) | c.3941G>A | Exon 32 | Missense | Cytoplasmic | TNDM | 7 | 0.00002479 | ([Patch, et al., 2007](#_ENREF_290)) |
| p.(Glu1324Lys) | c.3970G>A | Exon 32 | Missense | Cytoplasmic | HI | 0 | 0 | ([Faletra, et al., 2013b](#_ENREF_102)) |
| p.(Glu1324Ter) | c.3970G>T | Exon 32 | Nonsense |  | HI | 0 | 0 | Exeter unpublished |
| p.(Tyr1326Ter) | c.3978del | Exon 32 | Nonsense |  | HI | 0 | 0 | Exeter unpublished |
| p.(Glu1327Ter) | c.3979G>T | Exon 32 | Nonsense |  | HI | 0 | 0 | Exeter unpublished |
| p.? | c.3991+1G>A | Intron 32 | Aberrant splicing |  | HI | 0 | 0 | Exeter unpublished |
| p.? | c.3991+2T>C | Intron 32 | Aberrant splicing |  | HI | 2 | 0.000007968 | ([Martinez, et al., 2016](#_ENREF_243)) |
| p.? | c.3991+2_3991+15del | Intron 32 | Aberrant splicing |  | HI | 0 | 0 | ([Bellanne-Chantelot, et al., 2010](#_ENREF_31); [Giurgea, et al., 2006](#_ENREF_126); [Henquin, et al., 2011](#_ENREF_151)) |
| p.? | c.3992-9G>A | Intron 32 | Aberrant splicing |  | HI | 83 | 0.0002935 | ([Arya, et al., 2014b](#_ENREF_20); [Arya, et al., 2014c](#_ENREF_21); [Bellanne-Chantelot, et al., 2010](#_ENREF_31); [Calabria, et al., 2012](#_ENREF_51); [Christesen, et al., 2001](#_ENREF_63); [Damaj, et al., 2008](#_ENREF_75); [Del Roio Liberatore, et al., 2015](#_ENREF_86); [Fourtner, et al., 2006](#_ENREF_118); [Glaser, et al., 1999](#_ENREF_127); [Grimberg, et al., 2001](#_ENREF_139); [Gussinyer, et al., 2008](#_ENREF_141); [Guven, et al., 2016](#_ENREF_142); [Hardy, et al., 2007](#_ENREF_146); [Ismail, et al., 2012](#_ENREF_172); [Kapoor, et al., 2012](#_ENREF_189); [Mohnike, et al., 2014](#_ENREF_256); [Nestorowicz, et al., 1998](#_ENREF_265); [Nestorowicz, et al., 1996](#_ENREF_267); [Saint-Martin, et al., 2011](#_ENREF_324); [Shyng, et al., 1998](#_ENREF_350); [Stanley, et al., 2004](#_ENREF_361); [Suchi, et al., 2003](#_ENREF_364); [Suchi, et al., 2006](#_ENREF_365); [Thomas, et al., 1995](#_ENREF_387); [Tornovsky, et al., 2004](#_ENREF_392)) |
| p.? | c.3992-3C>G | Intron 32 | Aberrant splicing |  | HI | 0 | 0 | ([Bellanne-Chantelot, et al., 2010](#_ENREF_31); [Nestorowicz, et al., 1998](#_ENREF_265); [Shyng, et al., 1998](#_ENREF_350)) |
| p.? | c.3992-2A>G | Intron 32 | Aberrant splicing |  | HI | 1 | 0.000003536 | ([Proverbio, et al., 2013](#_ENREF_312)) |
| p.(Ser1333Ter) | c.3998C>A | Exon 33 | Nonsense |  | HI | 1 | 0.000003977 | Paris unpublished |
| p.(Lys1337Asn) | c.4011G>C | Exon33 | Missense | Cytoplasmic | HI | 8 | 0.00003181 | ([Henwood, et al., 2005](#_ENREF_152); [Yan, et al., 2007](#_ENREF_413)) |
| p.(Trp1339Arg) | c.4015T>C | Exon 33 | Missense | Cytoplasmic | HI | 0 | 0 | ([Snider, et al., 2013](#_ENREF_356)) |
| p.(Trp1339Ter) | c.4017G>A | Exon 33 | Nonsense |  | HI | 0 | 0 | ([Aguilar-Bryan and Bryan, 1999](#_ENREF_7); [Celik, et al., 2013](#_ENREF_56); [Durmaz, et al., 2014](#_ENREF_95)) |
| p.(Gln1342Ter) | c.4024C>T | Exon 33 | Nonsense |  | HI | 0 | 0 | ([Mohnike, et al., 2014](#_ENREF_256)) |
| p.(Gly1343Glu) | c.4028G>A | Exon 33 | Missense | Cytoplasmic | HI | 0 | 0 | ([Tornovsky, et al., 2004](#_ENREF_392)) |
| p.(Ile1347SerfsTer4) | c.4038del | Exon 33 | Frameshift |  | HI | 0 | 0 | ([Mohnike, et al., 2014](#_ENREF_256)) |
| p.(Ile1347Phe) | c.4039A>T | Exon 33 | Missense | Cytoplasmic | HI | 0 | 0 | Paris unpublished |
| p.(Gln1348Ter) | c.4042C>T | Exon 33 | Nonsense |  | HI | 0 | 0 | ([Ni, et al., 2019](#_ENREF_268)) |
| p.(Asn1349SerfsTer5) | c.4045_4061delinsT | Exon 33 | Frameshift |  | HI | 0 | 0 | Exeter unpublished |
| p.(Leu1350Gln) | c.4049T>A | Exon 33 | Missense | Cytoplasmic | HI | 0 | 0 | ([Stanley, et al., 2004](#_ENREF_361); [Suchi, et al., 2006](#_ENREF_365); [Yan, et al., 2007](#_ENREF_413)) |
| p.(Arg1353His) | c.4058G>A | Exon 33 | Missense | Cytoplasmic | HI  Later-onset diabetes | 5 | 0.00001988 | ([Calabria, et al., 2012](#_ENREF_51); [Khoriati, et al., 2013](#_ENREF_196); [Koufakis, et al., 2019](#_ENREF_212); [Magge, et al., 2004](#_ENREF_233)) |
| p.(Arg1353Pro) | c.4058G>C | Exon 33 | Missense | Cytoplasmic | HI | 2 | 0.000007954 | ([Giurgea, et al., 2004](#_ENREF_125); [Saint-Martin, et al., 2015](#_ENREF_325); [Verkarre, et al., 1998](#_ENREF_400)) |
| p.(Tyr1354Asn) | c.4060T>A | Exon 33 | Missense | Cytoplasmic | HI | 0 | 0 | ([Martinez, et al., 2016](#_ENREF_243)) |
| p.(Tyr1354Ter) | c.4062C>A | Exon 33 | Nonsense |  | HI | 0 | 0 | ([Bendix, et al., 2018](#_ENREF_32); [Ohkubo, et al., 2005](#_ENREF_275)) |
| p.(Pro1360Leu) | c.4079C>T | Exon 33 | Missense | Cytoplasmic | HI | 3 | 0.00001061 | ([Banerjee, et al., 2011](#_ENREF_27)) |
| p.(Val1361Met) | c.4081G>A | Exon 33 | Missense | Cytoplasmic | HI | 0 | 0 | ([Aguilar-Bryan and Bryan, 1999](#_ENREF_7)) |
| p.(His1364Gln) | c.4092C>G | Exon 33 | Missense | Cytoplasmic | HI | 0 | 0 | ([Bellanne-Chantelot, et al., 2010](#_ENREF_31)) |
| p.(Ala1367Thr) | c.4099G>A | Exon 33 | Missense | Cytoplasmic | Later-onset diabetes | 0 | 0 | ([Johansson, et al., 2012](#_ENREF_181)) |
| p.(Ala1367Asp) | c.4100C>A | Exon 33 | Missense | Cytoplasmic | HI | 0 | 0 | ([Ocal, et al., 2011](#_ENREF_274)) |
| p.(Gln1373Ter) | c.4117C>T | Exon 33 | Nonsense |  | HI | 1 | 0.00000398 | ([Kapoor, et al., 2013](#_ENREF_188)) |
| p.(Lys1374del) | c.4120_4122del | Exon 33 | In frame deletion | Cytoplasmic | HI | 0 | 0 | ([Rozenkova, et al., 2015](#_ENREF_318)) |
| p.(Lys1374Arg) | c.4121A>G | Exon 33 | Missense | Cytoplasmic | HI | 0 | 0 | ([Pinney, et al., 2008](#_ENREF_299); [Snider, et al., 2013](#_ENREF_356)) |
| p.? | c.4122+1G>A | Intron 33 | Aberrant splicing |  | HI | 0 | 0 | ([Snider, et al., 2013](#_ENREF_356))) |
| p.? | c.(4122+1_4123-1)_(4548+1_4549-1)del | Exons 34 -37 | Deletion |  | HI | 0 | 0 | ([Mohnike, et al., 2014](#_ENREF_256)) |
| p.(Gly1376Arg) | c.4126G>A | Exon 34 | Missense | Cytoplasmic | HI | 1 | 0.000005248 | ([Bellanne-Chantelot, et al., 2010](#_ENREF_31)) |
| p.(Gly1376_Ile1377del) | c.4126_4131del | Exon 34 | In frame deletion | Cytoplasmic | HI | 0 | 0 | ([Mohnike, et al., 2014](#_ENREF_256)) |
| p.(Gly1379Arg) | c.4135G>C | Exon 34 | Missense | NBD2 | HI | 0 | 0 | ([Mohnike, et al., 2014](#_ENREF_256); [Nestorowicz, et al., 1998](#_ENREF_265); [Suchi, et al., 2006](#_ENREF_365)) |
| p.(Gly1379Ser) | c.4135G>A | Exon 34 | Missense | NBD2 | HI | 0 | 0 | ([Mohnike, et al., 2014](#_ENREF_256); [Saint-Martin, et al., 2015](#_ENREF_325)) |
| p.(Arg1380Cys) | c.4138C>T | Exon 34 | Missense | NBD2 | TNDM | 1 | 0.000004973 | ([Alkorta-Aranburu, et al., 2014](#_ENREF_13); [Babenko, et al., 2006](#_ENREF_24); [de Wet, et al., 2007](#_ENREF_84); [Flanagan, et al., 2007](#_ENREF_113); [Hashimoto, et al., 2017](#_ENREF_149); [Karges, et al., 2012](#_ENREF_191)) |
| p.(Arg1380ValfsTer81) | c.4138_4141delinsGTG | Exon 34 | Frameshift |  | HI | 0 | 0 | ([de Lonlay-Debeney, et al., 1999](#_ENREF_79)) |
| p.(Arg1380Ser) | c.4138C>A | Exon 34 | Missense | NBD2 | NDM | 4 | 0.00001989 | ([Bennett, et al., 2015](#_ENREF_33)) |
| p.(Arg1380His) | c.4139G>A | Exon 34 | Missense | NBD2 | TNDM  Later-onset diabetes | 0 | 0 | ([Bourron, et al., 2012](#_ENREF_41); [Esmatjes, et al., 2008](#_ENREF_100); [Flanagan, et al., 2007](#_ENREF_113); [Hartemann-Heurtier, et al., 2009](#_ENREF_148); [Riveline, et al., 2012](#_ENREF_317); [Vaxillaire, et al., 2007](#_ENREF_396)) |
| p.(Arg1380Leu) | c.4139G>T | Exon 34 | Missense | NBD2 | NDM | 0 | 0 | ([de Wet, et al., 2007](#_ENREF_84); [Iafusco, et al., 2012](#_ENREF_162); [Patch, et al., 2007](#_ENREF_290); [Tammaro and Ashcroft, 2009](#_ENREF_374)) |
| p.(Arg1380Pro) | c.4139G>C | Exon 34 | Missense | NBD2 | NDM | 0 | 0 | Exeter unpublished |
| p.(Thr1381GlnfsTer80) | c.4141_4143delinsCA | Exon 34 | Frameshift |  | HI | 0 | 0 | ([Bennett, et al., 2015](#_ENREF_33)) |
| p.(Thr1381Asn) | c.4142C>A | Exon 34 | Missense | NBD2 | TNDM | 0 | 0 | Exeter unpublished |
| p.(Gly1382Ser) | c.4144G>A | Exon 34 | Missense | NBD2 | HI | 2 | 0.000009785 | ([Christesen, et al., 2001](#_ENREF_63); [Nestorowicz, et al., 1998](#_ENREF_265); [Salomon-Estebanez, et al., 2016](#_ENREF_328); [Shyng, et al., 1998](#_ENREF_350)) |
| p.(Gly1384Arg) | c.4150G>A | Exon 34 | Missense | NBD2 | HI | 0 | 0.000004773 | ([Bellanne-Chantelot, et al., 2010](#_ENREF_31); [Snider, et al., 2013](#_ENREF_356)) |
| p.(Gly1384Glu) | c.4151G>A | Exon 34 | Missense | NBD2 | HI | 0 | 0 | ([Macmullen, et al., 2011](#_ENREF_230)) |
| p.(Lys1385Gln) | c.4153A>C | Exon 34 | Missense | NBD2 | HI | 0 | 0 | ([Ohkubo, et al., 2005](#_ENREF_275)) |
| p.(Lys1385ValfsTer21) | c.4153_4154del | Exon 34 | Frameshift |  | HI | 0 | 0 | ([Fournet, et al., 2001](#_ENREF_117); [Giurgea, et al., 2004](#_ENREF_125)) |
| p.(Lys1385IlefsTer21) | c.4154_4155del | Exon 34 | Frameshift |  | HI | 0 | 0 | ([Bellanne-Chantelot, et al., 2010](#_ENREF_31)) |
| p.(Ser1386Pro) | c.4156T>C | Exon 34 | Missense | Cytoplasmic | HI | 0 | 0 | ([Natarajan, et al., 2007](#_ENREF_263); [Pinney, et al., 2008](#_ENREF_299)) |
| p.(Ser1387del) | c.4160_4162del | Exon 34 | In frame deletion | Cytoplasmic | HI | 0 | 0 | ([Calabria, et al., 2012](#_ENREF_51); [Pinney, et al., 2008](#_ENREF_299); [Thornton, et al., 2003](#_ENREF_389)) |
| p.(Ser1387Phe) | c.4160C>T | Exon 34 | Missense | Cytoplasmic | HI | 0 | 0 | ([Aguilar-Bryan and Bryan, 1999](#_ENREF_7); [Bellanne-Chantelot, et al., 2010](#_ENREF_31); [Henquin, et al., 2011](#_ENREF_151); [Macmullen, et al., 2011](#_ENREF_230); [Park, et al., 2011](#_ENREF_288); [Saint-Martin, et al., 2015](#_ENREF_325); [Stanley, et al., 2004](#_ENREF_361); [Yan, et al., 2007](#_ENREF_413)) |
| p.(Ser1387Tyr) | c.4160C>A | Exon 34 | Missense | Cytoplasmic | HI | 0 | 0 | ([Macmullen, et al., 2011](#_ENREF_230)) |
| p.(Phe1388del) | c.4163_4165del | Exon 34 | In frame deletion | Cytoplasmic | HI | 6 | 0.00002439 | ([Arya, et al., 2014c](#_ENREF_21); [Cartier, et al., 2001](#_ENREF_55); [Fourtner, et al., 2006](#_ENREF_118); [Grimberg, et al., 2001](#_ENREF_139); [Hardy, et al., 2007](#_ENREF_146); [Nestorowicz, et al., 1998](#_ENREF_265); [Nestorowicz, et al., 1996](#_ENREF_267); [Shyng, et al., 1998](#_ENREF_350); [Stanley, et al., 2004](#_ENREF_361); [Suchi, et al., 2003](#_ENREF_364); [Suchi, et al., 2006](#_ENREF_365)) |
| p.(Ser1389Tyr) | c.4166C>A | Exon 34 | Missense | Cytoplasmic | HI | 0 | 0 | ([Bellanne-Chantelot, et al., 2010](#_ENREF_31); [Macmullen, et al., 2011](#_ENREF_230)) |
| p.(Leu1390Pro) | c.4169T>C | Exon 34 | Missense | Cytoplasmic | HI | 0 | 0 | ([Bellanne-Chantelot, et al., 2010](#_ENREF_31); [Yan, et al., 2007](#_ENREF_413)) |
| p.(Leu1390Arg) | c.4169T>G | Exon 34 | Missense | Cytoplasmic | HI | 0 | 0 | ([Banerjee, et al., 2011](#_ENREF_27); [Kapoor, et al., 2011](#_ENREF_190)) |
| p.(Phe1393Ile) | c.4177T>A | Exon 34 | Missense | Cytoplasmic | HI | 0 | 0 | ([Bellanne-Chantelot, et al., 2010](#_ENREF_31)) |
| p.(Phe1393Val) | c.4177T>G | Exon 34 | Missense | Cytoplasmic | TNDM | 0 | 0 | ([Alkorta-Aranburu, et al., 2014](#_ENREF_13)) |
| p.(Phe1393Leu) | c.4179C>G | Exon 34 | Missense | Cytoplasmic | HI | 0 | 0 | ([Gong, et al., 2016](#_ENREF_136)) |
| p.(Arg1394His) | c.4181G>A | Exon 34 | Missense | Cytoplasmic | HI | 9 | 0.00003764 | ([Nestorowicz, et al., 1998](#_ENREF_265); [Partridge, et al., 2001](#_ENREF_289); [Shyng, et al., 1998](#_ENREF_350)) |
| p.(Arg1394Leu) | c.4181G>T | Exon 34 | Missense | Cytoplasmic | HI | 0 | 0 | ([Brady, et al., 2015](#_ENREF_43)) |
| p.(Met1395Arg) | c.4184T>G | Exon 34 | Missense | Cytoplasmic | HI | 0 | 0 | ([Faletra, et al., 2013b](#_ENREF_102)) |
| p.(Asp1397Tyr) | c.4189G>T | Exon 34 | Missense | Cytoplasmic | HI | 0 | 0 | ([Yorifuji, et al., 2011](#_ENREF_417)) |
| p.(Gly1401Arg) | c.4201G>A | Exon 34 | Missense | Cytoplasmic | HI | 1 | 0.0000323 | ([Ellard, et al., 2007](#_ENREF_98); [Greer, et al., 2007](#_ENREF_138); [Sandal, et al., 2009](#_ENREF_329); [Stanley, et al., 2004](#_ENREF_361); [Suchi, et al., 2006](#_ENREF_365)) |
| p.(Gly1401Trp) | c.4201G>T | Exon 34 | Missense | Cytoplasmic | HI | 0 | 0 | Odense unpublished |
| p.? | c.4201+1G>C | Intron 34 | Aberrant splicing |  | HI | 0 | 0 | ([Bendix, et al., 2018](#_ENREF_32)) |
| p.? | c.4201+1G>T | Intron 34 | Aberrant splicing |  | HI | 0 | 0 | ([Xu, et al., 2018](#_ENREF_409)) |
| p.? | c.4202-7_4206del | Intron 34 | Aberrant splicing |  | HI | 0 | 0 | ([Fournet, et al., 2001](#_ENREF_117)) |
| p.? | c.4202-2A>T | Intron 34 | Aberrant splicing |  | HI | 0 | 0 | ([Mohnike, et al., 2014](#_ENREF_256)) |
| p.(His1402ThrfsTer59) | c.4203del | Exon 35 | Frameshift |  | HI | 0 | 0 | Exeter unpublished |
| p.(Ile1404Met) | c.4212C>G | Exon 35 | Missense | Cytoplasmic | HI | 1 | 0.000006232 | ([Mohnike, et al., 2014](#_ENREF_256)) |
| p.(Ile1405del) | c.4212_4214del | Exon 35 | In frame deletion | Cytoplasmic | HI | 0 | 0 | Exeter unpublished |
| p.(Ile1405del) | c.4213_4215del | Exon 35 | In frame deletion | Cytoplasmic | HI | 0 | 0 | ([Bellanne-Chantelot, et al., 2010](#_ENREF_31); [Giurgea, et al., 2004](#_ENREF_125)) |
| p.(Ile1405Thr) | c.4214T>C | Exon 35 | Missense | Cytoplasmic | HI | 0 | 0 | ([Mohnike, et al., 2014](#_ENREF_256)) |
| p.(Asp1406His) | c.4216G>C | Exon 35 | Missense | Cytoplasmic | HI | 0 | 0 | ([Greer, et al., 2007](#_ENREF_138)) |
| p.(Ile1410Phe) | c.4228A>T | Exon 35 | Missense | Cytoplasmic | HI | 0 | 0 | ([Bellanne-Chantelot, et al., 2010](#_ENREF_31)) |
| p.(Pro1414Leu) | c.4241C>T | Exon 35 | Missense | Cytoplasmic | HI | 0 | 0 | ([Bellanne-Chantelot, et al., 2010](#_ENREF_31); [Bendix, et al., 2018](#_ENREF_32); [Giurgea, et al., 2006](#_ENREF_126); [Mohnike, et al., 2014](#_ENREF_256); [Ocal, et al., 2011](#_ENREF_274); [Sandal, et al., 2009](#_ENREF_329)) |
| p.(Arg1419His) | c.4256G>A | Exon 35 | Missense | Cytoplasmic | HI | 0 | 0 | ([Harel, et al., 2015](#_ENREF_147); [Tornovsky, et al., 2004](#_ENREF_392)) |
| p.(Arg1419Leu) | c.4256G>T | Exon 35 | Missense | Cytoplasmic | HI | 0 | 0 | ([Mohnike, et al., 2014](#_ENREF_256)) |
| p.(Arg1421Cys) | c.4261C>T | Exon 35 | Missense | Cytoplasmic | HI | 0 | 0 | ([Bellanne-Chantelot, et al., 2010](#_ENREF_31); [de Lonlay-Debeney, et al., 1999](#_ENREF_79); [Greer, et al., 2007](#_ENREF_138); [Matsuo, et al., 2000](#_ENREF_248); [Tanizawa, et al., 2000](#_ENREF_378); [Verkarre, et al., 1998](#_ENREF_400)) |
| p.(Arg1421His) | c.4262G>A | Exon 35 | Missense | Cytoplasmic | HI  Later-onset diabetes | 4 | 0.00002357 | ([Baier, et al., 2015](#_ENREF_26); [Saito-Hakoda, et al., 2012](#_ENREF_326)) |
| p.(Ser1423Pro) | c.4267T>C | Exon 35 | Missense | Cytoplasmic | HI | 0 | 0 | Exeter unpublished |
| p.(Ser1423Cys) | c.4268C>G | Exon 35 | Missense | Cytoplasmic | NDM | 0 | 0 | Chicago unpublished |
| p.(Ile1425Val) | c.4273A>G | Exon 35 | Missense | Cytoplasmic | PNDM | 0 | 0 | ([Babenko, et al., 2006](#_ENREF_24); [Ortiz and Bryan, 2015](#_ENREF_280)) |
| p.(Ile1425Leu) | c.4273A>C | Exon 35 | Missense | Cytoplasmic | HI | 0 | 0 | ([Nessa, et al., 2015](#_ENREF_264)) |
| p.(Asp1428ArgfsTer6) | c.4282_4298del | Exon 35 | Frameshift |  | HI | 0 | 0 | Chicago unpublished |
| p.(Pro1429LeufsTer8) | c.4286_4293del | Exon 35 | Frameshift |  | HI | 0 | 0 | Exeter unpublished |
| p.(Val1430SerfsTer31) | c.4287del | Exon 35 | Frameshift |  | HI | 0 | 0 | ([Banerjee, et al., 2011](#_ENREF_27); [Fan, et al., 2015](#_ENREF_103)) |
| p.(Val1430Ala) | c.4289T>C | Exon 35 | Missense | Cytoplasmic | HI | 0 | 0 | ([Mohnike, et al., 2014](#_ENREF_256)) |
| p.(Leu1431Phe) | c.4291C>T | Exon 35 | Missense | Cytoplasmic | HI | 0 | 0 | ([Arya, et al., 2014b](#_ENREF_20); [Kapoor, et al., 2011](#_ENREF_190)) |
| p.(Gly1434Ser) | c.4300G>A | Exon 35 | Missense | Cytoplasmic | HI | 1 | 0.00000523 | ([Bellanne-Chantelot, et al., 2010](#_ENREF_31)) |
| p.(Ile1436Ser) | c.4307T>G | Exon 35 | Missense | Cytoplasmic | HI | 0 | 0 | ([Shah, et al., 2015](#_ENREF_337)) |
| p.(Arg1437Gly) | c.4309C>G | Exon 35 | Missense | Cytoplasmic | HI | 0 | 0 | ([Bellanne-Chantelot, et al., 2010](#_ENREF_31)) |
| p.(Arg1437Ter) | c.4309C>T | Exon 35 | Nonsense |  | HI | 2 | 0.0000127 | ([Bendix, et al., 2018](#_ENREF_32); [Powell, et al., 2011](#_ENREF_302); [Snider, et al., 2013](#_ENREF_356)) |
| p.(Arg1437Gln) | c.4310G>A | Exon 35 | Missense | Cytoplasmic | HI | 1 | 0.00003186 | ([Arya, et al., 2014b](#_ENREF_20); [Arya, et al., 2014c](#_ENREF_21); [Bennett, et al., 2015](#_ENREF_33); [Darendeliler, et al., 2002](#_ENREF_76); [Dunne, et al., 1997](#_ENREF_93); [Fernandez-Marmiesse, et al., 2006](#_ENREF_106); [Greer, et al., 2007](#_ENREF_138); [Meissner, et al., 1997](#_ENREF_251); [Muzyamba, et al., 2007](#_ENREF_260); [Nestorowicz, et al., 1998](#_ENREF_265); [Rozenkova, et al., 2015](#_ENREF_318); [Tanizawa, et al., 2000](#_ENREF_378); [Thomas, et al., 1995](#_ENREF_387)) |
| p.? | c.4310+1G>A | Intron 35 | Aberrant splicing |  | HI | 0 | 0 | ([Craigie, et al., 2018](#_ENREF_72)) |
| p.? | c.4311-2A>G | Intron 35 | Aberrant splicing |  | HI | 3 | 0.00001529 | ([Kapoor, et al., 2013](#_ENREF_188)) |
| p.? | c.4311-1G>T | Intron 35 | Aberrant splicing |  | HI | 0 | 0 | Paris unpublished |
| p.(Pro1442LeufsTer19) | c.4325del | Exon 36 | Frameshift |  | HI | 1 | 0.000004624 | ([Bellanne-Chantelot, et al., 2010](#_ENREF_31); [Henquin, et al., 2011](#_ENREF_151); [Snider, et al., 2013](#_ENREF_356)) |
| p.(Ser1449dup) | c.4346_4348dup | Exon 36 | Duplication |  | HI | 0 | 0 | ([Bellanne-Chantelot, et al., 2010](#_ENREF_31)) |
| p.(Leu1451Arg) | c.4352T>G | Exon 36 | Missense | Cytoplasmic | HI | 0 | 0 | ([Giurgea, et al., 2004](#_ENREF_125)) |
| p.(Leu1451Pro) | c.4352T>C | Exon 36 | Missense | Cytoplasmic | HI | 0 | 0 | ([Bellanne-Chantelot, et al., 2010](#_ENREF_31); [Fernandez-Marmiesse, et al., 2006](#_ENREF_106); [Martinez, et al., 2016](#_ENREF_243)) |
| p.(Trp1452Cys) | c.4356G>C | Exon 36 | Missense | Cytoplasmic | HI | 0 | 0 | Paris unpublished |
| p.(Glu1453Asp) | c.4359G>T | Exon 36 | Missense | Cytoplasmic | HI | 0 | 0 | ([Henquin, et al., 2011](#_ENREF_151)) |
| p.(Leu1455Arg) | c.4364T>G | Exon 36 | Missense | Cytoplasmic | HI | 0 | 0 | ([Jahnavi, et al., 2014](#_ENREF_175)) |
| p.(Ala1458Thr) | c.4372G>A | Exon 36 | Missense | Cytoplasmic | HI | 0 | 0 | ([Bellanne-Chantelot, et al., 2010](#_ENREF_31); [Boodhansingh, et al., 2019](#_ENREF_40); [Giurgea, et al., 2004](#_ENREF_125); [Huopio, et al., 2002](#_ENREF_157); [Macmullen, et al., 2011](#_ENREF_230); [Mohnike, et al., 2014](#_ENREF_256); [Reimann, et al., 2003](#_ENREF_315)) |
| p.(Ala1458Val) | c.4373C>T | Exon 36 | Missense | Cytoplasmic | HI | 0 | 0 | ([Bellanne-Chantelot, et al., 2010](#_ENREF_31)) |
| p.(Gln1459Glu) | c.4375C>G | Exon 36 | Missense | Cytoplasmic | HI | 0 | 0 | ([Kapoor, et al., 2011](#_ENREF_190)) |
| p.(Gln1459His) | c.4377G>C | Exon 36 | Missense | Cytoplasmic | HI | 0 | 0 | ([Gong, et al., 2016](#_ENREF_136); [Macmullen, et al., 2011](#_ENREF_230)) |
| p.(Leu1460Arg) | c.4379T>G | Exon 36 | Missense | Cytoplasmic | HI | 0 | 0 | ([Snider, et al., 2013](#_ENREF_356)) |
| p.(Val1464Ter) | c.4390del | Exon 36 | Nonsense |  | HI | 0 | 0 | ([Bellanne-Chantelot, et al., 2010](#_ENREF_31)) |
| p.(Gly1469Ter) | c.4405G>T | Exon 36 | Nonsense |  | HI | 0 | 0 | ([Ohkubo, et al., 2005](#_ENREF_275)) |
| p.(Gly1470Val) | c.4409G>T | Exon 36 | Missense | Cytoplasmic | HI | 0 | 0 | ([Henquin, et al., 2011](#_ENREF_151); [Otonkoski, et al., 2006](#_ENREF_284)) |
| p.(Leu1471ProfsTer27) | c.4412del | Exon 36 | Frameshift |  | HI | 0 | 0 | ([Du, et al., 2019](#_ENREF_92); [Kapoor, et al., 2013](#_ENREF_188)) |
| p.(Asp1472Asn) | c.4414G>A | Exon 36 | Missense | Cytoplasmic | HI | 4 | 0.00002179 | ([Arya, et al., 2014b](#_ENREF_20); [Bellanne-Chantelot, et al., 2010](#_ENREF_31); [Bendix, et al., 2018](#_ENREF_32); [Brunetti-Pierri, et al., 2008](#_ENREF_47); [Greer, et al., 2007](#_ENREF_138); [Hardy, et al., 2007](#_ENREF_146); [Muzyamba, et al., 2007](#_ENREF_260); [Stanley, et al., 2004](#_ENREF_361)) |
| p.(Asp1472His) | c.4414G>C | Exon 36 | Missense | Cytoplasmic | HI | 0 | 0 | ([Bellanne-Chantelot, et al., 2010](#_ENREF_31); [Giurgea, et al., 2004](#_ENREF_125); [Stanley, et al., 2004](#_ENREF_361); [Yan, et al., 2007](#_ENREF_413)) |
| p.? | c.(4414+1_4415-1)_(*4749+34)del | Exons 37-39 | Deletion |  | HI | 0 | 0 | Exeter unpublished |
| p.? | c.4415-13G>A | Intron 36 | Aberrant splicing |  | HI | 1 | 0.000003978 | ([Aguilar-Bryan and Bryan, 1999](#_ENREF_7); [Al-Agha and Ahmad, 2013](#_ENREF_9); [Bellanne-Chantelot, et al., 2010](#_ENREF_31); [Bendix, et al., 2018](#_ENREF_32); [Del Roio Liberatore, et al., 2015](#_ENREF_86); [Henquin, et al., 2011](#_ENREF_151); [Meder, et al., 2015](#_ENREF_250); [Ohkubo, et al., 2005](#_ENREF_275); [Yorifuji, et al., 2011](#_ENREF_417)) |
| p.? | c.4415-2A>G | Intron 36 | Aberrant splicing |  | HI | 0 | 0 | ([Lee, et al., 2015](#_ENREF_219)) |
| p.(Gly1478Val) | c.4433G>T | Exon 37 | Missense | Cytoplasmic | HI | 0 | 0 | ([Pinney, et al., 2008](#_ENREF_299)) |
| p.(Gly1479Arg) | c.4435G>A | Exon 37 | Missense | Cytoplasmic | HI  Later-onset diabetes* | 0 | 0 | ([Arya, et al., 2014c](#_ENREF_21); [Kapoor, et al., 2011](#_ENREF_190); [Nichols, et al., 1996](#_ENREF_269); [Ortiz and Bryan, 2015](#_ENREF_280); [Pinney, et al., 2008](#_ENREF_299); [Sandal, et al., 2009](#_ENREF_329))  Paris unpublished* |
| p.(Gly1479Glu) | c.4436G>A | Exon 37 | Missense | Cytoplasmic | HI | 0 | 0 | ([Snider, et al., 2013](#_ENREF_356)) |
| p.(Gly1479Ala) | c.4436G>C | Exon 37 | Missense | Cytoplasmic | HI | 0 | 0 | ([Kapoor, et al., 2013](#_ENREF_188)) |
| p.(Asn1481Ile) | c.4442A>T | Exon 37 | Missense | Cytoplasmic | HI | 0 | 0 | ([Bellanne-Chantelot, et al., 2010](#_ENREF_31)) |
| p.(Gly1485Arg) | c.4453G>A | Exon 37 | Missense | Cytoplasmic | HI | 0 | 0 | ([Mohnike, et al., 2014](#_ENREF_256)) |
| p.(Gly1485Val) | c.4454G>T | Exon 37 | Missense | Cytoplasmic | HI | 0 | 0 | Chicago unpublished |
| p.(Gly1485Glu) | c.4454G>A | Exon 37 | Missense | Cytoplasmic | HI | 0 | 0 | ([Flanagan, et al., 2011](#_ENREF_111)) |
| p.(Gln1486Ter) | c.4456C>T | Exon 37 | Nonsense | - | HI | 1 | 0.000003977 | Exeter unpublished |
| p.(Arg1487AlafsTer26) | c.4459_4460del | Exon 37 | Frameshift | - | HI | 0 | 0 | ([Snider, et al., 2013](#_ENREF_356)) |
| p.(Arg1487Lys) | c.4460G>A | Exon 37 | Missense | Cytoplasmic | HI | 0 | 0 | ([Ohkubo, et al., 2005](#_ENREF_275)) |
| p.(Gln1488Ter) | c.4462C>T | Exon 37 | Nonsense | - | HI | 0 | 0 | ([Kapoor, et al., 2013](#_ENREF_188)) |
| p.(Gln1488Arg) | c.4463A>G | Exon 37 | Missense | Cytoplasmic | HI | 0 | 0 | Exeter unpublished |
| p.(Phe1490Ser) | c.4469T>C | Exon 37 | Missense | Cytoplasmic | HI | 0 | 0 | ([Ni, et al., 2019](#_ENREF_268)) |
| p.(Cys1491AlafsTer7) | c.4471del | Exon 37 | Frameshift | - | HI | 0 | 0 | Paris unpublished |
| p.(Ala1493Thr) | c.4477G>A | Exon 37 | Missense | Cytoplasmic | HI | 0 | 0 | ([Arya, et al., 2014b](#_ENREF_20); [Ismail, et al., 2012](#_ENREF_172); [Kassem, et al., 2001](#_ENREF_192)) |
| p.(Arg1494Trp) | c.4480C>T | Exon 37 | Missense | Cytoplasmic | HI | 4 | 0.00001591 | ([Arya, et al., 2014b](#_ENREF_20); [Bellanne-Chantelot, et al., 2010](#_ENREF_31); [Bendix, et al., 2018](#_ENREF_32); [de Lonlay-Debeney, et al., 1999](#_ENREF_79); [Faletra, et al., 2013a](#_ENREF_101); [Greer, et al., 2007](#_ENREF_138); [Henquin, et al., 2011](#_ENREF_151); [Ismail, et al., 2011](#_ENREF_173); [Sandal, et al., 2009](#_ENREF_329); [Tornovsky, et al., 2004](#_ENREF_392); [Verkarre, et al., 1998](#_ENREF_400)) |
| p.(Arg1494Gln) | c.4481G>A | Exon 37 | Missense | Cytoplasmic | HI | 1 | 0.000003537 | ([Bellanne-Chantelot, et al., 2010](#_ENREF_31); [Darendeliler, et al., 2002](#_ENREF_76); [Glaser, et al., 1999](#_ENREF_127); [Henquin, et al., 2011](#_ENREF_151); [Sang, et al., 2014a](#_ENREF_331); [Suchi, et al., 2003](#_ENREF_364); [Suchi, et al., 2006](#_ENREF_365)) |
| p.(Ser1501Arg) | c.4503C>A | Exon 37 | Missense | Cytoplasmic | TNDM  Later-onset diabetes | 0 | 0 | ([Artuso, et al., 2015](#_ENREF_18))  Exeter unpublished* |
| p.(Met1505Thr) | c.4514T>C | Exon 37 | Missense | Cytoplasmic | Later-onset diabetes | 3 | 0.00001194 | Paris unpublished |
| p.(Asp1506Asn) | c.4516G>A | Exon 37 | Missense | Cytoplasmic | HI progressed to diabetes | 0 | 0 | Paris unpublished |
| p.(Asp1506His) | c.4516G>C | Exon 37 | Missense | Cytoplasmic | HI | 0 | 0 | ([Sang, et al., 2014a](#_ENREF_331)) |
| p.(Asp1506Glu) | c.4518C>A | Exon 37 | Missense | Cytoplasmic | HI | 0 | 0 | ([Flanagan, et al., 2011](#_ENREF_111); [Nessa, et al., 2015](#_ENREF_264)) |
| p.(Asp1506Glu) | c.4518C>G | Exon 37 | Missense | Cytoplasmic | HI | 0 | 0 | ([Bellanne-Chantelot, et al., 2010](#_ENREF_31); [Saint-Martin, et al., 2015](#_ENREF_325)) |
| p.(Glu1507_Asp1513dup) | c.4519_4539dup | Exon 37 | In frame duplication | Cytoplasmic | HI | 0 | 0 | Chicago unpublished |
| p.(Glu1507Lys) | c.4519G>A | Exon 37 | Missense | Cytoplasmic | HI,  Later-onset diabetes | 0 | 0 | ([Aguilar-Bryan and Bryan, 1999](#_ENREF_7); [Arya, et al., 2014b](#_ENREF_20); [Cai, et al., 2016](#_ENREF_50); [De Marinis, et al., 2016](#_ENREF_80); [Faletra, et al., 2013a](#_ENREF_101); [Fan, et al., 2015](#_ENREF_103); [Giurgea, et al., 2004](#_ENREF_125); [Huopio, et al., 2002](#_ENREF_157); [Huopio, et al., 2003](#_ENREF_159); [Huopio, et al., 2000](#_ENREF_160); [Minute, et al., 2015](#_ENREF_252); [Ortiz and Bryan, 2015](#_ENREF_280); [Pinney, et al., 2008](#_ENREF_299); [Saint-Martin, et al., 2011](#_ENREF_324); [Shimomura, et al., 2013](#_ENREF_349); [Vieira, et al., 2010](#_ENREF_401); [Yorifuji, et al., 2011](#_ENREF_417)) |
| p.(Glu1507Gln) | c.4519G>C | Exon 37 | Missense | Cytoplasmic | TNDM | 0 | 0 | ([Ortiz and Bryan, 2015](#_ENREF_280); [Ortiz, et al., 2013](#_ENREF_281)) |
| p.(Glu1507Ter) | c.4519G>T | Exon 37 | Nonsense |  | HI | 0 | 0 | ([Martinez, et al., 2016](#_ENREF_243)) |
| p.(Glu1507Gly) | c.4520A>G | Exon 37 | Missense | Cytoplasmic | TNDM | 0 | 0 | ([Mannikko, et al., 2011a](#_ENREF_237)) |
| p.(Glu1507Asp) | c.4521G>T | Exon 37 | Missense | Cytoplasmic | TNDM | 0 | 0 | ([Mannikko, et al., 2011a](#_ENREF_237); [Ortiz and Bryan, 2015](#_ENREF_280)) |
| p.(Ala1508Pro) | c.4522G>C | Exon 37 | Missense | Cytoplasmic | HI | 0 | 0 | ([Arya, et al., 2014b](#_ENREF_20); [Kapoor, et al., 2011](#_ENREF_190)) |
| p.(Ala1510_Ser1511dup) | c.4527_4532dup | Exon 37 | Duplication |  | HI | 0 | 0 | ([Nestorowicz, et al., 1998](#_ENREF_265); [Shemer, et al., 2012](#_ENREF_342)) |
| p.(Ile1512Ser) | c.4535T>G | Exon 37 | Missense | Cytoplasmic | HI | 0 | 0 | ([Saint-Martin, et al., 2015](#_ENREF_325)) |
| p.(Ile1512Thr) | c.4535T>C | Exon 37 | Missense | Cytoplasmic | HI | 0 | 0 | ([Pinney, et al., 2008](#_ENREF_299); [Salomon-Estebanez, et al., 2016](#_ENREF_328)) |
| p.(Met1514Lys) | c.4541T>A | Exon 37 | Missense | Cytoplasmic | HI | 0 | 0 | ([Flanagan, et al., 2011](#_ENREF_111)) |
| p.(Thr1516Ala) | c.4546A>G | Exon 37 | Missense | Cytoplasmic | HI | 0 | 0 | ([Bennett, et al., 2015](#_ENREF_33); [Martinez, et al., 2016](#_ENREF_243)) |
| p.(Thr1516Met) | c.4547C>T | Exon 37 | Missense | Cytoplasmic | HI | 1 | 0.000003983 | ([Banerjee, et al., 2011](#_ENREF_27)) |
| p.? | c.4548+1G>C | Intron 37 | Aberrant splicing |  | HI | 0 | 0 | Odense unpublished |
| p.? | c.4549-1G>A | Intron 37 | Aberrant splicing |  | HI | 0 | 0 | ([Snider, et al., 2013](#_ENREF_356)) |
| p.(Glu1517Lys) | c.4549G>A | Exon 38 | Missense | Cytoplasmic | HI | 0 | 0 | ([Boodhansingh, et al., 2019](#_ENREF_40); [Macmullen, et al., 2011](#_ENREF_230)) |
| p.(Leu1520Pro) | c.4559T>C | Exon 38 | Missense | Cytoplasmic | HI | 0 | 0 | ([Greer, et al., 2007](#_ENREF_138)) |
| p.(Val1523Met) | c.4567G>A | Exon 38 | Missense | Cytoplasmic | TNDM,  Later-onset diabetes* | 0 | 0 | ([Iafusco, et al., 2012](#_ENREF_162))  Paris unpublished* |
| p.(Val1523Leu) | c.4567G>C | Exon 38 | Missense | Cytoplasmic | PNDM | 0 | 0 | ([Ellard, et al., 2007](#_ENREF_98)) |
| p.(Val1523Leu) | c.4567G>T | Exon 39 | Missense | Cytoplasmic | Later-onset diabetes | 0 | 0 | ([Bowman, et al., 2012](#_ENREF_42)) |
| p.(Val1523Ala) | c.4568T>C | Exon 38 | Missense | Cytoplasmic | PNDM | 0 | 0 | ([Ellard, et al., 2007](#_ENREF_98); [Shah, et al., 2012a](#_ENREF_336)) |
| p.(Val1524Met) | c.4570G>A | Exon 38 | Missense | Cytoplasmic | TNDM  PNDM  Later-onset diabetes | 0 | 0 | ([Carmody, et al., 2014](#_ENREF_54); [Vaxillaire, et al., 2007](#_ENREF_396)) |
| p.(Phe1528LeufsTer13) | c.4578_4590del | Exon 38 | Frameshift |  | HI | 0 | 0 | ([Yorifuji, et al., 2011](#_ENREF_417)) |
| p.(Thr1532Ala) | c.4594A>G | Exon 38 | Missense | Cytoplasmic | HI | 0 | 0 | ([Ellard, et al., 2013](#_ENREF_99); [Sandal, et al., 2009](#_ENREF_329)) |
| p.(Thr1535Pro) | c.4603A>C | Exon 38 | Missense | Cytoplasmic | HI | 0 | 0 | ([Snider, et al., 2013](#_ENREF_356)) |
| p.(Ala1537Pro) | c.4609G>C | Exon 38 | Missense | Cytoplasmic | PNDM | 0 | 0 | ([Taberner, et al., 2016](#_ENREF_369)) |
| p.(Ala1537Val) | c.4610C>T | Exon 38 | Missense | Cytoplasmic | HI | 2 | 0.000007957 | ([Kapoor, et al., 2011](#_ENREF_190)) |
| p.(Ala1537=) | c.4611G>A | Exon 38 | Missense | Cytoplasmic | HI | 0 | 0 | ([Park, et al., 2016](#_ENREF_287)) |
| p.? | c.4611+4A>G | Intron 38 | Aberrant splicing |  | HI | 0 | 0 | Paris unpublished |
| p.? | c.4612-1G>T | Intron 38 | Aberrant splicing |  | HI | 0 | 0 | ([Han, et al., 2016](#_ENREF_144)) |
| p.? | c.4612-2A>T | Intron 38 | Aberrant splicing |  | HI | 0 | 0 | ([Bellanne-Chantelot, et al., 2010](#_ENREF_31); [Fernandez-Marmiesse, et al., 2006](#_ENREF_106); [Martinez, et al., 2016](#_ENREF_243)) |
| p.? | c.4612-2A>G | Intron 38 | Aberrant splicing |  | HI | 0 | 0 | ([Darendeliler, et al., 2002](#_ENREF_76)) |
| p.(Arg1539Ter) | c.4615C>T | Exon 39 | Nonsense |  | HI | 0 | 0 | Paris unpublished |
| p.(Arg1539Gln) | c.4616G>A | Exon 39 | Missense | Cytoplasmic | HI  Later-onset diabetes* | 0 | 0 | ([Kapoor, et al., 2011](#_ENREF_190); [Park, et al., 2011](#_ENREF_288); [Pinney, et al., 2008](#_ENREF_299); [Salomon-Estebanez, et al., 2016](#_ENREF_328))  Paris unpublished* |
| p.(Val1540Met) | c.4618G>A | Exon 39 | Missense | Cytoplasmic | TNDM | 0 | 0 | Exeter unpublished |
| p.(Thr1542HisfsTer18) | c.4622_4623insT | Exon 39 | Frameshift |  | HI | 0 | 0 | ([Fernandez-Marmiesse, et al., 2006](#_ENREF_106)) |
| p.(Leu1544Pro) | c.4631T>C | Exon 39 | Missense | Cytoplasmic | HI | 3 | 0.00001195 | ([Aguilar-Bryan and Bryan, 1999](#_ENREF_7); [Bennett, et al., 2010](#_ENREF_34); [Greer, et al., 2007](#_ENREF_138); [Taschenberger, et al., 2002](#_ENREF_382)) |
| p.(Val1551Asp) | c.4652T>A | Exon 39 | Missense | Cytoplasmic | HI | 1 | 0.00000398 | ([Huopio, et al., 2002](#_ENREF_157); [Reimann, et al., 2003](#_ENREF_315)) |
| p.(Leu1552Val) | c.4654C>G | Exon 39 | Missense | Cytoplasmic | HI | 1 | 0.00000398 | ([Huopio, et al., 2002](#_ENREF_157); [Reimann, et al., 2003](#_ENREF_315)) |
| p.(Arg1554Gln) | c.4661G>A | Exon 39 | Missense | Cytoplasmic | PNDM  TNDM  Later-onset diabetes | 0 | 0 | ([Madani, 2019](#_ENREF_231)) |
| p.(Gly1555Ser) | c.4663G>A | Exon 39 | Missense | Cytoplasmic | HI | 0 | 0 | ([Fournet, et al., 2001](#_ENREF_117)) |
| p.(Gly1555Asp) | c.4664G>A | Exon 39 | Missense | Cytoplasmic | HI | 0 | 0 | ([Gong, et al., 2016](#_ENREF_136)) |
| p.(Gly1555Val) | c.4664G>T | Exon 39 | Missense | Cytoplasmic | HI | 1 | 0.000003979 | ([Galcheva, et al., 2017](#_ENREF_121)) |
| p.(Glu1559Ter) | c.4675G>T | Exon 39 | Nonsense |  | HI | 0 | 0 | Exeter unpublished |
| p.(Lys1562Ter) | c.4684A>T | Exon 39 | Nonsense |  | HI | 0 | 0 | ([Snider, et al., 2013](#_ENREF_356)) |
| p.(Pro1563Thr) | c.4687C>A | Exon 39 | Missense | Cytoplasmic | HI | 0 | 0 | ([Guven, et al., 2016](#_ENREF_142); [Senniappan, et al., 2014](#_ENREF_334)) |
| p.(Pro1563Ala) | c.4687C>G | Exon 39 | Missense | Cytoplasmic | HI | 0 | 0 | ([Faletra, et al., 2013a](#_ENREF_101)) |
| p.(Ser1572Arg) | c.4716C>A | Exon 39 | Missense | Cytoplasmic | HI | 0 | 0 | Paris unpublished |
| p.(Ala1575Pro) | c.4723G>C | Exon 39 | Missense | Cytoplasmic | HI | 0 | 0 | ([Martinez, et al., 2016](#_ENREF_243)) |
| p.(Arg1579GlnfsTer31) | c.4734_4737del | Exon 39 | Frameshift |  | HI | 0 | 0 | Paris unpublished |

**Supp Table S5: Variants of uncertain clinical significance identified in *ABCC8* (NM_001287174.1)*.*** NDM = Neonatal Diabetes. PNDM = Permanent Neonatal Diabetes Mellitus. TNDM = Transient Neonatal Diabetes Mellitus. HI= Hyperinsulinism. * in the Phenotype column highlights a new phenotype; the * in the Reference column indicates which laboratory has identified the variant in a patients with the new phenotype.

| **Protein change** | **Nucleotide Position** | **Position** | **Mutation Type** | **Protein domain (UniProt)** | **Phenotype** | **GnomAD AC** | **GnomAD MAF** | **Reference** |
| --- | --- | --- | --- | --- | --- | --- | --- | --- |
| p.? | c.-1215T>C | Promoter | Upstream substitution |  | HI | 0 | 0 | ([Snider, et al., 2013](#_ENREF_356)) |
| p.? | c.-827G>T | Promoter | Upstream substitution |  | HI | 0 | 0 | ([Snider, et al., 2013](#_ENREF_356)) |
| p.? | c.-565_-564insACC | Promoter | Upstream insertion |  | HI | 0 | 0 | ([Snider, et al., 2013](#_ENREF_356)) |
| p.? | c.-548_-547insAAA | Promoter | Upstream insertion |  | HI | 0 | 0 | ([Snider, et al., 2013](#_ENREF_356)) |
| p.(Ala14Ser) | c.40G>T | Exon 1 | Missense | Extracellular | Diabetes | 0 | 0 | Paris unpublished |
| p.(Tyr15Phe) | c.44A>T | Exon 1 | Missense | Extracellular | HI | 0 | 0 | Paris unpublished |
| p.(Asn23His) | c.67A>C | Exon 1 | Missense | Extracellular | NDM | 0 | 0 | ([Klupa, et al., 2009](#_ENREF_203)) |
| p.(Phe41Leu) | c.121T>C | Exon 1 | Missense | Transmembrane | Diabetes | 0 | 0 | Paris unpublished |
| p.(His59Asn) | c.175C>A | Exon 2 | Missense | Cytoplasmic | HI | 0 | 0 | Paris unpublished |
| p.(Gly97=) | c.291G>T | Exon 3 | Missense |  | Diabetes | 0 | 0 | Paris unpublished |
| p.(Glu100Lys) | c.298G>A | Exon 3 | Missense | Extracellular | MODY | 18 | 0.00007162 | ([Bowman, et al., 2012](#_ENREF_42)) |
| p.(Val121Met) | c.361G>A | Exon 3 | Missense | Transmembrane | Diabetes | 0 | 0 | Paris unpublished |
| p.(Val121Ala) | c.362T>C | Exon 3 | Missense | Transmembrane | NDM | 0 | 0 | Chicago unpublished |
| p.(His125Gln) | c.375C>G | Exon 3 | Missense | Cytoplasmic | HI | 23 | 0.00008132 | ([Nestorowicz, et al., 1998](#_ENREF_265); [Shyng, et al., 1998](#_ENREF_350)) |
| p.(Ile127Thr) | c.380T>C | Exon 3 | Missense | Cytoplasmic | Diabetes | 0 | 0 | Paris unpublished |
| p.(Ile137Ser) | c.410T>G | Exon 3 | Missense | Transmembrane | Diabetes | 0 | 0 | Paris unpublished |
| p.? | c.412+18G>A | Intron 3 | Intronic substitution |  | HI | 0 | 0 | ([Nestorowicz, et al., 1998](#_ENREF_265)) |
| p.? | c.413-48A>G | Intron 3 | Intronic substitution |  | HI | 13 | 0.00006792 | ([Bellanne-Chantelot, et al., 2010](#_ENREF_31)) |
| p.? | c.580-16_580-14del | Intron 4 | Intronic deletion |  | Diabetes | 5 | 0.00001776 | Paris unpublished |
| p.(Arg194Lys) | c.581G>A | Exon 5 | Missense | Transmembrane | Diabetes | 0 | 0 | Paris unpublished |
| p.(Pro201Leu) | c.602C>T | Exon 5 | Missense | Cytoplasmic | HI | 0 | 0 | Paris unpublished |
| p.(Leu224=) | c.670C>T | Exon 5 | Synonymous |  | HI | 0 | 0 | ([Snider, et al., 2013](#_ENREF_356)) |
| p.(Ala240Thr) | c.718G>A | Exon 5 | Missense | Cytoplasmic | HI | 0 | 0 | Paris unpublished |
| p.(Arg248Gln) | c.743G>A | Exon 5 | Missense | Cytoplasmic | Diabetes | 4 | 0.00001591 | ([Tarasov, et al., 2008](#_ENREF_381)) |
| p.(Ile250=) | c.750C>T | Exon 5 | Synonymous |  | Diabetes | 5 | 0.00001768 | ([Ohta, et al., 1998](#_ENREF_276)) |
| p.(Met257Leu) | c.769A>C | Exon 5 | Missense | Cytoplasmic | Diabetes | 1 | 0.000003976 | Paris unpublished |
| p.(Met257Thr) | c.770T>C | Exon 5 | Missense | Cytoplasmic | Diabetes | 0 | 0 | Paris unpublished |
| p.(Glu268Lys) | c.802G>A | Exon 5 | Missense | Cytoplasmic | HI | 1 | 0.000003979 | ([Snider, et al., 2013](#_ENREF_356)) |
| p.(Ala269Asp) | c.806C>A | Exon 5 | Missense | Cytoplasmic | Diabetes | 3 | 0.00001061 | ([Vaxillaire, et al., 2007](#_ENREF_396)) |
| p.(Phe270Cys) | c.809T>G | Exon 5 | Missense | Cytoplasmic | Diabetes | 0 | 0 | Paris unpublished |
| p.(Arg275Gln) | c.824G>A | Exon 6 | Missense | Cytoplasmic | NDM | 61 | 0.0002214 | ([Ohta, et al., 1998](#_ENREF_276)) |
| p.(Arg285Gln) | c.854G>A | Exon 6 | Missense | Cytoplasmic | NDM | 33 | 0.0001182 | ([Stoy, et al., 2008](#_ENREF_362)) |
| p.(His293Pro) | c.878A>C | Exon 6 | Missense | Cytoplasmic | HI | 0 | 0 | Chicago unpublished |
| p.(Arg306Cys) | c.916C>T | Exon 6 | Missense | Cytoplasmic | HI | 7 | 0.00002792 | ([Snider, et al., 2013](#_ENREF_356)) |
| p.(Ala309=) | c.927C>T | Exon 6 | Synonymous |  | HI | 9 | 0.00003188 | ([Snider, et al., 2013](#_ENREF_356)) |
| p.(Ala315=) | c.945C>T | Exon 6 | Synonymous | Transmembrane | HI | 135 | 0.0004779 | ([Nestorowicz, et al., 1996](#_ENREF_267)) |
| p.(Gly316Glu) | c.947G>A | Exon 6 | Missense | Transmembrane | HI | 0 | 0 | Chicago unpublished |
| p.(Gly342Arg) | c.1024G>A | Exon 7 | Missense | Extracellular | Diabetes | 4 | 0.00001591 | Paris unpublished |
| p.(Ala355Thr) | c.1063G>A | Exon 7 | Missense | Extracellular | HI NDM | 135 | 0.0004773 | ([Ismail, et al., 2011](#_ENREF_173); [Mohnike, et al., 2014](#_ENREF_256); [Russo, et al., 2011b](#_ENREF_321); [Salomon-Estebanez, et al., 2016](#_ENREF_328); [Snider, et al., 2013](#_ENREF_356)) |
| p.(Val357Ile) | c.1069G>A | Exon 7 | Missense | Transmembrane | HI | 8 | 0.00003181 | Odense unpublished  Paris Unpublished |
| p.(Ile395Phe) | c.1183A>T | Exon 8 | Missense | Cytoplasmic | NDM | 2 | 0.000007953 | Chicago unpublished |
| p.(His401Tyr) | c.1201C>T | Exon 8 | Missense | Cytoplasmic | NDM Diabetes | 0 | 0 | ([Deeb, et al., 2016](#_ENREF_85); [Rubio-Cabezas, et al., 2012](#_ENREF_319)) |
| p.(Thr413Ser) | c.1238C>G | Exon 8 | Missense | Cytoplasmic | Diabetes | 0 | 0 | Exeter unpublished |
| p.(Asp424Gly) | c.1271A>G | Exon 9 | Missense | Cytoplasmic | PNDM | 0 | 0 | Paris unpublished |
| p.(Asn426Ser) | c.1277A>G | Exon 8 | Missense | Cytoplasmic | NDM | 1 | 0.000003976 | ([Bennett, et al., 2015](#_ENREF_33)) |
| p.(Gln427=) | c.1281G>A | Exon 8 | Synonymous |  | HI | 3 | 0.00001193 | ([Snider, et al., 2013](#_ENREF_356)) |
| p.? | c.1333-28G>A | Intron 8 | Intronic substitution |  | HI | 0 | 0 | ([Tanizawa, et al., 2000](#_ENREF_378)) |
| p.(Ile446Thr) | c.1337T>C | Exon 9 | Missense | Transmembrane | Diabetes | 3 | 0.00001194 | Paris unpublished |
| p.(Gly457Arg) | c.1369G>A | Exon 9 | Missense | Extracellular | Diabetes | 13 | 0.00004598 | Paris unpublished |
| p.(Arg504Cys) | c.1510C>T | Exon 10 | Missense | Cytoplasmic | Diabetes | 2 | 0.000007969 | Paris unpublished |
| p.(Gly505Cys) | c.1513G>T | Exon 10 | Missense | Cytoplasmic | HI | 0 | 0 | Paris unpublished |
| p.(Ala513Thr) | c.1537G>A | Exon 10 | Missense | Cytoplasmic | Diabetes | 13 | 0.00004601 | Paris unpublished |
| p.(Arg521Trp) | c.1561C>T | Exon 10 | Missense | Cytoplasmic | Diabetes | 7 | 0.00002787 | Chicago unpublished  Paris unpublished |
| p.(Arg521Gln) | c.1562G>A | Exon 10 | Missense | Cytoplasmic | HI  Later-onset diabetes* | 27 | 0.00009556 | ([Calabria, et al., 2012](#_ENREF_51); [Snider, et al., 2013](#_ENREF_356))  Paris unpublished* |
| p.(Val522Met) | c.1564G>A | Exon 10 | Missense | Cytoplasmic | Diabetes | 2 | 0.000007078 | Paris unpublished |
| p.(Ala537Thr) | c.1609G>A | Exon 10 | Missense | Cytoplasmic | HI | 0 | 0 | Paris unpublished |
| p.(Thr540=) | c.1620C>T | Exon 10 | Synonymous |  | Diabetes | 5 | 0.00001769 | ([Ohta, et al., 1998](#_ENREF_276)) |
| p.? | c.1630+10C>T | Intron 10 | Intronic substitution |  | HI | 71 | 0.0002512 | ([Snider, et al., 2013](#_ENREF_356)) |
| p.? | c.1672-99T>C | Intron 11 | Intronic substitution |  | HI | 0 | 0 | ([Snider, et al., 2013](#_ENREF_356)) |
| p.? | c.1672-98G>A | Intron 11 | Intronic substitution |  | HI | 0 | 0 | ([Snider, et al., 2013](#_ENREF_356)) |
| p.(Glu568=) | c.1704G>A | Exon 12 | Synonymous |  | HI | 24 | 0.00008488 | ([Snider, et al., 2013](#_ENREF_356)) |
| p.(Val575Met) | c.1723G>A | Exon 12 | Missense | Extracellular | Diabetes | 4 | 0.00001591 | Paris unpublished |
| p.? | c.1818-7C>T | Intron 12 | Intronic substitution |  | Diabetes  HI | 12 | 0.00004281 | ([Ohta, et al., 1998](#_ENREF_276); [Someya, et al., 2000](#_ENREF_358)) |
| p.(Phe613Leu) | c.1837T>C | Exon 13 | Missense | Cytoplasmic | Diabetes | 0 | 0.00000000 | Paris unpublished |
| p.(Ala625Val) | c.1874C>T | Exon 13 | Missense | Cytoplasmic | HI | 60 | 0.0002124 | ([Snider, et al., 2013](#_ENREF_356)) |
| p.? | c.1924-44A>G | Intron 13 | Intronic substitution |  | HI | 0 | 0 | Odense unpublished |
| p.(Cys656Phe) | c.1967G>T | Exon 14 | Missense | Cytoplasmic | Diabetes | 1 | 0.000003984 | Paris unpublished |
| p.(Gly672=) | c.2016C>T | Exon 14 | Synonymous |  | HI | 8 | 0.00002832 | ([Snider, et al., 2013](#_ENREF_356)) |
| p.(Asp673Asn) | c.2017G>A | Exon 14 | Missense | Cytoplasmic | HI | 7 | 0.00002478 | ([Hansen, et al., 1998](#_ENREF_145); [Someya, et al., 2000](#_ENREF_358)) |
| p.? | c.2040+241A>G | Intron 14 | Intronic substitution |  | HI | 0 | 0 | ([Snider, et al., 2013](#_ENREF_356)) |
| p.? | c.2040+120C>T | Intron 14 | Intronic substitution |  | HI | 4 | 0.0001274 | ([Snider, et al., 2013](#_ENREF_356)) |
| p.? | c.2041-12C>T | Intron 14 | Intronic substitution |  | HI | 137 | 0.0004911 | ([Snider, et al., 2013](#_ENREF_356)) |
| p.(Thr695Ala) | c.2083A>G | Exon 15 | Missense | Cytoplasmic | Not diabetic | 0 | 0 | ([Bonnefond, et al., 2014](#_ENREF_39)) |
| p.(Arg702Cys) | c.2104C>T | Exon 15 | Missense | Cytoplasmic | Diabetes | 22 | 0.00008768 | Paris unpublished |
| p.? | c.2116+61A>G | Intron 15 | Intronic substitution |  | Diabetes | 1 | 0.00003187 | Paris unpublished |
| p.(Ala726Thr) | c.2176G>A | Exon 16 | Missense | Cytoplasmic | HI | 132 | 0.0004671 | ([Banerjee, et al., 2011](#_ENREF_27); [Mohnike, et al., 2014](#_ENREF_256)) |
| p.(Gln731Glu) | c.2191C>G | Exon 16 | Missense | Cytoplasmic | HI | 4 | 0.00001415 | Paris unpublished |
| p.? | c.2259-81G>A | Intron 17 | Intronic substitution |  | HI | 0 | 0 | ([Snider, et al., 2013](#_ENREF_356)) |
| p.? | c.2259-44T>G | Intron 17 | Intronic substitution |  | HI | 0 | 0 | ([Fernandez-Marmiesse, et al., 2006](#_ENREF_106)) |
| p.(Trp739Cys) | c.2217G>T | Exon 18 | Missense | Cytoplasmic | HI | 3 | 0.00001194 | ([Brady, et al., 2015](#_ENREF_43); [Kapoor, et al., 2013](#_ENREF_188)) |
| p.(Arg756Gln) | c.2267G>A | Exon 18 | Missense | Cytoplasmic | Not diabetic | 18 | 0.00007396 | ([Bonnefond, et al., 2014](#_ENREF_39)) |
| p.? | c.2294+239_2294+240insTTTTCTT | Intron 18 | Intronic insertion |  | HI | 0 | 0 | ([Snider, et al., 2013](#_ENREF_356)) |
| p.(Val770Met) | c.2308G>A | Exon 19 | Missense | Cytoplasmic | HI | 5 | 0.00001989 | Paris unpublished |
| p.? | c.2394-7A>T | Intron 19 | Intronic substitution |  | HI | 0 | 0 | ([Someya, et al., 2000](#_ENREF_358)) |
| p.? | c.2394-22C>A | Intron 19 | Intronic substitution |  | Diabetes | 3 | 0.00001193 | ([Ohta, et al., 1998](#_ENREF_276)) |
| p.(Asp811Asn) | c.2431G>A | Exon 20 | Missense | Cytoplasmic | Diabetes | 1 | 0.000003976 | ([Ohta, et al., 1998](#_ENREF_276)) |
| p.? | c.2478+77A>G | Intron 20 | Intronic substitution |  | HI | 0 | 0 | ([Snider, et al., 2013](#_ENREF_356)) |
| p.(Ser831Thr) | c.2491T>A | Exon 21 | Missense | Cytoplasmic | Diabetes | 0 | 0.00000000 | Paris unpublished |
| p.(Arg835Cys) | c.2503C>T | Exon 21 | Missense | Cytoplasmic | Diabetes HI | 13 | 0.00005289 | ([Ohta, et al., 1998](#_ENREF_276); [Someya, et al., 2000](#_ENREF_358)) |
| p.(Arg835His) | c.2504G>A | Exon 21 | Missense | Cytoplasmic | Diabetes | 6 | 0.00002442 | Paris unpublished |
| p.(Ile838Val) | c.2512A>G | Exon 21 | Missense | Cytoplasmic | Diabetes | 0 | 0 | Paris unpublished |
| p.(Val840Ala) | c.2519T>C | Exon 21 | Missense | Cytoplasmic | Diabetes | 0 | 0 | Paris unpublished |
| p.(Asn849Thr) | c.2546A>C | Exon 21 | Missense | Cytoplasmic | Diabetes | 0 | 0 | Paris unpublished |
| p.(Leu860=) | c.2580G>T | Exon 22 | Synonymous | Cytoplasmic | HI | 0 | 0 | ([Someya, et al., 2000](#_ENREF_358)) |
| p.(Ile862Met) | c.2586C>G | Exon 22 | Missense | Cytoplasmic | HI | 3 | 0.00001193 | ([Snider, et al., 2013](#_ENREF_356)) |
| p.(His863Arg) | c.2588A>G | Exon 22 | Missense | Cytoplasmic | Diabetes | 2 | 0.000007953 | Paris unpublished Exeter unpublished |
| p.(Asp880Asn) | c.2638G>A | Exon 22 | Missense | Cytoplasmic | NDM | 16 | 0.00006362 | ([Masia, et al., 2007a](#_ENREF_245)) |
| p.(Arg934Gln) | c.2801G>A | Intron 23 | Missense | Cytoplasmic | HI | 3 | 0.00001193 | Paris unpublished |
| p.? | c.2823+19G>A | Intron 23 | Intronic substitution |  | HI | 10 | 0.00003557 | ([Snider, et al., 2013](#_ENREF_356)) |
| p.? | c.2823+35C>A | Intron 23 | Intronic substitution |  | HI | 0 | 0 | ([Snider, et al., 2013](#_ENREF_356)) |
| p.? | c.2924-19G>A | Intron 24 | Intronic substitution |  | HI | 80 | 0.0003031 | ([Snider, et al., 2013](#_ENREF_356)) |
| p.? | c.2924-51C>T | Intron 24 | Intronic substitution |  | HI | 16 | 0.00006287 | ([Bellanne-Chantelot, et al., 2010](#_ENREF_31)) |
| p.(Ala978Thr) | c.2932G>A | Exon 25 | Missense | Cytoplasmic | HI | 22 | 0.00008026 | ([Banerjee, et al., 2011](#_ENREF_27)) |
| p.(Ala1002Thr) | c.3004G>A | Exon 25 | Missense | Cytoplasmic | HI | 10 | 0.00003575 | Paris unpublished |
| p.(Ser1014=) | c.3042G>A | Exon 25 | Synonymous | Transmembrane | Diabetes | 61 | 0.0002166 | ([Ohta, et al., 1998](#_ENREF_276)) |
| p.(Ser1019Leu) | c.3056C>T | Exon 25 | Missense | Transmembrane | Diabetes HI | 2 | 0.000007983 | Paris unpublished |
| p.(Thr1038Asn) | c.3113C>A | Exon 25 | Missense | Extracellular | Diabetes | 0 | 0 | Paris unpublished |
| p.(Ser1054Asn) | c.3161G>A | Exon 25 | Missense | Extracellular | NDM | 1 | 0.000003985 | ([Russo, et al., 2011b](#_ENREF_321)) |
| p.(Thr1116=) | c.3348G>T | Exon 27 | Synonymous |  | Diabetes | 0 | 0 | ([Ohta, et al., 1998](#_ENREF_276)) |
| p.(Thr1139=) | c.3417G>A | Exon 28 | Synonymous | Transmembrane | HI | 6 | 0.00002466 | ([Snider, et al., 2013](#_ENREF_356)) |
| p.(Val1166Met) | c.3496G>A | Exon 28 | Missense | Transmembrane | Diabetes | 25 | 0.00008843 | Chicago unpublished  Paris unpublished |
| p.(Val1174Met) | c.3520G>A | Exon 28 | Missense | Transmembrane | Diabetes | 4 | 0.00001415 | ([Snider, et al., 2013](#_ENREF_356)) |
| p.? | c.3561-36G>A | Intron 28 | Intronic substitution |  | HI | 7 | 0.00002795 | ([Snider, et al., 2013](#_ENREF_356)) |
| p.? | c.3561-19A>C | Intron 28 | Intronic substitution |  | HI | 0 | 0 | Chicago unpublished |
| p.(Asp1194Val) | c.3581A>T | Exon 29 | Missense | Cytoplasmic | HI  Later-onset diabetes* | 15 | 0.00005303 | ([Arya, et al., 2014b](#_ENREF_20); [Muzyamba, et al., 2007](#_ENREF_260))  Paris unpublished* |
| p.(Glu1209Lys) | c.3625G>A | Exon 29 | Missense | Cytoplasmic | HI | 0 | 0 | Paris unpublished |
| p.(Phe1217Leu) | c.3651C>G | Exon 29 | Missense | Cytoplasmic | TNDM | 0 | 0.00000000 | Paris unpublished |
| p.? | c.3653+4C>G | Intron 29 | Intronic substitution |  | Later-onset diabetes HI* | 41 | 0.0001449 | Paris unpublished  Exeter unpublished* |
| p.(Ala1221=) | c.3663C>T | Exon 30 | Synonymous |  | HI | 0 | 0 | ([Snider, et al., 2013](#_ENREF_356)) |
| p.(Leu1241Arg) | c.3722T>G | Exon 30 | Missense | Cytoplasmic | HI | 0 | 0 | Paris unpublished |
| p.(Glu1249Ala) | c.3746A>C | Exon 30 | Missense | Cytoplasmic | HI | 0 | 0 | Paris unpublished |
| p.? | c.3757-37C>T | Intron 30 | Intronic substitution |  | HI | 49 | 0.0001791 | ([Snider, et al., 2013](#_ENREF_356)) |
| p.(Glu1253Gly) | c.3758A>G | Exon 31 | Missense | Transmembrane | HI | 0 | 0 | Chicago unpublished |
| p.(Val1260Met) | c.3778G>A | Exon 31 | Missense | Transmembrane | Diabetes | 15 | 0.00005321 | Paris unpublished |
| p.(Ala1264=) | c.3792G>A | Exon 31 | Synonymous | Transmembrane | HI | 9 | 0.00003592 | ([Snider, et al., 2013](#_ENREF_356)) |
| p.? | c.3870+7G>A | Intron 31 | Intronic substitution |  | HI | 92 | 0.0003312 | ([Banerjee, et al., 2011](#_ENREF_27)) |
| p.? | c.3871-60G>C | Intron 31 | Intronic substitution |  | HI | 9 | 0.0002868 | ([Snider, et al., 2013](#_ENREF_356)) |
| p.(Glu1327Lys) | c.3979G>A | Exon 32 | Missense | Cytoplasmic | Diabetes HI | 41 | 0.0001452 | ([Ellard, et al., 2007](#_ENREF_98)) ([Bennett, et al., 2015](#_ENREF_33); [Du, et al., 2019](#_ENREF_92)) |
| p.? | c.3992-71G>T | Intron 32 | Intronic substitution |  | Diabetes | 0 | 0 | ([Ohta, et al., 1998](#_ENREF_276)) |
| p.? | c.3992-10C>T | Intron 32 | Intronic substitution |  | HI | 105 | 0.0004177 | Odense unpublished |
| p.(Pro1360=) | c.4080G>A | Exon 33 | Synonymous |  | HI | 8 | 0.00003182 | ([Snider, et al., 2013](#_ENREF_356)) |
| p.(Ile1347_Gln1348del) | c.4040_4045del | Exon 33 | In-frame deletion | Cytoplasmic | HI | 0 | 0 | ([Bellanne-Chantelot, et al., 2010](#_ENREF_31); [Damaj, et al., 2008](#_ENREF_75)) |
| p.(Val1361Gly) | c.4082T>G | Exon 33 | Missense | Cytoplasmic | HI | 0 | 0 | ([Nestorowicz, et al., 1996](#_ENREF_267)) |
| p.? | c.4123-53T>A | Intron 33 | Intronic substitution |  | HI | 0 | 0 | ([Snider, et al., 2013](#_ENREF_356)) |
| p.? | c.4123-25T>C | Intron 33 | Intronic substitution |  | HI Diabetes | 0 | 0 | ([Hansen, et al., 1998](#_ENREF_145); [Ohkubo, et al., 2005](#_ENREF_275); [Ohta, et al., 1998](#_ENREF_276); [Tanizawa, et al., 2000](#_ENREF_378)) |
| p.? | c.4123-17T>C | Intron 33 | Intronic substitution |  | Diabetes | 0 | 0 | Chicago unpublished |
| p.? | c.4123-16C>T | Intron 33 | Intronic substitution |  | HI | 0 | 0 | ([Ohkubo, et al., 2005](#_ENREF_275); [Ohta, et al., 1998](#_ENREF_276); [Tanizawa, et al., 2000](#_ENREF_378)) |
| p.? | c.4201+23del | Intron 34 | Intronic deletion |  | HI | 0 | 0 | ([Bellanne-Chantelot, et al., 2010](#_ENREF_31)) |
| p.? | c.4201+71C>T | Intron 34 | Intronic substitution |  | HI | 0 | 0 | ([Bellanne-Chantelot, et al., 2010](#_ENREF_31)) |
| p.(Pro1414=) | c.4242G>A | Exon 35 | Synonymous |  | Diabetes | 10 | 0.00005929 | ([Jahnavi, et al., 2013](#_ENREF_176)) |
| p.(Arg1419Cys) | c.4255C>T | Exon 35 | Missense | Cytoplasmic | HI | 0 | 0 | ([Bellanne-Chantelot, et al., 2010](#_ENREF_31); [Giurgea, et al., 2004](#_ENREF_125)) |
| p.(Ser1423Phe) | c.4268C>T | Exon 35 | Missense | Cytoplasmic | HI | 0 | 0 | Paris unpublished |
| p.(Gln1427Lys) | c.4279C>A | Exon 35 | Missense | Cytoplasmic | Diabetes | 0 | 0 | Paris unpublished |
| p.? | c.4310+62G>A | Intron 35 | Intronic substitution |  | HI | 1 | 0.0000318 | ([Nestorowicz, et al., 1996](#_ENREF_267)) |
| p.(Asn1439=) | c.4317C>T | Exon 36 | Synonymous |  | HI | 3 | 0.00001458 | Paris unpublished |
| p.(Pro1442Leu) | c.4325C>T | Exon 36 | Missense | Cytoplasmic | HI | 0 | 0 | Paris unpublished |
| p.(Trp1452Arg) | c.4354T>C | Exon 36 | Missense | Cytoplasmic | HI | 0 | 0 | ([Henquin, et al., 2011](#_ENREF_151)) |
| p.(Ile1457=) | c.4371C>T | Exon 36 | Synonymous |  | Diabetes | 43 | 0.0001917 | ([Jahnavi, et al., 2013](#_ENREF_176)) |
| p.(Ala1458Pro) | c.4372G>C | Exon 36 | Missense | Cytoplasmic | HI | 0 | 0 | ([Bellanne-Chantelot, et al., 2010](#_ENREF_31)) |
| p.? | c.4415-148A>G | Intron 36 | Intronic substitution |  | HI | 0 | 0 | ([Snider, et al., 2013](#_ENREF_356)) |
| p.(Gly1478=) | c.4434C>T | Exon 37 | Synonymous |  | HI | 48 | 0.0001697 | Chicago unpublished |
| p.(Arg1494Gly) | c.4480C>G | Exon 37 | Missense | Cytoplasmic | Diabetes | 0 | 0 | ([Ang, et al., 2016](#_ENREF_14)) |
| p.(Ala1495=) | c.4485C>T | Exon 37 | Synonymous |  | HI | 63 | 0.0002228 | Chicago unpublished |
| p.(Val1497Met) | c.4489G>A | Exon 37 | Missense | Cytoplasmic | HI | 2 | 0.000007957 | Paris unpublished  Odense unpublished |
| p.(Ile1504Asn) | c.4511T>A | Exon 37 | Missense | Cytoplasmic | Diabetes | 0 | 0 | Paris unpublished |
| p.? | c.4548+9T>C | Intron 37 | Intronic substitution |  | HI | 3 | 0.00001196 | ([Bellanne-Chantelot, et al., 2010](#_ENREF_31)) |
| p.(Arg1531His) | c.4592G>A | Exon 38 | Missense | Cytoplasmic | Diabetes | 3 | 0.00001061 | Chicago unpublished |
| p.(Val1534Leu) | c.4600G>C | Exon 38 | Missense | Cytoplasmic | Diabetes | 0 | 0 | Paris unpublished |
| p.? | c.4612-100_4612-99insC | Intron 38 | Intronic insertion |  | Diabetes | 0 | 0 | ([Hansen, et al., 1998](#_ENREF_145)) |
| p.? | c.4612-15T>A | Intron 38 | Intronic substitution |  | Diabetes | 0 | 0 | ([Ohta, et al., 1998](#_ENREF_276)) |
| p.(Lys1522Asn) | c.4566G>T | Exon 38 | Missense | Cytoplasmic | Diabetes HI | 114 | 0.0004033 | ([Snider, et al., 2013](#_ENREF_356); [Tarasov, et al., 2008](#_ENREF_381)) |
| p.(Ser1576Pro) | c.4726T>C | Exon 39 | Missense | Cytoplasmic | HI | 0 | 0 | Paris unpublished |
| p.(Arg1579His) | c.4736G>A | Exon 39 | Missense | Cytoplasmic | Diabetes | 14 | 0.00004952 | Paris unpublished |
| p.? | c.*6del | 3' UTR | 3' UTR deletion |  | HI | 0 | 0 | ([Fan, et al., 2015](#_ENREF_103)) |

**Supp Table S6: Benign variants identified in *ABCC8* (NM_001287174.1)*.*** (¥) denotes variants which have been reclassified as benign following publication as a result of their frequency in population data.

| **Protein change** | **Nucleotide Position** | **Position** | **GnomAD AC** | **GnomAD MAF** | **Reference** |
| --- | --- | --- | --- | --- | --- |
| p.? | c.-1224T>C | Promoter | 16097 | 0.5137 | ([Snider, et al., 2013](#_ENREF_356)) |
| p.? | c.-1061C>T | Promoter | 2635 | 0.08397 | ([Snider, et al., 2013](#_ENREF_356)) |
| p.? | c.-850G>A | Promoter | 1160 | 0.03696 | ([Snider, et al., 2013](#_ENREF_356)) |
| p.? | c.-825G>T | Promoter | 1169 | 0.03726 | ([Snider, et al., 2013](#_ENREF_356)) |
| p.? | c.-549_-547dup | Promoter | 11716 | 0.3780 | ([Snider, et al., 2013](#_ENREF_356)) |
| p.? | c.-72G>A | Promoter | 215 | 0.005277 | ([Snider, et al., 2013](#_ENREF_356)) |
| p.? | c.-49G>C | Promoter | 1260 | 0.01481 | ([Bellanne-Chantelot, et al., 2010](#_ENREF_31)) |
| p.? | c.-19A>G | Promoter | 737 | 0.00448 | ([Snider, et al., 2013](#_ENREF_356)) |
| p.? | c.-8G>T | Promoter | 455 | 0.002562 | ([Banerjee, et al., 2011](#_ENREF_27); [Proks, et al., 2006a](#_ENREF_307)) |
| p.(Asn24=) | c.72C>T | Exon 1 | 2 | 0.0000082 | ([Bellanne-Chantelot, et al., 2010](#_ENREF_31)) |
| p.(Val34=) | c.102G>A | Exon 1 | 274 | 0.0009893 | ([Snider, et al., 2013](#_ENREF_356)) |
| p.? | c.149-156T>C | Intron 1 | 1360 | 0.04336 | ([Snider, et al., 2013](#_ENREF_356)) |
| p.(Pro69=) | c.207T>C | Exon 2 | 133255 | 0.4719 | ([Fernandez-Marmiesse, et al., 2006](#_ENREF_106); [Hansen, et al., 1998](#_ENREF_145); [Nestorowicz, et al., 1998](#_ENREF_265); [Ohkubo, et al., 2005](#_ENREF_275); [Ohta, et al., 1998](#_ENREF_276); [Proks, et al., 2006a](#_ENREF_307); [Someya, et al., 2000](#_ENREF_358); [Tanizawa, et al., 2000](#_ENREF_378)) |
| p.? | c.291-142C>T | Intron 2 | 667 | 0.02126 | ([Snider, et al., 2013](#_ENREF_356)) |
| p.? | c.291-62G>T | Intron 2 | 2172 | 0.06922 | ([Bellanne-Chantelot, et al., 2010](#_ENREF_31)) |
| p.(Ala110=) | c.330C>T | Exon 3 | 13307 | 0.04707 | ([Fernandez-Marmiesse, et al., 2006](#_ENREF_106); [Gonen, et al., 2012](#_ENREF_135); [Nestorowicz, et al., 1998](#_ENREF_265)) |
| p.(Val118=) | c.354C>T | Exon 3 | 1355 | 0.004792 | ([Snider, et al., 2013](#_ENREF_356)) |
| p.? | c.412+77G>A | Intron 3 | 13667 | 0.4363 | ([Snider, et al., 2013](#_ENREF_356)) |
| p.(Val141=) | c.423G>A | Exon 4 | 1200 | 0.005278 | ([Fernandez-Marmiesse, et al., 2006](#_ENREF_106); [Nestorowicz, et al., 1998](#_ENREF_265)) |
| p.? | c.579+14C>T | Intron 4 | 100280 | 0.3988 | ([Fernandez-Marmiesse, et al., 2006](#_ENREF_106); [Nestorowicz, et al., 1998](#_ENREF_265); [Ohkubo, et al., 2005](#_ENREF_275)) |
| p.? | c.579+29G>C | Intron 4 | 1212 | 0.005027 | ([Nestorowicz, et al., 1998](#_ENREF_265)) |
| p.? | c.580-117G>A | Intron 4 | 429 | 0.01367 | ([Snider, et al., 2013](#_ENREF_356)) |
| p.? | c.822+20C>T | Intron 5 | 121 | 0.0004292 | ([Snider, et al., 2013](#_ENREF_356)) |
| p.? | c.823-107C>A | Intron 5 | 2550 | 0.08124 | ([Snider, et al., 2013](#_ENREF_356)) |
| p.? | c.823-106C>T | Intron 5 | 7222 | 0.2309 | ([Snider, et al., 2013](#_ENREF_356)) |
| p.? | c.823-105A>G | Intron 5 | 11602 | 0.3713 | ([Snider, et al., 2013](#_ENREF_356)) |
| p.? | c.823-49G>A | Intron 5 | 2218 | 0.008355 | ([Bellanne-Chantelot, et al., 2010](#_ENREF_31)) |
| p.? | c.1011+87A>G | Intron 6 | 11682 | 0.3729 | ([Snider, et al., 2013](#_ENREF_356)) |
| p.(Asn386=) | c.1158C>T | Exon 7 | 1219 | 0.004309 | ([Snider, et al., 2013](#_ENREF_356)) |
| p.? | c.1176+85C>T | Intron 7 | 398 | 0.01267 | ([Snider, et al., 2013](#_ENREF_356)) |
| p.? | c.1177-56G>A | Intron 7 | 24 | 0.0007649 | ([Bellanne-Chantelot, et al., 2010](#_ENREF_31)) |
| p.(Cys418Arg) | c.1252T>C | Exon 8 | 205 | 0.0007246 | ([Aguilar-Bryan and Bryan, 1999](#_ENREF_7); [Hoffman, et al., 2007](#_ENREF_154); [Otonkoski, et al., 2006](#_ENREF_284); [Riveline, et al., 2012](#_ENREF_317)) (¥) |
| p.? | c.1332+4del | Intron 8 | 318 | 0.001124 | ([Suchi, et al., 2006](#_ENREF_365)) (¥) |
| p.(Ile462Val) | c.1384A>G | Exon 9 | 257 | 0.000909 | ([Sandal, et al., 2009](#_ENREF_329); [Snider, et al., 2013](#_ENREF_356)) (¥) |
| p.? | c.1468-105A>G | Intron 9 | 13578 | 0.4357 | ([Snider, et al., 2013](#_ENREF_356)) |
| p.(Thr524=) | c.1572G>A | Exon 10 | 928 | 0.003284 | ([Snider, et al., 2013](#_ENREF_356)) |
| p.(Thr548=) | c.1644G>T | Exon 11 | 21 | 0.0001106 | Paris unpublished |
| p.? | c.1672-97A>C | Intron 11 | 3289 | 0.1048 | ([Snider, et al., 2013](#_ENREF_356)) |
| p.? | c.1672-74G>A | Intron 11 | 3903 | 0.1244 | ([Snider, et al., 2013](#_ENREF_356)) |
| p.(Val560Met) | c.1678G>A | Exon 12 | 178 | 0.0006312 | ([Ohta, et al., 1998](#_ENREF_276)) |
| p.(His562=) | c.1686C>T | Exon 12 | 119292 | 0.4231 | ([Fernandez-Marmiesse, et al., 2006](#_ENREF_106); [Hansen, et al., 1998](#_ENREF_145); [Nestorowicz, et al., 1996](#_ENREF_267); [Ohta, et al., 1998](#_ENREF_276); [Proks, et al., 2006a](#_ENREF_307); [Someya, et al., 2000](#_ENREF_358); [Tanizawa, et al., 2000](#_ENREF_378)) |
| p.(Ala569=) | c.1707C>T | Exon 12 | 335 | 0.001185 | ([Fernandez-Marmiesse, et al., 2006](#_ENREF_106)) |
| p.(Arg620Cys) | c.1858C>T | Exon 13 | 253 | 0.0008956 | ([Aguilar-Bryan and Bryan, 1999](#_ENREF_7); [Hardy, et al., 2007](#_ENREF_146); [Riveline, et al., 2012](#_ENREF_317); [Snider, et al., 2013](#_ENREF_356))(¥) |
| p.(Pro642=) | c.1926C>G | Exon 14 | 247 | 0.0008765 | ([Snider, et al., 2013](#_ENREF_356)) |
| p.(Lys649=) | c.1947G>A | Exon 14 | 48644 | 0.1724 | ([Fernandez-Marmiesse, et al., 2006](#_ENREF_106); [Hansen, et al., 1998](#_ENREF_145); [Ohta, et al., 1998](#_ENREF_276); [Someya, et al., 2000](#_ENREF_358); [Tanizawa, et al., 2000](#_ENREF_378)) |
| p.(Arg653Gln) | c.1958G>A | Exon 14 | 147 | 0.0005206 | ([Jahnavi, et al., 2013](#_ENREF_176))(¥) |
| p.? | c.2117-3C>T | Intron 15 | 120700 | 0.4337 | ([Fernandez-Marmiesse, et al., 2006](#_ENREF_106); [Hansen, et al., 1998](#_ENREF_145); [Nestorowicz, et al., 1996](#_ENREF_267); [Ohkubo, et al., 2005](#_ENREF_275); [Ohta, et al., 1998](#_ENREF_276); [Someya, et al., 2000](#_ENREF_358); [Thomas, et al., 1996b](#_ENREF_388)) |
| p.? | c.2259-50T>C | Intron 17 | 219038 | 0.8761 | ([Fernandez-Marmiesse, et al., 2006](#_ENREF_106)) |
| p.(Thr760=) | c.2280C>T | Exon 18 | 7485 | 0.02727 | ([Bellanne-Chantelot, et al., 2010](#_ENREF_31); [Fernandez-Marmiesse, et al., 2006](#_ENREF_106); [Gonen, et al., 2012](#_ENREF_135); [Hansen, et al., 1998](#_ENREF_145); [Inoue, et al., 1996](#_ENREF_167); [Nestorowicz, et al., 1996](#_ENREF_267)) |
| p.? | c.2295-36C>T | Intron 18 | 53106 | 0.1889 | ([Christesen, et al., 2007](#_ENREF_62); [Fernandez-Marmiesse, et al., 2006](#_ENREF_106); [Nestorowicz, et al., 1998](#_ENREF_265); [Tanizawa, et al., 2000](#_ENREF_378)) |
| p.? | c.2295-34T>C | Intron 18 | 58500 | 0.2079 | ([Christesen, et al., 2007](#_ENREF_62); [Fernandez-Marmiesse, et al., 2006](#_ENREF_106); [Nestorowicz, et al., 1998](#_ENREF_265); [Tanizawa, et al., 2000](#_ENREF_378)) |
| p.? | c.2393+123C>T | Intron 19 | 22485 | 0.718 | ([Snider, et al., 2013](#_ENREF_356)) |
| p.(Leu830=) | c.2488C>T | Exon 21 | 27787 | 0.1017 | ([Fernandez-Marmiesse, et al., 2006](#_ENREF_106); [Nestorowicz, et al., 1996](#_ENREF_267); [Ohta, et al., 1998](#_ENREF_276); [Tanizawa, et al., 2000](#_ENREF_378); [Thomas, et al., 1996b](#_ENREF_388)) |
| p.(His847=) | c.2541C>T | Exon 21 | 951 | 0.003416 | ([Snider, et al., 2013](#_ENREF_356)) |
| p.? | c.2559+22G>A | Intron 21 | 915 | 0.003427 | ([Snider, et al., 2013](#_ENREF_356)) |
| p.(Ala871=) | c.2613C>T | Exon 22 | 193 | 0.0006823 | ([Snider, et al., 2013](#_ENREF_356)) |
| p.? | c.2823+17A>G | Intron 23 | 173078 | 0.6170 | ([Snider, et al., 2013](#_ENREF_356)) |
| p.? | c.2823+67C>T | Intron 23 | 4242 | 0.1354 | ([Bellanne-Chantelot, et al., 2010](#_ENREF_31)) |
| p.(Ser987=) | c.2961G>A | Exon 25 | 326 | 0.001171 | ([Snider, et al., 2013](#_ENREF_356)) |
| p.? | c.3332+6C>T | Intron 26 | 3259 | 0.01162 | ([Hansen, et al., 1998](#_ENREF_145); [Nestorowicz, et al., 1996](#_ENREF_267)) |
| p.? | c.3402+13G>A | Intron 27 | 258 | 0.000912 | ([Nestorowicz, et al., 1998](#_ENREF_265); [Snider, et al., 2013](#_ENREF_356)) |
| p.? | c.3402+45C>A | Intron 27 | 3111 | 0.011 | ([Nestorowicz, et al., 1998](#_ENREF_265)) |
| p.? | c.3560+118_3560+121del | Intron 28 | 2265 | 0.07234 | ([Snider, et al., 2013](#_ENREF_356)) |
| p.? | c.3561-95C>T | Intron 28 | 3486 | 0.1111 | ([Snider, et al., 2013](#_ENREF_356)) |
| p.(Ala1205=) | c.3615C>T | Exon 29 | 5257 | 0.01858 | ([Nestorowicz, et al., 1998](#_ENREF_265); [Ohta, et al., 1998](#_ENREF_276)) |
| p.(Arg1274=) | c.3822G>A | Exon 31 | 84311 | 0.2995 | ([Fernandez-Marmiesse, et al., 2006](#_ENREF_106); [Goksel, et al., 1998](#_ENREF_133); [Gonen, et al., 2012](#_ENREF_135); [Hansen, et al., 1998](#_ENREF_145); [Nestorowicz, et al., 1996](#_ENREF_267); [Nikolac, et al., 2012](#_ENREF_271); [Ohkubo, et al., 2005](#_ENREF_275); [Ohta, et al., 1998](#_ENREF_276); [Proks, et al., 2006a](#_ENREF_307); [Tanizawa, et al., 2000](#_ENREF_378)) |
| p.(Ala1370Ser) | c.4108G>T | Exon 33 | 181656 | 0.6434 | ([Fernandez-Marmiesse, et al., 2006](#_ENREF_106); [Gonen, et al., 2012](#_ENREF_135); [Hansen, et al., 1998](#_ENREF_145); [Inoue, et al., 1996](#_ENREF_167); [Nestorowicz, et al., 1996](#_ENREF_267); [Ohkubo, et al., 2005](#_ENREF_275); [Ohta, et al., 1998](#_ENREF_276); [Ortiz, et al., 2012](#_ENREF_282); [Someya, et al., 2000](#_ENREF_358); [Tanizawa, et al., 2000](#_ENREF_378)) |
| p.(Gln1373=) | c.4119G>A | Exon 33 | 45 | 0.0001592 | Chicago unpublished |
| p.? | c.4122+18A>G | Intron 33 | 896 | 0.003178 | ([Bellanne-Chantelot, et al., 2010](#_ENREF_31)) |
| p.? | c.4122+93G>T | Intron 33 | 6218 | 0.1986 | ([Snider, et al., 2013](#_ENREF_356)) |
| p.? | c.4123-27T>C | Intron 33 | 71768 | 0.3689 | ([Fernandez-Marmiesse, et al., 2006](#_ENREF_106); [Goksel, et al., 1998](#_ENREF_133)) |
| p.? | c.4123-19C>T | Intron 33 | 3301 | 0.01674 | ([Bellanne-Chantelot, et al., 2010](#_ENREF_31); [Fernandez-Marmiesse, et al., 2006](#_ENREF_106)) |
| p.? | c.4201+62G>A | Intron 34 | 1645 | 0.05242 | ([Goksel, et al., 1998](#_ENREF_133)) |
| p.? | c.4415-179C>T | Intron 36 | 1595 | 0.05084 | ([Snider, et al., 2013](#_ENREF_356)) |
| p.? | c.4415-151C>T | Intron 36 | 554 | 0.01766 | ([Snider, et al., 2013](#_ENREF_356)) |
| p.(Ala1515=) | c.4545C>A | Exon 37 | 413 | 0.001462 | ([Bellanne-Chantelot, et al., 2010](#_ENREF_31)) |
| p.? | c.4548+13C>T | Intron 37 | 195 | 0.0006911 | ([Snider, et al., 2013](#_ENREF_356)) |
| p.? | c.4611+54G>C | Intron 38 | 22087 | 0.7046 | ([Snider, et al., 2013](#_ENREF_356)) |
| p.? | c.4612-40A>G | Intron 38 | 93820 | 0.341 | ([Fernandez-Marmiesse, et al., 2006](#_ENREF_106); [Goksel, et al., 1998](#_ENREF_133)) |
| p.? | c.4612-82G>A | Intron 38 | 7006 | 0.2258 | ([Goksel, et al., 1998](#_ENREF_133)) |
| p.(Lys1553=) | c.4659G>A | Exon 39 | 118 | 0.0004174 | Chicago unpublished |
| p.(Arg1569Gln) | c.4706G>A | Exon 39 | 194 | 0.0006861 | ([Snider, et al., 2013](#_ENREF_356)) |
| p.(Val1573Ile) | c.4717G>A | Exon 39 | 15302 | 0.05412 | ([Fernandez-Marmiesse, et al., 2006](#_ENREF_106); [Giurgea, et al., 2004](#_ENREF_125); [Nestorowicz, et al., 1996](#_ENREF_267)) |
| p.(Phe1577=) | c.4731C>T | Exon 39 | 1104 | 0.003904 | ([Snider, et al., 2013](#_ENREF_356)) |
| p.? | c.*525C>T | 3' UTR | 14195 | 0.453 | ([Snider, et al., 2013](#_ENREF_356)) |
| p.? | c.*593C>T | 3' UTR | 14191 | 0.4531 | ([Snider, et al., 2013](#_ENREF_356)) |
| p.? | c.*970A>G | 3' UTR | 8383 | 0.268 | ([Snider, et al., 2013](#_ENREF_356)) |
| p.? | c.*1578T>C | 3' UTR | 22941 | 0.7326 | ([Snider, et al., 2013](#_ENREF_356)) |
| p.? | c.*1769G>T | 3' UTR | 10695 | 0.3431 | ([Snider, et al., 2013](#_ENREF_356)) |
| p.? | c.*1773G>T | 3' UTR | 10689 | 0.3429 | ([Snider, et al., 2013](#_ENREF_356)) |
| p.? | c.*1774C>T | 3' UTR | 10689 | 0.3432 | ([Snider, et al., 2013](#_ENREF_356)) |
| p.? | c.*1821C>A | 3' UTR | 8480 | 0.2706 | ([Snider, et al., 2013](#_ENREF_356)) |
| p.? | c.*2363T>C | 3' UTR | 8414 | 0.2724 | ([Snider, et al., 2013](#_ENREF_356)) |

**REFERENCES**

Abbasi F, Saba S, Ebrahim-Habibi A, Sayahpour FA, Amiri P, Larijani B, Amoli MM. 2012. Detection of KCNJ11 gene mutations in a family with neonatal diabetes mellitus: implications for therapeutic management of family members with long-standing disease. Mol Diagn Ther 16(2):109-14.

Abdulhadi-Atwan M, Bushman J, Tornovsky-Babaey S, Perry A, Abu-Libdeh A, Glaser B, Shyng SL, Zangen DH. 2008. Novel de novo mutation in sulfonylurea receptor 1 presenting as hyperinsulinism in infancy followed by overt diabetes in early adolescence. Diabetes 57(7):1935-40.

Abraham MB, Shetty VB, Price G, Smith N, Bock M, Siafarikas A, Resnick S, Whan E, Ellard S, Flanagan SE and others. 2015. Efficacy and safety of sirolimus in a neonate with persistent hypoglycaemia following near-total pancreatectomy for hyperinsulinaemic hypoglycaemia. J Pediatr Endocrinol Metab 28(11-12):1391-8.

Abraham N, Ahamed A, Unnikrishnan AG, Kumar H, Ellard S. 2014. Permanent neonatal diabetes mellitus due to an ABCC8 mutation: a case report. JOP 15(2):198-200.

Abujbara MA, Liswi MI, El-Khateeb MS, Flanagan SE, Ellard S, Ajlouni KM. 2014. Permanent neonatal diabetes mellitus in Jordan. J Pediatr Endocrinol Metab 27(9-10):879-83.

Adi A, Abbas BB, Hamed MA, Tassan NA, Bakheet D. 2015. Screening for Mutations in ABCC8 and KCNJ11 Genes in Saudi Persistent Hyperinsulinemic Hypoglycemia of Infancy (PHHI) Patients. Genes (Basel) 6(2):206-15.

Aguilar-Bryan L, Bryan J. 1999. Molecular biology of adenosine triphosphate-sensitive potassium channels. Endocr Rev 20(2):101-35.

Ahn SY, Kim GH, Yoo HW. 2015. Successful sulfonylurea treatment in a patient with permanent neonatal diabetes mellitus with a novel KCNJ11 mutation. Korean J Pediatr 58(8):309-12.

Al-Agha AE, Ahmad IA. 2013. Characterization of the ABCC8 gene mutation and phenotype in patients with congenital hyperinsulinism in western Saudi Arabia. Saudi Med J 34(10):1002-6.

Al-Mahdi M, Al Mutair A, Al Balwi M, Hussain K. 2010. Successful transfer from insulin to oral sulfonylurea in a 3-year-old girl with a mutation in the KCNJ11 gene. Ann Saudi Med 30(2):162-4.

Al Senani A, Hamza N, Al Azkawi H, Al Kharusi M, Al Sukaiti N, Al Badi M, Al Yahyai M, Johnson M, De Franco E, Flanagan S and others. 2018. Genetic mutations associated with neonatal diabetes mellitus in Omani patients. J Pediatr Endocrinol Metab 31(2):195-204.

Albaqumi M, Alhabib FA, Shamseldin HE, Mohammed F, Alkuraya FS. 2014. A syndrome of congenital hyperinsulinism and rhabdomyolysis is caused by KCNJ11 mutation. J Med Genet 51(4):271-4.

Alkorta-Aranburu G, Carmody D, Cheng YW, Nelakuditi V, Ma L, Dickens JT, Das S, Greeley SAW, Del Gaudio D. 2014. Phenotypic heterogeneity in monogenic diabetes: the clinical and diagnostic utility of a gene panel-based next-generation sequencing approach. Mol Genet Metab 113(4):315-320.

Ang SF, Lim SC, Tan C, Fong JC, Kon WY, Lian JX, Subramanium T, Sum CF. 2016. A preliminary study to evaluate the strategy of combining clinical criteria and next generation sequencing (NGS) for the identification of monogenic diabetes among multi-ethnic Asians. Diabetes Res Clin Pract 119:13-22.

Anik A, Catli G, Abaci A, Yis U, Oren H, Guleryuz H, Kizildag S, Bober E. 2014. A novel activating ABCC8 mutation underlying neonatal diabetes mellitus in an infant presenting with cerebral sinovenous thrombosis. J Pediatr Endocrinol Metab 27(5-6):533-7.

Apperley L, Giri D, Houghton JAL, Flanagan SE, Didi M, Senniappan S. 2019. A rare case of congenital hyperinsulinism (CHI) due to dual genetic aetiology involving HNF4A and ABCC8. J Pediatr Endocrinol Metab 32(3):301-304.

Arbizu Lostao J, Fernandez-Marmiesse A, Garrastachu Zumarran P, Martino Casado E, Azcona San Julian C, Carracedo A, Richter Echevarria JA. 2008. [18F-fluoro-L-DOPA PET-CT imaging combined with genetic analysis for optimal classification and treatment in a child with severe congenital hyperinsulinism]. An Pediatr (Barc) 68(5):481-5.

Artuso R, Provenzano A, Mazzinghi B, Giunti L, Palazzo V, Andreucci E, Blasetti A, Chiuri RM, Gianiorio FE, Mandich P and others. 2015. Therapeutic implications of novel mutations of the RFX6 gene associated with early-onset diabetes. Pharmacogenomics J 15(1):49-54.

Arya VB, Aziz Q, Nessa A, Tinker A, Hussain K. 2014a. Congenital hyperinsulinism: clinical and molecular characterisation of compound heterozygous ABCC8 mutation responsive to Diazoxide therapy. Int J Pediatr Endocrinol 2014(1):24.

Arya VB, Guemes M, Nessa A, Alam S, Shah P, Gilbert C, Senniappan S, Flanagan SE, Ellard S, Hussain K. 2014b. Clinical and histological heterogeneity of congenital hyperinsulinism due to paternally inherited heterozygous ABCC8/KCNJ11 mutations. Eur J Endocrinol 171(6):685-95.

Arya VB, Mohammed Z, Blankenstein O, De Lonlay P, Hussain K. 2014c. Hyperinsulinaemic hypoglycaemia. Horm Metab Res 46(3):157-70.

Aydin BK, Bundak R, Bas F, Maras H, Saka N, Gunoz H, Darendeliler F. 2012. Permanent neonatal diabetes mellitus: same mutation, different glycemic control with sulfonylurea therapy on long-term follow-up. J Clin Res Pediatr Endocrinol 4(2):107-10.

Babenko AP. 2008. A novel ABCC8 (SUR1)-dependent mechanism of metabolism-excitation uncoupling. J Biol Chem 283(14):8778-82.

Babenko AP, Polak M, Cave H, Busiah K, Czernichow P, Scharfmann R, Bryan J, Aguilar-Bryan L, Vaxillaire M, Froguel P. 2006. Activating mutations in the ABCC8 gene in neonatal diabetes mellitus. N Engl J Med 355(5):456-66.

Babiker T, Vedovato N, Patel K, Thomas N, Finn R, Mannikko R, Chakera AJ, Flanagan SE, Shepherd MH, Ellard S and others. 2016. Successful transfer to sulfonylureas in KCNJ11 neonatal diabetes is determined by the mutation and duration of diabetes. Diabetologia 59(6):1162-6.

Baier LJ, Muller YL, Remedi MS, Traurig M, Piaggi P, Wiessner G, Huang K, Stacy A, Kobes S, Krakoff J and others. 2015. ABCC8 R1420H Loss-of-Function Variant in a Southwest American Indian Community: Association With Increased Birth Weight and Doubled Risk of Type 2 Diabetes. Diabetes 64(12):4322-32.

Banerjee I, Skae M, Flanagan SE, Rigby L, Patel L, Didi M, Blair J, Ehtisham S, Ellard S, Cosgrove KE and others. 2011. The contribution of rapid KATP channel gene mutation analysis to the clinical management of children with congenital hyperinsulinism. Eur J Endocrinol 164(5):733-40.

Bas VN, Ozkan M, Zenciroglu A, Cavusoglu YH, Cetinkaya S, Aycan Z. 2012. Seizure due to somatostatin analog discontinuation in a case diagnosed as congenital hyperinsulinism novel mutation. J Pediatr Endocrinol Metab 25(5-6):553-5.

Batra CM, Gupta N, Atwal G, Gupta V. 2009. Transient neonatal diabetes due to activating mutation in the ABCC8 gene encoding SUR1. Indian J Pediatr 76(11):1169-72.

Battaglia D, Lin YW, Brogna C, Crino A, Grasso V, Mozzi AF, Russo L, Spera S, Colombo C, Ricci S and others. 2012. Glyburide ameliorates motor coordination and glucose homeostasis in a child with diabetes associated with the KCNJ11/S225T, del226-232 mutation. Pediatr Diabetes 13(8):656-60.

Bellanne-Chantelot C, Saint-Martin C, Ribeiro MJ, Vaury C, Verkarre V, Arnoux JB, Valayannopoulos V, Gobrecht S, Sempoux C, Rahier J and others. 2010. ABCC8 and KCNJ11 molecular spectrum of 109 patients with diazoxide-unresponsive congenital hyperinsulinism. J Med Genet 47(11):752-9.

Bendix J, Laursen MG, Mortensen MB, Melikian M, Globa E, Detlefsen S, Rasmussen L, Petersen H, Brusgaard K, Christesen HT. 2018. Intraoperative Ultrasound: A Tool to Support Tissue-Sparing Curative Pancreatic Resection in Focal Congenital Hyperinsulinism. Front Endocrinol (Lausanne) 9:478.

Bennett JT, Vasta V, Zhang M, Narayanan J, Gerrits P, Hahn SH. 2015. Molecular genetic testing of patients with monogenic diabetes and hyperinsulinism. Mol Genet Metab 114(3):451-8.

Bennett K, James C, Hussain K. 2010. Pancreatic beta-cell KATP channels: Hypoglycaemia and hyperglycaemia. Rev Endocr Metab Disord 11(3):157-63.

Biagiotti L, Proverbio MC, Bosio L, Gervasi F, Rovida E, Cerioni V, Bove M, Valin PS, Albarello L, Zamproni I and others. 2007. Identification of two novel frameshift mutations in the KCNJ11 gene in two Italian patients affected by Congenital Hyperinsulinism of Infancy. Exp Mol Pathol 83(1):59-64.

Bitner-Glindzicz M, Lindley KJ, Rutland P, Blaydon D, Smith VV, Milla PJ, Hussain K, Furth-Lavi J, Cosgrove KE, Shepherd RM and others. 2000. A recessive contiguous gene deletion causing infantile hyperinsulinism, enteropathy and deafness identifies the Usher type 1C gene. Nat Genet 26(1):56-60.

Bonnefond A, Durand E, Sand O, De Graeve F, Gallina S, Busiah K, Lobbens S, Simon A, Bellanne-Chantelot C, Letourneau L and others. 2010. Molecular diagnosis of neonatal diabetes mellitus using next-generation sequencing of the whole exome. PLoS One 5(10):e13630.

Bonnefond A, Philippe J, Durand E, Dechaume A, Huyvaert M, Montagne L, Marre M, Balkau B, Fajardy I, Vambergue A and others. 2012. Whole-exome sequencing and high throughput genotyping identified KCNJ11 as the thirteenth MODY gene. PLoS One 7(6):e37423.

Bonnefond A, Philippe J, Durand E, Muller J, Saeed S, Arslan M, Martinez R, De Graeve F, Dhennin V, Rabearivelo I and others. 2014. Highly sensitive diagnosis of 43 monogenic forms of diabetes or obesity through one-step PCR-based enrichment in combination with next-generation sequencing. Diabetes Care 37(2):460-7.

Boodhansingh KE, Kandasamy B, Mitteer L, Givler S, De Leon DD, Shyng SL, Ganguly A, Stanley CA. 2019. Novel dominant KATP channel mutations in infants with congenital hyperinsulinism: Validation by in vitro expression studies and in vivo carrier phenotyping. Am J Med Genet A.

Bourron O, Chebbi F, Halbron M, Saint-Martin C, Bellanne-Chantelot C, Abed A, Charbit B, Magnan C, Lacorte JM, Hartemann A. 2012. Incretin effect of glucagon-like peptide 1 receptor agonist is preserved in presence of ABCC8/SUR1 mutation in beta-cell. Diabetes Care 35(11):e76.

Bowman P, Flanagan SE, Edghill EL, Damhuis A, Shepherd MH, Paisey R, Hattersley AT, Ellard S. 2012. Heterozygous ABCC8 mutations are a cause of MODY. Diabetologia 55(1):123-7.

Brady C, Palladino AA, Gutmark-Little I. 2015. A novel case of compound heterozygous congenital hyperinsulinism without high insulin levels. Int J Pediatr Endocrinol 2015(1):16.

Bremer AA, Ranadive S, Lustig RH. 2008. Outpatient transition of an infant with permanent neonatal diabetes due to a KCNJ11 activating mutation from subcutaneous insulin to oral glyburide. Pediatr Diabetes 9(3 Pt 1):236-9.

Brereton MF, Iberl M, Shimomura K, Zhang Q, Adriaenssens AE, Proks P, Spiliotis, II, Dace W, Mattis KK, Ramracheya R and others. 2014. Reversible changes in pancreatic islet structure and function produced by elevated blood glucose. Nat Commun 5:4639.

Bruederle CE, Gay J, Shyng SL. 2011. A role of the sulfonylurea receptor 1 in endocytic trafficking of ATP-sensitive potassium channels. Traffic 12(9):1242-56.

Brunetti-Pierri N, Olutoye OO, Heptulla R, Tatevian N. 2008. Case report: pathological features of aberrant pancreatic development in congenital hyperinsulinism due to ABCC8 mutations. Ann Clin Lab Sci 38(4):386-9.

Bushman JD, Gay JW, Tewson P, Stanley CA, Shyng SL. 2010. Characterization and functional restoration of a potassium channel Kir6.2 pore mutation identified in congenital hyperinsulinism. J Biol Chem 285(9):6012-23.

Busiah K, Verkarre V, Cave H, Scharfmann R, Polak M. 2014. Human pancreas endocrine cell populations and activating ABCC8 mutations. Horm Res Paediatr 82(1):59-64.

Cai M, Bompada P, Atac D, Laakso M, Groop L, De Marinis Y. 2016. Epigenetic regulation of glucose-stimulated osteopontin (OPN) expression in diabetic kidney. Biochem Biophys Res Commun 469(1):108-113.

Calabria AC, Li C, Gallagher PR, Stanley CA, De Leon DD. 2012. GLP-1 receptor antagonist exendin-(9-39) elevates fasting blood glucose levels in congenital hyperinsulinism owing to inactivating mutations in the ATP-sensitive K+ channel. Diabetes 61(10):2585-91.

Calton EA, Temple IK, Mackay DJ, Lever M, Ellard S, Flanagan SE, Davies JH, Hussain K, Gray JC. 2013. Hepatoblastoma in a child with a paternally-inherited ABCC8 mutation and mosaic paternal uniparental disomy 11p causing focal congenital hyperinsulinism. Eur J Med Genet 56(2):114-7.

Cao B, Gong C, Wu D, Lu C, Liu F, Liu X, Zhang Y, Gu Y, Qi Z, Li X and others. 2016. Genetic Analysis and Follow-Up of 25 Neonatal Diabetes Mellitus Patients in China. J Diabetes Res 2016:6314368.

Carmody D, Bell CD, Hwang JL, Dickens JT, Sima DI, Felipe DL, Zimmer CA, Davis AO, Kotlyarevska K, Naylor RN and others. 2014. Sulfonylurea treatment before genetic testing in neonatal diabetes: pros and cons. J Clin Endocrinol Metab 99(12):E2709-14.

Cartier EA, Conti LR, Vandenberg CA, Shyng SL. 2001. Defective trafficking and function of KATP channels caused by a sulfonylurea receptor 1 mutation associated with persistent hyperinsulinemic hypoglycemia of infancy. Proc Natl Acad Sci U S A 98(5):2882-7.

Celik N, Cinaz P, Emeksiz HC, Hussain K, Camurdan O, Bideci A, Doger E, Yuce O, Turkyilmaz Z, Oguz AD. 2013. Octreotide-induced long QT syndrome in a child with congenital hyperinsulinemia and a novel missense mutation (p.Met115Val) in the ABCC8 gene. Horm Res Paediatr 80(4):299-303.

Chai-Udom R, Sahakitrungruang T, Wacharasindhu S, Supornsilchai V. 2016. A girl with permanent neonatal diabetes due to KCNJ11 mutation presented with Mauriac syndrome after improper adjustment in sulfonylurea dosage over 6 years. J Pediatr Endocrinol Metab 29(9):1095-101.

Chan KW, Zhang H, Logothetis DE. 2003. N-terminal transmembrane domain of the SUR controls trafficking and gating of Kir6 channel subunits. EMBO J 22(15):3833-43.

Chan YM, Laffel LM. 2007. Transition from insulin to glyburide in a 4-month-old girl with neonatal diabetes mellitus caused by a mutation in KCNJ11. Pediatr Diabetes 8(4):235-8.

Chang WL, Huang CJ, Lei TH, Niu DM, Chiu CY, Jap TS. 2014. A novel mutation of KCNJ11 gene in a patient with permanent neonatal diabetes mellitus. Diabetes Res Clin Pract 104(1):e29-32.

Chen PC, Olson EM, Zhou Q, Kryukova Y, Sampson HM, Thomas DY, Shyng SL. 2013. Carbamazepine as a novel small molecule corrector of trafficking-impaired ATP-sensitive potassium channels identified in congenital hyperinsulinism. J Biol Chem 288(29):20942-54.

Christesen HB, Brusgaard K, Alm J, Sjoblad S, Hussain K, Fenger C, Rasmussen L, Hovendal C, Otonkoski T, Jacobsen BB. 2007. Rapid genetic analysis in congenital hyperinsulinism. Horm Res 67(4):184-8.

Christesen HB, Feilberg-Jorgensen N, Jacobsen BB. 2001. Pancreatic beta-cell stimulation tests in transient and persistent congenital hyperinsulinism. Acta Paediatr 90(10):1116-20.

Christesen HT, Brusgaard K, Hussain K. 2012. Recurrent spontaneous hypoglycaemia causes loss of neurogenic and neuroglycopaenic signs in infants with congenital hyperinsulinism. Clin Endocrinol (Oxf) 76(4):548-54.

Clark R, Mannikko R, Stuckey DJ, Iberl M, Clarke K, Ashcroft FM. 2012. Mice expressing a human K(ATP) channel mutation have altered channel ATP sensitivity but no cardiac abnormalities. Diabetologia 55(4):1195-204.

Clark RH, McTaggart JS, Webster R, Mannikko R, Iberl M, Sim XL, Rorsman P, Glitsch M, Beeson D, Ashcroft FM. 2010. Muscle dysfunction caused by a KATP channel mutation in neonatal diabetes is neuronal in origin. Science 329(5990):458-61.

Codner E, Flanagan S, Ellard S, Garcia H, Hattersley AT. 2005. High-dose glibenclamide can replace insulin therapy despite transitory diarrhea in early-onset diabetes caused by a novel R201L Kir6.2 mutation. Diabetes Care 28(3):758-9.

Codner E, Flanagan SE, Ugarte F, Garcia H, Vidal T, Ellard S, Hattersley AT. 2007. Sulfonylurea treatment in young children with neonatal diabetes: dealing with hyperglycemia, hypoglycemia, and sick days. Diabetes Care 30(5):e28-9.

Colombo C, Delvecchio M, Zecchino C, Faienza MF, Cavallo L, Barbetti F. 2005. Transient neonatal diabetes mellitus is associated with a recurrent (R201H) KCNJ11 (KIR6.2) mutation. Diabetologia 48(11):2439-41.

Coventry A, Bull-Otterson LM, Liu X, Clark AG, Maxwell TJ, Crosby J, Hixson JE, Rea TJ, Muzny DM, Lewis LR and others. 2010. Deep resequencing reveals excess rare recent variants consistent with explosive population growth. Nat Commun 1:131.

Craig TJ, Shimomura K, Holl RW, Flanagan SE, Ellard S, Ashcroft FM. 2009. An in-frame deletion in Kir6.2 (KCNJ11) causing neonatal diabetes reveals a site of interaction between Kir6.2 and SUR1. J Clin Endocrinol Metab 94(7):2551-7.

Craigie RJ, Salomon-Estebanez M, Yau D, Han B, Mal W, Newbould M, Cheesman E, Bitetti S, Mohamed Z, Sajjan R and others. 2018. Clinical Diversity in Focal Congenital Hyperinsulinism in Infancy Correlates With Histological Heterogeneity of Islet Cell Lesions. Front Endocrinol (Lausanne) 9:619.

D'Amato E, Tammaro P, Craig TJ, Tosi A, Giorgetti R, Lorini R, Ashcroft FM. 2008. Variable phenotypic spectrum of diabetes mellitus in a family carrying a novel KCNJ11 gene mutation. Diabet Med 25(6):651-6.

Dalvi NN, Shaikh ST, Shivane VK, Lila AR, Bandgar TR, Shah NS. 2017. Genetically Confirmed Neonatal Diabetes: A Single Centre Experience. Indian J Pediatr 84(1):86-88.

Damaj L, le Lorch M, Verkarre V, Werl C, Hubert L, Nihoul-Fekete C, Aigrain Y, de Keyzer Y, Romana SP, Bellanne-Chantelot C and others. 2008. Chromosome 11p15 paternal isodisomy in focal forms of neonatal hyperinsulinism. J Clin Endocrinol Metab 93(12):4941-7.

Darendeliler F, Fournet JC, Bas F, Junien C, Gross MS, Bundak R, Saka N, Gunoz H. 2002. ABCC8 (SUR1) and KCNJ11 (KIR6.2) mutations in persistent hyperinsulinemic hypoglycemia of infancy and evaluation of different therapeutic measures. J Pediatr Endocrinol Metab 15(7):993-1000.

Dastamani A, Guemes M, Pitfield C, Morgan K, Rajab M, Rottenburger C, Bomanji J, De Coppi P, Dattani M, Shah P. 2019. The Use of a Long-Acting Somatostatin Analogue (Lanreotide) in Three Children with Focal Forms of Congenital Hyperinsulinaemic Hypoglycaemia. Horm Res Paediatr 91(1):56-61.

Daublin G, Lorenz-Depiereux B, Strom TM, Blankenstein O, Raile K. 2007. Early glibenclamide treatment in a clinical newborn with KCNJ11 gene mutation. Diabetes Care 30(10):e104.

de Lonlay-Debeney P, Poggi-Travert F, Fournet JC, Sempoux C, Dionisi Vici C, Brunelle F, Touati G, Rahier J, Junien C, Nihoul-Fekete C and others. 1999. Clinical features of 52 neonates with hyperinsulinism. N Engl J Med 340(15):1169-75.

De Marinis Y, Cai M, Bompada P, Atac D, Kotova O, Johansson ME, Garcia-Vaz E, Gomez MF, Laakso M, Groop L. 2016. Epigenetic regulation of the thioredoxin-interacting protein (TXNIP) gene by hyperglycemia in kidney. Kidney Int 89(2):342-53.

De Vroede M, Bax NM, Brusgaard K, Dunne MJ, Groenendaal F. 2004. Laparoscopic diagnosis and cure of hyperinsulinism in two cases of focal adenomatous hyperplasia in infancy. Pediatrics 114(4):e520-2.

de Wet H, Proks P. 2015. Molecular action of sulphonylureas on KATP channels: a real partnership between drugs and nucleotides. Biochem Soc Trans 43(5):901-7.

de Wet H, Proks P, Lafond M, Aittoniemi J, Sansom MS, Flanagan SE, Pearson ER, Hattersley AT, Ashcroft FM. 2008. A mutation (R826W) in nucleotide-binding domain 1 of ABCC8 reduces ATPase activity and causes transient neonatal diabetes. EMBO Rep 9(7):648-54.

de Wet H, Rees MG, Shimomura K, Aittoniemi J, Patch AM, Flanagan SE, Ellard S, Hattersley AT, Sansom MS, Ashcroft FM. 2007. Increased ATPase activity produced by mutations at arginine-1380 in nucleotide-binding domain 2 of ABCC8 causes neonatal diabetes. Proc Natl Acad Sci U S A 104(48):18988-92.

Deeb A, Habeb A, Kaplan W, Attia S, Hadi S, Osman A, Al-Jubeh J, Flanagan S, DeFranco E, Ellard S. 2016. Genetic characteristics, clinical spectrum, and incidence of neonatal diabetes in the Emirate of AbuDhabi, United Arab Emirates. Am J Med Genet A 170(3):602-9.

Del Roio Liberatore R, Jr., Ramos PM, Guerra G, Jr., Manna TD, Silva IN, Martinelli CE, Jr. 2015. Clinical and molecular data from 61 Brazilian cases of Congenital Hyperinsulinemic Hypoglycemia. Diabetol Metab Syndr 7:5.

Della Manna T, Battistim C, Radonsky V, Savoldelli RD, Damiani D, Kok F, Pearson ER, Ellard S, Hattersley AT, Reis AF. 2008. Glibenclamide unresponsiveness in a Brazilian child with permanent neonatal diabetes mellitus and DEND syndrome due to a C166Y mutation in KCNJ11 (Kir6.2) gene. Arq Bras Endocrinol Metabol 52(8):1350-5.

Demirbilek H, Arya VB, Ozbek MN, Akinci A, Dogan M, Demirel F, Houghton J, Kaba S, Guzel F, Baran RT and others. 2014. Clinical characteristics and phenotype-genotype analysis in Turkish patients with congenital hyperinsulinism; predominance of recessive KATP channel mutations. Eur J Endocrinol 170(6):885-92.

Demirbilek H, Arya VB, Ozbek MN, Houghton JA, Baran RT, Akar M, Tekes S, Tuzun H, Mackay DJ, Flanagan SE and others. 2015. Clinical characteristics and molecular genetic analysis of 22 patients with neonatal diabetes from the South-Eastern region of Turkey: predominance of non-KATP channel mutations. Eur J Endocrinol 172(6):697-705.

Doneray H, Houghton J, Tekgunduz KS, Balkir F, Caner I. 2014. Permanent neonatal diabetes mellitus caused by a novel mutation in the KCNJ11 gene. J Pediatr Endocrinol Metab 27(3-4):367-71.

Drain P, Li L, Wang J. 1998. KATP channel inhibition by ATP requires distinct functional domains of the cytoplasmic C terminus of the pore-forming subunit. Proc Natl Acad Sci U S A 95(23):13953-8.

Du Y, Ju R, Xi Y, Gou P. 2019. A Newborn with Congenital Hyperinsulinism. Fetal Pediatr Pathol:1-6.

Dunne MJ, Kane C, Shepherd RM, Sanchez JA, James RF, Johnson PR, Aynsley-Green A, Lu S, Clement JPt, Lindley KJ and others. 1997. Familial persistent hyperinsulinemic hypoglycemia of infancy and mutations in the sulfonylurea receptor. N Engl J Med 336(10):703-6.

Dupont J, Pereira C, Medeira A, Duarte R, Ellard S, Sampaio L. 2012. Permanent neonatal diabetes mellitus due to KCNJ11 mutation in a Portuguese family: transition from insulin to oral sulfonylureas. J Pediatr Endocrinol Metab 25(3-4):367-70.

Durmaz E, Flanagan SE, Parlak M, Ellard S, Akcurin S, Bircan I. 2014. A combination of nifedipine and octreotide treatment in an hyperinsulinemic hypoglycemic infant. J Clin Res Pediatr Endocrinol 6(2):119-21.

Edghill EL, Gloyn AL, Gillespie KM, Lambert AP, Raymond NT, Swift PG, Ellard S, Gale EA, Hattersley AT. 2004. Activating mutations in the KCNJ11 gene encoding the ATP-sensitive K+ channel subunit Kir6.2 are rare in clinically defined type 1 diabetes diagnosed before 2 years. Diabetes 53(11):2998-3001.

Edghill EL, Gloyn AL, Goriely A, Harries LW, Flanagan SE, Rankin J, Hattersley AT, Ellard S. 2007. Origin of de novo KCNJ11 mutations and risk of neonatal diabetes for subsequent siblings. J Clin Endocrinol Metab 92(5):1773-7.

Ellard S, Flanagan SE, Girard CA, Patch AM, Harries LW, Parrish A, Edghill EL, Mackay DJ, Proks P, Shimomura K and others. 2007. Permanent neonatal diabetes caused by dominant, recessive, or compound heterozygous SUR1 mutations with opposite functional effects. Am J Hum Genet 81(2):375-82.

Ellard S, Lango Allen H, De Franco E, Flanagan SE, Hysenaj G, Colclough K, Houghton JA, Shepherd M, Hattersley AT, Weedon MN and others. 2013. Improved genetic testing for monogenic diabetes using targeted next-generation sequencing. Diabetologia 56(9):1958-63.

Esmatjes E, Jimenez A, Diaz G, Mora M, Casamitjana R, Perez de Nanclares G, Castano L, Jose Ricart M. 2008. Neonatal diabetes with end-stage nephropathy: pancreas transplantation decision. Diabetes Care 31(11):2116-7.

Faletra F, Athanasakis E, Morgan A, Biarnes X, Fornasier F, Parini R, Furlan F, Boiani A, Maiorana A, Dionisi-Vici C and others. 2013a. Congenital hyperinsulinism: clinical and molecular analysis of a large Italian cohort. Gene 521(1):160-5.

Faletra F, Snider K, Shyng SL, Bruno I, Athanasakis E, Gasparini P, Dionisi-Vici C, Ventura A, Zhou Q, Stanley CA and others. 2013b. Co-inheritance of two ABCC8 mutations causing an unresponsive congenital hyperinsulinism: clinical and functional characterization of two novel ABCC8 mutations. Gene 516(1):122-5.

Fan ZC, Ni JW, Yang L, Hu LY, Ma SM, Mei M, Sun BJ, Wang HJ, Zhou WH. 2015. Uncovering the molecular pathogenesis of congenital hyperinsulinism by panel gene sequencing in 32 Chinese patients. Mol Genet Genomic Med 3(6):526-36.

Fanciullo L, Iovane B, Gkliati D, Monti G, Sponzilli I, Cangelosi AM, Matrorilli C, Chiari G, Barbetti F, Dall'Aglio E and others. 2012. Sulfonylurea-responsive neonatal diabetes mellitus diagnosed through molecular genetics in two children and in one adult after a long period of insulin treatment. Acta Biomed 83(1):56-61.

Feigerlova E, Pruhova S, Dittertova L, Lebl J, Pinterova D, Kolostova K, Cerna M, Pedersen O, Hansen T. 2006. Aetiological heterogeneity of asymptomatic hyperglycaemia in children and adolescents. Eur J Pediatr 165(7):446-52.

Fernandez-Marmiesse A, Salas A, Vega A, Fernandez-Lorenzo JR, Barreiro J, Carracedo A. 2006. Mutation spectra of ABCC8 gene in Spanish patients with Hyperinsulinism of Infancy (HI). Hum Mutat 27(2):214.

Flanagan S, Damhuis A, Banerjee I, Rokicki D, Jefferies C, Kapoor R, Hussain K, Ellard S. 2012. Partial ABCC8 gene deletion mutations causing diazoxide-unresponsive hyperinsulinaemic hypoglycaemia. Pediatr Diabetes 13(3):285-9.

Flanagan SE, De Franco E, Lango Allen H, Zerah M, Abdul-Rasoul MM, Edge JA, Stewart H, Alamiri E, Hussain K, Wallis S and others. 2014. Analysis of transcription factors key for mouse pancreatic development establishes NKX2-2 and MNX1 mutations as causes of neonatal diabetes in man. Cell Metab 19(1):146-54.

Flanagan SE, Dung VC, Houghton JAL, De Franco E, Ngoc CTB, Damhuis A, Ashcroft FM, Harries LW, Ellard S. 2017. An ABCC8 Nonsense Mutation Causing Neonatal Diabetes Through Altered Transcript Expression. J Clin Res Pediatr Endocrinol 9(3):260-264.

Flanagan SE, Edghill EL, Gloyn AL, Ellard S, Hattersley AT. 2006. Mutations in KCNJ11, which encodes Kir6.2, are a common cause of diabetes diagnosed in the first 6 months of life, with the phenotype determined by genotype. Diabetologia 49(6):1190-7.

Flanagan SE, Kapoor RR, Banerjee I, Hall C, Smith VV, Hussain K, Ellard S. 2011. Dominantly acting ABCC8 mutations in patients with medically unresponsive hyperinsulinaemic hypoglycaemia. Clin Genet 79(6):582-7.

Flanagan SE, Patch AM, Ellard S. 2010. Using SIFT and PolyPhen to predict loss-of-function and gain-of-function mutations. Genet Test Mol Biomarkers 14(4):533-7.

Flanagan SE, Patch AM, Mackay DJ, Edghill EL, Gloyn AL, Robinson D, Shield JP, Temple K, Ellard S, Hattersley AT. 2007. Mutations in ATP-sensitive K+ channel genes cause transient neonatal diabetes and permanent diabetes in childhood or adulthood. Diabetes 56(7):1930-7.

Flanagan SE, Xie W, Caswell R, Damhuis A, Vianey-Saban C, Akcay T, Darendeliler F, Bas F, Guven A, Siklar Z and others. 2013. Next-generation sequencing reveals deep intronic cryptic ABCC8 and HADH splicing founder mutations causing hyperinsulinism by pseudoexon activation. Am J Hum Genet 92(1):131-6.

Flechtner I, Vaxillaire M, Cave H, Scharfmann R, Froguel P, Polak M. 2008. Neonatal hyperglycaemia and abnormal development of the pancreas. Best Pract Res Clin Endocrinol Metab 22(1):17-40.

Florez JC, Jablonski KA, Kahn SE, Franks PW, Dabelea D, Hamman RF, Knowler WC, Nathan DM, Altshuler D. 2007. Type 2 diabetes-associated missense polymorphisms KCNJ11 E23K and ABCC8 A1369S influence progression to diabetes and response to interventions in the Diabetes Prevention Program. Diabetes 56(2):531-6.

Fournet JC, Mayaud C, de Lonlay P, Gross-Morand MS, Verkarre V, Castanet M, Devillers M, Rahier J, Brunelle F, Robert JJ and others. 2001. Unbalanced expression of 11p15 imprinted genes in focal forms of congenital hyperinsulinism: association with a reduction to homozygosity of a mutation in ABCC8 or KCNJ11. Am J Pathol 158(6):2177-84.

Fourtner SH, Stanley CA, Kelly A. 2006. Protein-sensitive hypoglycemia without leucine sensitivity in hyperinsulinism caused by K(ATP) channel mutations. J Pediatr 149(1):47-52.

Gaal Z, Klupa T, Kantor I, Mlynarski W, Albert L, Tolloczko J, Balogh I, Czajkowski K, Malecki MT. 2012. Sulfonylurea use during entire pregnancy in diabetes because of KCNJ11 mutation: a report of two cases. Diabetes Care 35(6):e40.

Gach A, Wyka K, Pietrzak I, Wegner O, Malecki MT, Mlynarski W. 2009. Neonatal diabetes in a child positive for islet cell antibodies at onset and Kir6.2 activating mutation. Diabetes Res Clin Pract 86(2):e25-7.

Galcheva S, Iotova V, Ellard S, Flanagan SE, Halvadzhiyan I, Petrova C, Hussain K. 2017. Clinical presentation and treatment response to diazoxide in two siblings with congenital hyperinsulinism as a result of a novel compound heterozygous ABCC8 missense mutation. J Pediatr Endocrinol Metab 30(4):471-474.

Ganesh R, Suresh N, Vasanthi T, Ravikumar KG. 2017. Neonatal Diabetes: A Case Series. Indian Pediatr 54(1):33-36.

Girard CA, Shimomura K, Proks P, Absalom N, Castano L, Perez de Nanclares G, Ashcroft FM. 2006. Functional analysis of six Kir6.2 (KCNJ11) mutations causing neonatal diabetes. Pflugers Arch 453(3):323-32.

Girard CA, Wunderlich FT, Shimomura K, Collins S, Kaizik S, Proks P, Abdulkader F, Clark A, Ball V, Zubcevic L and others. 2009. Expression of an activating mutation in the gene encoding the KATP channel subunit Kir6.2 in mouse pancreatic beta cells recapitulates neonatal diabetes. J Clin Invest 119(1):80-90.

Giurgea I, Laborde K, Touati G, Bellanne-Chantelot C, Nassogne MC, Sempoux C, Jaubert F, Khoa N, Chigot V, Rahier J and others. 2004. Acute insulin responses to calcium and tolbutamide do not differentiate focal from diffuse congenital hyperinsulinism. J Clin Endocrinol Metab 89(2):925-9.

Giurgea I, Sempoux C, Bellanne-Chantelot C, Ribeiro M, Hubert L, Boddaert N, Saudubray JM, Robert JJ, Brunelle F, Rahier J and others. 2006. The Knudson's two-hit model and timing of somatic mutation may account for the phenotypic diversity of focal congenital hyperinsulinism. J Clin Endocrinol Metab 91(10):4118-23.

Glaser B, Ryan F, Donath M, Landau H, Stanley CA, Baker L, Barton DE, Thornton PS. 1999. Hyperinsulinism caused by paternal-specific inheritance of a recessive mutation in the sulfonylurea-receptor gene. Diabetes 48(8):1652-7.

Globa E, Zelinska N, Mackay DJ, Temple KI, Houghton JA, Hattersley AT, Flanagan SE, Ellard S. 2015. Neonatal diabetes in Ukraine: incidence, genetics, clinical phenotype and treatment. J Pediatr Endocrinol Metab 28(11-12):1279-86.

Gloyn AL, Cummings EA, Edghill EL, Harries LW, Scott R, Costa T, Temple IK, Hattersley AT, Ellard S. 2004a. Permanent neonatal diabetes due to paternal germline mosaicism for an activating mutation of the KCNJ11 Gene encoding the Kir6.2 subunit of the beta-cell potassium adenosine triphosphate channel. J Clin Endocrinol Metab 89(8):3932-5.

Gloyn AL, Diatloff-Zito C, Edghill EL, Bellanne-Chantelot C, Nivot S, Coutant R, Ellard S, Hattersley AT, Robert JJ. 2006. KCNJ11 activating mutations are associated with developmental delay, epilepsy and neonatal diabetes syndrome and other neurological features. Eur J Hum Genet 14(7):824-30.

Gloyn AL, Pearson ER, Antcliff JF, Proks P, Bruining GJ, Slingerland AS, Howard N, Srinivasan S, Silva JM, Molnes J and others. 2004b. Activating mutations in the gene encoding the ATP-sensitive potassium-channel subunit Kir6.2 and permanent neonatal diabetes. N Engl J Med 350(18):1838-49.

Gloyn AL, Reimann F, Girard C, Edghill EL, Proks P, Pearson ER, Temple IK, Mackay DJ, Shield JP, Freedenberg D and others. 2005. Relapsing diabetes can result from moderately activating mutations in KCNJ11. Hum Mol Genet 14(7):925-34.

Goksel DL, Fischbach K, Duggirala R, Mitchell BD, Aguilar-Bryan L, Blangero J, Stern MP, O'Connell P. 1998. Variant in sulfonylurea receptor-1 gene is associated with high insulin concentrations in non-diabetic Mexican Americans: SUR-1 gene variant and hyperinsulinemia. Hum Genet 103(3):280-5.

Gole E, Oikonomou S, Ellard S, De Franco E, Karavanaki K. 2018. A Novel KCNJ11 Mutation Associated with Transient Neonatal Diabetes. J Clin Res Pediatr Endocrinol 10(2):175-178.

Gonen MS, Arikoglu H, Erkoc Kaya D, Ozdemir H, Ipekci SH, Arslan A, Kayis SA, Gogebakan B. 2012. Effects of single nucleotide polymorphisms in K(ATP) channel genes on type 2 diabetes in a Turkish population. Arch Med Res 43(4):317-23.

Gong C, Huang S, Su C, Qi Z, Liu F, Wu D, Cao B, Gu Y, Li W, Liang X and others. 2016. Congenital hyperinsulinism in Chinese patients: 5-yr treatment outcome of 95 clinical cases with genetic analysis of 55 cases. Pediatr Diabetes 17(3):227-34.

Gonsorcikova L, Vaxillaire M, Pruhova S, Dechaume A, Dusatkova P, Cinek O, Pedersen O, Froguel P, Hansen T, Lebl J. 2011. Familial mild hyperglycemia associated with a novel ABCC8-V84I mutation within three generations. Pediatr Diabetes 12(3 Pt 2):266-9.

Greer RM, Shah J, Jeske YW, Brown D, Walker RM, Cowley D, Bowling FG, Liaskou D, Harris M, Thomsett MJ and others. 2007. Genotype-phenotype associations in patients with severe hyperinsulinism of infancy. Pediatr Dev Pathol 10(1):25-34.

Grimberg A, Ferry RJ, Jr., Kelly A, Koo-McCoy S, Polonsky K, Glaser B, Permutt MA, Aguilar-Bryan L, Stafford D, Thornton PS and others. 2001. Dysregulation of insulin secretion in children with congenital hyperinsulinism due to sulfonylurea receptor mutations. Diabetes 50(2):322-8.

Gurgel LC, Crispim F, Noffs MH, Belzunces E, Rahal MA, Moises RS. 2007. Sulfonylrea treatment in permanent neonatal diabetes due to G53D mutation in the KCNJ11 gene: improvement in glycemic control and neurological function. Diabetes Care 30(11):e108.

Gussinyer M, Clemente M, Cebrian R, Yeste D, Albisu M, Carrascosa A. 2008. Glucose intolerance and diabetes are observed in the long-term follow-up of nonpancreatectomized patients with persistent hyperinsulinemic hypoglycemia of infancy due to mutations in the ABCC8 gene. Diabetes Care 31(6):1257-9.

Guven A, Cebeci AN, Ellard S, Flanagan SE. 2016. Clinical and Genetic Characteristics, Management and Long-Term Follow-Up of Turkish Patients with Congenital Hyperinsulinism. J Clin Res Pediatr Endocrinol 8(2):197-204.

Haliloglu B, Tuzun H, Flanagan SE, Celik M, Kaya A, Ellard S, Ozbek MN. 2018. Sirolimus-Induced Hepatitis in Two Patients with Hyperinsulinemic Hypoglycemia. J Clin Res Pediatr Endocrinol 10(3):279-283.

Han B, Newbould M, Batra G, Cheesman E, Craigie RJ, Mohamed Z, Rigby L, Padidela R, Skae M, Mironov A and others. 2016. Enhanced Islet Cell Nucleomegaly Defines Diffuse Congenital Hyperinsulinism in Infancy but Not Other Forms of the Disease. Am J Clin Pathol 145(6):757-68.

Hansen T, Echwald SM, Hansen L, Moller AM, Almind K, Clausen JO, Urhammer SA, Inoue H, Ferrer J, Bryan J and others. 1998. Decreased tolbutamide-stimulated insulin secretion in healthy subjects with sequence variants in the high-affinity sulfonylurea receptor gene. Diabetes 47(4):598-605.

Hardy OT, Hernandez-Pampaloni M, Saffer JR, Suchi M, Ruchelli E, Zhuang H, Ganguly A, Freifelder R, Adzick NS, Alavi A and others. 2007. Diagnosis and localization of focal congenital hyperinsulinism by 18F-fluorodopa PET scan. J Pediatr 150(2):140-5.

Harel S, Cohen AS, Hussain K, Flanagan SE, Schlade-Bartusiak K, Patel M, Courtade J, Li JB, Van Karnebeek C, Kurata H and others. 2015. Alternating hypoglycemia and hyperglycemia in a toddler with a homozygous p.R1419H ABCC8 mutation: an unusual clinical picture. J Pediatr Endocrinol Metab 28(3-4):345-51.

Hartemann-Heurtier A, Simon A, Bellanne-Chantelot C, Reynaud R, Cave H, Polak M, Vaxillaire M, Grimaldi A. 2009. Mutations in the ABCC8 gene can cause autoantibody-negative insulin-dependent diabetes. Diabetes Metab 35(3):233-5.

Hashimoto Y, Dateki S, Hirose M, Satomura K, Sawada H, Mizuno H, Sugihara S, Maruyama K, Urakami T, Sugawara H and others. 2017. Molecular and clinical features of KATP -channel neonatal diabetes mellitus in Japan. Pediatr Diabetes 18(7):532-539.

Hashimoto Y, Sakakibara A, Kawakita R, Hosokawa Y, Fujimaru R, Nakamura T, Fukushima H, Igarashi A, Masue M, Nishibori H and others. 2015. Focal form of congenital hyperinsulinism clearly detectable by contrast-enhanced computed tomography imaging. Int J Pediatr Endocrinol 2015(1):20.

Henquin JC, Nenquin M, Sempoux C, Guiot Y, Bellanne-Chantelot C, Otonkoski T, de Lonlay P, Nihoul-Fekete C, Rahier J. 2011. In vitro insulin secretion by pancreatic tissue from infants with diazoxide-resistant congenital hyperinsulinism deviates from model predictions. J Clin Invest 121(10):3932-42.

Henwood MJ, Kelly A, Macmullen C, Bhatia P, Ganguly A, Thornton PS, Stanley CA. 2005. Genotype-phenotype correlations in children with congenital hyperinsulinism due to recessive mutations of the adenosine triphosphate-sensitive potassium channel genes. J Clin Endocrinol Metab 90(2):789-94.

Heo JW, Kim SW, Cho EH. 2013. Unsuccessful switch from insulin to sulfonylurea therapy in permanent neonatal diabetes mellitus due to an R201H mutation in the KCNJ11 gene: a case report. Diabetes Res Clin Pract 100(1):e1-2.

Hoffman TL, Blanco E, Lane A, Galvin-Parton P, Gadi I, Santer R, DeLeon D, Stanley C, Wilson TA. 2007. Glucose metabolism and insulin secretion in a patient with ABCC8 mutation and Fanconi-Bickel syndrome caused by maternal isodisomy of chromosome 3. Clin Genet 71(6):551-7.

Hosy E, Dupuis JP, Vivaudou M. 2010. Impact of disease-causing SUR1 mutations on the KATP channel subunit interface probed with a rhodamine protection assay. J Biol Chem 285(5):3084-91.

Hugill A, Shimomura K, Ashcroft FM, Cox RD. 2010. A mutation in KCNJ11 causing human hyperinsulinism (Y12X) results in a glucose-intolerant phenotype in the mouse. Diabetologia 53(11):2352-6.

Huopio H, Jaaskelainen J, Komulainen J, Miettinen R, Karkkainen P, Laakso M, Tapanainen P, Voutilainen R, Otonkoski T. 2002. Acute insulin response tests for the differential diagnosis of congenital hyperinsulinism. J Clin Endocrinol Metab 87(10):4502-7.

Huopio H, Miettinen PJ, Ilonen J, Nykanen P, Veijola R, Keskinen P, Nanto-Salonen K, Vangipurapu J, Raivo J, Stancakova A and others. 2016. Clinical, Genetic, and Biochemical Characteristics of Early-Onset Diabetes in the Finnish Population. J Clin Endocrinol Metab 101(8):3018-26.

Huopio H, Otonkoski T, Vauhkonen I, Reimann F, Ashcroft FM, Laakso M. 2003. A new subtype of autosomal dominant diabetes attributable to a mutation in the gene for sulfonylurea receptor 1. Lancet 361(9354):301-7.

Huopio H, Reimann F, Ashfield R, Komulainen J, Lenko HL, Rahier J, Vauhkonen I, Kere J, Laakso M, Ashcroft F and others. 2000. Dominantly inherited hyperinsulinism caused by a mutation in the sulfonylurea receptor type 1. J Clin Invest 106(7):897-906.

Hussain K, Flanagan SE, Smith VV, Ashworth M, Day M, Pierro A, Ellard S. 2008. An ABCC8 gene mutation and mosaic uniparental isodisomy resulting in atypical diffuse congenital hyperinsulinism. Diabetes 57(1):259-63.

Iafusco D, Massa O, Pasquino B, Colombo C, Iughetti L, Bizzarri C, Mammi C, Lo Presti D, Suprani T, Schiaffini R and others. 2012. Minimal incidence of neonatal/infancy onset diabetes in Italy is 1:90,000 live births. Acta Diabetol 49(5):405-8.

Ilamaran V, Venkatesh C, Manish K, Adhisivam B. 2010. Persistent hyperinsulinemic hypoglycemia of infancy due to homozygous KCNJ11 (T294M) mutation. Indian J Pediatr 77(7):803-4.

Ille J, Putarek NR, Radica A, Hattersley A, Ellard S, Dumic M. 2010. [Low doses of sulphonyluria as a successful replacement for insulin therapy in a patient with neonatal diabetes due to a mutation of KCNJ11 gene encoding Kir6.2]. Lijec Vjesn 132(3-4):90-3.

Ince DA, Sahin NM, Ecevit A, Kurt A, Kinik ST, Flanagan SE, Hussain K, Tarcan A. 2014. Congenital hyperinsulinism in a newborn with a novel homozygous mutation (p.Q392H) in the ABCC8 gene. J Pediatr Endocrinol Metab 27(11-12):1253-5.

Inoue H, Ferrer J, Warren-Perry M, Zhang Y, Millns H, Turner RC, Elbein SC, Hampe CL, Suarez BK, Inagaki N and others. 1997. Sequence variants in the pancreatic islet beta-cell inwardly rectifying K+ channel Kir6.2 (Bir) gene: identification and lack of role in Caucasian patients with NIDDM. Diabetes 46(3):502-7.

Inoue H, Ferrer J, Welling CM, Elbein SC, Hoffman M, Mayorga R, Warren-Perry M, Zhang Y, Millns H, Turner R and others. 1996. Sequence variants in the sulfonylurea receptor (SUR) gene are associated with NIDDM in Caucasians. Diabetes 45(6):825-31.

Ioacara S, Flanagan S, Frohlich-Reiterer E, Goland R, Fica S. 2017. First case of neonatal diabetes with KCNJ11 Q52R mutation successfully switched from insulin to sulphonylurea treatment. J Diabetes Investig 8(5):716-719.

Ioannou YS, Ellard S, Hattersley A, Skordis N. 2011. KCNJ11 activating mutations cause both transient and permanent neonatal diabetes mellitus in Cypriot patients. Pediatr Diabetes 12(2):133-7.

Irgens HU, Molnes J, Johansson BB, Ringdal M, Skrivarhaug T, Undlien DE, Sovik O, Joner G, Molven A, Njolstad PR. 2013. Prevalence of monogenic diabetes in the population-based Norwegian Childhood Diabetes Registry. Diabetologia 56(7):1512-9.

Isik E, Demirbilek H, Houghton JA, Ellard S, Flanagan SE, Hussain K. 2019. Congenital Hyperinsulinism and Evolution to Sulfonylurearesponsive Diabetes Later in Life due to a Novel Homozygous p.L171F ABCC8 Mutation. J Clin Res Pediatr Endocrinol 11(1):82-87.

Ismail D, Kapoor RR, Smith VV, Ashworth M, Blankenstein O, Pierro A, Flanagan SE, Ellard S, Hussain K. 2012. The heterogeneity of focal forms of congenital hyperinsulinism. J Clin Endocrinol Metab 97(1):E94-9.

Ismail D, Smith VV, de Lonlay P, Ribeiro MJ, Rahier J, Blankenstein O, Flanagan SE, Bellanne-Chantelot C, Verkarre V, Aigrain Y and others. 2011. Familial focal congenital hyperinsulinism. J Clin Endocrinol Metab 96(1):24-8.

Itoh S, Matsuoka H, Yasuda Y, Miyake N, Suzuki K, Yorifuji T, Sugihara S. 2013. DEND syndrome due to V59A mutation in KCNJ11 gene: unresponsive to sulfonylureas. J Pediatr Endocrinol Metab 26(1-2):143-6.

Jahnavi S, Poovazhagi V, Kanthimathi S, Balamurugan K, Bodhini D, Yadav J, Jain V, Khadgawat R, Sikdar M, Bhavatharini A and others. 2014. Novel ABCC8 (SUR1) gene mutations in Asian Indian children with congenital hyperinsulinemic hypoglycemia. Ann Hum Genet 78(5):311-9.

Jahnavi S, Poovazhagi V, Mohan V, Bodhini D, Raghupathy P, Amutha A, Suresh Kumar P, Adhikari P, Shriraam M, Kaur T and others. 2013. Clinical and molecular characterization of neonatal diabetes and monogenic syndromic diabetes in Asian Indian children. Clin Genet 83(5):439-45.

Jain V, Flanagan SE, Ellard S. 2012. Permanent neonatal diabetes caused by a novel mutation. Indian Pediatr 49(6):486-8.

Jeron A, Hengstenberg C, Holmer S, Wollnik B, Riegger GA, Schunkert H, Erdmann J. 2004. KCNJ11 polymorphisms and sudden cardiac death in patients with acute myocardial infarction. J Mol Cell Cardiol 36(2):287-93.

Jesic MM, Jesic MD, Maglajlic S, Sajic S, Necic S. 2011. Successful sulfonylurea treatment of a neonate with neonatal diabetes mellitus due to a new KCNJ11 mutation. Diabetes Res Clin Pract 91(1):e1-3.

Jindal R, Ahmad A, Siddiqui MA, Kochar IS, Wangnoo SK. 2014. Novel mutation c.597_598dup in exon 5 of ABCC8 gene causing congenital hyperinsulinism. Diabetes Metab Syndr 8(1):45-7.

Johansson S, Irgens H, Chudasama KK, Molnes J, Aerts J, Roque FS, Jonassen I, Levy S, Lima K, Knappskog PM and others. 2012. Exome sequencing and genetic testing for MODY. PLoS One 7(5):e38050.

John SA, Weiss JN, Ribalet B. 2001. Regulation of cloned ATP-sensitive K channels by adenine nucleotides and sulfonylureas: interactions between SUR1 and positively charged domains on Kir6.2. J Gen Physiol 118(4):391-405.

Jones AG, Hattersley AT. 2010. Reevaluation of a case of type 1 diabetes mellitus diagnosed before 6 months of age. Nat Rev Endocrinol 6(6):347-51.

Jose BB, T. Hattersley, A.T. Milles, J.J. 2009. Glibenclamide controls ketosis-prone diabetes in a 38-year-old woman with Kir6.2 mutation. Practical Diabetes International 26(6):3.

Joshi R, Phatarpekar A. 2011. Neonatal diabetes mellitus due to L233F mutation in the KCNJ11 gene. World J Pediatr 7(4):371-2.

Kalish JM, Boodhansingh KE, Bhatti TR, Ganguly A, Conlin LK, Becker SA, Givler S, Mighion L, Palladino AA, Adzick NS and others. 2016. Congenital hyperinsulinism in children with paternal 11p uniparental isodisomy and Beckwith-Wiedemann syndrome. J Med Genet 53(1):53-61.

Kanakatti Shankar R, Pihoker C, Dolan LM, Standiford D, Badaru A, Dabelea D, Rodriguez B, Black MH, Imperatore G, Hattersley A and others. 2013. Permanent neonatal diabetes mellitus: prevalence and genetic diagnosis in the SEARCH for Diabetes in Youth Study. Pediatr Diabetes 14(3):174-80.

Kapoor RR, Flanagan SE, Arya VB, Shield JP, Ellard S, Hussain K. 2013. Clinical and molecular characterisation of 300 patients with congenital hyperinsulinism. Eur J Endocrinol 168(4):557-64.

Kapoor RR, Flanagan SE, Ellard S, Hussain K. 2012. Congenital hyperinsulinism: marked clinical heterogeneity in siblings with identical mutations in the ABCC8 gene. Clin Endocrinol (Oxf) 76(2):312-3.

Kapoor RR, Flanagan SE, James CT, McKiernan J, Thomas AM, Harmer SC, Shield JP, Tinker A, Ellard S, Hussain K. 2011. Hyperinsulinaemic hypoglycaemia and diabetes mellitus due to dominant ABCC8/KCNJ11 mutations. Diabetologia 54(10):2575-83.

Karges B, Schnur D, Ellard S, Kentrup H, Karges W. 2012. Effective treatment of diabetes caused by activating ABCC8/SUR1 mutation with glimepiride. Diabet Med 29(5):692-3.

Kassem SA, Ariel I, Thornton PS, Hussain K, Smith V, Lindley KJ, Aynsley-Green A, Glaser B. 2001. p57(KIP2) expression in normal islet cells and in hyperinsulinism of infancy. Diabetes 50(12):2763-9.

Katanic D, Vorgucin I, Hattersley A, Ellard S, Houghton JAL, Obreht D, Knezevic Pogancev M, Vlaski J, Pavkov D. 2017. A successful transition to sulfonylurea treatment in male infant with neonatal diabetes caused by the novel abcc8 gene mutation and three years follow-up. Diabetes Res Clin Pract 129:59-61.

Khadilkar VV, Khadilkar AV, Kapoor RR, Hussain K, Hattersley AT, Ellard S. 2010. KCNJ11 activating mutation in an Indian family with remitting and relapsing diabetes. Indian J Pediatr 77(5):551-4.

Khawash P, Hussain K, Flanagan SE, Chatterjee S, Basak D. 2015. Nifedipine in Congenital Hyperinsulinism - A Case Report. J Clin Res Pediatr Endocrinol 7(2):151-4.

Khoriati D, Arya VB, Flanagan SE, Ellard S, Hussain K. 2013. Prematurity, macrosomia, hyperinsulinaemic hypoglycaemia and a dominant ABCC8 gene mutation. BMJ Case Rep 2013.

Khurana A, Shao ES, Kim RY, Vilin YY, Huang X, Yang R, Kurata HT. 2011. Forced gating motions by a substituted titratable side chain at the bundle crossing of a potassium channel. J Biol Chem 286(42):36686-93.

Khurana D, Contreras M, Malhotra N, Bargman R. 2012. The diagnosis of neonatal diabetes in a mother at 25 years of age. Diabetes Care 35(8):e59.

Kiff S, Babb C, Guemes M, Dastamani A, Gilbert C, Flanagan SE, Ellard S, Barton J, Dattani M, Shah P. 2019. Partial diazoxide responsiveness in a neonate with hyperinsulinism due to homozygous ABCC8 mutation. Endocrinol Diabetes Metab Case Rep 2019.

Kim MS, Kim SY, Kim GH, Yoo HW, Lee DW, Lee DY. 2007. Sulfonylurea therapy in two Korean patients with insulin-treated neonatal diabetes due to heterozygous mutations of the KCNJ11 gene encoding Kir6.2. J Korean Med Sci 22(4):616-20.

Klee P, Bellanne-Chantelot C, Depret G, Llano JP, Paget C, Nicolino M. 2012. A novel ABCC8 mutation illustrates the variability of the diabetes phenotypes associated with a single mutation. Diabetes Metab 38(2):179-82.

Klupa T, Edghill EL, Nazim J, Sieradzki J, Ellard S, Hattersley AT, Malecki MT. 2005. The identification of a R201H mutation in KCNJ11, which encodes Kir6.2, and successful transfer to sustained-release sulphonylurea therapy in a subject with neonatal diabetes: evidence for heterogeneity of beta cell function among carriers of the R201H mutation. Diabetologia 48(5):1029-31.

Klupa T, Kowalska I, Wyka K, Skupien J, Patch AM, Flanagan SE, Noczynska A, Arciszewska M, Ellard S, Hattersley AT and others. 2009. Mutations in the ABCC8 (SUR1 subunit of the K(ATP) channel) gene are associated with a variable clinical phenotype. Clin Endocrinol (Oxf) 71(3):358-62.

Klupa T, Kozek E, Nowak N, Cyganek K, Gach A, Milewicz T, Czajkowski K, Tolloczko J, Mlynarski W, Malecki MT. 2010a. The first case report of sulfonylurea use in a woman with permanent neonatal diabetes mellitus due to KCNJ11 mutation during a high-risk pregnancy. J Clin Endocrinol Metab 95(8):3599-604.

Klupa T, Skupien J, Mirkiewicz-Sieradzka B, Gach A, Noczynska A, Zubkiewicz-Kucharska A, Szalecki M, Kozek E, Nazim J, Mlynarski W and others. 2010b. Efficacy and safety of sulfonylurea use in permanent neonatal diabetes due to KCNJ11 gene mutations: 34-month median follow-up. Diabetes Technol Ther 12(5):387-91.

Kocaay P, Siklar Z, Ellard S, Yagmurlu A, Camtosun E, Erden E, Berberoglu M, Flanagan SE. 2016. Coexistence of Mosaic Uniparental Isodisomy and a KCNJ11 Mutation Presenting as Diffuse Congenital Hyperinsulinism and Hemihypertrophy. Horm Res Paediatr 85(6):421-5.

Kochar IP, Kulkarni KP. 2010. Transient Neonatal Diabetes due to Kcnj11 Mutation. Indian Pediatr 47(4):359-60.

Kong JH, Kim JB. 2011. Transient neonatal diabetes mellitus caused by a de novoABCC8 gene mutation. Korean J Pediatr 54(4):179-82.

Korula S, Chapla A, Priyambada L, Mathai S, Simon A. 2018. Sirolimus therapy for congenital hyperinsulinism in an infant with a novel homozygous KCNJ11 mutation. J Pediatr Endocrinol Metab 31(1):87-89.

Koster JC, Kurata HT, Enkvetchakul D, Nichols CG. 2008. DEND mutation in Kir6.2 (KCNJ11) reveals a flexible N-terminal region critical for ATP-sensing of the KATP channel. Biophys J 95(10):4689-97.

Koster JC, Remedi MS, Dao C, Nichols CG. 2005. ATP and sulfonylurea sensitivity of mutant ATP-sensitive K+ channels in neonatal diabetes: implications for pharmacogenomic therapy. Diabetes 54(9):2645-54.

Koufakis T, Sertedaki A, Tatsi EB, Trakatelli CM, Karras SN, Manthou E, Kanaka-Gantenbein C, Kotsa K. 2019. First Report of Diabetes Phenotype due to a Loss-of-Function ABCC8 Mutation Previously Known to Cause Congenital Hyperinsulinism. Case Rep Genet 2019:3654618.

Kumaraguru J, Flanagan SE, Greeley SA, Nuboer R, Stoy J, Philipson LH, Hattersley AT, Rubio-Cabezas O. 2009. Tooth discoloration in patients with neonatal diabetes after transfer onto glibenclamide: a previously unreported side effect. Diabetes Care 32(8):1428-30.

Kumaran A, Kapoor RR, Flanagan SE, Ellard S, Hussain K. 2010. Congenital hyperinsulinism due to a compound heterozygous ABCC8 mutation with spontaneous resolution at eight weeks. Horm Res Paediatr 73(4):287-92.

Lahmann C, Clark RH, Iberl M, Ashcroft FM. 2014. A mutation causing increased KATP channel activity leads to reduced anxiety in mice. Physiol Behav 129:79-84.

Landau Z, Wainstein J, Hanukoglu A, Tuval M, Lavie J, Glaser B. 2007. Sulfonylurea-responsive diabetes in childhood. J Pediatr 150(5):553-5.

Lau E, Correia C, Freitas P, Nogueira C, Costa M, Saavedra A, Costa C, Carvalho D, Fontoura M. 2015. Permanent neonatal diabetes by a new mutation in KCNJ11: unsuccessful switch to sulfonylurea. Arch Endocrinol Metab 59(6):559-61.

Lauridsen MH, Boesgaard TW, Pedersen OB, Hansen T, Hertz B. 2009. [Diabetes in infants may be treated with sulfonylurea as a replacement for insulin]. Ugeskr Laeger 171(23):1923-4.

Lee BH, Lee J, Kim JM, Kang M, Kim GH, Choi JH, Kim J, Kim CJ, Kim DY, Kim SC and others. 2015. Three novel pathogenic mutations in KATP channel genes and somatic imprinting alterations of the 11p15 region in pancreatic tissue in patients with congenital hyperinsulinism. Horm Res Paediatr 83(3):204-10.

Lek M, Karczewski KJ, Minikel EV, Samocha KE, Banks E, Fennell T, O'Donnell-Luria AH, Ware JS, Hill AJ, Cummings BB and others. 2016. Analysis of protein-coding genetic variation in 60,706 humans. Nature 536(7616):285-91.

Letha S, Mammen D, Valamparampil JJ. 2007. Permanent neonatal diabetes due to KCNJ11 gene mutation. Indian J Pediatr 74(10):947-9.

Li C, Ackermann AM, Boodhansingh KE, Bhatti TR, Liu C, Schug J, Doliba N, Han B, Cosgrove KE, Banerjee I and others. 2017. Functional and Metabolomic Consequences of KATP Channel Inactivation in Human Islets. Diabetes 66(7):1901-1913.

Lin CW, Lin YW, Yan FF, Casey J, Kochhar M, Pratt EB, Shyng SL. 2006a. Kir6.2 mutations associated with neonatal diabetes reduce expression of ATP-sensitive K+ channels: implications in disease mechanism and sulfonylurea therapy. Diabetes 55(6):1738-46.

Lin YW, Akrouh A, Hsu Y, Hughes N, Nichols CG, De Leon DD. 2012. Compound heterozygous mutations in the SUR1 (ABCC 8) subunit of pancreatic K(ATP) channels cause neonatal diabetes by perturbing the coupling between Kir6.2 and SUR1 subunits. Channels (Austin) 6(2):133-8.

Lin YW, Bushman JD, Yan FF, Haidar S, MacMullen C, Ganguly A, Stanley CA, Shyng SL. 2008. Destabilization of ATP-sensitive potassium channel activity by novel KCNJ11 mutations identified in congenital hyperinsulinism. J Biol Chem 283(14):9146-56.

Lin YW, Li A, Grasso V, Battaglia D, Crino A, Colombo C, Barbetti F, Nichols CG. 2013. Functional characterization of a novel KCNJ11 in frame mutation-deletion associated with infancy-onset diabetes and a mild form of intermediate DEND: a battle between K(ATP) gain of channel activity and loss of channel expression. PLoS One 8(5):e63758.

Lin YW, MacMullen C, Ganguly A, Stanley CA, Shyng SL. 2006b. A novel KCNJ11 mutation associated with congenital hyperinsulinism reduces the intrinsic open probability of beta-cell ATP-sensitive potassium channels. J Biol Chem 281(5):3006-12.

Liu L, Nagashima K, Yasuda T, Liu Y, Hu HR, He G, Feng B, Zhao M, Zhuang L, Zheng T and others. 2013. Mutations in KCNJ11 are associated with the development of autosomal dominant, early-onset type 2 diabetes. Diabetologia 56(12):2609-18.

Loechner KJ, Akrouh A, Kurata HT, Dionisi-Vici C, Maiorana A, Pizzoferro M, Rufini V, de Ville de Goyet J, Colombo C, Barbetti F and others. 2011. Congenital hyperinsulinism and glucose hypersensitivity in homozygous and heterozygous carriers of Kir6.2 (KCNJ11) mutation V290M mutation: K(ATP) channel inactivation mechanism and clinical management. Diabetes 60(1):209-17.

Macmullen CM, Zhou Q, Snider KE, Tewson PH, Becker SA, Aziz AR, Ganguly A, Shyng SL, Stanley CA. 2011. Diazoxide-unresponsive congenital hyperinsulinism in children with dominant mutations of the beta-cell sulfonylurea receptor SUR1. Diabetes 60(6):1797-804.

Madani HE, R.; Alkholy, B.; Musa, N.; Shaalan, Y.; Elkaffas, R.; Hassan, M.; Hafez, M.; Flanagan, S.E.; De Franco, E.; Hussain, K. 2019. Identification of novel variants in neonatal diabetes mellitus genes in Egyptian patients with permanent NDM. International Journal of Diabetes in Developing Countries 39(1):53-59.

Maejima Y, Hasegawa S, Horita S, Kumamoto K, Galvanovskis J, Takenoshita S, Shimomura K. 2015. Water intake disorder in a DEND syndrome afflicted patient with R50P mutation. Endocr J 62(4):387-92.

Magge SN, Shyng SL, MacMullen C, Steinkrauss L, Ganguly A, Katz LE, Stanley CA. 2004. Familial leucine-sensitive hypoglycemia of infancy due to a dominant mutation of the beta-cell sulfonylurea receptor. J Clin Endocrinol Metab 89(9):4450-6.

Maiorana A, Barbetti F, Boiani A, Rufini V, Pizzoferro M, Francalanci P, Faletra F, Nichols CG, Grimaldi C, de Ville de Goyet J and others. 2014. Focal congenital hyperinsulinism managed by medical treatment: a diagnostic algorithm based on molecular genetic screening. Clin Endocrinol (Oxf) 81(5):679-88.

Mak CM, Lee CY, Lam CW, Siu WK, Hung VC, Chan AY. 2012. Personalized medicine switching from insulin to sulfonylurea in permanent neonatal diabetes mellitus dictated by a novel activating ABCC8 mutation. Diagn Mol Pathol 21(1):56-9.

Mankouri J, Taneja TK, Smith AJ, Ponnambalam S, Sivaprasadarao A. 2006. Kir6.2 mutations causing neonatal diabetes prevent endocytosis of ATP-sensitive potassium channels. EMBO J 25(17):4142-51.

Mannikko R, Flanagan SE, Sim X, Segal D, Hussain K, Ellard S, Hattersley AT, Ashcroft FM. 2011a. Mutations of the same conserved glutamate residue in NBD2 of the sulfonylurea receptor 1 subunit of the KATP channel can result in either hyperinsulinism or neonatal diabetes. Diabetes 60(6):1813-22.

Mannikko R, Jefferies C, Flanagan SE, Hattersley A, Ellard S, Ashcroft FM. 2010. Interaction between mutations in the slide helix of Kir6.2 associated with neonatal diabetes and neurological symptoms. Hum Mol Genet 19(6):963-72.

Mannikko R, Stansfeld PJ, Ashcroft AS, Hattersley AT, Sansom MS, Ellard S, Ashcroft FM. 2011b. A conserved tryptophan at the membrane-water interface acts as a gatekeeper for Kir6.2/SUR1 channels and causes neonatal diabetes when mutated. J Physiol 589(Pt 13):3071-83.

Marshall BA, Green RP, Wambach J, White NH, Remedi MS, Nichols CG. 2015. Remission of severe neonatal diabetes with very early sulfonylurea treatment. Diabetes Care 38(3):e38-9.

Marthinet E, Bloc A, Oka Y, Tanizawa Y, Wehrle-Haller B, Bancila V, Dubuis JM, Philippe J, Schwitzgebel VM. 2005. Severe congenital hyperinsulinism caused by a mutation in the Kir6.2 subunit of the adenosine triphosphate-sensitive potassium channel impairing trafficking and function. J Clin Endocrinol Metab 90(9):5401-6.

Martin-Frias M, Colino E, Perez de Nanclares G, Alonso M, Ros P, Barrio R. 2009. Glibenclamide treatment in relapsed transient neonatal diabetes as a result of a KCNJ11 activating mutation (N48D). Diabet Med 26(5):567-9.

Martinez R, Fernandez-Ramos C, Vela A, Velayos T, Aguayo A, Urrutia I, Rica I, Castano L, Spanish Congenital Hyperinsulinism G. 2016. Clinical and genetic characterization of congenital hyperinsulinism in Spain. Eur J Endocrinol 174(6):717-26.

Martins L, Lourenco R, Maia AL, Maciel P, Monteiro MI, Pacheco L, Anselmo J, Cesar R, Gomes MF. 2015. Transient neonatal diabetes due to a missense mutation (E227K) in the gene encoding the ATP-sensitive potassium channel (KCNJ11). Clin Case Rep 3(10):781-5.

Masia R, De Leon DD, MacMullen C, McKnight H, Stanley CA, Nichols CG. 2007a. A mutation in the TMD0-L0 region of sulfonylurea receptor-1 (L225P) causes permanent neonatal diabetes mellitus (PNDM). Diabetes 56(5):1357-62.

Masia R, Koster JC, Tumini S, Chiarelli F, Colombo C, Nichols CG, Barbetti F. 2007b. An ATP-binding mutation (G334D) in KCNJ11 is associated with a sulfonylurea-insensitive form of developmental delay, epilepsy, and neonatal diabetes. Diabetes 56(2):328-36.

Massa O, Iafusco D, D'Amato E, Gloyn AL, Hattersley AT, Pasquino B, Tonini G, Dammacco F, Zanette G, Meschi F and others. 2005. KCNJ11 activating mutations in Italian patients with permanent neonatal diabetes. Hum Mutat 25(1):22-7.

Matsuo M, Trapp S, Tanizawa Y, Kioka N, Amachi T, Oka Y, Ashcroft FM, Ueda K. 2000. Functional analysis of a mutant sulfonylurea receptor, SUR1-R1420C, that is responsible for persistent hyperinsulinemic hypoglycemia of infancy. J Biol Chem 275(52):41184-91.

McTaggart JS, Jenkinson N, Brittain JS, Greeley SA, Hattersley AT, Ashcroft FM. 2013. Gain-of-function mutations in the K(ATP) channel (KCNJ11) impair coordinated hand-eye tracking. PLoS One 8(4):e62646.

Meder U, Bokodi G, Balogh L, Korner A, Szabo M, Pruhova S, Szabo AJ. 2015. Severe Hyperinsulinemic Hypoglycemia in a Neonate: Response to Sirolimus Therapy. Pediatrics 136(5):e1369-72.

Meissner T, Brune W, Mayatepek E. 1997. Persistent hyperinsulinaemic hypoglycaemia of infancy: therapy, clinical outcome and mutational analysis. Eur J Pediatr 156(10):754-7.

Minute M, Patti G, Tornese G, Faleschini E, Zuiani C, Ventura A. 2015. Sirolimus Therapy in Congenital Hyperinsulinism: A Successful Experience Beyond Infancy. Pediatrics 136(5):e1373-6.

Mlynarski W, Tarasov AI, Gach A, Girard CA, Pietrzak I, Zubcevic L, Kusmierek J, Klupa T, Malecki MT, Ashcroft FM. 2007. Sulfonylurea improves CNS function in a case of intermediate DEND syndrome caused by a mutation in KCNJ11. Nat Clin Pract Neurol 3(11):640-5.

Mohamadi A, Clark LM, Lipkin PH, Mahone EM, Wodka EL, Plotnick LP. 2009. Medical and developmental impact of transition from subcutaneous insulin to oral glyburide in a 15-yr-old boy with neonatal diabetes mellitus and intermediate DEND syndrome: extending the age of KCNJ11 mutation testing in neonatal DM. Pediatr Diabetes.

Mohan V, Radha V, Nguyen TT, Stawiski EW, Pahuja KB, Goldstein LD, Tom J, Anjana RM, Kong-Beltran M, Bhangale T and others. 2018. Comprehensive genomic analysis identifies pathogenic variants in maturity-onset diabetes of the young (MODY) patients in South India. BMC Med Genet 19(1):22.

Mohnike K, Wieland I, Barthlen W, Vogelgesang S, Empting S, Mohnike W, Meissner T, Zenker M. 2014. Clinical and genetic evaluation of patients with KATP channel mutations from the German registry for congenital hyperinsulinism. Horm Res Paediatr 81(3):156-68.

Moreira MC, Piazzon FB, Carvalho MD, Quaio CR, Dutra AB, Ceccon ME, Della-Manna T, Tannuri U, Lee JH, Zerbini MC and others. 2013. A dominant ABCC8-related hyperinsulinism: familial case report. Moreira et al. ABCC8-related hyperinsulinism. Fetal Pediatr Pathol 32(5):384-6.

Moritani M, Yokota I, Tsubouchi K, Takaya R, Takemoto K, Minamitani K, Urakami T, Kawamura T, Kikuchi N, Itakura M and others. 2013. Identification of INS and KCNJ11 gene mutations in type 1B diabetes in Japanese children with onset of diabetes before 5 years of age. Pediatr Diabetes 14(2):112-20.

Mukherjee S, Rastogi A, Venkatesan R, Sundaramoorthi G, Mohan V, Bhansali A. 2017. An infant with diabetes mellitus: Is it always T1DM? Diabetes Res Clin Pract 125:62-64.

Muzyamba M, Farzaneh T, Behe P, Thomas A, Christesen HB, Brusgaard K, Hussain K, Tinker A. 2007. Complex ABCC8 DNA variations in congenital hyperinsulinism: lessons from functional studies. Clin Endocrinol (Oxf) 67(1):115-24.

Myngheer N, Allegaert K, Hattersley A, McDonald T, Kramer H, Ashcroft FM, Verhaeghe J, Mathieu C, Casteels K. 2014. Fetal macrosomia and neonatal hyperinsulinemic hypoglycemia associated with transplacental transfer of sulfonylurea in a mother with KCNJ11-related neonatal diabetes. Diabetes Care 37(12):3333-5.

Nagano N, Urakami T, Mine Y, Watanabe H, Yoshida A, Suzuki J, Saito H, Ishige M, Takahashi S, Mugishima H and others. 2012. Diabetes caused by Kir6.2 mutation: successful treatment with oral glibenclamide switched from continuous subcutaneous insulin infusion in the early phase of the disease. Pediatr Int 54(2):277-9.

Natarajan G, Aggarwal S, Merritt TA. 2007. A novel mutation associated with congenital hyperinsulinism. Am J Perinatol 24(7):401-4.

Nessa A, Aziz QH, Thomas AM, Harmer SC, Tinker A, Hussain K. 2015. Molecular mechanisms of congenital hyperinsulinism due to autosomal dominant mutations in ABCC8. Hum Mol Genet 24(18):5142-53.

Nestorowicz A, Glaser B, Wilson BA, Shyng SL, Nichols CG, Stanley CA, Thornton PS, Permutt MA. 1998. Genetic heterogeneity in familial hyperinsulinism. Hum Mol Genet 7(7):1119-28.

Nestorowicz A, Inagaki N, Gonoi T, Schoor KP, Wilson BA, Glaser B, Landau H, Stanley CA, Thornton PS, Seino S and others. 1997. A nonsense mutation in the inward rectifier potassium channel gene, Kir6.2, is associated with familial hyperinsulinism. Diabetes 46(11):1743-8.

Nestorowicz A, Wilson BA, Schoor KP, Inoue H, Glaser B, Landau H, Stanley CA, Thornton PS, Clement JPt, Bryan J and others. 1996. Mutations in the sulonylurea receptor gene are associated with familial hyperinsulinism in Ashkenazi Jews. Hum Mol Genet 5(11):1813-22.

Ni J, Ge J, Zhang M, Hussain K, Guan Y, Cheng R, Xi L, Zheng Z, Ren S, Luo F. 2019. Genotype and phenotype analysis of a cohort of patients with congenital hyperinsulinism based on DOPA-PET CT scanning. Eur J Pediatr 178(8):1161-1169.

Nichols CG, Shyng SL, Nestorowicz A, Glaser B, Clement JPt, Gonzalez G, Aguilar-Bryan L, Permutt MA, Bryan J. 1996. Adenosine diphosphate as an intracellular regulator of insulin secretion. Science 272(5269):1785-7.

Nieves-Rivera F, Gonzalez-Pijem L. 2011. Neonatal diabetes mellitus: description of two Puerto Rican children with KCNJ11 activating gene mutation. P R Health Sci J 30(2):87-9.

Nikolac N, Simundic AM, Saracevic A, Katalinic D. 2012. ABCC8 polymorphisms are associated with triglyceride concentration in type 2 diabetics on sulfonylurea therapy. Genet Test Mol Biomarkers 16(8):924-30.

Nyangabyaki-Twesigye C, Muhame MR, Bahendeka S. 2015. Permanent neonatal diabetes mellitus - a case report of a rare cause of diabetes mellitus in East Africa. Afr Health Sci 15(4):1339-41.

O'Connell SM, Proks P, Kramer H, Mattis KK, Sachse G, Joyce C, Houghton JA, Ellard S, Hattersley AT, Ashcroft FM and others. 2015. The value of in vitro studies in a case of neonatal diabetes with a novel Kir6.2-W68G mutation. Clin Case Rep 3(10):884-7.

Ocal G, Flanagan SE, Hacihamdioglu B, Berberoglu M, Siklar Z, Ellard S, Savas Erdeve S, Okulu E, Akin IM, Atasay B and others. 2011. Clinical characteristics of recessive and dominant congenital hyperinsulinism due to mutation(s) in the ABCC8/KCNJ11 genes encoding the ATP-sensitive potasium channel in the pancreatic beta cell. J Pediatr Endocrinol Metab 24(11-12):1019-23.

Ohkubo K, Nagashima M, Naito Y, Taguchi T, Suita S, Okamoto N, Fujinaga H, Tsumura K, Kikuchi K, Ono J. 2005. Genotypes of the pancreatic beta-cell K-ATP channel and clinical phenotypes of Japanese patients with persistent hyperinsulinaemic hypoglycaemia of infancy. Clin Endocrinol (Oxf) 62(4):458-65.

Ohta Y, Tanizawa Y, Inoue H, Hosaka T, Ueda K, Matsutani A, Repunte VP, Yamada M, Kurachi Y, Bryan J and others. 1998. Identification and functional analysis of sulfonylurea receptor 1 variants in Japanese patients with NIDDM. Diabetes 47(3):476-81.

Oka H, Suzuki S, Furuya A, Matsuo K, Amamiya S, Oshima M, Oka T, Mukai T, Okayama A, Araki A and others. 2014. Glycemic control and motor development in a patient with intermediate DEND. Pediatr Int 56(3):432-5.

Ooi HL, Wu LL. 2012. Three cases of permanent neonatal diabetes mellitus: genotypes and management outcome. Singapore Med J 53(7):e142-4.

Orio Hernandez M, de la Serna Martinez M, Gonzalez Casado I, Lapunzina P, Gracia Bouthelier R. 2008. [Neonatal diabetes mellitus and KCNJ11 gene mutation: report of a family case]. An Pediatr (Barc) 68(6):602-4.

Ortiz D, Bryan J. 2015. Neonatal Diabetes and Congenital Hyperinsulinism Caused by Mutations in ABCC8/SUR1 are Associated with Altered and Opposite Affinities for ATP and ADP. Front Endocrinol (Lausanne) 6:48.

Ortiz D, Gossack L, Quast U, Bryan J. 2013. Reinterpreting the action of ATP analogs on K(ATP) channels. J Biol Chem 288(26):18894-902.

Ortiz D, Voyvodic P, Gossack L, Quast U, Bryan J. 2012. Two neonatal diabetes mutations on transmembrane helix 15 of SUR1 increase affinity for ATP and ADP at nucleotide binding domain 2. J Biol Chem 287(22):17985-95.

Otonkoski T, Ammala C, Huopio H, Cote GJ, Chapman J, Cosgrove K, Ashfield R, Huang E, Komulainen J, Ashcroft FM and others. 1999. A point mutation inactivating the sulfonylurea receptor causes the severe form of persistent hyperinsulinemic hypoglycemia of infancy in Finland. Diabetes 48(2):408-15.

Otonkoski T, Nanto-Salonen K, Seppanen M, Veijola R, Huopio H, Hussain K, Tapanainen P, Eskola O, Parkkola R, Ekstrom K and others. 2006. Noninvasive diagnosis of focal hyperinsulinism of infancy with [18F]-DOPA positron emission tomography. Diabetes 55(1):13-8.

Ozsu E, Giri D, Seymen Karabulut G, Senniappan S. 2016. Successful transition to sulfonylurea therapy in two Iraqi siblings with neonatal diabetes mellitus and iDEND syndrome due to ABCC8 mutation. J Pediatr Endocrinol Metab 29(12):1403-1406.

Oztekin O, Durmaz E, Kalay S, Flanagan SE, Ellard S, Bircan I. 2012. Successful sulfonylurea treatment of a neonate with neonatal diabetes mellitus due to a novel missense mutation, p.P1199L, in the ABCC8 gene. J Perinatol 32(8):645-7.

Park JS, Lee HJ, Park CH. 2016. A novel mutation of ABCC8 gene in a patient with diazoxide-unresponsive congenital hyperinsulinism. Korean J Pediatr 59(Suppl 1):S116-S120.

Park SE, Flanagan SE, Hussain K, Ellard S, Shin CH, Yang SW. 2011. Characterization of ABCC8 and KCNJ11 gene mutations and phenotypes in Korean patients with congenital hyperinsulinism. Eur J Endocrinol 164(6):919-26.

Partridge CJ, Beech DJ, Sivaprasadarao A. 2001. Identification and pharmacological correction of a membrane trafficking defect associated with a mutation in the sulfonylurea receptor causing familial hyperinsulinism. J Biol Chem 276(38):35947-52.

Patch AM, Flanagan SE, Boustred C, Hattersley AT, Ellard S. 2007. Mutations in the ABCC8 gene encoding the SUR1 subunit of the KATP channel cause transient neonatal diabetes, permanent neonatal diabetes or permanent diabetes diagnosed outside the neonatal period. Diabetes Obes Metab 9 Suppl 2:28-39.

Pearson ER, Flechtner I, Njolstad PR, Malecki MT, Flanagan SE, Larkin B, Ashcroft FM, Klimes I, Codner E, Iotova V and others. 2006. Switching from insulin to oral sulfonylureas in patients with diabetes due to Kir6.2 mutations. N Engl J Med 355(5):467-77.

Pena-Almazan S. 2015. Successful transition to sulfonylurea in neonatal diabetes, developmental delay, and seizures (DEND syndrome) due to R50P KCNJ11 mutation. Diabetes Res Clin Pract 108(1):e18-20.

Peranteau WH, Bathaii SM, Pawel B, Hardy O, Alavi A, Stanley CA, Adzick NS. 2007. Multiple ectopic lesions of focal islet adenomatosis identified by positron emission tomography scan in an infant with congenital hyperinsulinism. J Pediatr Surg 42(1):188-92.

Peranteau WH, Ganguly A, Steinmuller L, Thornton P, Johnson MP, Howell LJ, Stanley CA, Adzick NS. 2006. Prenatal diagnosis and postnatal management of diffuse congenital hyperinsulinism: a case report. Fetal Diagn Ther 21(6):515-8.

Petraitiene I, Barauskas G, Gulbinas A, Malcius D, Hussain K, Verkauskas G, Verkauskiene R. 2014. Congenital hyperinsulinism. Medicina (Kaunas) 50(3):190-5.

Philla KQ, Bauer AJ, Vogt KS, Greeley SA. 2013. Successful transition from insulin to sulfonylurea therapy in a patient with monogenic neonatal diabetes owing to a KCNJ11 F333L [corrected] mutation. Diabetes Care 36(12):e201.

Piccini B, Coviello C, Drovandi L, Rosangela A, Monzali F, Casalini E, Giglio S, Toni S, Dani C. 2018. Transient Neonatal Diabetes Mellitus in a Very Preterm Infant due to ABCC8 Mutation. AJP Rep 8(1):e39-e42.

Pinney SE, Ganapathy K, Bradfield J, Stokes D, Sasson A, Mackiewicz K, Boodhansingh K, Hughes N, Becker S, Givler S and others. 2013. Dominant form of congenital hyperinsulinism maps to HK1 region on 10q. Horm Res Paediatr 80(1):18-27.

Pinney SE, MacMullen C, Becker S, Lin YW, Hanna C, Thornton P, Ganguly A, Shyng SL, Stanley CA. 2008. Clinical characteristics and biochemical mechanisms of congenital hyperinsulinism associated with dominant KATP channel mutations. J Clin Invest 118(8):2877-86.

Polak M, Cave H. 2007. Neonatal diabetes mellitus: a disease linked to multiple mechanisms. Orphanet J Rare Dis 2:12.

Poovazhagi VT, S. 2014. Relapsing Transient Neonatal Diabetes Mellitus due to ABCC8 Mutation. Journal of Molecular and Genetic Medicine 8(4).

Powell PD, Bellanne-Chantelot C, Flanagan SE, Ellard S, Rooman R, Hussain K, Skae M, Clayton P, de Lonlay P, Dunne MJ and others. 2011. In Vitro Recovery of ATP-Sensitive Potassium Channels in {beta}-Cells From Patients With Congenital Hyperinsulinism of Infancy. Diabetes 60(4):1223-8.

Prado-Carro AM, Calzada-Hernandez J, Marin S, Cardona-Hernandez R, Oriola J, Nicolas M, Ramon-Krauel M. 2014. Patient with iDEND syndrome-related mutation. Diabetes Care 37(6):e123-4.

Pratt EB, Shyng SL. 2011. ATP activates ATP-sensitive potassium channels composed of mutant sulfonylurea receptor 1 and Kir6.2 with diminished PIP2 sensitivity. Channels (Austin) 5(4):314-9.

Pratt EB, Yan FF, Gay JW, Stanley CA, Shyng SL. 2009. Sulfonylurea receptor 1 mutations that cause opposite insulin secretion defects with chemical chaperone exposure. J Biol Chem 284(12):7951-9.

Proks P, Antcliff JF, Lippiat J, Gloyn AL, Hattersley AT, Ashcroft FM. 2004. Molecular basis of Kir6.2 mutations associated with neonatal diabetes or neonatal diabetes plus neurological features. Proc Natl Acad Sci U S A 101(50):17539-44.

Proks P, Arnold AL, Bruining J, Girard C, Flanagan SE, Larkin B, Colclough K, Hattersley AT, Ashcroft FM, Ellard S. 2006a. A heterozygous activating mutation in the sulphonylurea receptor SUR1 (ABCC8) causes neonatal diabetes. Hum Mol Genet 15(11):1793-800.

Proks P, de Wet H, Ashcroft FM. 2010. Activation of the K(ATP) channel by Mg-nucleotide interaction with SUR1. J Gen Physiol 136(4):389-405.

Proks P, Girard C, Ashcroft FM. 2005a. Functional effects of KCNJ11 mutations causing neonatal diabetes: enhanced activation by MgATP. Hum Mol Genet 14(18):2717-26.

Proks P, Girard C, Baevre H, Njolstad PR, Ashcroft FM. 2006b. Functional effects of mutations at F35 in the NH2-terminus of Kir6.2 (KCNJ11), causing neonatal diabetes, and response to sulfonylurea therapy. Diabetes 55(6):1731-7.

Proks P, Girard C, Haider S, Gloyn AL, Hattersley AT, Sansom MS, Ashcroft FM. 2005b. A gating mutation at the internal mouth of the Kir6.2 pore is associated with DEND syndrome. EMBO Rep 6(5):470-5.

Proverbio MC, Mangano E, Gessi A, Bordoni R, Spinelli R, Asselta R, Valin PS, Di Candia S, Zamproni I, Diceglie C and others. 2013. Whole genome SNP genotyping and exome sequencing reveal novel genetic variants and putative causative genes in congenital hyperinsulinism. PLoS One 8(7):e68740.

Qubbaj W, Al-Swaid A, Al-Hassan S, Awartani K, Deek H, Coskun S. 2011. First successful application of preimplantation genetic diagnosis and haplotyping for congenital hyperinsulinism. Reprod Biomed Online 22(1):72-9.

Rafiq M, Flanagan SE, Patch AM, Shields BM, Ellard S, Hattersley AT. 2008. Effective treatment with oral sulfonylureas in patients with diabetes due to sulfonylurea receptor 1 (SUR1) mutations. Diabetes Care 31(2):204-9.

Reimann F, Huopio H, Dabrowski M, Proks P, Gribble FM, Laakso M, Otonkoski T, Ashcroft FM. 2003. Characterisation of new KATP-channel mutations associated with congenital hyperinsulinism in the Finnish population. Diabetologia 46(2):241-9.

Rica I, Luzuriaga C, Perez de Nanclares G, Estalella I, Aragones A, Barrio R, Bilbao JR, Carles C, Fernandez C, Fernandez JM and others. 2007. The majority of cases of neonatal diabetes in Spain can be explained by known genetic abnormalities. Diabet Med 24(7):707-13.

Riveline JP, Rousseau E, Reznik Y, Fetita S, Philippe J, Dechaume A, Hartemann A, Polak M, Petit C, Charpentier G and others. 2012. Clinical and metabolic features of adult-onset diabetes caused by ABCC8 mutations. Diabetes Care 35(2):248-51.

Rozenkova K, Malikova J, Nessa A, Dusatkova L, Bjorkhaug L, Obermannova B, Dusatkova P, Kytnarova J, Aukrust I, Najmi LA and others. 2015. High Incidence of Heterozygous ABCC8 and HNF1A Mutations in Czech Patients With Congenital Hyperinsulinism. J Clin Endocrinol Metab 100(12):E1540-9.

Rubio-Cabezas O, Flanagan SE, Damhuis A, Hattersley AT, Ellard S. 2012. KATP channel mutations in infants with permanent diabetes diagnosed after 6 months of life. Pediatr Diabetes 13(4):322-5.

Russo C, Salina A, Aloi C, Iafusco D, Lorini R, d'Annunzio G. 2011a. Mother and daughter carrying the same KCNJ11 mutation but with a different response to switching from insulin to sulfonylurea. Diabetes Res Clin Pract 94(2):e50-2.

Russo L, Iafusco D, Brescianini S, Nocerino V, Bizzarri C, Toni S, Cerutti F, Monciotti C, Pesavento R, Iughetti L and others. 2011b. Permanent diabetes during the first year of life: multiple gene screening in 54 patients. Diabetologia 54(7):1693-701.

Ryan F, Devaney D, Joyce C, Nestorowicz A, Permutt MA, Glaser B, Barton DE, Thornton PS. 1998. Hyperinsulinism: molecular aetiology of focal disease. Arch Dis Child 79(5):445-7.

Sagen JV, Raeder H, Hathout E, Shehadeh N, Gudmundsson K, Baevre H, Abuelo D, Phornphutkul C, Molnes J, Bell GI and others. 2004. Permanent neonatal diabetes due to mutations in KCNJ11 encoding Kir6.2: patient characteristics and initial response to sulfonylurea therapy. Diabetes 53(10):2713-8.

Saint-Martin C, Arnoux JB, de Lonlay P, Bellanne-Chantelot C. 2011. KATP channel mutations in congenital hyperinsulinism. Semin Pediatr Surg 20(1):18-22.

Saint-Martin C, Zhou Q, Martin GM, Vaury C, Leroy G, Arnoux JB, de Lonlay P, Shyng SL, Bellanne-Chantelot C. 2015. Monoallelic ABCC8 mutations are a common cause of diazoxide-unresponsive diffuse form of congenital hyperinsulinism. Clin Genet 87(5):448-54.

Saito-Hakoda A, Yorifuji T, Kanno J, Kure S, Fujiwara I. 2012. Nateglinide is Effective for Diabetes Mellitus with Reactive Hypoglycemia in a Child with a Compound Heterozygous ABCC8 Mutation. Clin Pediatr Endocrinol 21(3):45-52.

Sakura H, Wat N, Horton V, Millns H, Turner RC, Ashcroft FM. 1996. Sequence variations in the human Kir6.2 gene, a subunit of the beta-cell ATP-sensitive K-channel: no association with NIDDM in while Caucasian subjects or evidence of abnormal function when expressed in vitro. Diabetologia 39(10):1233-6.

Salomon-Estebanez M, Flanagan SE, Ellard S, Rigby L, Bowden L, Mohamed Z, Nicholson J, Skae M, Hall C, Craigie R and others. 2016. Conservatively treated Congenital Hyperinsulinism (CHI) due to K-ATP channel gene mutations: reducing severity over time. Orphanet J Rare Dis 11(1):163.

Sandal T, Laborie LB, Brusgaard K, Eide SA, Christesen HB, Sovik O, Njolstad PR, Molven A. 2009. The spectrum of ABCC8 mutations in Norwegian patients with congenital hyperinsulinism of infancy. Clin Genet 75(5):440-8.

Sang Y, Ni G, Gu Y, Liu M. 2011. AV59M KCNJ11 gene mutation leading to intermediate DEND syndrome in a Chinese child. J Pediatr Endocrinol Metab 24(9-10):763-6.

Sang Y, Xu Z, Liu M, Yan J, Wu Y, Zhu C, Ni G. 2014a. Mutational analysis of ABCC8, KCNJ11, GLUD1, HNF4A and GCK genes in 30 Chinese patients with congenital hyperinsulinism. Endocr J 61(9):901-10.

Sang Y, Yang W, Yan J, Wu Y. 2014b. KCNJ11 gene mutation analysis on nine Chinese patients with type 1B diabetes diagnosed before 3 years of age. J Pediatr Endocrinol Metab 27(5-6):519-23.

Sebastian Ochoa A, Fernandez-Garcia D, Rozas Moreno P, Reyes-Garcia R, Lopez-Ibarra Lozano PJ, Fernandez Garcia JM. 2008. Neonatal diabetes: genetic implications in treatment. Endocrinol Nutr 55(3):142-5.

Senniappan S, Alexandrescu S, Tatevian N, Shah P, Arya V, Flanagan S, Ellard S, Rampling D, Ashworth M, Brown RE and others. 2014. Sirolimus therapy in infants with severe hyperinsulinemic hypoglycemia. N Engl J Med 370(12):1131-7.

Senniappan S, Sadeghizadeh A, Flanagan SE, Ellard S, Hashemipour M, Hosseinzadeh M, Salehi M, Hussain K. 2015. Genotype and phenotype correlations in Iranian patients with hyperinsulinaemic hypoglycaemia. BMC Res Notes 8:350.

Shah B, Breidbart E, Pawelczak M, Lam L, Kessler M, Franklin B. 2012a. Improved long-term glucose control in neonatal diabetes mellitus after early sulfonylurea allergy. J Pediatr Endocrinol Metab 25(3-4):353-6.

Shah P, Arya VB, Flanagan SE, Morgan K, Ellard S, Senniappan S, Hussain K. 2015. Sirolimus therapy in a patient with severe hyperinsulinaemic hypoglycaemia due to a compound heterozygous ABCC8 gene mutation. J Pediatr Endocrinol Metab 28(5-6):695-9.

Shah RP, Spruyt K, Kragie BC, Greeley SA, Msall ME. 2012b. Visuomotor performance in KCNJ11-related neonatal diabetes is impaired in children with DEND-associated mutations and may be improved by early treatment with sulfonylureas. Diabetes Care 35(10):2086-8.

Shahawy S, Chan NK, Ellard S, Young E, Shahawy H, Mace J, Peverini R, Chinnock R, Njolstad PR, Hattersley AT and others. 2011. A pathway to insulin independence in newborns and infants with diabetes. J Perinatol 31(8):567-70.

Sharma N, Crane A, Gonzalez G, Bryan J, Aguilar-Bryan L. 2000. Familial hyperinsulinism and pancreatic beta-cell ATP-sensitive potassium channels. Kidney Int 57(3):803-8.

Shaw ND, Majzoub JA. 2009. Permanent Neonatal Diabetes in a Patient with a KCNJ11/Q52R Mutation Accompanied by Intermittent Hypoglycemia and Liver Failure. Int J Pediatr Endocrinol 2009:453240.

Shemer R, Avnon Ziv C, Laiba E, Zhou Q, Gay J, Tunovsky-Babaey S, Shyng SL, Glaser B, Zangen DH. 2012. Relative expression of a dominant mutated ABCC8 allele determines the clinical manifestation of congenital hyperinsulinism. Diabetes 61(1):258-63.

Sherif EM, Abdelmaksoud AA, Elbarbary NS, Njolstad PR. 2013. An Egyptian case of congenital hyperinsulinism of infancy due to a novel mutation in KCNJ11 encoding Kir6.2 and response to octreotide. Acta Diabetol 50(5):801-5.

Shield JP, Flanagan SE, Mackay DJ, Harries LW, Proks P, Girard C, Ashcroft FM, Temple IK, Ellard S. 2008. Mosaic paternal uniparental isodisomy and an ABCC8 gene mutation in a patient with permanent neonatal diabetes and hemihypertrophy. Diabetes 57(1):255-8.

Shimomura K, de Nanclares GP, Foutinou C, Caimari M, Castano L, Ashcroft FM. 2010. The first clinical case of a mutation at residue K185 of Kir6.2 (KCNJ11): a major ATP-binding residue. Diabet Med 27(2):225-9.

Shimomura K, Flanagan SE, Zadek B, Lethby M, Zubcevic L, Girard CA, Petz O, Mannikko R, Kapoor RR, Hussain K and others. 2009. Adjacent mutations in the gating loop of Kir6.2 produce neonatal diabetes and hyperinsulinism. EMBO Mol Med 1(3):166-77.

Shimomura K, Girard CA, Proks P, Nazim J, Lippiat JD, Cerutti F, Lorini R, Ellard S, Hattersley AT, Barbetti F and others. 2006. Mutations at the same residue (R50) of Kir6.2 (KCNJ11) that cause neonatal diabetes produce different functional effects. Diabetes 55(6):1705-12.

Shimomura K, Horster F, de Wet H, Flanagan SE, Ellard S, Hattersley AT, Wolf NI, Ashcroft F, Ebinger F. 2007. A novel mutation causing DEND syndrome: a treatable channelopathy of pancreas and brain. Neurology 69(13):1342-9.

Shimomura K, Tusa M, Iberl M, Brereton MF, Kaizik S, Proks P, Lahmann C, Yaluri N, Modi S, Huopio H and others. 2013. A mouse model of human hyperinsulinism produced by the E1506K mutation in the sulphonylurea receptor SUR1. Diabetes 62(11):3797-806.

Shyng SL, Ferrigni T, Shepard JB, Nestorowicz A, Glaser B, Permutt MA, Nichols CG. 1998. Functional analyses of novel mutations in the sulfonylurea receptor 1 associated with persistent hyperinsulinemic hypoglycemia of infancy. Diabetes 47(7):1145-51.

Siklar Z, Ellard S, Okulu E, Berberoglu M, Young E, Savas Erdeve S, Mungan IA, Hacihamdioglu B, Erdeve O, Arsan S and others. 2011. Transient neonatal diabetes with two novel mutations in the KCNJ11 gene and response to sulfonylurea treatment in a preterm infant. J Pediatr Endocrinol Metab 24(11-12):1077-80.

Simsek E, Binay C, Flanagan SE, Ellard S, Hussain K, Kabukcuoglu S. 2013. Congenital hyperinsulinism presenting with different clinical, biochemical and molecular genetic spectra. Turk J Pediatr 55(6):584-90.

Singh P, Rao SC, Parikh R. 2014. Neonatal diabetes with intractable epilepsy: DEND syndrome. Indian J Pediatr 81(12):1387-8.

Skupien J, Malecki MT, Mlynarski W, Klupa T, Wanic K, Gach A, Solecka I, Sieradzki J. 2006. Assessment of insulin sensitivity in adults with permanent neonatal diabetes mellitus due to mutations in the KCNJ11 gene encoding Kir6.2. Rev Diabet Stud 3(1):17-20.

Slingerland AS, Hurkx W, Noordam K, Flanagan SE, Jukema JW, Meiners LC, Bruining GJ, Hattersley AT, Hadders-Algra M. 2008. Sulphonylurea therapy improves cognition in a patient with the V59M KCNJ11 mutation. Diabet Med 25(3):277-81.

Snider KE, Becker S, Boyajian L, Shyng SL, MacMullen C, Hughes N, Ganapathy K, Bhatti T, Stanley CA, Ganguly A. 2013. Genotype and phenotype correlations in 417 children with congenital hyperinsulinism. J Clin Endocrinol Metab 98(2):E355-63.

Sogno Valin P, Proverbio MC, Diceglie C, Gessi A, di Candia S, Mariani B, Zamproni I, Mangano E, Asselta R, Battaglia C and others. 2013. Genetic analysis of Italian patients with congenital hyperinsulinism of infancy. Horm Res Paediatr 79(4):236-42.

Someya T, Miki T, Sugihara S, Minagawa M, Yasuda T, Kohno Y, Seino S. 2000. Characterization of genes encoding the pancreatic beta-cell ATP-sensitive K+ channel in persistent hyperinsulinemic hypoglycemia of infancy in Japanese patients. Endocr J 47(6):715-22.

Stanik J, Gasperikova D, Paskova M, Barak L, Javorkova J, Jancova E, Ciljakova M, Hlava P, Michalek J, Flanagan SE and others. 2007. Prevalence of permanent neonatal diabetes in Slovakia and successful replacement of insulin with sulfonylurea therapy in KCNJ11 and ABCC8 mutation carriers. J Clin Endocrinol Metab 92(4):1276-82.

Stanik J, Lethby M, Flanagan SE, Gasperikova D, Milosovicova B, Lever M, Bullman H, Zubcevic L, Hattersley AT, Ellard S and others. 2008. Coincidence of a novel KCNJ11 missense variant R365H with a paternally inherited 6q24 duplication in a patient with transient neonatal diabetes. Diabetes Care 31(9):1736-7.

Stanley CA, Thornton PS, Ganguly A, MacMullen C, Underwood P, Bhatia P, Steinkrauss L, Wanner L, Kaye R, Ruchelli E and others. 2004. Preoperative evaluation of infants with focal or diffuse congenital hyperinsulinism by intravenous acute insulin response tests and selective pancreatic arterial calcium stimulation. J Clin Endocrinol Metab 89(1):288-96.

Stoy J, Greeley SA, Paz VP, Ye H, Pastore AN, Skowron KB, Lipton RB, Cogen FR, Bell GI, Philipson LH. 2008. Diagnosis and treatment of neonatal diabetes: a United States experience. Pediatr Diabetes 9(5):450-9.

Straub SG, Cosgrove KE, Ammala C, Shepherd RM, O'Brien RE, Barnes PD, Kuchinski N, Chapman JC, Schaeppi M, Glaser B and others. 2001. Hyperinsulinism of infancy: the regulated release of insulin by KATP channel-independent pathways. Diabetes 50(2):329-39.

Suchi M, MacMullen C, Thornton PS, Ganguly A, Stanley CA, Ruchelli ED. 2003. Histopathology of congenital hyperinsulinism: retrospective study with genotype correlations. Pediatr Dev Pathol 6(4):322-33.

Suchi M, MacMullen CM, Thornton PS, Adzick NS, Ganguly A, Ruchelli ED, Stanley CA. 2006. Molecular and immunohistochemical analyses of the focal form of congenital hyperinsulinism. Mod Pathol 19(1):122-9.

Sumnik Z, Kolouskova S, Wales JK, Komarek V, Cinek O. 2007. Sulphonylurea treatment does not improve psychomotor development in children with KCNJ11 mutations causing permanent neonatal diabetes mellitus accompanied by developmental delay and epilepsy (DEND syndrome). Diabet Med 24(10):1176-8.

Suzuki S, Makita Y, Mukai T, Matsuo K, Ueda O, Fujieda K. 2007. Molecular basis of neonatal diabetes in Japanese patients. J Clin Endocrinol Metab 92(10):3979-85.

Szymanowski M, Estebanez MS, Padidela R, Han B, Mosinska K, Stevens A, Damaj L, Pihan-Le Bars F, Lascouts E, Reynaud R and others. 2016. mTOR Inhibitors for the Treatment of Severe Congenital Hyperinsulinism: Perspectives on Limited Therapeutic Success. J Clin Endocrinol Metab 101(12):4719-4729.

Taberner P, Flanagan SE, Mackay DJ, Ellard S, Taverna MJ, Ferraro M. 2016. Clinical and genetic features of Argentinian children with diabetes-onset before 12months of age: Successful transfer from insulin to oral sulfonylurea. Diabetes Res Clin Pract 117:104-10.

Takagi M, Takeda R, Yagi H, Ariyasu D, Fukuzawa R, Hasegawa T. 2016. A case of transient neonatal diabetes due to a novel mutation in ABCC8. Clin Pediatr Endocrinol 25(4):139-141.

Takagi T, Furuta H, Miyawaki M, Nagashima K, Shimada T, Doi A, Matsuno S, Tanaka D, Nishi M, Sasaki H and others. 2013. Clinical and functional characterization of the Pro1198Leu ABCC8 gene mutation associated with permanent neonatal diabetes mellitus. J Diabetes Investig 4(3):269-73.

Takeda R, Takagi M, Miyai K, Shinohara H, Yagi H, Moritani M, Yokota I, Hasegawa Y. 2015. A case of a Japanese patient with neonatal diabetes mellitus caused by a novel mutation in the ABCC8 gene and successfully controlled with oral glibenclamide. Clin Pediatr Endocrinol 24(4):191-3.

Tammaro P, Ashcroft FM. 2007. A mutation in the ATP-binding site of the Kir6.2 subunit of the KATP channel alters coupling with the SUR2A subunit. J Physiol 584(Pt 3):743-53.

Tammaro P, Ashcroft FM. 2009. A cytosolic factor that inhibits KATP channels expressed in Xenopus oocytes by impairing Mg-nucleotide activation by SUR1. J Physiol 587(Pt 8):1649-56.

Tammaro P, Flanagan SE, Zadek B, Srinivasan S, Woodhead H, Hameed S, Klimes I, Hattersley AT, Ellard S, Ashcroft FM. 2008. A Kir6.2 mutation causing severe functional effects in vitro produces neonatal diabetes without the expected neurological complications. Diabetologia 51(5):802-10.

Tammaro P, Girard C, Molnes J, Njolstad PR, Ashcroft FM. 2005. Kir6.2 mutations causing neonatal diabetes provide new insights into Kir6.2-SUR1 interactions. Embo J 24(13):2318-30.

Taneja TK, Mankouri J, Karnik R, Kannan S, Smith AJ, Munsey T, Christesen HB, Beech DJ, Sivaprasadarao A. 2009. Sar1-GTPase-dependent ER exit of KATP channels revealed by a mutation causing congenital hyperinsulinism. Hum Mol Genet 18(13):2400-13.

Tanizawa Y, Matsuda K, Matsuo M, Ohta Y, Ochi N, Adachi M, Koga M, Mizuno S, Kajita M, Tanaka Y and others. 2000. Genetic analysis of Japanese patients with persistent hyperinsulinemic hypoglycemia of infancy: nucleotide-binding fold-2 mutation impairs cooperative binding of adenine nucleotides to sulfonylurea receptor 1. Diabetes 49(1):114-20.

Tarasov AI, Girard CA, Ashcroft FM. 2006. ATP sensitivity of the ATP-sensitive K+ channel in intact and permeabilized pancreatic beta-cells. Diabetes 55(9):2446-54.

Tarasov AI, Girard CA, Larkin B, Tammaro P, Flanagan SE, Ellard S, Ashcroft FM. 2007. Functional analysis of two Kir6.2 (KCNJ11) mutations, K170T and E322K, causing neonatal diabetes. Diabetes Obes Metab 9 Suppl 2:46-55.

Tarasov AI, Nicolson TJ, Riveline JP, Taneja TK, Baldwin SA, Baldwin JM, Charpentier G, Gautier JF, Froguel P, Vaxillaire M and others. 2008. A rare mutation in ABCC8/SUR1 leading to altered ATP-sensitive K+ channel activity and beta-cell glucose sensing is associated with type 2 diabetes in adults. Diabetes 57(6):1595-604.

Taschenberger G, Mougey A, Shen S, Lester LB, LaFranchi S, Shyng SL. 2002. Identification of a familial hyperinsulinism-causing mutation in the sulfonylurea receptor 1 that prevents normal trafficking and function of KATP channels. J Biol Chem 277(19):17139-46.

Thakkar AN, Muranjan MN, Karande S, Shah NS. 2014. Neonatal diabetes mellitus due to a novel ABCC8 gene mutation mimicking an organic acidemia. Indian J Pediatr 81(7):702-4.

Thakur S, Flanagan SE, Ellard S, Verma IC. 2011. Congenital hyperinsulinism caused by mutations in ABCC8 (SUR1) gene. Indian Pediatr 48(9):733-4.

Thewjitcharoen Y, Wanothayaroj E, Himathongkam T, Flanagan SE, Ellard S, Hattersley AT. 2014. Permanent neonatal diabetes misdiagnosed as type 1 diabetes in a 28-year-old female: a life-changing diagnosis. Diabetes Res Clin Pract 106(2):e22-4.

Thomas P, Ye Y, Lightner E. 1996a. Mutation of the pancreatic islet inward rectifier Kir6.2 also leads to familial persistent hyperinsulinemic hypoglycemia of infancy. Hum Mol Genet 5(11):1809-12.

Thomas PM, Cote GJ, Wohllk N, Haddad B, Mathew PM, Rabl W, Aguilar-Bryan L, Gagel RF, Bryan J. 1995. Mutations in the sulfonylurea receptor gene in familial persistent hyperinsulinemic hypoglycemia of infancy. Science 268(5209):426-9.

Thomas PM, Wohllk N, Huang E, Kuhnle U, Rabl W, Gagel RF, Cote GJ. 1996b. Inactivation of the first nucleotide-binding fold of the sulfonylurea receptor, and familial persistent hyperinsulinemic hypoglycemia of infancy. Am J Hum Genet 59(3):510-8.

Thornton PS, MacMullen C, Ganguly A, Ruchelli E, Steinkrauss L, Crane A, Aguilar-Bryan L, Stanley CA. 2003. Clinical and molecular characterization of a dominant form of congenital hyperinsulinism caused by a mutation in the high-affinity sulfonylurea receptor. Diabetes 52(9):2403-10.

Ting WH, Huang CY, Lo FS, Lee HC, Lin CL, Guo WL, Lee YJ. 2009. Improved diabetic control during oral sulfonylurea treatment in two children with permanent neonatal diabetes mellitus. J Pediatr Endocrinol Metab 22(7):661-7.

Tonini G, Bizzarri C, Bonfanti R, Vanelli M, Cerutti F, Faleschini E, Meschi F, Prisco F, Ciacco E, Cappa M and others. 2006. Sulfonylurea treatment outweighs insulin therapy in short-term metabolic control of patients with permanent neonatal diabetes mellitus due to activating mutations of the KCNJ11 (KIR6.2) gene. Diabetologia 49(9):2210-3.

Tornovsky S, Crane A, Cosgrove KE, Hussain K, Lavie J, Heyman M, Nesher Y, Kuchinski N, Ben-Shushan E, Shatz O and others. 2004. Hyperinsulinism of infancy: novel ABCC8 and KCNJ11 mutations and evidence for additional locus heterogeneity. J Clin Endocrinol Metab 89(12):6224-34.

Unal S, Gonulal D, Ucakturk A, Siyah Bilgin B, Flanagan SE, Gurbuz F, Tayfun M, Elmaogullari S, Arasli A, Demirel F and others. 2016. A Novel Homozygous Mutation in the KCNJ11 Gene of a Neonate with Congenital Hyperinsulinism and Successful Management with Sirolimus. J Clin Res Pediatr Endocrinol 8(4):478-481.

Valayannopoulos V, Vaxillaire M, Aigrain Y, Jaubert F, Bellanne-Chantelot C, Ribeiro MJ, Brunelle F, Froguel P, Robert JJ, Polak M and others. 2007. Coexistence in the same family of both focal and diffuse forms of hyperinsulinism. Diabetes Care 30(6):1590-2.

Vasanwala RF, Lim SH, Ellard S, Yap F. 2014. Neonatal Diabetes in a Singapore Children's Hospital: Molecular Diagnoses of Four Cases. Ann Acad Med Singapore 43(6):314-9.

Vaxillaire M, Dechaume A, Busiah K, Cave H, Pereira S, Scharfmann R, de Nanclares GP, Castano L, Froguel P, Polak M and others. 2007. New ABCC8 mutations in relapsing neonatal diabetes and clinical features. Diabetes 56(6):1737-41.

Vaxillaire M, Populaire C, Busiah K, Cave H, Gloyn AL, Hattersley AT, Czernichow P, Froguel P, Polak M. 2004. Kir6.2 mutations are a common cause of permanent neonatal diabetes in a large cohort of French patients. Diabetes 53(10):2719-22.

Vedovato N, Cliff E, Proks P, Poovazhagi V, Flanagan SE, Ellard S, Hattersley AT, Ashcroft FM. 2016. Neonatal diabetes caused by a homozygous KCNJ11 mutation demonstrates that tiny changes in ATP sensitivity markedly affect diabetes risk. Diabetologia 59(7):1430-6.

Vendramini MF, Gurgel LC, Moises RS. 2010. Long-term response to sulfonylurea in a patient with diabetes due to mutation in the KCNJ11 gene. Arq Bras Endocrinol Metabol 54(8):682-4.

Verkarre V, Fournet JC, de Lonlay P, Gross-Morand MS, Devillers M, Rahier J, Brunelle F, Robert JJ, Nihoul-Fekete C, Saudubray JM and others. 1998. Paternal mutation of the sulfonylurea receptor (SUR1) gene and maternal loss of 11p15 imprinted genes lead to persistent hyperinsulinism in focal adenomatous hyperplasia. J Clin Invest 102(7):1286-91.

Vieira TC, Bergamin CS, Gurgel LC, Moises RS. 2010. Hyperinsulinemic hypoglycemia evolving to gestational diabetes and diabetes mellitus in a family carrying the inactivating ABCC8 E1506K mutation. Pediatr Diabetes 11(7):505-8.

Wagner VM, Kremke B, Hiort O, Flanagan SE, Pearson ER. 2009. Transition from insulin to sulfonylurea in a child with diabetes due to a mutation in KCNJ11 encoding Kir6.2--initial and long-term response to sulfonylurea therapy. Eur J Pediatr 168(3):359-61.

Wambach JA, Marshall BA, Koster JC, White NH, Nichols CG. 2010. Successful sulfonylurea treatment of an insulin-naive neonate with diabetes mellitus due to a KCNJ11 mutation. Pediatr Diabetes 11(4):286-8.

Wang SY, Zhang LJ, He ZQ, Tian Q, Li XD. 2012. [Neonatal diabetes mellitus caused by KCNJ11 mutation: a case report]. Zhongguo Dang Dai Er Ke Za Zhi 14(1):73-5.

Winkler M, Lutz R, Russ U, Quast U, Bryan J. 2009. Analysis of two KCNJ11 neonatal diabetes mutations, V59G and V59A, and the analogous KCNJ8 I60G substitution: differences between the channel subtypes formed with SUR1. J Biol Chem 284(11):6752-62.

Wu MK, de Kock L, Conwell LS, Stewart CJ, King BR, Choong CS, Hussain K, Sabbaghian N, MacRae IJ, Fabian MR and others. 2016. Functional characterization of multiple DICER1 mutations in an adolescent. Endocr Relat Cancer 23(2):L1-5.

Xiao X, Wang T, Li W, Song H, Gong C, Diao C, Yu M, Yuan T, Zhang Y, Sun X and others. 2009. Transfer from insulin to sulfonylurea treatment in a chinese patient with permanent neonatal diabetes mellitus due to a KCNJ11 R201H mutation. Horm Metab Res 41(7):580-2.

Xu ZD, Yu HF, Sang YM, Zhang YN, Yan J, Wu YJ, Zhu C, Ni GC. 2013. [ABCC8, KCNJ11 and GLUD1 gene mutation analysis in congenital hyperinsulinism pedigree]. Zhonghua Yi Xue Za Zhi 93(14):1089-92.

Xu ZD, Zhang W, Liu M, Wang HM, Hui PP, Liang XJ, Yan J, Wu YJ, Sang YM, Zhu C and others. 2018. Analysis on the pathogenic genes of 60 Chinese children with congenital hyperinsulinemia. Endocr Connect 7(12):1251-1261.

Yadav D, Dhingra B, Kumar S, Kumar V, Dutta AK. 2012. Persistent hyperinsulinemic hypoglycemia of infancy. J Pediatr Endocrinol Metab 25(5-6):591-3.

Yamazaki M, Sugie H, Oguma M, Yorifuji T, Tajima T, Yamagata T. 2017. Sulfonylurea treatment in an infant with transient neonatal diabetes mellitus caused by an adenosine triphosphate binding cassette subfamily C member 8 gene mutation. Clin Pediatr Endocrinol 26(3):165-169.

Yan F, Lin CW, Weisiger E, Cartier EA, Taschenberger G, Shyng SL. 2004. Sulfonylureas correct trafficking defects of ATP-sensitive potassium channels caused by mutations in the sulfonylurea receptor. J Biol Chem 279(12):11096-105.

Yan FF, Lin YW, MacMullen C, Ganguly A, Stanley CA, Shyng SL. 2007. Congenital hyperinsulinism associated ABCC8 mutations that cause defective trafficking of ATP-sensitive K+ channels: identification and rescue. Diabetes 56(9):2339-48.

Yang Y, Shi W, Chen X, Cui N, Konduru AS, Shi Y, Trower TC, Zhang S, Jiang C. 2011. Molecular basis and structural insight of vascular K(ATP) channel gating by S-glutathionylation. J Biol Chem 286(11):9298-307.

Yen CF, Huang CY, Chan CI, Hsu CH, Wang NL, Wang TY, Lin CL, Ting WH. 2016. Successful treatment of a newborn with congenital hyperinsulinism having a novel heterozygous mutation in the ABCC8 gene using subtotal pancreatectomy. Ci Ji Yi Xue Za Zhi 28(4):162-165.

Yorifuji T, Kawakita R, Hosokawa Y, Fujimaru R, Matsubara K, Aizu K, Suzuki S, Nagasaka H, Nishibori H, Masue M. 2013. Efficacy and safety of long-term, continuous subcutaneous octreotide infusion for patients with different subtypes of KATP-channel hyperinsulinism. Clin Endocrinol (Oxf) 78(6):891-7.

Yorifuji T, Kawakita R, Nagai S, Sugimine A, Doi H, Nomura A, Masue M, Nishibori H, Yoshizawa A, Okamoto S and others. 2011. Molecular and clinical analysis of Japanese patients with persistent congenital hyperinsulinism: predominance of paternally inherited monoallelic mutations in the KATP channel genes. J Clin Endocrinol Metab 96(1):E141-5.

Yorifuji T, Nagashima K, Kurokawa K, Kawai M, Oishi M, Akazawa Y, Hosokawa M, Yamada Y, Inagaki N, Nakahata T. 2005. The C42R mutation in the Kir6.2 (KCNJ11) gene as a cause of transient neonatal diabetes, childhood diabetes, or later-onset, apparently type 2 diabetes mellitus. J Clin Endocrinol Metab 90(6):3174-8.

Zhang M, Chen X, Shen S, Li T, Chen L, Hu M, Cao L, Cheng R, Zhao Z, Luo F. 2015a. Sulfonylurea in the treatment of neonatal diabetes mellitus children with heterogeneous genetic backgrounds. J Pediatr Endocrinol Metab 28(7-8):877-84.

Zhang W, Liu L, Wen Z, Cheng J, Li C, Li X, Niu H, Wang F, Sheng H, Liu H. 2015b. A compound heterozygous mutation of ABCC8 gene causing a diazoxide-unresponsive congenital hyperinsulinism with an atypical form: Not a focal lesion in the pancreas reported by (1)(8)F-DOPA-PET/CT scan. Gene 572(2):222-6.

Zhou Q, Chen PC, Devaraneni PK, Martin GM, Olson EM, Shyng SL. 2014. Carbamazepine inhibits ATP-sensitive potassium channel activity by disrupting channel response to MgADP. Channels (Austin) 8(4):376-82.

Zhou Q, Garin I, Castano L, Argente J, Munoz-Calvo MT, Perez de Nanclares G, Shyng SL. 2010. Neonatal diabetes caused by mutations in sulfonylurea receptor 1: interplay between expression and Mg-nucleotide gating defects of ATP-sensitive potassium channels. J Clin Endocrinol Metab 95(12):E473-8.

Zung A, Glaser B, Nimri R, Zadik Z. 2004. Glibenclamide treatment in permanent neonatal diabetes mellitus due to an activating mutation in Kir6.2. J Clin Endocrinol Metab 89(11):5504-7.

Zwaveling-Soonawala N, Hagebeuk EE, Slingerland AS, Ris-Stalpers C, Vulsma T, van Trotsenburg AS. 2011. Successful transfer to sulfonylurea therapy in an infant with developmental delay, epilepsy and neonatal diabetes (DEND) syndrome and a novel ABCC8 gene mutation. Diabetologia 54(2):469-71.
